# Towards a taxonomic revision of the genus *Cyrtochilum* (Orchidaceae) in Northwestern South America (Northern Peru, Ecuador, Colombia, Venezuela)

**DOI:** 10.7717/peerj.8566

**Published:** 2020-02-14

**Authors:** Dariusz L. Szlachetko, Marta Kolanowska

**Affiliations:** 1 Department of Plant Taxonomy and Nature Conservation, Faculty of Biology, University of Gdansk, Gdańsk, Poland; 2 Department of Geobotany and Plant Ecology, Faculty of Biology and Environmental Protection, University of Lodz, Lodz, Poland; 3 Department of Biodiversity Research, Global Change Research Institute AS CR, Brno, Czech Republic

**Keywords:** Neotropics, Biodiversity, Orchids, Conservation status, New species

## Abstract

This article is a presentation of taxonomic diversity of the orchid genus *Cyrtochilum* in Northwestern South America. The morphological characteristics of over 90 species occurring in northern Peru, Ecuador, Colombia and Venezuela are presented together with illustrations of their floral segments. Information about the distribution of each taxon is provided. Ten morphologically consistent groups have been delineated to facilitate identification of *Cyrtochilum* representatives in the studied area. Keys for determination of species within each group are provided. Seven new species of *Cyrtochilum* are described and one new combination is proposed.

## Introduction

Northwestern South America is one of the most diverse regions of the world in terms of physiography, habitat variation and species richness. About 10% of all known ecoregions are located in just four countries: Colombia, Ecuador, Peru and Venezuela and two important biodiversity hotspots, Tumbes-Chocó-Magdalena and Tropical Andes, are recognised within this region. The occurrence of about 20,000 vascular plant species was reported from Peru that includes about 2,020 Orchidaceae representatives (Brako & Zarucchi, 1993; Rodríguez et al., 2006). Zelenko & Bermúdez (2009) however, listed 2,873 orchid species for this country. The *Catalogue of the Vascular Plants of Ecuador* documented 15,901 plant species and listed an additional 186 species that are expected to occur (Jørgensen & León-Yánez, 1999). Over 4,016 orchid species were reported from Ecuador (Endara & León-Yánez, 2006). In Venezuela about 15,820 vascular plant species are found (De Stefano et al., 2009) including about 1,800 Orchidaceae representatives (Carnevali, Gerlach & Romero-González, 2008). The highest diversity is observed in Colombia where 24,528 vascular plant species were found and the occurrence of almost 3,600 orchid species was reported (Bernal, Gradstein & Celis, 2016). However, Szlachetko & Kolanowska (2017a) supposed that as much as 5,000 orchid species may grow in this country. The extraordinary diversity of Orchidaceae is increasing each year as new species from all four countries are described (Cornejo & Dodson, 2011; De Azevedo & Van den Berg, 2012; Damián & Karremans, 2016; Damián & Ormerod, 2016; Kolanowska & Szlachetko, 2014; Kolanowska, Szlachetko & Medina Trejo, 2016; Leopardi, Carnevali & Romero-González, 2012; Fernández-Concha & Cetzal-Ix, 2012).

Unfortunately, while new discoveries are published frequently, not many comprehensive studies on South American orchid genera were conducted and numerous previous revisions are out-of-date. The aim of the current study was to present a synopsis of *Cyrtochilum* in NW South America.

The genus *Cyrtochilum* was described in the early XIXth century (Kunth, 1816) and typified over 150 years later (Garay, 1974). *Cyrtochilum* was initially accepted by Lindley (1833), who eventually changed his opinion and transferred these orchids to *Oncidium* Sw. (Lindley, 1838). The separateness of the genus was contested by Reichenbach who assigned plants corresponding to this taxon to *Oncidium* or *Odontoglossum* Kunth. By the XXth century only one taxonomist, Friedrich Kraenzlin, recognised *Cyrtochilum* as separate genus (Kraenzlin, 1917), but this concept was not acknowledged by other orchidologists. In 2001 Dalström revitalised the genus, proposing several transfers and describing new species (Dalström, 2001, 2010). He included here representatives of *Odontoglossum*, *Dasyglossum* Königer & Schildh., *Trigonochilum* Königer & Schildh., *Siederella* Szlach., Mytnik, Górniak & Romowicz, *Rusbyella* Rolfe *ex* Rusby, *Buesiella* C. Schweinf., and *Neodryas* Rchb. f. In 2010, Dalström recognised about 140 species in *Cyrtochilum* and several novelties were described more recently (Dalström, 2015; Dalström & Deburghgraeve, 2015; Szlachetko & Kolanowska, 2014a, 2014b, 2017b). The broad concept of the genus was also postulated by Neubig et al. (2012) who, however, included most *Odontoglossum* species in *Oncidium*.

The morphological integrity of *Cyrtochilum s.l*. (Chase, 2009; Dalström, 2010; Neubig et al., 2012) was recently questioned by Szlachetko et al. (2017) who proposed to maintain a narrow concept of this genus.

## Materials and Methods

Herbarium material of *Cyrtochilum s.str*. is not commonly found in herbaria. We estimate that about 70% of specimens represented in collections involve only a fragment of inflorescence. It is understandable, if we consider a significant size of the plant, of which leaves are often 50–60 cm long, and an outstandingly long inflorescence, which can reach 2–3 m, or more. Unfortunately, that limits the information of these characters, what can be especially annoying in the case of plants with alien flowers, with segments and gynostemium details clearly different from those known in existing species. In such situations we decided to describe them as new even if the collection lacks vegetative parts.

A total of about 500 *Cyrtochilum s.str*. specimens deposited at or loaned from herbaria AMES, COL, K, MO, P, UGDA, US and W were examined according to the standard procedures. Plant material was not collected in the natural habitats of *Cyrtochilum* during field studies. The list of holotypes is presented in Table S1. This part of our work constituted the background for the descriptions of species we present below. Only in exceptional cases we made use of data given in the literature, which is transcribed into the text. Herbaria acronyms are cited in this article according to ‘Index Herbariorum’ (Thiers, 2018). Each studied specimen was photographed and the data from the label were taken. The pseudobulb shape and surface as well as number and form of subtending sheaths were examined. This was followed by examination of leaves number, shape and size. Flowers, floral bracts and ovaries of each specimen were studied using a stereomicroscope after softening them in the boiling water. The lip shape, its curvature, and the angle of the connexion with the gynostemium are the most important floral characters used for determination of the species. It seems that the structure of the lip callus, its composition of various kinds of projections, keels, lobes and knobs, as well as its surface (glabrous vs papillate) can be very useful key characters, although very difficult to use. The form of tepals (e.g. presence/absence of a claw and claw appendages, lamina form) can separate groups of species and in some cases allows naming the particular examined specimen. Last but not least are characters of the gynostemium, especially attendance and form of lateral, elongate appendages present usually on both sides of stigma base. All of the above mentioned characters can discriminate species of *Cyrtochilum s.str*.

In this article *Cyrtochilum* was divided into 10 morphologically consistent groups in order to facilitate identification of the species. In each group taxa are listed according to the similarity levels that allow easy comparison of particular taxa.

### Nomenclature

The electronic version of this article in Portable Document Format will represent a published work according to the International Code of Nomenclature for algae, fungi and plants (ICN; Turland et al., 2018), and hence the new names contained in the electronic version are effectively published under that Code from the electronic edition alone. In addition, new names contained in this work that have been issued with identifiers by IPNI will eventually be made available to the Global Names Index. Several new species are described here based on single of very few collections, however, we expect that future local research will reveal the existence of additional populations of these taxa. The IPNI Life Science Identifiers (LSIDs) can be resolved and the associated information viewed through any standard web browser by appending the LSID contained in this publication to the prefix ‘http://ipni.org/’. The online version of this work is archived and available from the following digital repositories: PeerJ, PubMed Central and CLOCKSS.

## Results

**Taxonomic treatment**

***Cyrtochilum*** Kunth *in* F.W.H. von Humboldt, A.J.A. Bonpland & C.S. Kunth, Nov. Gen. Sp. 1: 349. 1816. TYPE (Garay, 1974): *Cyrtochilum undulatum* Kunth.

**Figure 1:**
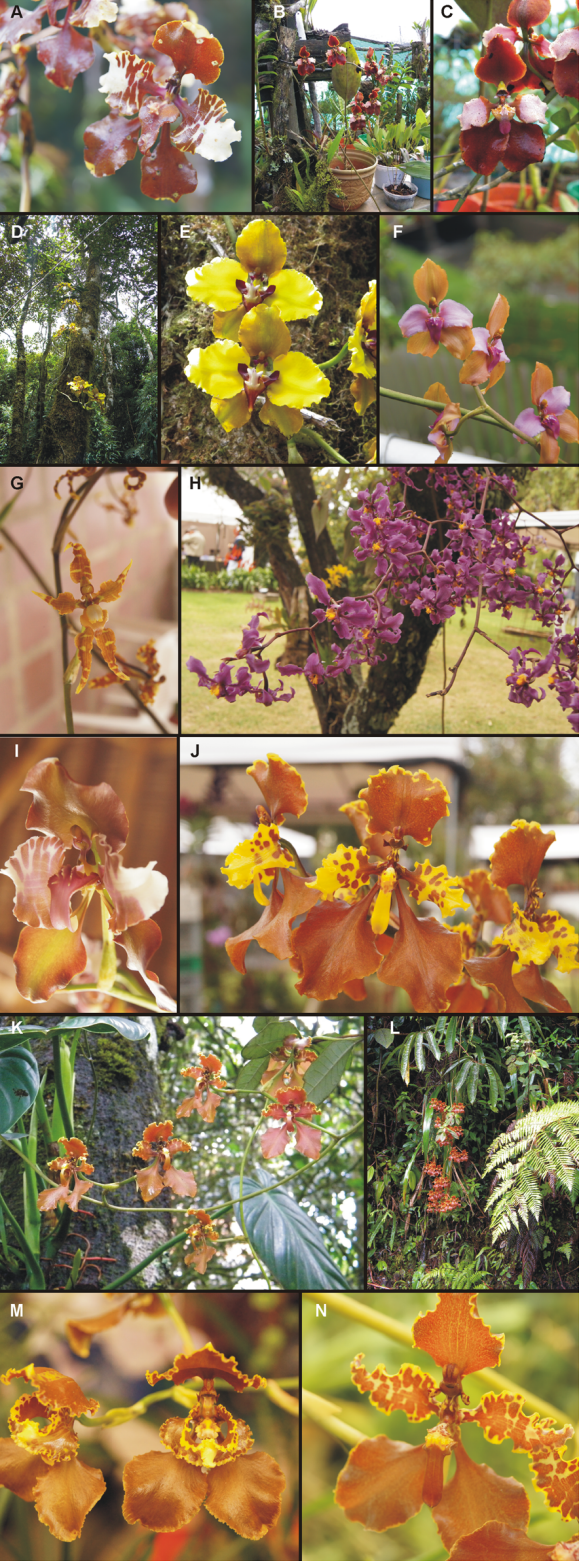
Photos of *Cyrtochilum* representatives. (A) *C. superbiens* (photo: Luis Carlos Piña *&* Maria Luisa Hincapié); (B) *C. pastasae* (photo: Marta Kolanowska); (C) *C. pastasae* (photo: Marta Kolanowska); (D) *C. maranthum* (photo: Marta Kolanowska); (E) *C. maranthum* (photo: Marta Kolanowska); (F) *C. orgyale* (photo: Luis Carlos Piña *&* Maria Luisa Hincapié); (G) *C. divaricatum* (photo: Luis Carlos Piña *&* Maria Luisa Hincapié); (H) *C. ioplocon* (photo: Luis Carlos Piña *&* Maria Luisa Hincapié); (I) *C. halteratum* (photo: Luis Carlos Piña *&* Maria Luisa Hincapié); (J) *C. tetracopis* (photo: Luis Carlos Piña *&* Maria Luisa Hincapié); (K) *C. trifurcatum* (photo: Marta Kolanowska); (L) *C. aemulum* (photo: Marta Kolanowska); (M) *C. annulare* (photo: Luis Carlos Piña *&* Maria Luisa Hincapié); (N) *C. gargantua* (photo: Luis Carlos Piña *&* Maria Luisa Hincapié).

Epiphytic, lithophytic or terrestrial plants. Pseudobulbs ovoid, usually elliptic in cross-section, distributed on elongate creeping or ascending rhizome, sometimes caespitose, but clusters of pseudobulbs usually distantly remote along the rhizome. Rhizome enclothed in bladeless sheaths. Pseudobulbs enveloped with 2–6 foliaceous sheaths. Leaves 1–4 per pseudobulb, conduplicate, articulate, linear-oblanceolate, acute. Inflorescence usually very long, flexuose, branched, branches with few to many flowers. Flowers resupinate, showy, white, yellow, pink, brown, purple or of mixed colours. Tepals free (lateral sepals sometimes shortly connate at the base), prominently unguiculate, similar in size and shape or petals shorter and wider. Lip triangular to ovate, rarely hastate to panduriform, reflexed near the middle to expose a complex callus, consisting often of digitate, tuberculate or horned, glabrous or papillate projections. Gynostemium gently sigmoid to erect above basal junction with the lip, elongated, slender, clavate, usually forming right angle with the lip. Anther subventral, incumbent, operculate, ellipsoid-ovoid. Pollinia 2, oblong ellipsoid, hard, unequally and deeply cleft, empty inside. Stigma large, elliptic, deeply concave. Rostellum short. Viscidium single, relatively large, elliptic, very thick, concave in the centre of the outer surface. Tegula single, very small, transversely elliptic-obtriangular, obscurely bilobulate at the apex, thin, lamellate. Rostellum remnant with oblique shallowly concave plate at the apex, canaliculate on the upper surface. Capsule triangular (Fig. 1).

The genus embraces more than 100 species restricted in distribution to the central and northern Andes. Little is known about the pollination of *Cyrtochilum*, but Van der Pijl & Dodson (1966) noted *Bombus hortulanum* and *Centris* bees pollinating *C. macranthum*.

The authors consider the lip shape and structure of callus (e.g. number and form of keels/lamellae and additional appendages) as well as tepals form as the most important characters differentiating species of *Cyrtochilum s.str*. Whether they are really discriminative characters requires further studies, including genetic analyses. Based on these features, to facilitate plant identification, we divided the genus into 10 artificial, morphological groups and the keys to species determination are presented within the groups.

### Artificial Key to the Groups

1 Lip shield-like, more or less equally long and wide(10) *Volubile*-group
1* Lip not as above, much longer than wide2
2. Lip prominently 3-lobed at the base, hence deeply hastate in general outline, lateral lobes at least 1/3 as long as the middle lobe3
2* Lip unlobed or with inconspicuous lateral lobes, not as above4
3 Lip lateral lobes obliquely rhombic to triangular, acute to obtuse(1) *Minax*-group
3* Lip lateral lobes elliptic, with rounded apices(9) *Ioplocon*-group
4 Lip pandurate in general outline, expanded apically to form a wide plate, wider than basal part(2) *Diceratum*-group
4* Lip not as above, basal part wider than apical one5
5 Lip more or less triangular or ovate in outline6
5* Lip oblong to ligulate in outline7
6 Dorsal sepal with cordate base, lamina wider than long(3) *Tetracopis-*group
6* Dorsal sepal with broadly cuneate base, lamina usually longer than wide(4) *Detortum-*group
7 Lip callus simple, consisting of 1–3 plain keels, without any additional outgrowth8
7* Lip callus complicated, with main keels and additional thickenings9
8 Lip basal part obscurely deltoid, apical part attenuate towards acute to obtuse apex(5) *Zebrinum*-group
8* Lip more or less ligulate, apex emarginated(6) *Annulare-*group
9 Lip oblong-ovate, callus consisting of 3 main lamellae variously modified(7) *Trilamellatum*-group
9* Lip narrow, callus in form of a single ridge(8) *Superbiens*-group

**(1) *Minax*-group**

Lip prominently 3-lobed just above the base, hastate or triangular in general outline, widest near the base, lateral lobes very prominent, obliquely rhombic to triangular. Gynostemium connate with the lip at the base only, then upcurved, more or less sigmoid.

Most of the 19 species of this group occur in Peru, with some representatives reported from Colombia, Ecuador and Bolivia.

### Key to the species

1 Lip callus papillate2
1* Lip callus glabrous5
2 Lip pandurate in general outline, apical part clawed, gradually transforming above cuneate base into flabellate blade*C. cryptocopis*
2* Lip hastate in general outline, apical part narrow, ligulate to lanceolate, acute to obtuse, but never flabellate3
3 Gynostemium lateral appendages obliquely oblanceolate, subacute*C. hastatum*
3* Gynostemium lateral appendages more or less obtriangular in general outline, apically truncate4
4 Gynostemium with an additional pair of curved down, falcate, acute projections just below stigma*C. gargantua*
4* Gynostemium without additional appendages*C. ionodon*
5 Basal part of the lip middle lobe papillate*C. minax*
5* Lip glabrous6
6 Lip middle lobe horseshoe-shaped*C. betancurii*
6* Lip middle lobe not as above7
7 Dorsal sepal about 3–4 times longer than wide*C. methonica*
7* Dorsal sepal about as long as wide8
8 Gynostemium with appendages composed of several series of toothed filaments*C. ramiro-medinae*
8* Gynostemium appendages without toothed filaments9
9 Gynostemium with two pairs of appendages*C. trifurcatum*
9* Gynostemium with a pair of appendages only10
10 Petals with strongly undulate-serrate margins*C. serratum*
10* Petals undulate, but never serrate11
11 Lip lateral lobes truncate, deeply incised in basal third*C. kienastianum*
11* Lip lateral lobes entire12
12 Lip lateral lobes obtuse to truncate13
12* Lip lateral lobes more or less triangular with acute apex15
13 Lip lateral lobes obliquely obovate, weakly serrate or undulate on margin14
13* Lip lateral lobes broadly linear, elongate, obliquely rounded at apex*C. deburghgraeveanum*
14 Lip callus of three low, longitudinal keels, emerging from the base and extending to near the middle lobe, spreading, the lateral pair ends in raised, blunt, angulate keels, while the central keel continues with two new emerging, lateral, spreading keels on each side of the angular, nose-like apex*C. ruizii*
14* Lip callus consisting of a single, high, horn-like keel, flanked by two smaller appendages and with an additional outgrowths*C. hastiferum*
15 Gynostemium with a pair of falcate, acute and upcurved wings*C. cordatum*
15* Gynostemium with a pair of apically truncate or bidentate wings16
16 Blade of dorsal sepal ovate-triangular, base cuneate*C. pastorellii*
16* Blade of dorsal sepal broadly ovate to transversely elliptic, base more or less cordate17
17 Lip callus rectangular-pandurate in outline in front view*C. lamelligerum*
17* Lip callus not as above18
18 Lateral keels of lip callus erect*C. macranthum*
18* Lateral keels of lip callus laterally spreading*C. xanthocinctum*

***Cyrtochilum trifurcatum*** (Lindl.) Kraenzl., Notizbl. Bot. Gart. Berlin-Dahlem 7: 91. 1917. ≡ *Oncidium trifurcatum* Lindl., Ann. Mag. Nat. Hist. 15: 384. 1845. TYPE: Peru or Ecuador? *Hartweg s.n*. (holotype, K).

**Figure 2:**
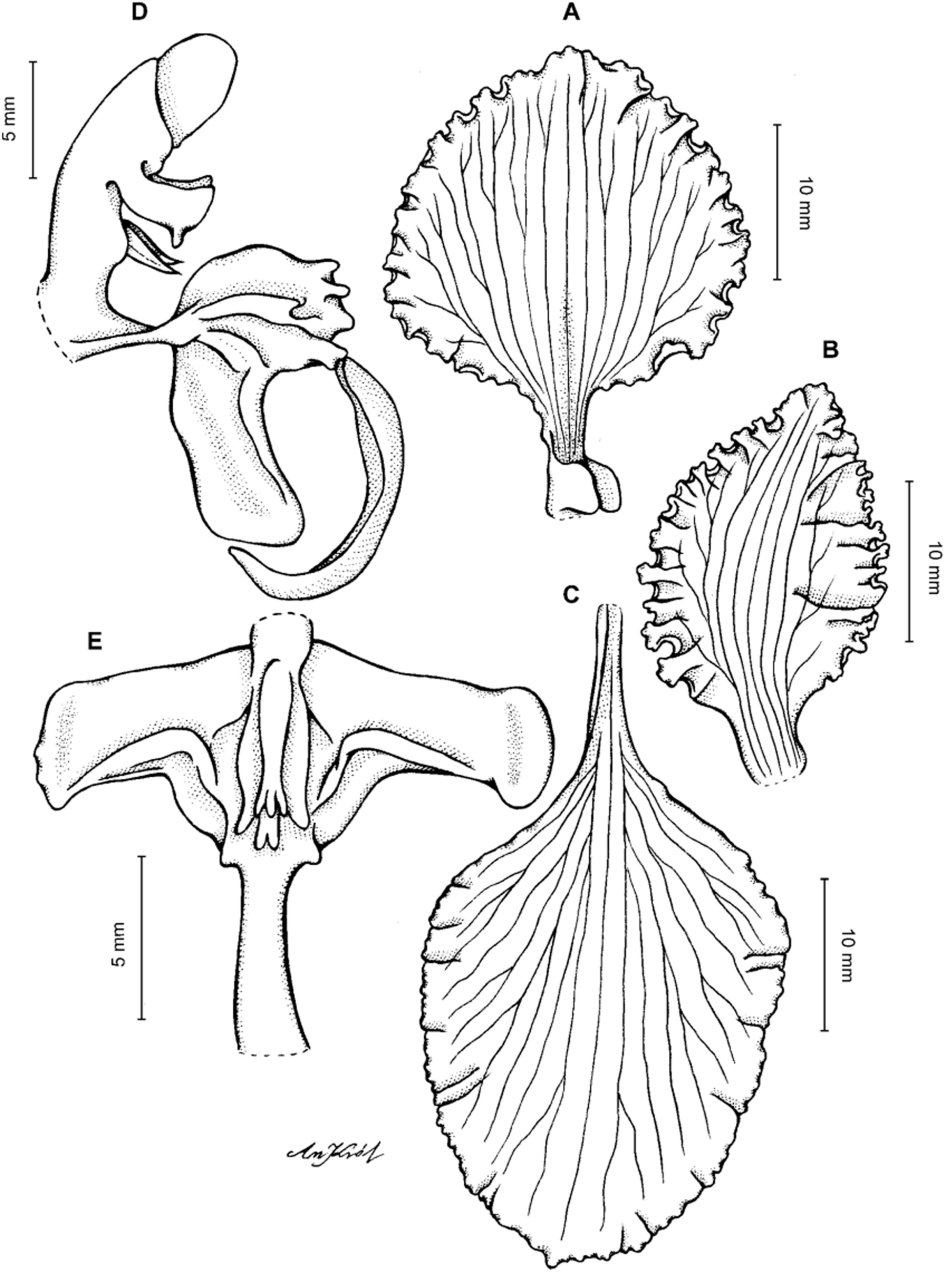
*Cyrtochilum trifurcatum* (Lindl.) Kraenzl. (A) Dorsal sepal; (B) Petal; (C) Lateral sepal; (D) Lip and gynostemium, side view; (E) Lip basal part. Drawn by Anna Król from *Ospina 142a* (COL).

**Figure 3:**
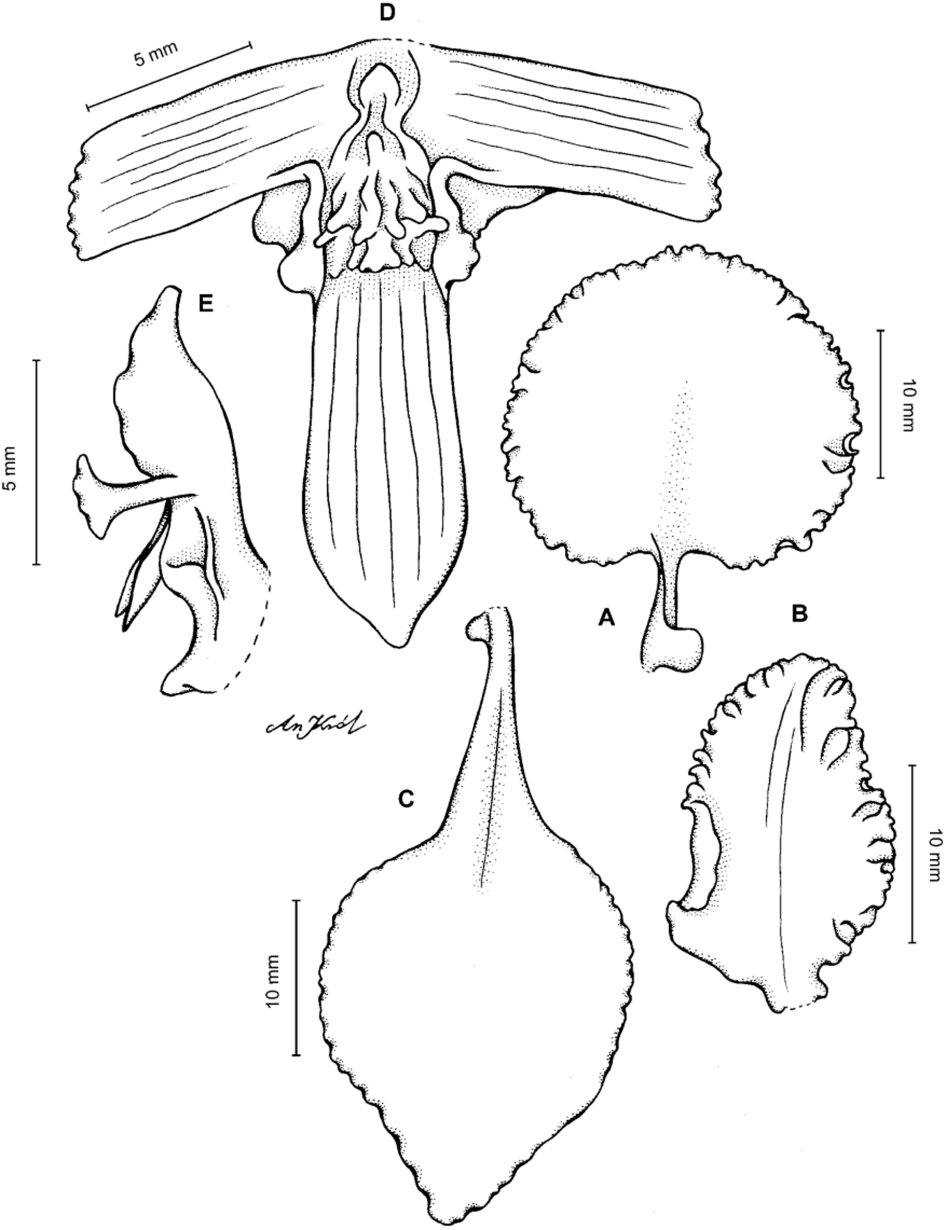
*Cyrtochilum trifurcatum* (Lindl.) Kraenzl. (A) Dorsal sepal; (B) Petal; (C) Lateral sepal; (D) Lip; (E) Gynostemium, side view. Drawn by Anna Król from *van der Werff & Palacios 9263* (AMES).

Rhizome ascending. Pseudobulbs 9–14 cm long, 1.6–3 cm in diameter, narrowly ovoid, smooth, comewhat compressed, subtended basally by leafy sheaths, 1–2-foliate. Leaves up to 70 cm long and 5 cm wide, oblanceolate, acute. Inflorescence up to 3 m long, paniculate, wiry, with well-spaced few-flowered branches. Flowers medium-sized, conspicuous, sepals brown to reddish-brown with yellow apex, petals brown to reddish-brown with yellow irregular stripes and apex, lip brown with yellow callus. Floral bracts 15–20 mm long. Pedicel and ovary 40 mm long. Dorsal sepal clawed; claw 6–9 mm long, narrow, canaliculate, with prominent rhombic wings just above the base; blade 17–23 mm long, 19–26 mm wide, suborbicular to transversely elliptic, margins strongly undulate, base subcordate or truncate, apex rounded. Petals shortly clawed; claw about 3–5 mm long, wide; blade 20 mm long, 15–19 mm wide when spread, obliquely elliptic-ovate, base cuneate, apex obtuse, margins crenate and strongly undulate. Lateral sepals prominently clawed; claw 10–14 mm long, narrow, with obscure basal wing; blade 27–35 mm long, 18–28 mm wide, obliquely elliptic-ovate in general outline, cuneate at the base, apex rounded, margins crenate, obscurely to strongly undulate. Lip 19–21 mm long in total, hastate in general outline, 3-lobed just above the truncate base, gently curved in natural position exposing large callus; basal part 4–5 mm long, 10–16 mm wide across, transversely rectangular in general outline, apex obliquely truncate; callus glabrous, consisting of a prominent, median ridge dissected at the apex and flanked by smaller lateral ridges, with an additional thickenings parallel to the apical margin of the basal part of the lip; apical part 12–13 mm long, 3–6 mm wide, ligulate, apex obtuse to rounded. Gynostemium 10–12 mm long, gently arcuate, connate basally with the lip, lateral appendages oblong, apically truncate with small projection on the lower margin, hence appearing obscurely bilobed, not reaching the anther base, and with an additional pair of linear pending acute projections just below stigma (Figs. 2 and 3).

*Ecology*: Epiphyte or terrestrial in lower and upper montane, very humid forest.

*Distribution*: Colombia, Ecuador, Peru (Dalström, 2010). Alt. 1850–3200 m.

*Notes*: *Cyrtochilum trifurcatum* and *C. gargantua* are rather similar species, but they can be distinguished by glabrous vs papillate lip callus, respectively. The lip lateral lobes can be somewhat distinct in both species (rectangular-rhombic in the former vs obliquely triangular in the latter) as well as basal gynostemium appendages (linear, pending acute projections vs curved down, falcate). *Cyrtochilum trifurcatum* is easily distinguishable from *C. serratum* based on gynostemium appendages (two pairs in *C. trifurcatum*, one pair in *C. serratum*). These additional appendages separate *C. trifurcatum* from other species with strongly undulate petals, as *C. kienastianum*.

*Representative specimens*: COLOMBIA. **Cauca**: Popayán, 2,200–2,500 m, August–February, *F.C. Lehmann 6761* (AMES!, UGDA-DLSz!—drawing); Mpio. Inza. Vereda Belencito, 2°29′18.4″N 76°5′7.4″W, 2,485 m, 23 November 1998, *O. Rivera Diaz, P. Galvis & A. Ortega ORD945* (COL!, UGDA-DLSz!—drawing). **Huila**: Mpio. Palermo. Inspeccion del Carmen, vereda Pinares, 2,350–2,550 m, 1 August 1992, *F. Llanos & J. Camacho 2367* (COL!). **Putumayo**: Bosque alto al N del Valle de Sibundoy. Hacia la mina de San Francisco, 2,800 m, 9 January 1957, *M. Ospina H. 142a* (COL!, UGDA-DLSz!—drawing, photo); Bosque alto al norte del Valle de Sibundoy, hacia la mina de San Francisco, 2,800 m, 9 January 1957, *M. Ospina H. & J. Idrobo 142* (AMES!, UGDA-DLSz!—fragment, drawing, photo). **Risaralda**: Mpio. Pereira. Parque regional Ucumari. Entre El Cedral y La Pastora, 2,300 m, 15 June 1989, *R. Bernal & Estud. Sist. 1673* (COL!). ECUADOR. **Azuay**: Gualaceo-Limon, Plan de Milagro, 1,800 m, 7 October 1981, Collected and cultivated in Cuenca by Alfonso Pozo, *C. Dodson & P. Dodson 11655* (SEL—Dodson & Dodson, 1984). **Carchi**: Tulcan-La Bonita, 2,540 m, 17 May 1981, *F. Kuhn et al. 146* (SEL—Dodson & Dodson, 1984). **Loja**: Nudo de Sabanillo, along road Yangana-Valladolid, forest or scrub on sanstone (with *Sphagnum, Xyris, Pinguicula* and *Eriocaulaceae*), 2,500–3,000 m, 3 May 1987, *H. van der Werff & W. Palacios 9263* (AMES! *ex* MO, UGDA-DLSz!—drawing). Yangana-Valladolid, 2,250 m, 11 May 1981, *F. Kuhn et al. 90* (SEL—Dodson & Dodson, 1984); km 12 Loja-Zamora, 2,600 m, 21 September 1980, *C. Dodson et al. 10523* (SEL—Dodson & Dodson, 1984). **Napo**: Quijos Canton. Parroquia Baez. Comunidad de Santa Lucia de Bermejo. Bosque muy humedo Montano Bajo, 0°31′S 77°55′W, 2,200–2,400 m, 14 December 1993, *A. Alvarez & O. Brito 863* (MO!, UGDA-DLSz!—drawing); Tena, Parque Nacional Llanganates. Via Salcedo-Tena, km 72–74, riberas del Rio Mulatos. Bosque muy humedo Montano Bajo, 0°00′S 78°11′W, 2,040 m, 15 September 1998, *H. Vargas, E. Narvaez & S. Orellana 2405* (MO!); Cosanga, Baeza-Tena, 1,600 m, 26 June 1983, *C. Dodson et al. 14034* (SEL—Dodson & Dodson, 1984). **Tungurahua**: East-facing slopes of Volcan Tungurahua, 3,200 m, 23 October 1961, *C. Dodson & L. Thien 1084* (SEL—Dodson & Dodson, 1984); near Baños, 2,400 m, 13 October 1961, *C. Dodson & L. Thien 1005* (SEL, US—Dodson & Dodson, 1984). **Zamora-Chinchipe**: Km 17 Loja-Zamora, 2,400 m, 16 April 1973, *L. Holm-Nielsen et al. 3606* (AAU, SEL—Dodson & Dodson, 1984).

***Cyrtochilum gargantua*** (Rchb. f.) Kraenzl., Notizbl. Bot. Gart. Berlin-Dahlem 7: 91. 1917. ≡ *Oncidium gargantua* Rchb.f., Linnaea 41: 24. 1876. TYPE: Peru. *Pearce 835* (holotype, W-R! 1458, UGDA-DLSz!—drawing, photo).

= *Cyrtochilum davisii* (Rchb. f.) Dalström, Lindleyana 16(2): 61. 2001. ≡ *Oncidium davisii* Rchb. f., Linnaea 41: 24. 1877[1876]. TYPE: Peru. *Davis s.n*. (holotype, W-R! 48881, UGDA-DLSz!—drawing, photo).

**Figure 4:**
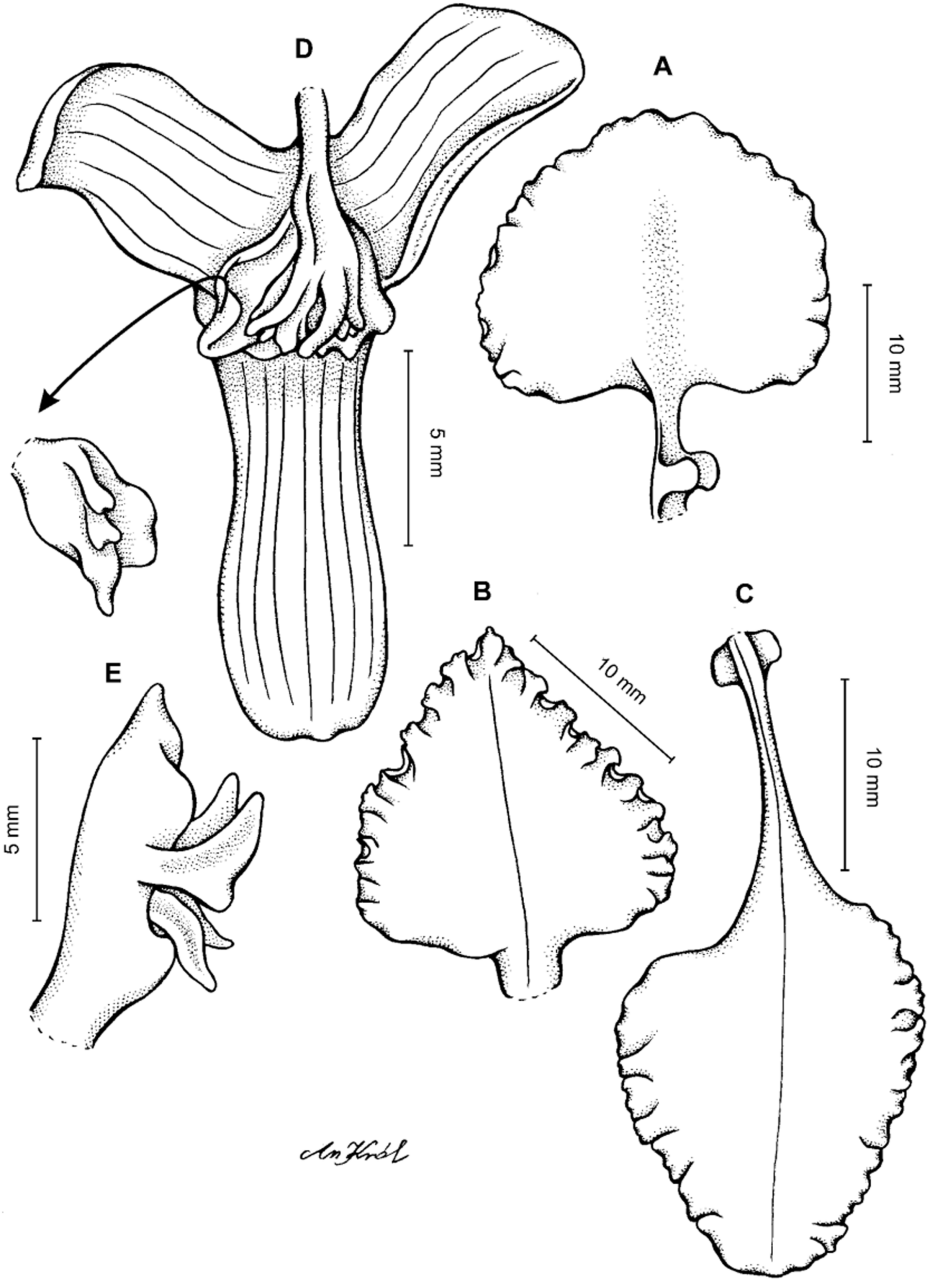
*Cyrtochilum gargantua* (Rchb. f.) Kraenzl. (A) Dorsal sepal; (B) Petal; (C) Lateral sepal; (D) Lip; (E) Gynostemium. Drawn by Anna Król from *Lehmann 6006* (AMES).

**Figure 5:**
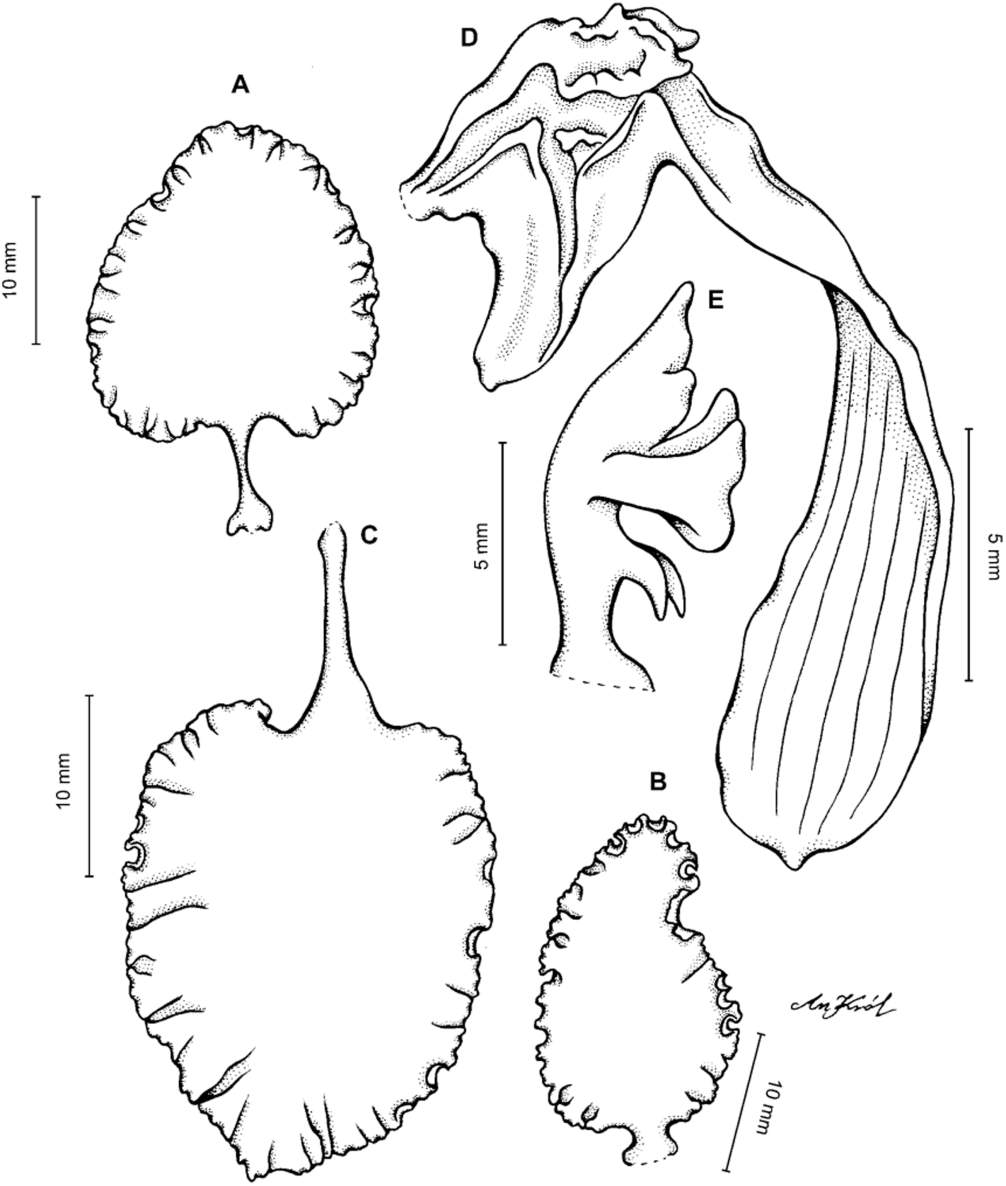
*Cyrtochilum gargantua* (Rchb. f.) Kraenzl. (A) Dorsal sepal; (B) Petal; (C) Lateral sepal; (D) Lip; (E) Gynostemium. Drawn by Anna Król from *Pearce 835* (W-R).

**Figure 6:**
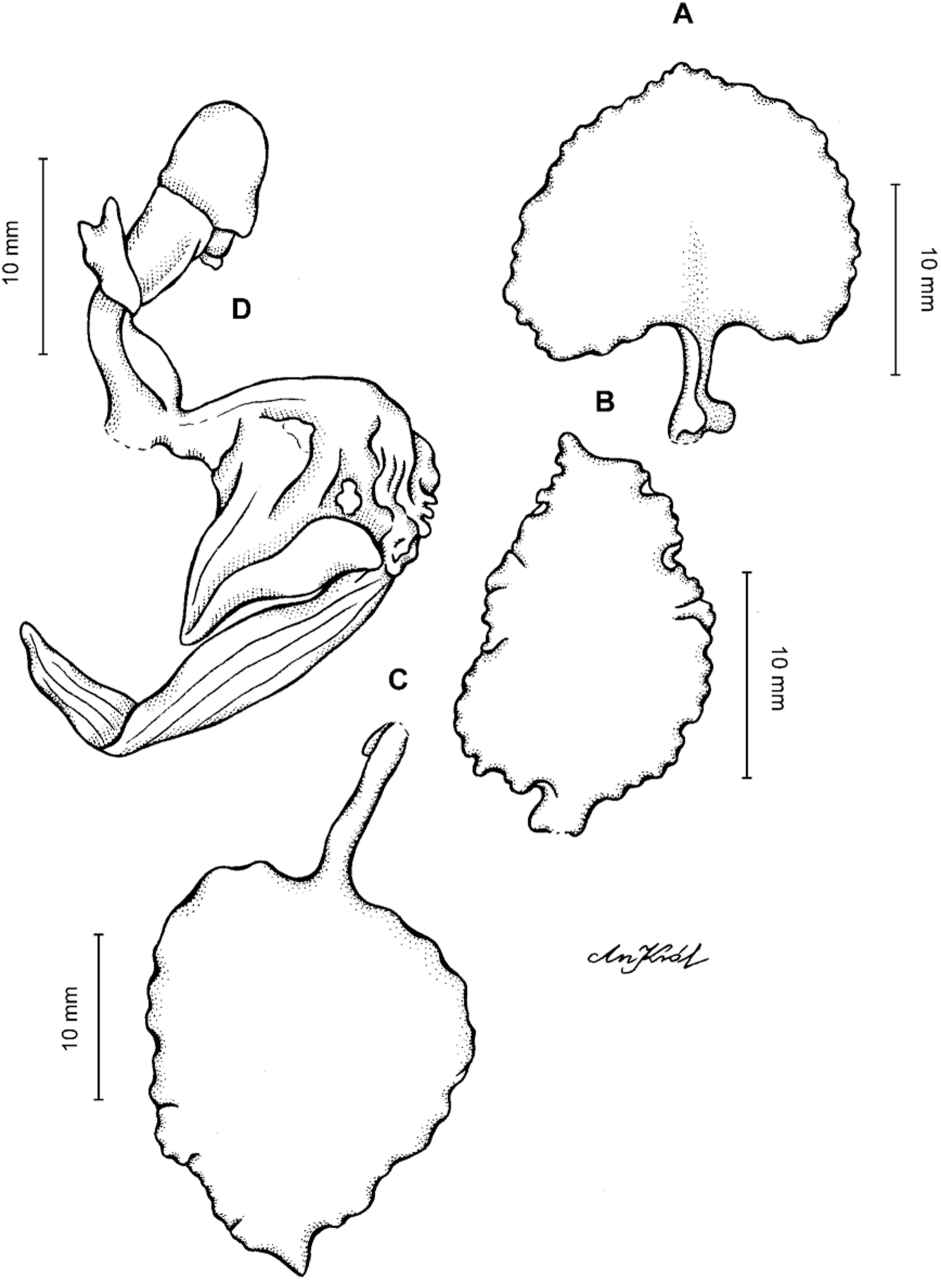
*Cyrtochilum gargantua* (Rchb. f.) Kraenzl. (A) Dorsal sepal; (B) Petal; (C) Lateral sepal; (D) Lip and gynostemium. Drawn by Anna Król from *Davis s.n*. (W-R).

Rhizome ascending. Pseudobulbs 11 cm long, 2 cm in diameter, narrowly ovoid, smooth, somewhat compressed, subtended basally by leafy sheaths, bifoliate. Leaves up to 60 cm long and 4.5 cm wide, oblanceolate, acute. Inflorescence up to 3 m long, paniculate, wiry, with well-spaced few-flowered branches. Flowers medium-sized, conspicuous, sepals brown to reddish-brown with yellow apex, petals brown to reddish-brown with yellow irregular stripes and apex, lip brown with yellow callus. Floral bracts 15–20 mm long. Pedicel and ovary 35–45 mm long. Dorsal sepal clawed; claw 8–9 mm long, narrow, canaliculate, with prominent rhombic wings just above the base; blade 19–28 mm long, 18–23 mm wide, broadly ovate to reniform, margins strongly undulate, base subcordate, apex rounded. Petals shortly clawed; claw about 3 mm long, wide; blade 18–24 mm long, 14–18 mm wide when spread, obliquely elliptic-ovate to almost triangular, base broadly cuneate to subtruncate, apex obtuse, margins crenate, strongly undulate. Lateral sepals prominently clawed; claw 14–15 mm long, narrow, more or less winged at base; blade 24–37 mm long, 21–24 mm wide, obliquely elliptic-ovate to broadly elliptic in general outline, cordate at the base, apex rounded, shortly acuminate, margins crenate, strongly undulate. Lip 18–23 mm long in total, hastate in general outline, 3-lobed just above the base, gently curved in natural position exposing large, partially papillate callus; basal part 8 mm long, 10–14 mm wide across, base subcordate, apex subacute; callus papillate, consisting of a prominent, median ridge dissected at the apex into a bunch of smaller ridges, flanked laterally by small outgrowths; apical part 10–13 mm long, 4 mm wide, ligulate, apex obtuse to rounded. Gynostemium 9–10 mm long, gently arcuate, connate basally with the lip, lateral appendages obtriangular, apically truncate with small projection on the lower margin, hence appearing obscurely bilobed, reaching the anther base, and with an additional pair of curved down, falcate, acute projections just below stigma (Figs. 4–6).

*Ecology*: Terrestrial.

*Distribution*: Colombia, Ecuador, Peru. Alt. 800–1,100 m.

*Notes*: There are four species in this group having papillate lip callus, i.e. *C. cryptocopis, C. hastatum, C. ionodon* and *C. gargantua*. From all aforementioned species *C. cryptocopis* can be easily differentiated by the lip form which is pandurate in general outline, with clawed apical part, gradually transforming above cuneate base into flabellate blade. Unlike in *C. hastatum*, gynostemium lateral appendages of *C. gargantua* and *C. ionodon* are more or less obtriangular in general outline with truncate apex. *Cyrtochilum gargantua* is distinguishable from *C. ionodon* based on gynostemium morphology. In the former species lateral appendages have small projection on the lower margin, hence they appear obscurely bilobed while in *C. ionodon* lateral appendages are oblong, widened and truncate at the apex. Moreover, in *C. ionodon* there are no additional projections just below stigma. *Cyrtochilum gargantua* appears to be similar to *C. trifurcatum* as well, but has papillate lip callus, somewhat different form of the lip lateral lobes and form of the column lower appendages.

*Representative specimens*: COLOMBIA. **Boyacá**: Cordillera Oriental, carretera al Llano, entre Santamaría y Piedracampana, 800–1,100 m, 20, 25 July 1964, *H. Garcia-Barriga 18074* (AMES!, UGDA-DLSz!—drawing, photo). **Cauca**: Popayán, 2,400–2,800 m, December, *F.C. Lehmann 6006* (AMES!, UGDA-DLSz!—drawing, photo). *Sine loc. Sine coll*. (W-R!, UGDA-DLSz!—drawing, photo). ECUADOR. Cuenca, January 1883, *F. Klaboch s.n*. (W!, UGDA-DLSz!—drawing, photo). PERU. *Sine loc., G. Pearce 835* (W-R!, UGDA-DLSz!—drawing, photo). *Sine loc., W. Davis s.n*. (W-R!, UGDA-DLSz!—drawing, photo).

***Cyrtochilum minax*** (Rchb. f.) Kraenzl., Notizbl. Bot. Gart. Berlin-Dahlem 7: 91. 1917. ≡ *Oncidium minax* Rchb. f., Linnaea 41: 22. 1877[1876]. TYPE: Peru. *Pearce 818* (holotype, W-R! 1455; isotype, W-R! 48962, UGDA-DLSz!—drawing, photo).

**Figure 7:**
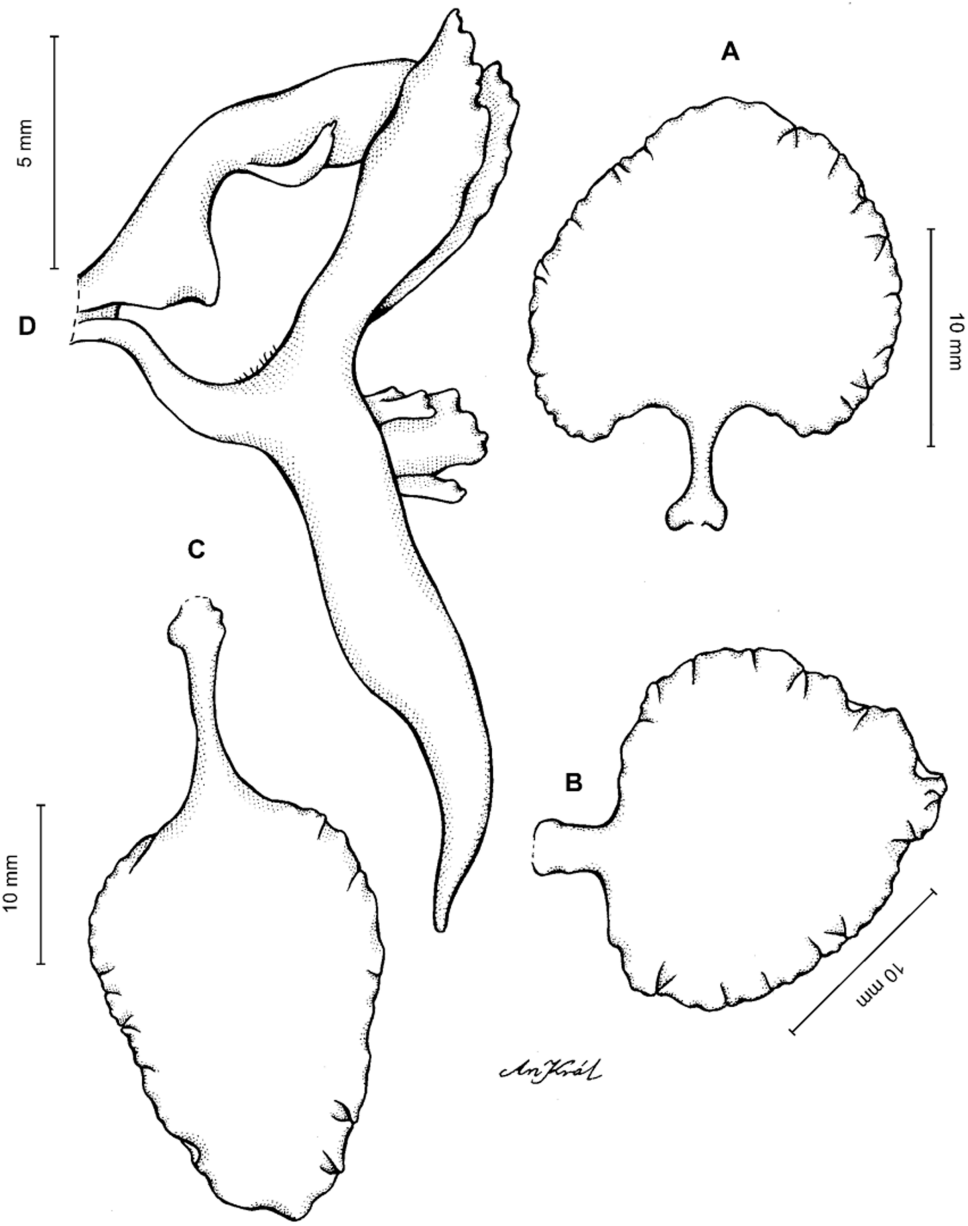
*Cyrtochilum minax* (Rchb. f.) Kraenzl. (A) Dorsal sepal; (B) Petal; (C) Lateral sepal; (D) Lip and gynostemium, side view. Drawn by Anna Król from *Pearce 818* (W-R).

Rhizome relatively short, ascending. Pseudobulbs up to 10 cm long and 3 cm wide, oblong ovoid, smooth, somewhat compressed, basally enclothed in leafy sheaths. Leaves 2–3, about 40–50 cm long, up to 5 cm wide, linear-oblanceolate, acute. Inflorescence about 1.5–2 m long, elongate and climbing, sparsely branching, branches (only 2 separate ones present in the type) erect, 13–16 cm long, apparently once-branched below, loosely several-flowered. Flowers rather large. Floral bracts 15 mm long. Pedicel and ovary 30 mm long. Dorsal sepal clawed; claw 5 mm long, narrow, canaliculate, with prominent obliquely ovate wings just above the base; blade 14 mm long, 16 mm wide, reniform, base cordate, margins entire, somewhat undulate, apex rounded. Petals clawed; claw about 3–4 mm long; blade 13–14 mm long, 13 mm wide when spread, somewhat oblique, suborbicular-ovate, base truncate, apex obtuse, margins irregularly undulate. Lateral sepals prominently clawed; claw 10 mm long, narrow, with basal obscure wings; blade 17 mm long, 14 mm wide, obliquely elliptic-ovate in general outline, basally broadly cuneate, obtuse at the apex, margins very slightly undulate. Lip 19 mm long in total, hastate in general outline, prominently 3-lobed just above the base, clawed; claw about 6 mm long, narrow; basal part 2–2.5 mm long, 18 mm wide across, oblong oblanceolate in outline, erect, apex irregularly dentate, base papillate; callus at the base of the middle lobe consisting of median rhombic ridge flanked by smaller, digitate outgrowths on both sides and in front; apical part 13 mm long, 2.6 mm wide, ligulate-linear, attenuate towards the apex, acute. Gynostemium 10 mm long, arcuate, connate basally with the lip, and with prominent chin-like protruding just above it, lateral appendages falcate, oblanceolate, not reaching the anther base (Fig. 7).

*Ecology*: Epiphyte growing in cloud forest.

*Distribution*: Ecuador (Jørgensen & Ulloa Ulloa, 1994), Peru (Schweinfurth, 1961; Vargas Calderón, 1965; Brako & Zarucchi, 1993).

*Notes*: *Cyrtochilum minax* is characterized by peculiar lip form, with oblong oblanceolate lateral lobes, which are erect, with apex irregularly dentate, and with papillate base. The prominent callus is perpendicular to the lip surface and much exposed in the lip centre. Somewhat similar in the general outline of the lip is Peruvian *C. pastorellii*. The latter species however, has non-undulate margins of sepals and petals, glabrous lip base, fringed projections along margins of lateral lobes and prominent triangular ridges just below stigma, instead of basal chin-like protruding found in *C. minax*. Unlike recently described C. *deburghgraeveanum* lip lateral lobes of *C. minax* are irregularly dentate at apex and petals are almost as long as wide.

*Representative specimen*: PERU. *Sine loc., G. Pearce 818* (W-R!, UGDA-DLSz!—drawing, photo).

***Cyrtochilum pastorellii*** (Dodson & D.E. Benn.) Senghas *in* F.R.R. Schlechter, Orchideen Beschreib. Kult. Zücht., ed. 3, I/C(33–36): 2205. 1997. ≡ *Oncidium pastorellii* Dodson & D.E. Benn., Icon. Pl. Trop., ser. 2, 2: t. 137. 1989. TYPE: Peru. *Bennett 3522* (holotype, MO).

Rhizome short. Pseudobulbs 5 cm long, 1 cm wide, narrowly ovoid-pyriform, basally enclothed in 2–3 pairs of imbricating, foliaceous sheaths, unifoliate. Leaf up to 30 cm long, 3 cm wide, elliptic-lanceolate, acute, conduplicate at the base. Inflorescence up to 150 cm long, paniculate, branch 2–4-flowered. Flowers showy, tepals light brown with yellow apices and margins, lip light brown with cream-white callus. Dorsal sepal clawed; claw about 4 mm long, narrow, winged; blade 20 mm long, 14 mm wide, ovate-triangular, acute, margins flat, non-undulate. Petals 24 mm long in total, 12 mm wide, shortly clawed, falcate, ovate-elliptic, acute, margins flat, non-undulate. Lateral sepals clawed; claw about 5 mm long, prominently winged; blade 23 mm long, 12 mm wide, ovate-lanceolate to oblong-ovate, acute, margins flat, non-undulate. Lip 22 mm long in total, 3-lobed just above the base, more or less hastate-triangular in general outline; basal part 4–5 mm long, 18 mm wide when spread, rhombic-ovate, acute, with fringed projections along margins; callus consisting of median ridge and flanked by prominent lateral ridges, deeply dissected by relatively short tuberculate outgrowths; apical part 14–15 mm long, about 5 mm wide, oblong to oblong-lanceolate, acute. Gynostemium 12 mm long, slightly sigmoid, wings linear, bidentate, upcurved, with prominent triangular ridges just below stigma.

*Ecology*: Terrestrial in wet cloud forest.

*Distribution*: Peru. Alt. 2,800 m.

*Notes*: Dalström (2001) considered this species to be conspecific with *C. cordatum*, but the lip callus of the latter is dissected only in apical, more elevated part (vs keels dissected from the base almost to the apex of the callus) and column appendages are falcate and acute (vs linear, bidentate). Additionally, lip lateral lobes of *C. pastorellii* are adorned with fringed projections, which are missing in *C. cordatum*. *Cyrtochilum pastorellii* differs from similar *C. lamelligerum*, *C. macranthum* and *C. xanthocinctum* in form of the dorsal sepal which is broadly ovate to transversely elliptic, basally more or less cordate in these three species (vs ovate-triangular, basally cuneate in *C. pastorellii*). In general, the flower architecture of *C. pastorelli* resembles that of *C. minax*, described above and of *C. deburghgraeveanum*, which we discuss below. Sepals and petals of both these species, however, are more or less undulate. Additionally, lip base of *C. minax* is papillate, lip lateral lobes have entire margins, and gynostemium has prominent basal, chin-like protruding. Lip callus of *C. pastorellii* is not as high and exposed as in *C. minax*.

*Representative specimen*: PERU. **Huánuco**: Carpish Pass along road to Tingo Maria, 2,800 m, 6 November 1986, *D. Bennett 3522* (MO).

***Cyrtochilum deburghgraeveanum*** Dalström & S. Ruíz, Lankesteriana 12(2): 93. 2012. TYPE: Peru. *Dalström 3498* (holotype, USM).

**Figure 8:**
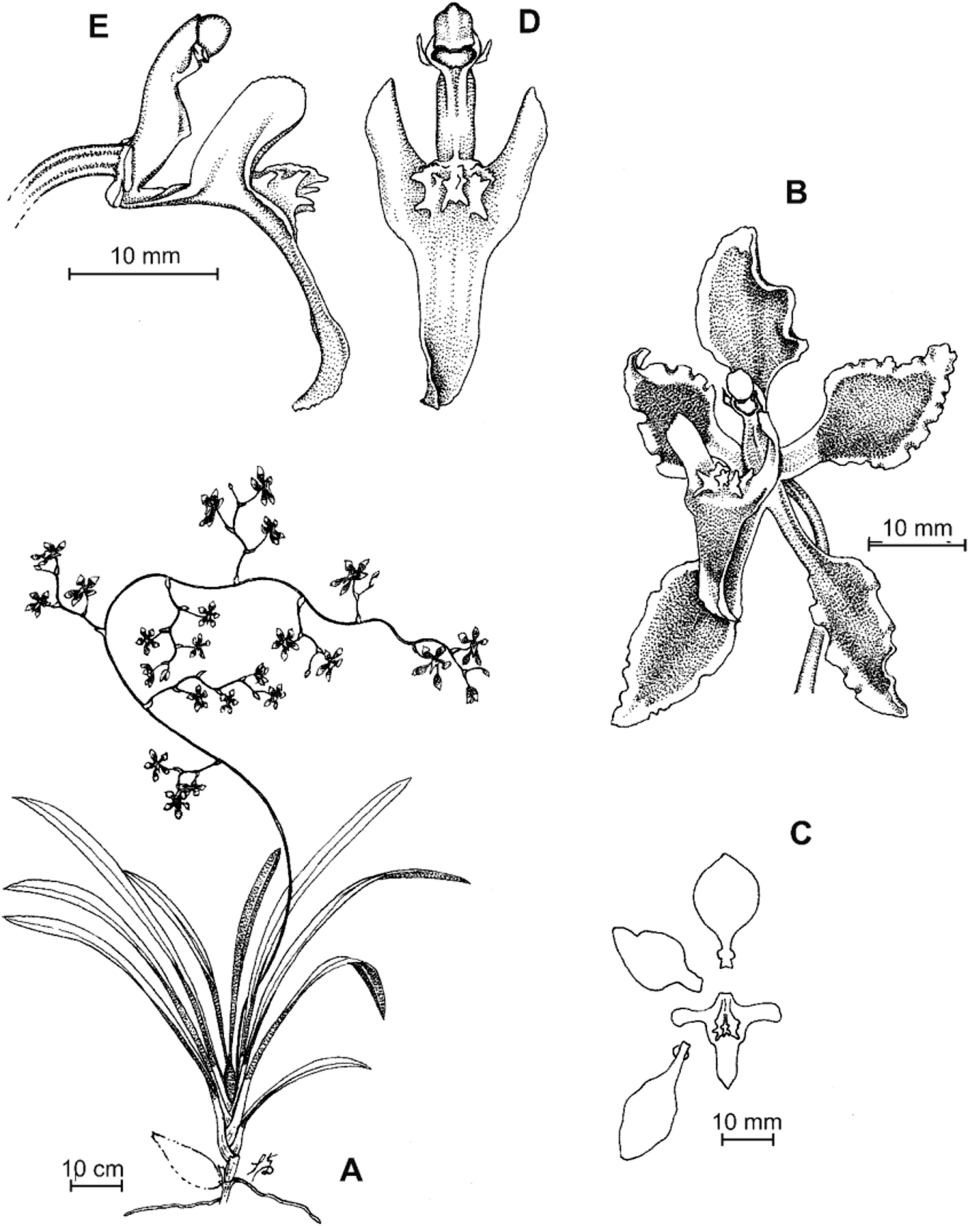
*Cyrtochilum deburghgraeveanum* Dalström & S. Ruíz. (A) Habit; (B) Flower; (C) Dissected perianth; (D) Gynostemium and lip, front view; (E) Gynostemium and lip, side view. Drawn by Stig Dalström from the holotype, *Dalström 3498* (USM).

Pseudobulbs about 10 cm long, 5 cm wide, caespitose or slightly creeping on a bracteate rhizome, oblong ovoid, distantly bifoliate (terminal leaf about 2 cm above lower leaf), surrounded basally by 7–8 distichous sheaths, the uppermost foliaceous. Leaves subpetiolate, 30–45 cm long, 1.5–2.5 cm wide, conduplicate, narrowly elliptic to oblanceolate, narrowly acute to broadly acuminate. Inflorescence up to about 160 cm long, paniculate, axillary from the uppermost sheaths, erect, then wiry and flexuous, with widely spaced 3–5 flowered lateral branches. Flower stellate to slightly campanulate, showy, tepals brown with white to yellow edges and apex, lip white with pale brown front lobe and white callus. Floral bracts 10–15 mm long, involute and cucullate. Pedicel with ovary 20–35 mm long. Dorsal sepal 13–24 mm long, 11–12 mm wide, spathulate with basal auricles, then laminate, ovate, obtuse to acute, undulate. Petals 23–24 mm long, 10–13 mm wide, broadly spathulate, ovate, obtuse to acute, oblique, undulate. Lateral sepals 25–30 mm long, 9–10 mm wide, spathulate with basal auricles, laminate, elongate ovate, obtuse to obliquely acute. Lip 22 mm long in total, rigidly attached to the base of the gynostemium, hastate in general outline, cuneate at base, 3-lobed above; basal part about 4–5 mm long, 22 mm wide across, lateral lobes erect, broadly linear and elongate, obliquely rounded at apex; callus with three low and flattened, fleshy, longitudinal keels emerging from the base and extending to the front-lobe, then spreading, with the lateral pair ending in erect, laterally flattened angulate keels, with an additional emerging pair of erect, angular, laterally flattened, denticulate keels that are placed on each side of the rectangularly angulate central keel; apical part about 12–14 mm long, 5 mm wide, narrowly triangular-ligulate, apically slightly convolute, obtuse to acute, recurved. Gynostemium about 11 mm long, slightly sigmoid, with two parallel longitudinal keels below the stigma, and a pair of lateral, erect and falcate, slightly unequally bilobed to digitate and pointed wings (Fig. 8).

*Ecology*: Epiphyte in heavily deforested montane area.

*Distribution*: Peru. Alt. about 2,200 m.

*Notes*: This species resembles *C. cordatum* but differs in the rounded and white lateral lobes of the lip versus pointed and brown to purple ones, bordered with white for *C. cordatum*. The callus of *C. cordatum* is also more elaborate with an additional lateral tooth next to the main central structure (Dalström, Deburghgraeve & Ruíz Perez, 2012). *Cyrtochilum deburghgraeveanum* is distinguishable from *C. ruizii* and *C. hastiferum* by the lip form whose lateral lobes are obliquely obovate, slightly serrate or undulate on margin in the latter two species. There are additional two species with which it can be alternatively confused, i.e. *C. minax* and *C. pastorellii*. *Cyrtochilum deburghgraeveanum* is somewhat similar to *C. minax* by having more or less undulate sepals and petals, but differs from the latter i.a. by having glabrous lip base (vs papillate in *C. minax*), obliquely rounded apex of lip lateral lobes (vs irregularly dentate in in *C. minax*) and relatively longer and narrower petals. Flower segments of *C. deburghgraeveanum* reminds those of *C. pastorellii*, in which however, their margins are non-undulate, but lip lateral lobes are more or less fringed.

*Representative specimen*: PERU. **Amazonas**: Jumbilla, Florida, Gualulo, about 2,200 m. collected by S. Ruíz and G. Deburghgraeve November 2010, *S. Dalström 3498* (USM).

***Cyrtochilum cordatum*** (Lindl.) Kraenzl., Notizbl. Bot. Gart. Berlin-Dahlem 7: 92. 1917. ≡ *Oncidium cordatum* Lindl., Sert. Orchid.: t. 25. 1838. TYPE: Peru. *Mathews 1067* (holotype, K-L; isotype, BM, W-R! 48961, UGDA-DLSz!—drawing, photo).

**Figure 9:**
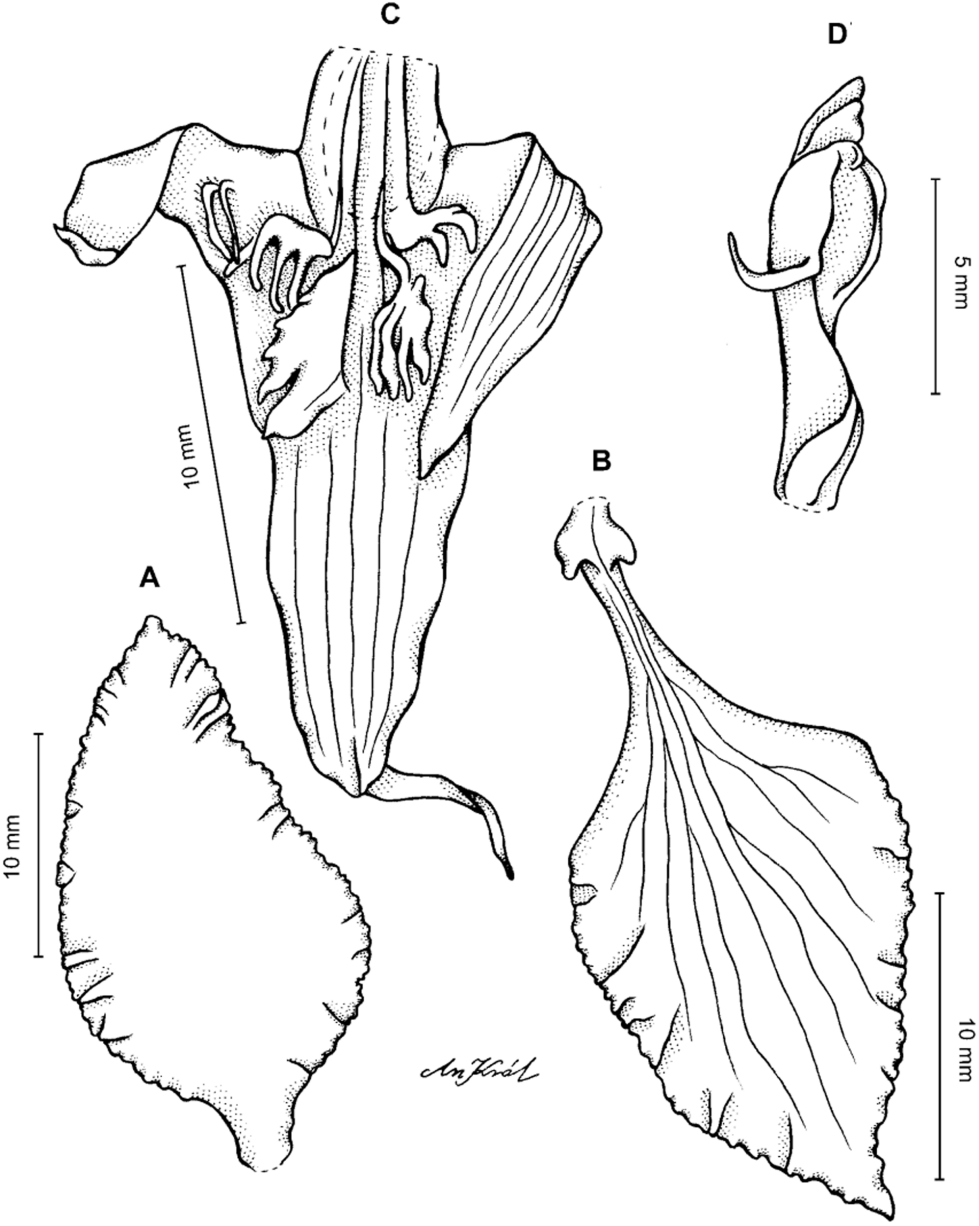
*Cyrtochilum cordatum* (Lindl.) Kraenzl. (A) Petal; (B) Lateral sepal; (C) Lip; (D) Gynostemium, side view. Drawn by Anna Król from *Mathews s.n*. (W-R).

Pseudobulbs about 10 cm long, 5 cm wide, caespitose or slightly creeping on a bracteate rhizome, oblong ovoid, distantly bifoliate (terminal leaf about 2 cm above lower leaf), surrounded basally by 7–8 distichous sheaths, the uppermost foliaceous. Leaves subpetiolate, 30–45 cm long, 1.5–2.5 cm wide, conduplicate, narrowly elliptic to oblanceolate, narrowly acute to broadly acuminate. Inflorescence up to about 160 cm long, paniculate, axillary from the uppermost sheaths, erect, then wiry and flexuous, with widely spaced 3–5 flowered lateral branches. Flower stellate to slightly campanulate, showy, tepals brown with white to yellow edges and apex, lip white. Floral bracts 10–15 mm long, involute and cucullate. Pedicel with ovary 20–35 mm long. Dorsal sepal 13–24 mm long, 11–12 mm wide, spathulate with basal auricles, then laminate, ovate, obtuse to acute, undulate. Petals 25 mm long, 13 mm wide, ovate to elliptic-ovate, subobtuse to subacute, oblique, margins undulate. Lateral sepals clawed; claw about 9 mm long, narrow, with prominent basal auricles; blade about 20 mm long, 12 mm wide, deltoid-ovate, acute, margins crenate and undulate. Lip 25 mm long in total, rigidly attached to the base of the gynostemium, hastate in general outline, truncate at base just above short claw, 3-lobed; basal part about 2–3 mm long, 22 mm wide across, spread, lateral lobes linear-lanceolate, acute; callus consisting of 3 prominent, longitudinal keels extending apically and here deeply dissected, flanked by filiform outgrowths; apical part about 20 mm long, 6 mm wide, narrowly triangular-lanceolate, apically slightly convolute, acuminate, recurved. Gynostemium about 10 mm long, slightly erect-sigmoid, with two, obscure, parallel longitudinal keels below the stigma, and a pair of falcate, acute and upcurved wings (Fig. 9).

*Ecology*: Terrestrial or lithophytic plant growing in wet montane forest and on rocks near banks of river.

*Distribution*: Peru. Alt. 2,300–2,400 m.

*Notes*: *Cyrtochilum cordatum* appears to be similar to *C. xanthocinctum* from which it differs by the form of callus. Lip callus of the latter species is more laterally spreading and not so deeply dissected. *Cyrtochilum cordatum* differs from *C. pastorellii* in gynostemium appendages form (falcate, acute and upcurved in *C. cordatum* vs linear, bidentate, upcurved in *C. pastorellii*) and more dissected lip callus. Unlike *C. minax* lip base of *C. cordatum* is glabrous and petals prominently longer than wide.

*Representative specimens*: PERU. **Junín**: Prov. of Tarma, mountains of Yanangu, east of Huacapistana, 2,300–2,400 m, *A. Weberbauer 2119* (Schweinfurth, 1961). Near Pangoa, on rocks near banks of river, *A. Mathews 1067* (BM, K-L, W-R!).

***Cyrtochilum xanthocinctum*** Dalström & S. Ruíz, Lankesteriana 12(2): 98. 2012. TYPE: Peru. *S. Dalström 3450* (holotype, USM).

**Figure 10:**
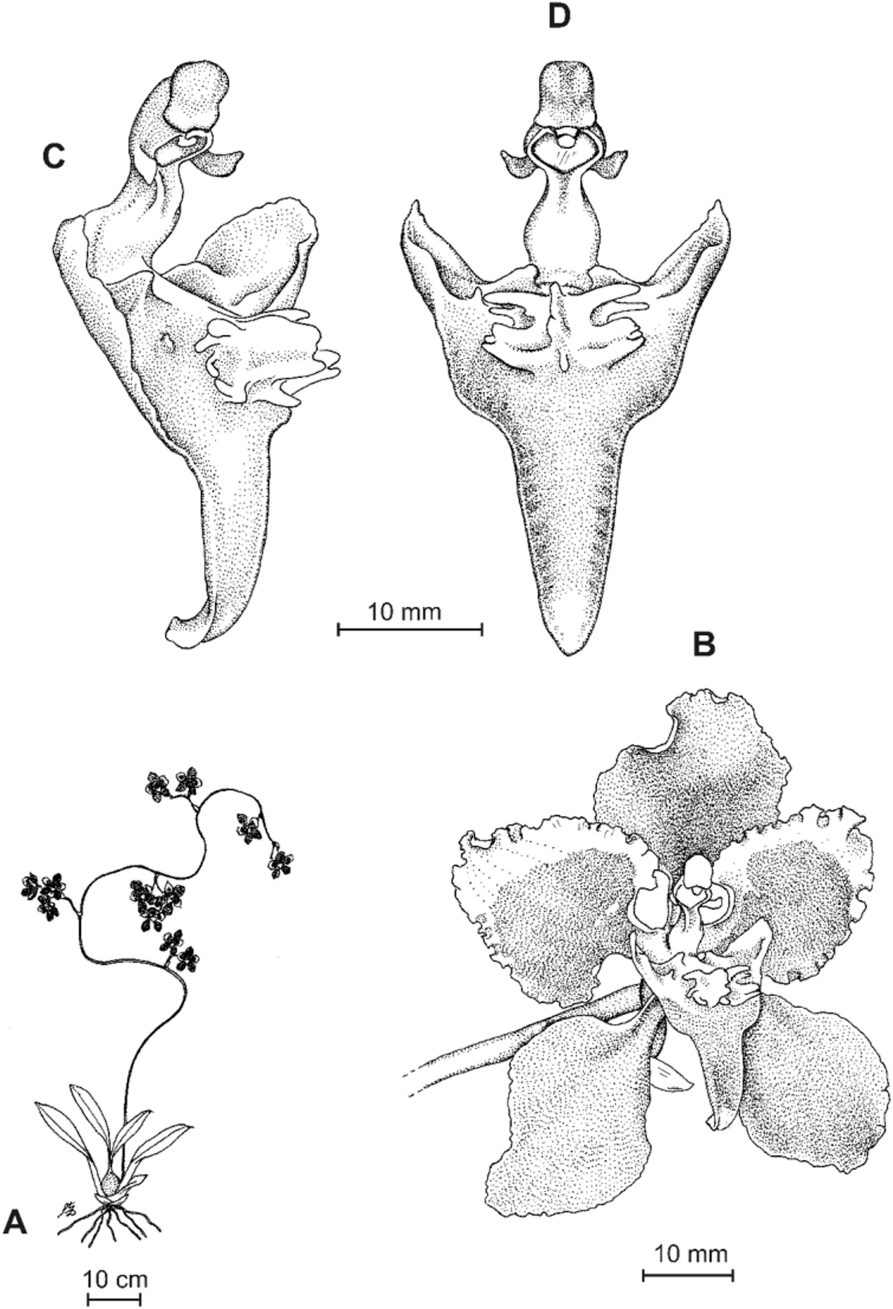
*Cyrtochilum xanthocinctum* Dalström & S. Ruíz. (A) Habit; (B) Flower; (C) Gynostemium and lip, side view; (D) Gynostemium and lip, front view. Drawn by Stig Dalström from the holotype, *Dalström 3450* (USM).

Pseudobulbs caespitose, about 7.5 cm long, 4.5 cm wide, broadly ovoid, bifoliate, surrounded basally by 4–6 distichous sheaths, the uppermost foliaceous. Leaves 13–17 cm long, about 3.5 cm wide, subpetiolate, conduplicate, oblong-oblanceolate, acute. Inflorescence up to about 150 cm long, axillary, from the uppermost sheaths, erect then wiry. Flower stellate and showy, sepals brown, petals basally white then with a large brown blotch, bordered with yellow, lip basally pale lilac, then white, and then brown with a lighter apex and whitish callus. Floral bracts about 10–15 mm long, involute cucullate. Pedicel with ovary about 50 mm long. Dorsal sepal about 28 mm long, 23 mm wide, spathulate with basal auricles, then cordate laminate, broadly ovate, obtuse and apiculate, widely undulate. Petals 27 mm long, 22 mm wide, broadly and shortly spathulate, then cordate laminate, ovate, rounded obtuse, strongly undulate. Lateral sepals 35 mm long, 20 mm wide, spathulate with basal auricles, then cordate laminate, ovate, rounded obtuse, widely undulate. Lip 23 mm long, 20 mm wide, rigidly attached to the base of the column, hastate-triangular in general outline, 3-lobed, with a pair of basal, erect and slightly incurved denticles, and widely spreading semi-erect and slightly concave, angular lateral lobes, with or without additional erect minor denticles, and a narrowly triangular, ligulate, slightly recurved, acute middle lobe, callus of a fleshy central, longitudinal, low keel turning into an erect structure near the base of the front lobe, similar to some strange aircraft with spreading double pairs of wings. Gynostemium 12 mm long, slightly sigmoid, with a pair of parallel longitudinal ventral keels below the stigma, and with lateral, oblique obdeltoid, wine-red spreading wings (Fig. 10).

*Ecology*: Epiphyte in scrubby patch of previously burnt vegetation.

*Distribution*: Peru. Alt. 2,400 m.

*Notes*: Although morphologically similar to the sympatric *C. macranthum*, *C. xanthoxinctum* can be easily recognized by the brown petals with strikingly contrasting yellow borders. Also the lateral callus keels on the lip are more laterally spreading (Dalström, Deburghgraeve & Ruíz Perez, 2012). Other species similar to *C. xanthocinctum* are *C. minax*, *C. pastorellii, C. cordatum* and *C. deburghgraeveanum*. From all of these species, sepals and petals of *C. pastorellii* are non-undulate, and margins of lip lateral lobes are ornamented with finger-like projections. The lip base of *C. minax* is papillate, what is unknown in *C. xanthocinctum*. The lip callus of *C. cordatum* is deeply dissected for numerous segments and more complicated than in *C. xanthocinctum*. Flowers segments of *C. deburghgraeveanum* are narrower than those of *C. xanthocinctum*, and lip callus is distinct with more prominent median keel.

*Representative specimen*: PERU. **Amazonas**: Chachapoyas, Molinopampa. Alt. about 2,400 m. Collected by S. Ruíz in March 2011. *S. Dalström 3450* (USM).

***Cyrtochilum macranthum*** (Lindl.) Kraenzl., Notizbl. Bot. Gart. Berlin-Dahlem 7: 95. 1917. ≡ *Oncidium macranthum* Lindl., Gen. Sp. Orchid. Pl.: 205. 1833. TYPE: Peru. *Ruiz & Pavón s.n*. (holotype, K; isotype, BM).

**Figure 11:**
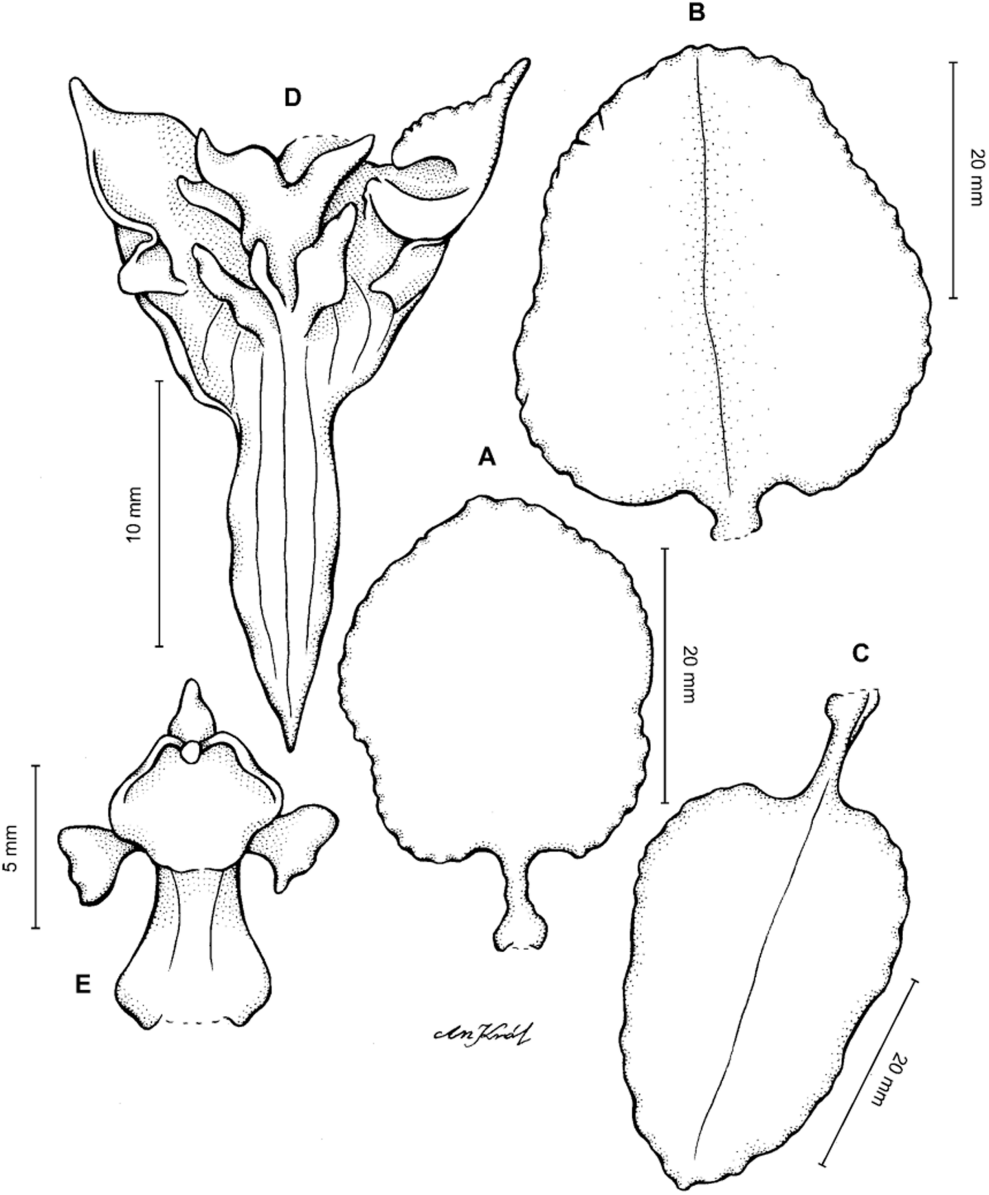
*Cyrtochilum macranthum* (Lindl.) Kraenzl. (A) Dorsal sepal; (B) Petal; (C) Lateral sepal; (D) Lip; (E) Gynostemium, front view. Drawn by Anna Król from *Pearce 461* (W-R).

**Figure 12:**
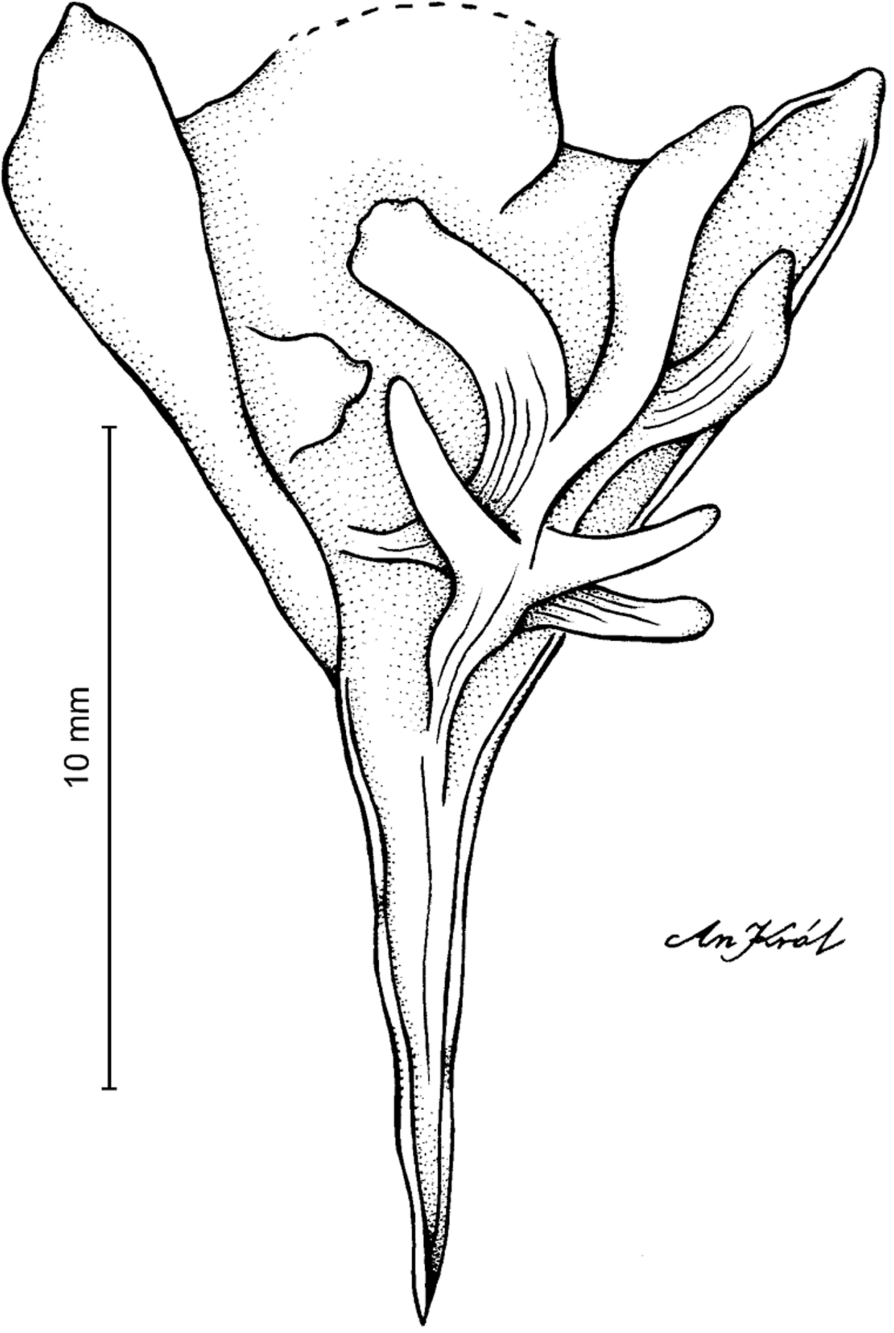
*Cyrtochilum macranthum* (Lindl.) Kraenzl. Lip. Drawn by Anna Król from *Warszewicz s.n*. (AMES).

Rhizome creeping. Pseudobulbs more or less distant or subcaespitose, up to 10 cm long and 4 cm wide, ovoid, somewhat compressed, subtended basally by leafy sheaths. Leaves 1–2, up to 40 cm long and 5 cm wide, oblanceolate, acute. Inflorescence up to 300 cm long, wiry, paniculate, with well-spaced, few-flowered branches. Flowers large, showy, sepals yellow to deep bronze, petals bright yellow with brown suffusion, lip lilac to purple with yellow middle lobe, callus white and purple. Floral bracts 25–30 mm long. Pedicel and ovary up to 65 mm long. Dorsal sepal clawed; claw about 5–10 mm long, narrow, canaliculate, with elliptic-rhombic wings just above the base; blade 27–35 mm long, 24–30 mm wide, suborbicular, base subcordate, margins somewhat irregularly undulate, apex rounded. Petals clawed; claw about 3–5 mm long, basally somewhat winged; blade 38–42 mm long, 34–35 mm wide when spread, somewhat oblique, broadly elliptic-ovate, obtuse or truncate at the apex, base subcordate, margins somewhat undulate. Lateral sepals clawed; claw 8–15 mm long, narrow, with basal wing on the outer margin; blade up to 35 mm long, 22–26 mm wide, obliquely elliptic-ovate in general outline, basally rounded, subobtuse at the apex, margins very slightly undulate. Lip 18–30 mm long in total, 12–23 mm wide, triangular in outline, base truncate-subcordate, convex in the centre, callus prominent, complicated, in form of elevated keel apically 3-lobed and flanked basally by 3-lobed process; apical part linear-triangular to ligulate-lanceolate, acute. Gynostemium 9–11 mm long, subarcuate, connate basally with the lip, lateral appendages obliquely obtriangular, not reaching the anther base (Figs. 11 and 12).

*Ecology*: Epiphytic or terrestrial, growing in understory of cloud forest.

*Distribution*: Colombia (Schweinfurth, 1961), Ecuador, Peru. Alt. 2,200–2,500 m.

*Notes*: *Cyrtochilum macranthum* is an easily recognisable species by having large, yellow or yellow with brown suffusion flowers, hastate lip with very prominent lip callus in form of elevated keel apically 3-lobed and flanked basally by 3-lobed process. This species can be compared to *C. xanthocinctum* but has different lip callus with spreading lateral keels.

*Representative specimens*: ECUADOR. **Tungurahua**: Trail along W slope of Rio Ulba Canyon above Hacienda San Antonio (4 km up Rio Ulba from village of Ulba), 1°25′S 78°22′W, 2,200–2,500 m, 3 June 1985, *B. Stein 2957* (MO!, UGDA-DLSz!—drawing); *Sine loc. G. Pearce 461* (W-R! 1957, UGDA-DLSz!—drawing). [**Pichincha**]: Andes Quitensis, 1,857–1,859, *R. Spruce 6071* (W-R! 48830, UGDA-DLSz!—drawing). PERU. **Amazonas**: East of Chachapoyas between Tambos Bagazon and Almirante, 2,400 m, *A. Weberbauer 4441* (Schweinfurth, 1961); Sources of the Maranon, *J. Warszewicz 131* (Schweinfurth, 1961); Chuquiribamba, *Andre 4498* (Schweinfurth, 1961). *Sine loc., J. Warszewicz s.n*. (AMES!, UGDA-DLSz!—drawing).

***Cyrtochilum hastatum*** (Ruiz & Pavon) Mansf. *ex* Dalstrom, Lindleyana **16**(2): 66. 2001. ≡ *Cyrtochilum pavonii* (Rchb. f.) Kraenzl., Notizbl. Bot. Gart. Berlin-Dahlem 7: 92. 1917. ≡ *Oncidium pavonii* Rchb.f. & Warsz. *ex* Lindl., Fol. Orchid. 6: 5. 1855. TYPE: Peru. *Ruiz & Pavón s.n*. (holotype, W-R! 48944; isotype, G [photo, F-025492], UGDA-DLSz!—drawing), replacement name for *Maxillaria hastata* Ruiz & Pavon, Syst. Veg. Fl. Peruv. Chil. **1**: 222. 1798.

**Figure 13:**
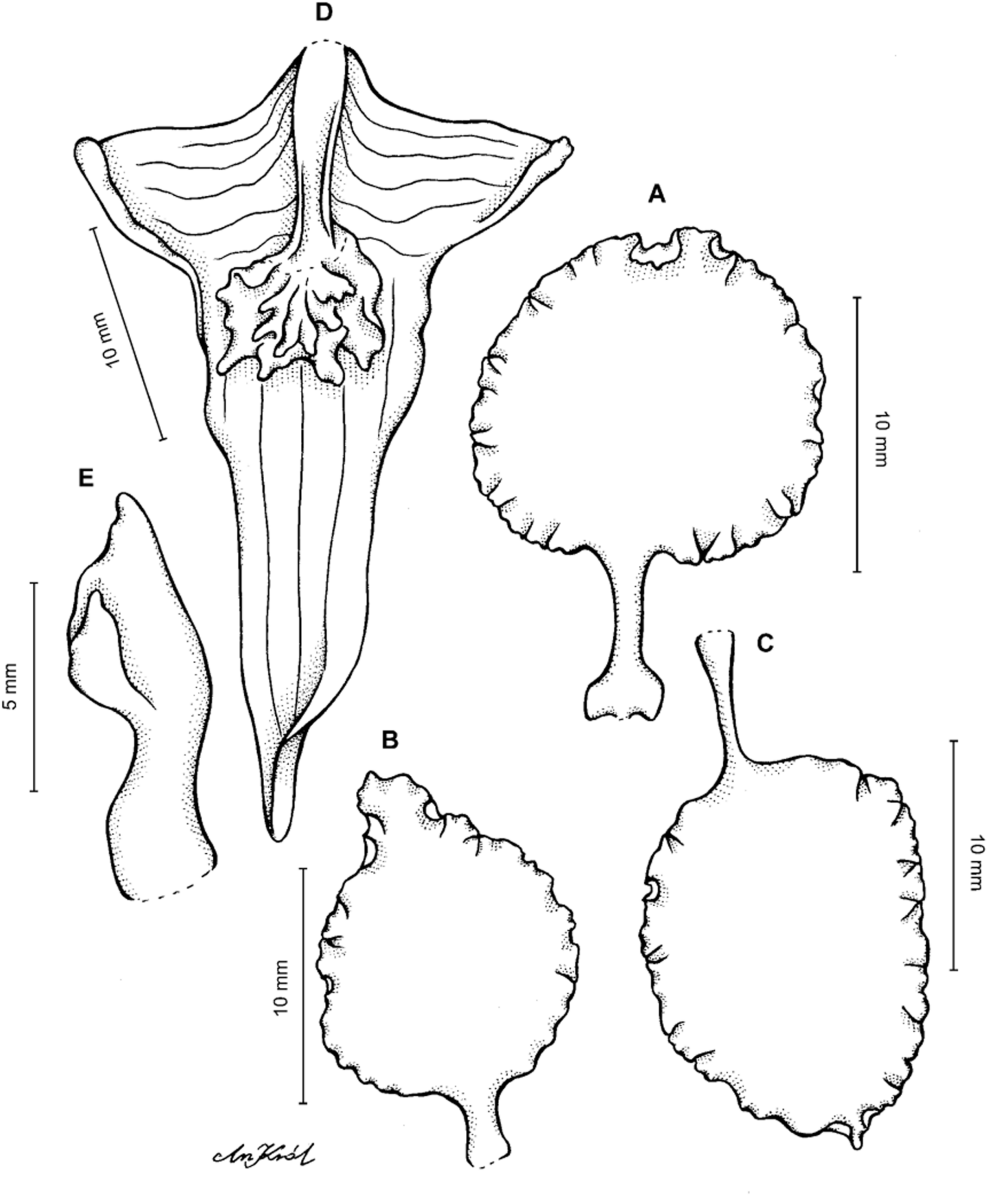
*Cyrtochilum hastatum* (Ruiz & Pavon) Mansf. *ex* Dalström. (A) Dorsal sepal; (B) Petal; (C) Lateral sepal; (D) Lip; (E) Gynostemium, side view. Drawn by Anna Król from *Pavón s.n*. (W-R).

**Figure 14:**
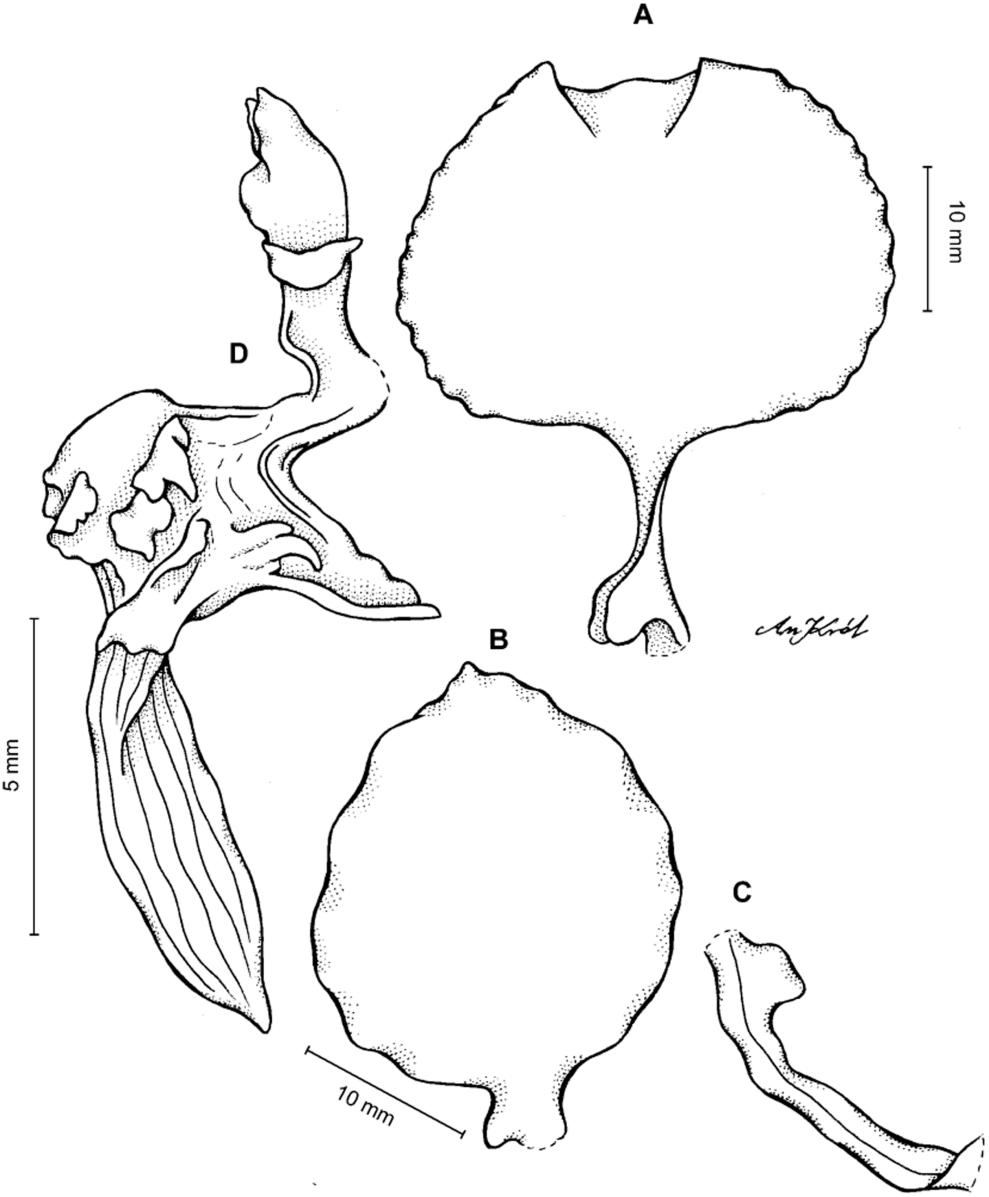
*Cyrtochilum hastatum* (Ruiz & Pavon) Mansf. *ex* Dalström. (A) Dorsal sepal; (B) Petal fragment; (C) Lateral sepal; (D) Lip and gynostemium, side view. Drawn by Anna Król from *Pavón 1067* (W-R).

Rhizome creeping. Pseudobulbs more or less distant or subcaespitose, up to 10 cm long and 4 cm wide, ovoid, somewhat compressed, subtended basally by leafy sheaths. Leaves 1–2, up to 40 cm long and 5 cm wide, oblanceolate, acute. Inflorescence up to 300 cm long, wiry, paniculate, with well-spaced, few-flowered branches. Flowers large, showy, sepals yellow to deep bronze, petals bright yellow with brown suffusion, lip lilac to purple with yellow middle lobe, callus white and purple. Floral bracts 10–15 mm long. Pedicel and ovary 25–33 mm long. Dorsal sepal clawed; claw 6–8 mm long, narrow, canaliculate, with rhombic wings just above the base; blade 12–16 mm long, 12–17.5 mm wide, suborbicular to transversely elliptic, base subcordate or truncate, margins more or less irregularly undulate, apex rounded. Petals clawed; claw about 3–4 mm long, basally somewhat winged; blade 14–20 mm long, 11–17 mm wide when spread, somewhat oblique, broadly elliptic-ovate to triangular-ovate, subacute at the apex, base broadly cuneate, margins somewhat undulate. Lateral sepals clawed; claw 8–10 mm long, narrow, obscurely winged; blade up to 20 mm long and 17 mm wide, obliquely elliptic-ovate in general outline, basally rounded, subacute at the apex, margins slightly undulate. Lip 18–20 mm long in total, narrowly-triangular in outline, base truncate above short claw, convex in the centre, callus very prominent, papillate, complicated, in form of 3 keels, elevated and more or less dissected at the apex, flanked by additional projections; apical part about 11–13 mm long, up to 6 mm wide, linear-triangular, acute. Gynostemium 9–10 mm long, suberect, connate basally with the lip, lateral appendages obliquely oblanceolate, subacute, not reaching the anther base (Figs. 13 and 14).

*Ecology*: No data.

*Distribution*: Peru.

*Notes*: *Cyrtochilum hastatum* and *C. ionodon* are morphologically quite similar. Both have very similar lip form with very prominent, papillate callus, but can be separated by the form of gynostemium appendages (obliquely oblanceolate, subacute in *C. hastatum* vs oblong, widened and truncate at the apex in *C. ionodon*). Additionally, lip callus of *C. ionodon* is more protruding than in *C. hastatum*. Taxonomic status of both species requires further study, including genetic analyses.

*Representative specimens*: PERU. *Sine loc., H. Ruiz & J. Pavón s.n*. (W-R!, G—photo, UGDA-DLSz!—drawing). *Sine loc., J. Pavón 1067* (W-R!, UGDA-DLSz!—drawing).

***Cyrtochilum ionodon*** (Rchb.f.) Dalström, Lindleyana 17: 92. 2002. ≡ *Oncidium ionodon* Rchb.f., Linnaea 41: 23 (1876). TYPE: Peru. *Laurentius ex Linden s.n*. (holotype, W-R! 48924, UGDA-DLSz!—drawing).

**Figure 15:**
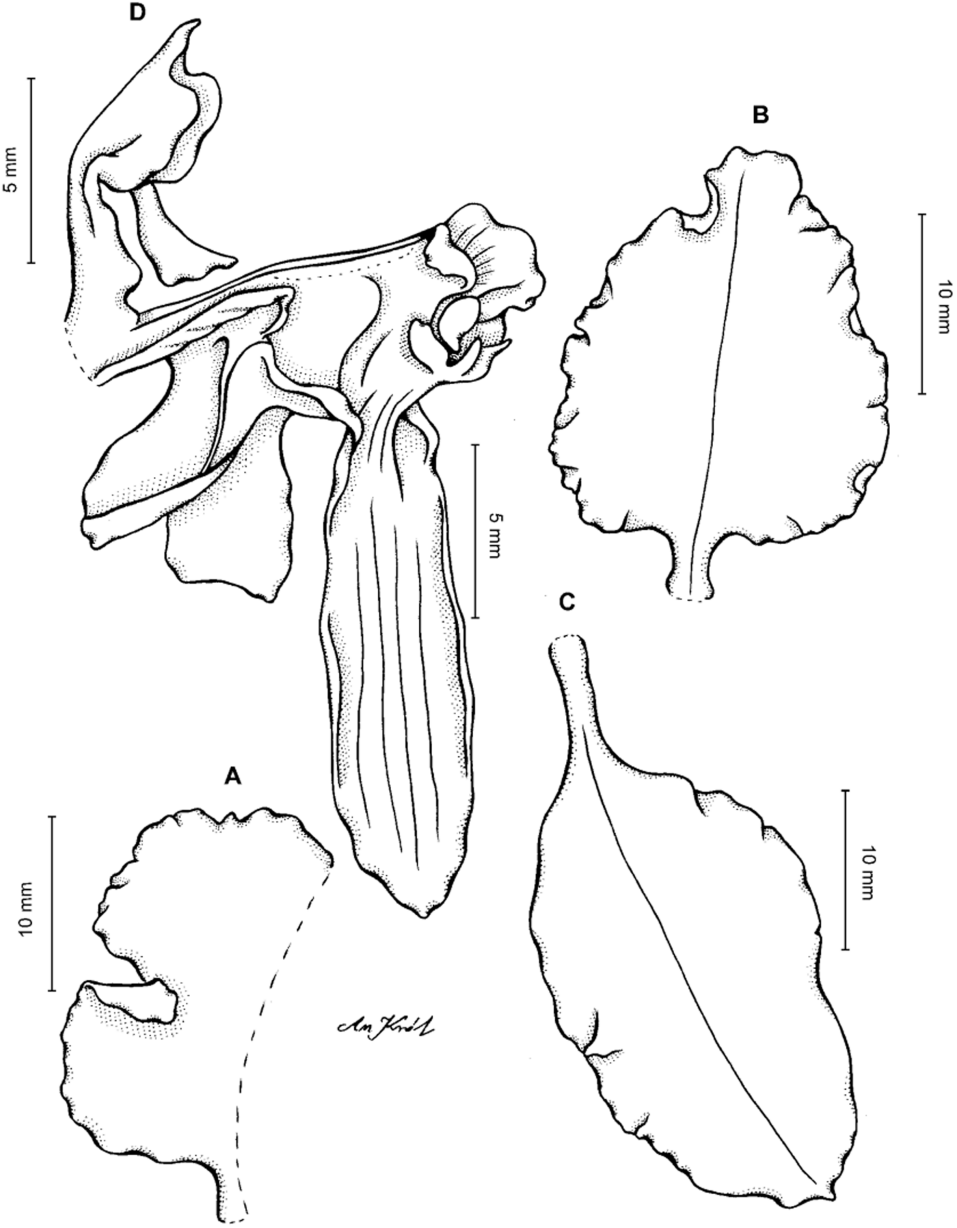
*Cyrtochilum ionodon* (Rchb.f.) Dalström. (A) Dorsal sepal; (B) Petal; (C) Lateral sepal; (D) Lip and gynostemium. Drawn by Anna Król from *Laurentius ex Linden s.n*. (W-R).

Plant caespitose. Pseudobulbs 9 cm long, 3 cm wide, slightly compressed, subtended by several foliaceous sheaths forming a massive fan-shaped growth. Leaves 55–60 cm long, 4–5.5 cm wide, erect-arching, linear, acuminate. Inflorescence up to 230 cm long, axillary, an erect then more or less wiry panicle with widely spaced branches. Flower stellate, sepals brown with yellow margin, petals yellow with many brown, irregular spots in basal 2/3 of lamina length, lip reddish brown with a yellowish centre. Dorsal sepal shortly clawed; lamina 25 mm long, 19 mm wide, ovate-orbicular, obtuse to truncate, margin more or less undulate. Petals shortly clawed; lamina 24 mm long, about 19 mm wide, broadly ovate, somewhat oblique, apex more or less bifid with a small apicule, margin undulate. Lateral sepals clawed; claw up to 10 mm long, narrow, basally auriculate; lamina 30 mm long, 14–19 mm wide, oblong ovate, subfalcate, apiculate, margin slightly undulate. Lip shortly clawed, hastate in general outline, basally cordate, 3-lobed just above claw, basal part papillate; basal part 3 mm long, 14 mm wide across, subrectangular, apical margins oblique; callus consisting of a central, longitudinal, elevated, fleshy, faintly 3-partite keel abruptly turning into an erect, irregularly dentate 3-lamellate structure with the central keel the highest and lateral ones spreading; apical part 14 mm long, 4 mm wide, oblong ligulate, subobtuse. Gynostemium 8.5 mm long, lateral appendages oblong, widened and truncate at the apex (Fig. 15).

*Ecology*: Epiphyte in cloud forest.

*Distribution*: Ecuador, Peru. Alt. 2,130–2,500 m.

*Notes*: *Cyrtochilum ionodon* appears to be similar to its Peruvian congener *C. hastatum*. Both species have very similar lip form with very prominent callus exposed in the lip centre and papillate lower part. Both can be distinguished by form of the column wings—they are oblong, widened and truncate at the apex in *C. ionodon*, and obliquely oblanceolate, subacute in *C. hastatum*.

*Representative specimens*: ECUADOR. **Napo**: Quito-Baeza, 2500, Novomber 1980, *A. Andreetta 712* (SEL—Dalström, 2010); Cuyuja, 2,400 m, *C. Dodson 17074* (MO, QCNE, SEL—Dalström, 2010); km 73, 2,450 m, *C. Dodson & P. Dodson 16428* (RPSC—Dalström, 2010); Cosanga to Rio Aliso, 5 km SW of Cosanga, 2,130 m, *J. Kirkbridge & R. Chamba 4242* (NY, US—Dalström, 2010). PERU. *Laurentius ex J. Linden s.n*. (W-R!, UGDA-DLSz!—drawing). *Sine loc. Sine coll*. (W-R 2.1882!).

***Cyrtochilum hastiferum*** (Rchb.f. & Warsz.) Kraenzl., Notizbl. Bot. Gart. Berlin-Dahlem 7: 91. 1917. ≡ *Oncidium hastiferum* Rchb.f. & Warsz., Bonplandia (Hannover) 2: 102. 1854. TYPE: Ecuador. *Warszewicz s.n*. (holotype, W-R! 48973, UGDA-DLSz!—drawing). ≡ *Oncidium macranthum* var. *hastiferum* (Rchb.f. & Warsz.) C. Schweinf., Bot. Mus. Leafl. 14: 70. 1949.

**Figure 16:**
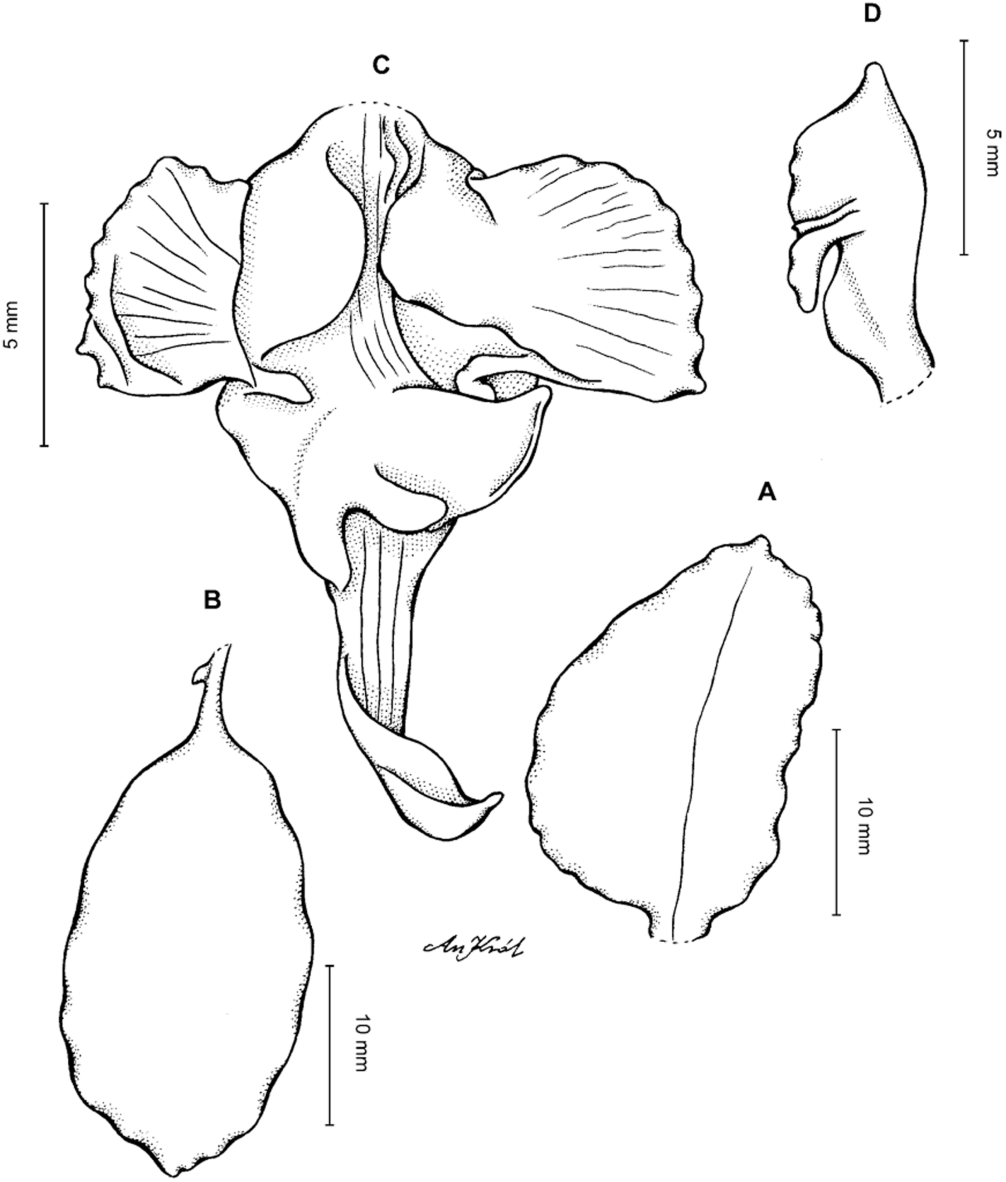
*Cyrtochilum hastiferum* (Rchb.f. & Warsz.) Kraenzl. (A) Petal; (B) Lateral sepal; (C) Lip; (D) Gynostemium. Drawn by Anna Król from *Warszewicz s.n*. (W-R).

Vegetative parts unknown, presumably similar to *C. macranthum*. Pedicel and ovary 35 mm long. Dorsal sepal not seen. Petals shortly unguiculate, 23 mm long in total, 16 mm wide, obliquely elliptic-ovate, obtuse, shortly apiculate, margin slightly undulate. Lateral sepals clawed; claw about 5–6 mm long, narrow, with rhombic wing on the outer margin near the base; blade 27 mm long, 14 mm wide, elliptic-obovate to elliptic, somewhat oblique, obtuse, shortly apiculate, margin slightly undulate. Lip 16 mm long and wide, hastate in general outline, base broadly cuneate, 3-lobed; lateral lobes obliquely wing-like, with somewhat crenate and undulate apical margin; callus very large, at the base of the middle lobe, consisting of single, high, horn-like keel, flanked by two smaller appendages and with an additional outgrowth; apical part ligulate-lanceolate, acute, acuminate, recurved. Gynostemium 8 mm long, slightly arcuate, with two lateral appendages on each side, larger one lanceolate, obtuse, smaller one linear (Fig. 16).

*Ecology*: No data.

*Distribution*: Ecuador, Peru? (Schweinfurth, 1961).

*Notes*: Dalström (2001) considered this species as conspecific with *C. macranthum*. In our opinion, however, this species is morphologically similar to *C. ruizii* from Peru rather than to *C. macranthum*. Both *C. hastiferum* and *C. ruizii* can be distinguished by the lip callus. It is confined to the basal part of the middle lobe in *C. hastiferum* and consists of single, high, horn-like keel, flanked by two smaller appendages and with an additional outgrowth. Lip callus of *C. ruizii* is formed of three low, longitudinal keels emerging from the base and extending to near the middle lobe, then spreading, the lateral pair ends in raised, blunt, angulate keels, while the central keel continues with two new emerging, lateral, spreading keels on each side of the angular, nose-like apex.

*Representative specimen*: ECUADOR. **Loja**: *J. Warszewicz s.n*. (W-R!, UGDA-DLSz!—drawing).

***Cyrtochilum ruizii*** Dalström & Deburghgraeve, Lankesteriana 12(2): 95. 2012. TYPE: Peru. *Dalström 3495* (holotype, USM).

**Figure 17:**
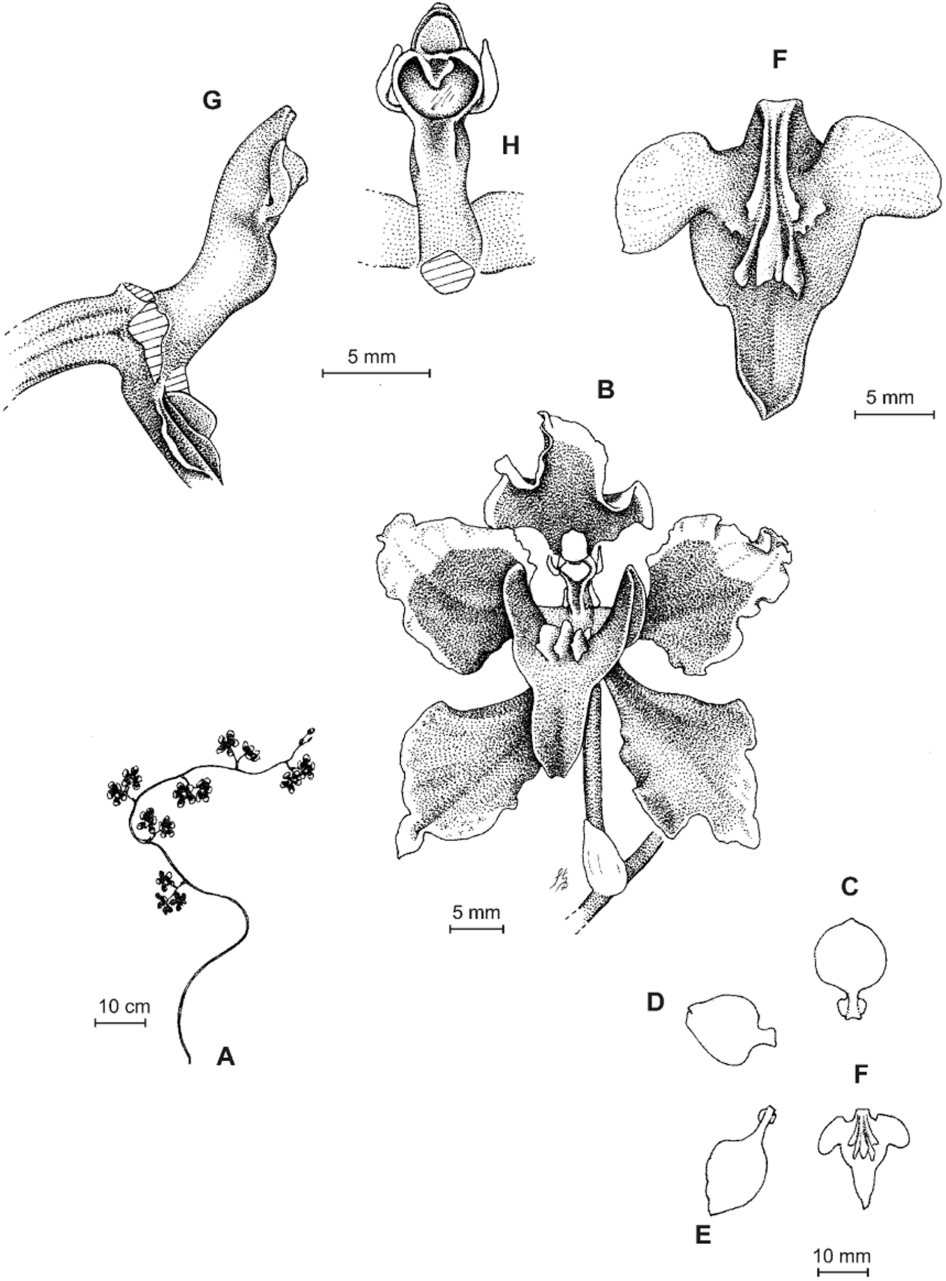
*Cyrtochilum ruizii* Dalström & Deburghgraeve. (A) Inflorescence; (B) Flower; (C) Dorsal sepal; (D) Petal; (E) Lateral sepal; (F) Lip; (G) Gynostemium, side view; (H) Gynostemium, front view. Drawn by Stig Dalström from *Dalström 3495* (USM).

Pseudobulbs caespitose, ovoid, unifoliate or bifoliate, surrounded basally by distichous, foliaceous sheaths. Leaves subpetiolate, conduplicate, obovate, obtuse to acuminate (no vegetative parts were included in the type specimen). Inflorescence up to more than 100 cm long, paniculate (tip broken off on type specimen, but much longer inflorescences have been observed), axillary, from the base of the uppermost sheaths, erect, then wiry and flexuous, with widely spaced few-flowered lateral branches. Flowers stellate to slightly campanulate, sometimes irregular but showy, sepals brown with whitish edges and apex, petals basally whitish to pale pink, then with brown irregular mottling and a white apical third, lip yellowish brown with yellow around the yellow callus. Floral bracts 10–12 mm long, appressed, involute, cucullate. Pedicel with ovary 20–30 mm long. Dorsal sepal clawed; claw 5–8 mm long, broadly winged; blade 17–21 mm long, about 15 mm wide, laminate, broadly ovate, obtuse, widely undulate. Petals clawed; claw 3 mm long; blade up to 20 mm long, about 15 mm wide, broadly ovate, obtuse, widely undulate. Lateral sepals clawed; claw up to about 7 mm long, narrow, basally winged; blade 20–21 mm long, about 13 mm wide, broadly elliptic-ovate, obtuse, widely undulate and slightly oblique. Lip about 22 mm long in total, about the same width, rigidly attached to the base of the gynostemium, cuneate, then hastate to cordate, trilobed with erect, obovate, weakly serrate and oblique lateral lobes, and a narrowly triangular, ligulate, apically slightly convolute and recurved, acute to slightly acuminate middle lobe; callus of three low, longitudinal keels, emerging from the base and extending to near the middle lobe, spreading, the lateral pair ends in raised, blunt, angulate keels, while the central keel continues with two new emerging, lateral, spreading keels on each side of the angular, nose-like apex. Gynostemium about 13 mm long, slightly sigmoid, with a slight swelling and two parallel short keels at the middle of the ventral side below the stigma, and with a pair of falcate, digitate wings on each side of the stigma (Fig. 17).

*Ecology*: Epiphyte or terrestrial in deforested montane area.

*Distribution*: Ecuador, Peru. Alt. about 2,700 m.

*Notes*: This species differs from both *C. deburghgraeveanum* and *C. cordatum* by the large and widely rounded, slightly spreading, unicolored brown lateral lobes of the lip, and by the attractive white petals, covered basally by a large brown blotch (Dalström, Deburghgraeve & Ruíz Perez, 2012). It is morphologically very similar to *C. hastiferum* but has different lip callus, consisting of 3 low, longitudinal keels described above.

*Representative specimens*: ECUADOR. **Loja**: “Rio Zenen”, color transparency by R. Thompson (Dalström, Deburghgraeve & Ruíz Perez, 2012). PERU. **Cajamarca**: Incahuási, about 2,700 m, 06°26.700S 079°01.177W, collected by Saul Ruiz, 23 May 2011, *S. Dalström 3495* (USM).

***Cyrtochilum serratum*** (Lindl.) Kraenzl., Notizbl. Bot. Gart. Berlin-Dahlem 7: 93. 1917. ≡ *Oncidium serratum* Lindl., Sert. Orchid.: t. 48. 1841. TYPE: Peru. *Mathews s.n*. (holotype, K-L).

**Figure 18:**
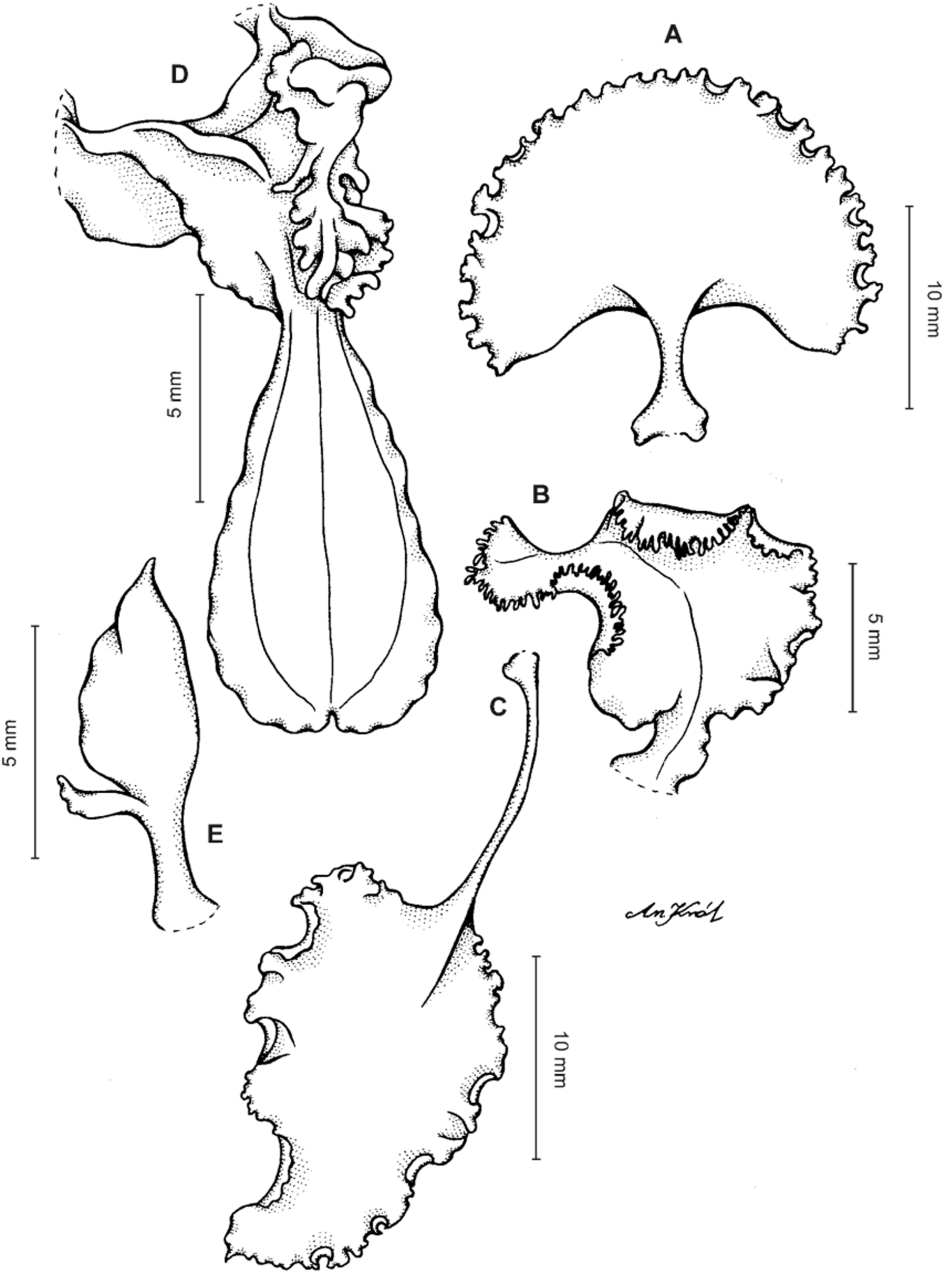
*Cyrtochilum serratum* (Lindl.) Kraenzl. (A) Dorsal sepal; (B) Petal; (C) Lateral sepal; (D) Lip; (E) Gynostemium, side view. Drawn by Anna Król from *Spruce 5282* (W-R).

Rhizome creeping, bracteate. Pseudobulbs 10–15 cm long, 3–4 cm wide, distant, elongate-ovoid, uni- or bifoliate, surrounded basally by 8 or more distichous foliaceaous sheaths. Leaves 45–50 cm long, 4–7 cm wide, conduplicate, subpetiolate, obovate, acute to acuminate. Inflorescence up to about 3 m long, axillary, erect, then wiry, paniculate with widely spaced, wiry, several-flowered branches. Floral bracts 16 mm long. Pedicel and ovary 40 mm long. Dorsal sepal clawed; claw up to 8 mm long, canaliculate, with a pair of obliquely subrectangular appendages at the base; blade 18 mm long, 26 mm wide, blade reniform, rounded, base subcordate, margins strongly undulate. Petals 20 mm long in total, 9 mm wide when spread, shortly clawed; blade oblong-ovate, obtuse, strongly falcate, margins strongly undulate-serrate. Lateral sepals clawed; claw 15 mm long, canaliculate, with a minute appendage at the base; blade 26 mm long, 16 mm wide, oblong ovate, subfalcate, apiculate, margins strongly undulate. Lip 16 mm long in total, hastate in outline, curved in natural position, base cuneate, 3-lobed; basal part 6 mm long, 12 mm wide across, trapeziform in outline, callus basal, large, dissected into numerous projections; apical part 10 mm long, 4 mm wide, oblong obovate, minutely bifid at the apex, margin slightly undulate. Gynostemium 8 mm long, lateral appendages oblong, apically shallowly 3-toothed (Fig. 18).

*Ecology*: Epiphytic or terrestrial growing on embankments.

*Distribution*: Ecuador, Peru. Alt. 1,300–3,100 m.

*Notes*: There is a group of species with strongly undulate petals, often touching each other at the apex and forming a kind of circle surrounding lip base ang gynostemium. Additionally, the apical part of the lip here is narrow and more or less ligulate to oblong elliptic. This group includes *C. serratum, C. kienastianum, C. ramiro-medinae, C. lamelligerum* and *C. methonica. Cyrtochilum serratum* is the only species of the group having petals with strongly undulate-serrate margins. Both petals are connate together along the apical margins and hard to separate. Cyrtochilum *kienastianum* can be easily recognized from all aforementioned species by completely different lip form. The unique character of *C. ramiro-medinae* is the gynostemium with two appendages composed of several series of toothed filaments. Cyrtochilum *lamelligerum* can be separated based on the large and massive, very complicated lip callus. Cyrtochilum *methonica* is characterized by the relatively narrow dorsal sepal hardly reaching 6 mm width.

*Representative specimens*: ECUADOR. **Bolívar**: Echeandía-Guanujo, 2,400 m, 8 July 1979, *L. Holm-Nielsen & R. Andrade 18569* (AAU—Dalström, 2010). **Cañar**: Km 110–115, Duran-Tambo, 1,700–1,900 m, 1 July 1960, *C. Dodson 114* (SEL—Dodson & Dodson, 1980). **Chimborazo**: Durán-Riobamba, 5 km N of Huígra, 3,000 m, 19 May 1945, *W. Camp E-3353* (AMES, NY, SEL—Dodson & Dodson, 1980). **Cotopaxi**: Near Macuchi on road Quevedo-Latacunga, 1,500 m, 10 July 1979, *C. Dodson 9288* (SEL—Dodson & Dodson, 1980). **El Oro**: Near Ayapamba, 1,300 m, 15 May 1962, *Mármol 23* (SEL—Dodson & Dodson, 1980). **Esmeraldas**: Lta-Cristal, km 10, 1,450 m, 29 December 1990, *C. Dodson & al. 18601* (MO—Dalström, 2010). **Imbabura**: Selva Alegre to Otovalo, 1,550 m, 28 February 1992, *S. Dalström 1558* (SEL—Dalström, 2010). **Pichincha**: Km 65 old road Quito-Santo Domingo, 1,600 m 3 July 1967, *C. Dodson et al. 3725* (SEL—Dodson & Dodson, 1980); km 29 old road Quito-Santo Domingo, 2,900 m, 22 October 1961, *C. Dodson & L. Thien 1026* (SEL—Dodson & Dodson, 1980); crater of Pululahua, 20 km north of Quito, 2,600–3,100 m, 12 May 1973, *L. Holm-Nielsen 5196* (SEL, AAU—Dodson & Dodson, 1980). *Sine loc., R. Spruce 5282* (W-R!, UGDA-DLSz!—drawing). PERU. *A. Mathews s.n*. (K-L).

***Cyrtochilum kienastianum*** (Rchb.f.) Kraenzl., Notizbl. Bot. Gart. Berlin-Dahlem 7: 91. 1917. *≡ Oncidium kienastianum* Rchb.f., Gard. Chron., n.s., 9: 558. 1878. TYPE: Colombia? *Roezl s.n*. (holotype, W-R! 48807, UGDA-DLSz!—drawing).

*Oncidium trilingue* Sander, Orchid. Guide: 193. 1901, *nom illeg*.

**Figure 19:**
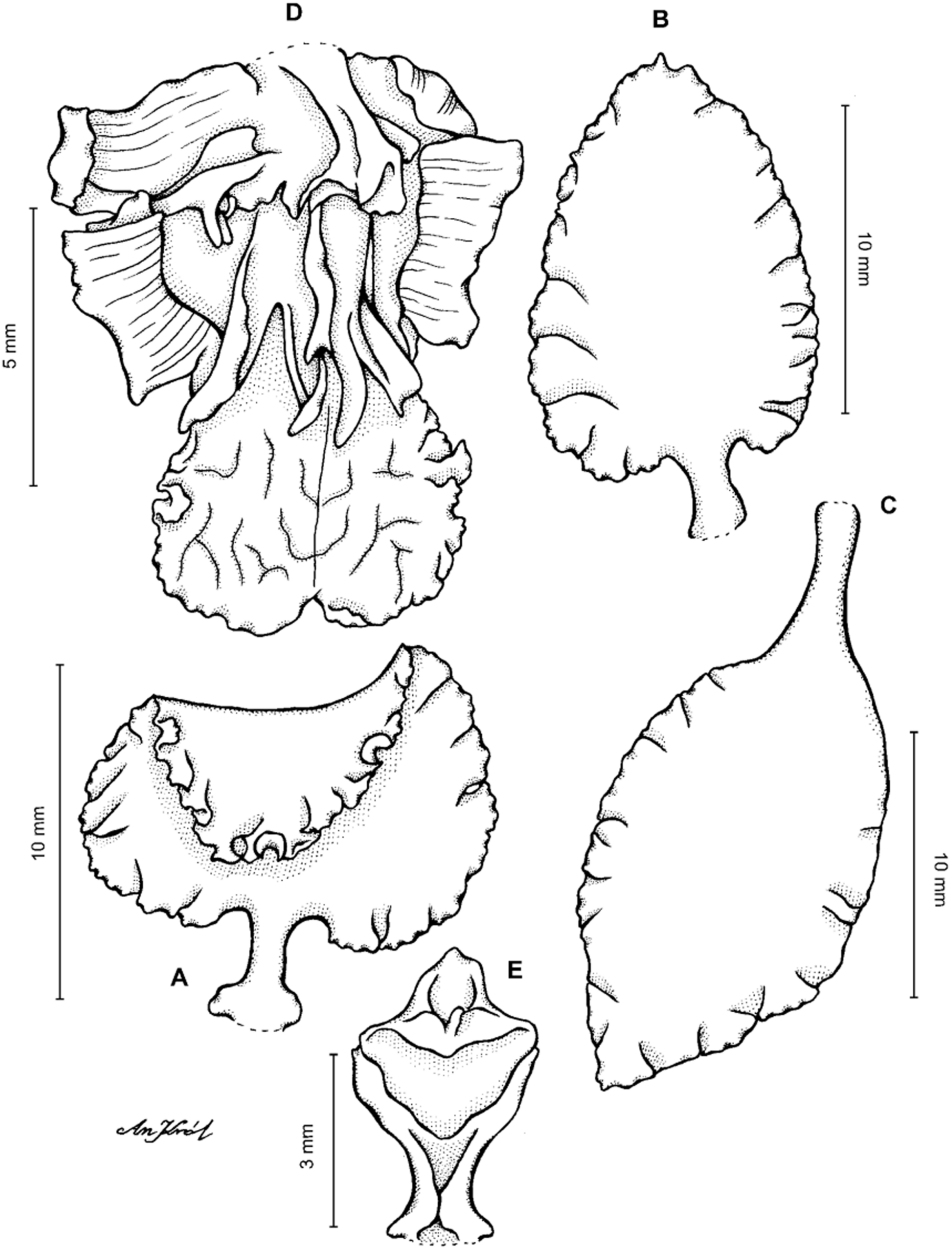
*Cyrtochilum kienastianum* (Rchb.f.) Kraenzl. (A) Dorsal sepal; (B) Petal; (C) Lateral sepal; (D) Lip; (E) Gynostemium. Drawn by Anna Król from *Kinest 9-79* (W-R).

**Figure 20:**
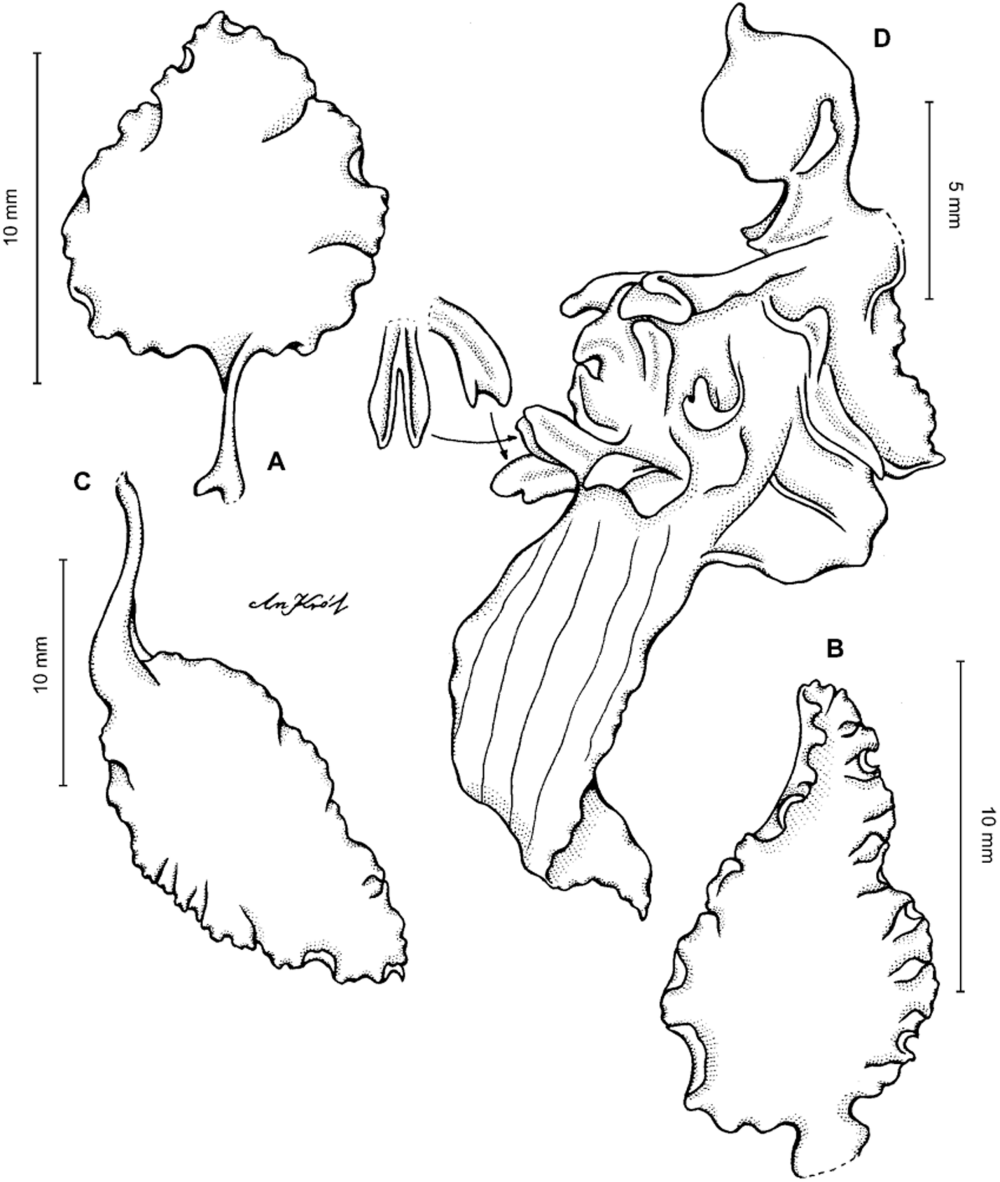
*Cyrtochilum kienastianum* (Rchb.f.) Kraenzl. (A) Dorsal sepal; (B) Petal; (C) Lateral sepal; (D) Lip and gynostemium, side view. Drawn by Anna Król from *Warszewicz s.n*. (W-R) type of *Oncidium trilingue* Sander.

Pseudobulbs about 8 cm long, aggregated according to original description, oblong-ovoid or ellipsoid, more or less ancipitous, bifoliate, clothed at the base by several pairs of conduplicate, leaf-bearing sheaths. Leaves more than 50 cm long, 2.5 cm wide according to original description, oblanceolate-oblong (“linear” in original description), acute or acuminate. Scape very long, twining, paniculate above, the short, few-flowered branches described as 3–4 cm long. Flowers rather large, sepals brown with pale yellow margins, petals and lip yellowish with brown or purple spots. Floral bracts about 20 mm long, oblong-ovate, acute. Pedicellate ovary 30–35 mm long. Dorsal sepal clawed; claw about 5–6 mm long, with prominent basal wings; blade 15 mm long, 9.5–10 mm wide, ovate to triangular-ovate, acute to apiculate, margins crenate-undulate, base truncate. Petals clawed; claw about 3 mm long, wide; blade 14–16 mm long, 8–12 mm wide, broadly ovate, more or less sigmoid, subacute, margin strongly undulate-crenate. Lateral sepals clawed; claw up to 9 mm long, narrow, wingless; blade 20–23 mm long, 7.5–11 mm wide, elliptic, apiculate, more or less sigmoid, margins undulate. Lip about 11–16 mm long in total, base truncate, 3-lobed just above base; basal part about 5 mm long, 8–13 mm wide across, subrectangular, lobules subquadrate, truncate, deeply incised in basal third; callus prominent, consisting of a 3-carinate crest, the central keel highest, the lateral ones semilunate-curved with few lamellae on each side and at the apex; apical part up to 10 mm long, 5 mm wide, ligulate, acute, occasionally rounded, margins more or less undulate. Gynostemium 5–9 mm long, arcuate, with a pair of angular, fleshy processes at the base and a pair of oblong, shallowly 3-dentate appendages above (Figs. 19 and 20).

*Ecology*: No data.

*Distribution*: Peru (Schweinfurth, 1961), Colombia.

*Notes*: *Cyrtochilum kienastianum* is characterized by the ligulate, relatively broad lip middle lobe with more or less undulate margins. Lateral lobes of the lip are relatively short and wide and split into two parts each. Otherwise, this species resembles somewhat *C. serratum*.

*Representative specimens*: COLOMBIA. *Sine loc., B. Roezl s.n*. (W-R!). *Sine loc., Kinest 9–79* (W-R!, UGDA-DLSz!—drawing). *Sine loc., J. Warszewicz s.n*. (W-R!, UGDA-DLSz!—drawing).

***Cyrtochilum ramiro-medinae*** P.Ortiz, Orquideólogo Supl. 1: 15. 2012. TYPE: Colombia. *Medina 456* (holotype, HPUJ).

Rhizome ascending. Pseudobulbs 12 cm distant, 9.5–10.5 cm long, 5–6 cm wide, oblong-ovate, narrowed at the apex, surrounded at the base by 3 foliar sheaths at each side up to 49 cm long, 4.5 cm wide, apically 2–3-foliate. Leaves 42 cm long, 4.5 cm wide. Inflorescences 2, from the axil of the foliar sheaths, 330 cm long, one with a total of 83 flowers, other with 93 flowers, main scape voluble, branched, lateral branches fractiflex, flowers separated with triangular floral bracts 19 mm long. Flowers medium-sized, sepals brown, apices and margins yellow with brown dots, basal half of the petals brown, apical half yellow-greenish, lip yellowish with brow spot in the centre, callus white, column yellowish with violet shading. Dorsal sepal 30 mm long, 18 mm wide, base unguiculate, apex acute, with a prominent vein on the underside. Petals 31 mm long, 6 mm wide, falcate, curved inward, forming a semicircle, apex acute. Lateral sepals 41 mm long, 11 mm wide, connate at the base for 13 mm, oblong-elliptic, subacute, central vein prominent on the underside. Lip 20 mm long, hastate in general outline, 3-lobed; lateral lobes quadrate, decurved, 4 mm long, 4 mm wide, with semiglobose convexivity in the middle; callus at the base of the lip prominent, with a mass projected forward like a narrow nose, concave underneath, with 2 series of small tubercles at each side, upper ones white, lower ones green; middle lobe 12 mm long, 4 mm wide, ligulate, concave, retroflex. Gynostemium sigmoid, with two appendages composed of several series of toothed filaments, the central one longer.

*Ecology*: Epiphyte.

*Distribution*: Colombia. Alt. 2,400 m.

*Notes*: This species can be easily separated from any other representatives of this group by peculiar gynostemium structure, precisely presence of a pair of column wings in the form of bird wings, composed of several series of toothed filaments, the central one longer.

*Representative specimen*: COLOMBIA. **Putumayo**: San Francisco, Vereda San Pablo Alto, 2,400 m, 15 December 2006, *R. Medina 456* (HPUJ).

***Cyrtochilum lamelligerum*** (Rchb.f.) Kraenzl., Notizbl. Bot. Gart. Berlin-Dahlem 7: 92. 1917. ≡ *Oncidium lamelligerum* Rchb.f., Gard. Chron., n.s., 6: 808. 1876. TYPE: Ecuador. *Klaboch s.n*. (holotype, W-R! 48960; UGDA-DLSz!—drawing) & *Hruby s.n*. (paratype: W-R! 48960) & *Wilkes 87* (paratype: W-R! 48960).

**Figure 21:**
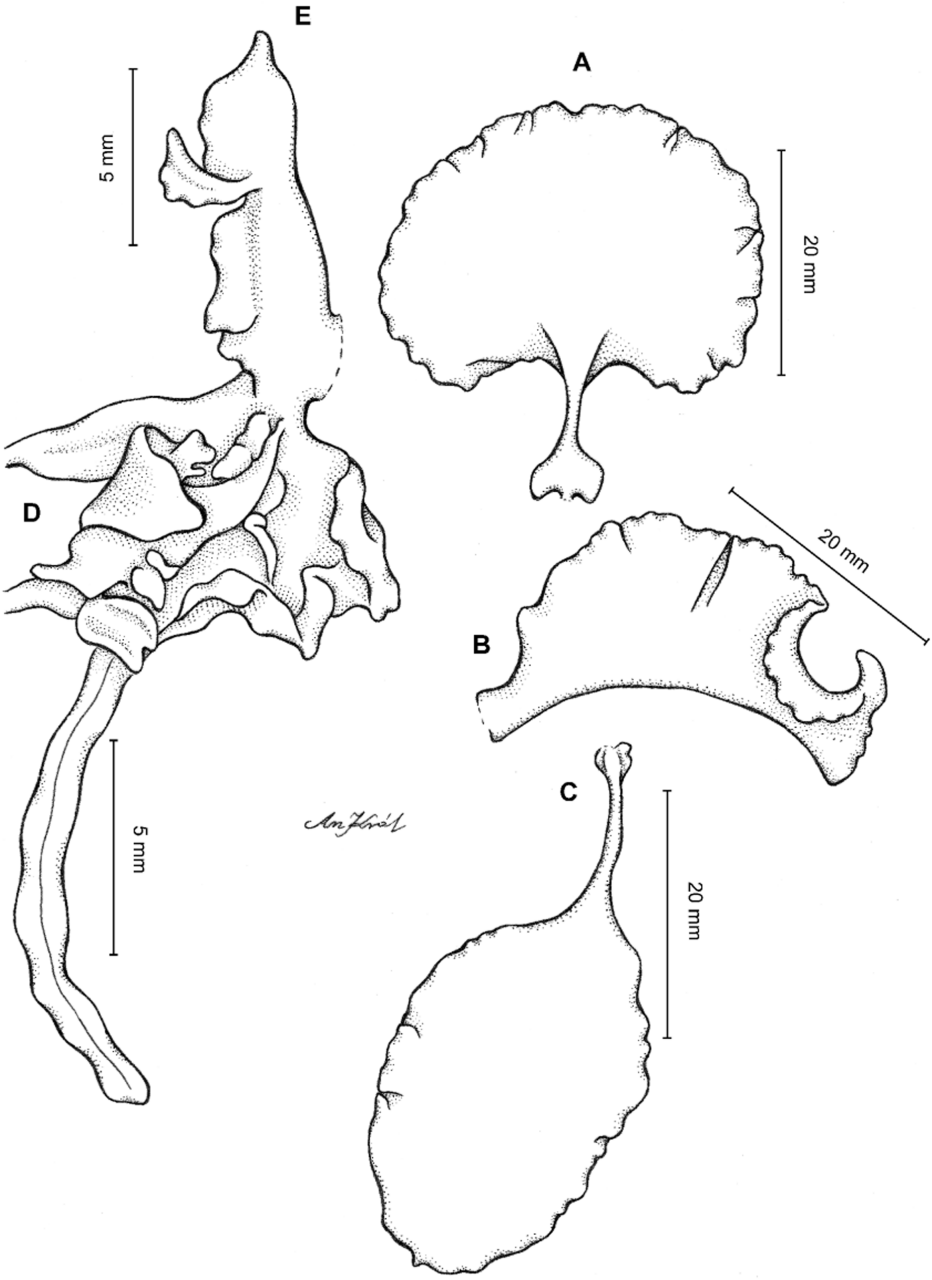
*Cyrtochilum lamelligerum* (Rchb.f.) Kraenzl. (A) Dorsal sepal; (B) Petal; (C) Lateral sepal; (D) Lip; (E) Gynostemium, side view. Drawn by Anna Król from *Klaboch s.n*. (W-R).

**Figure 22:**
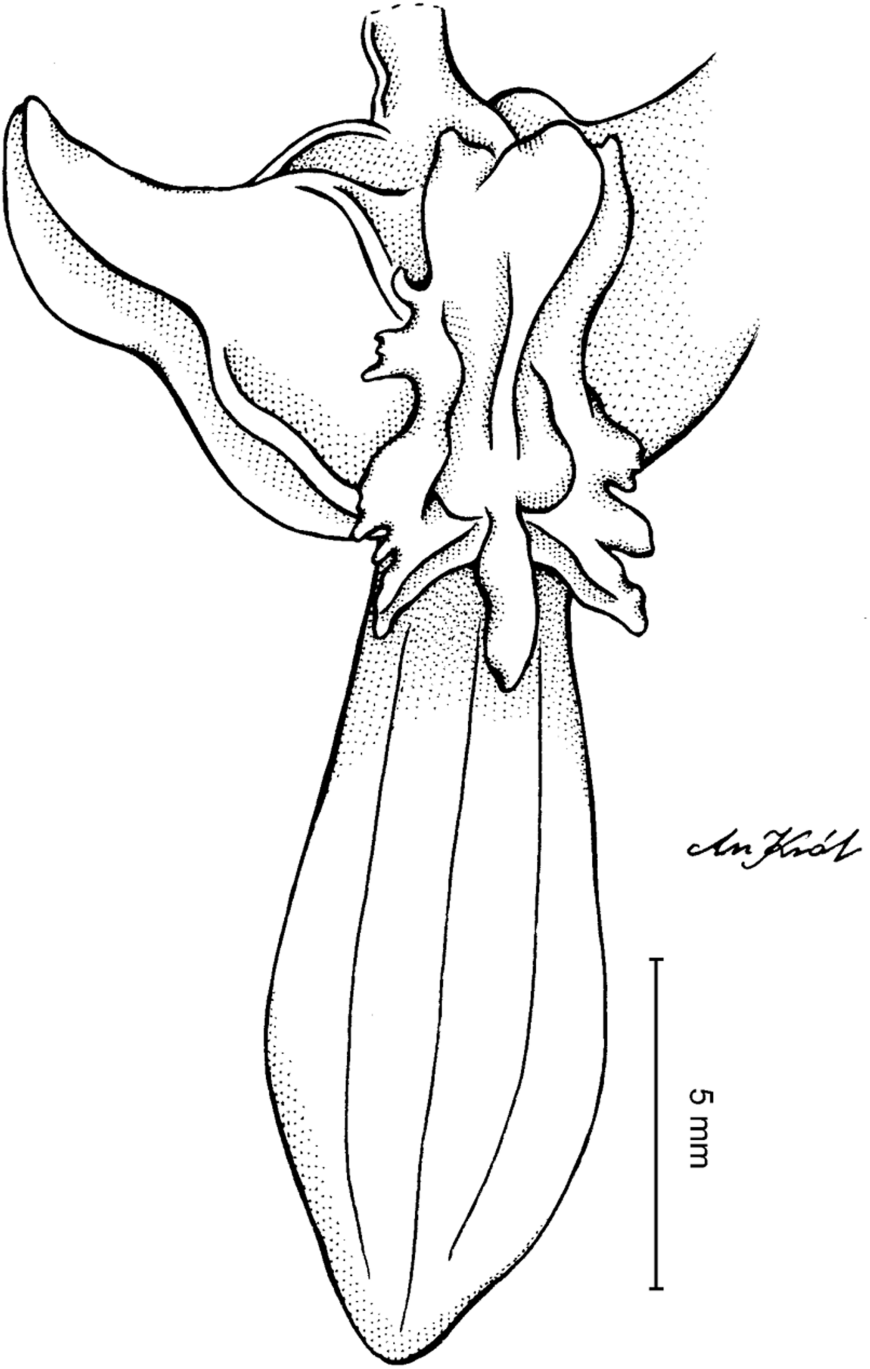
*Cyrtochilum lamelligerum* (Rchb.f.) Kraenzl. Lip. Drawn by Anna Król from *Vogel 287* (AMES).

Rhizome creeping, bracteate. Pseudobulbs more or less distant. Leaves not seen. Inflorescence several meters long, wiry, with well-spaced, few-flowered branches. Flowers large, conspicuous, showy, sepals bronzy-brown, petals yellow with brown spots, lip reddish-brown, with yellow apex, callus white and purple. Floral bracts 5–20 mm long. Pedicel and ovary up to 80 mm long. Dorsal sepal shortly clawed; claw 10–12 mm long, narrow, canaliculate, with prominent elliptic-rhombic wings just above the base; blade 23–25 mm long, 30 mm wide, reniform, base subcordate, margins somewhat irregularly undulate, apex rounded. Petals clawed; claw about 4–5 mm long, basally somewhat winged; blade 27–28 mm long, 20 mm wide when spread, somewhat oblique, elliptic-ovate, obtuse or truncate at the apex, margins irregularly undulate. Lateral sepals prominently clawed; claw 12–14 mm long, narrow, with basal wing on the outer margin; blade 28–30 mm long, 20 mm wide, obliquely elliptic-ovate in general outline, basally rounded, obtuse at the apex, margins very slightly undulate. Lip 18–19 mm long in total, hastate in outline, curved in natural position exposing large and massive callus; basal part 8 mm long, about 11–12 mm wide, broadly triangular, base truncate-subcordate, convex along the centre; callus very complicated, rectangular-pandurate in outline when see from front, consisting of high median ridge, more or less triangular, flanked by various outgrowths, margins entire; apical part 11 mm long, about 3–4 mm wide, ligulate, attenuate towards apex. Gynostemium 9 mm long, subarcuate, connate basally with the lip, lateral appendages obliquely obovate, with irregularly dentate lower margin, reaching the anther base (Figs. 21 and 22).

*Ecology*: Epiphytic or terrestrial along road-cuts in cloud forest and upper montane wet forest.

*Distribution*: Colombia, Ecuador. Alt. 2,600–3,000 m.

*Notes*: *Cyrtochlium lamelligerum* can be easily separated from all other species of this group by having a prominent, large lip callus, which main tissue is rectangular-pandurate in outline when see from front, with truncate base. Garay (1978) cited *Klaboch 4* collection as the type of the species. Unfortunately, we were not able to trace it in W.

*Representative specimens*: COLOMBIA. **Nariño**: Epiphytisch im niedrigen Gesprüpp der Nebelregion am Wege von El Tambo nach Chiles (Strasse nach Mayasquer), 3,000 m, 11 July 1956, *St. Vogel 287* (AMES!, UGDA-DLSz!—drawing). ECUADOR. **Bolivar**: Alt. 2,600 m, *S. Dalström 668* (SEL—Dalström, 2010). **Cañar**: Cola de San Pablo, collected and cultivated in Cuenca by Alfonso Pozo, *C. Dodson 12875* (SEL—Dodson & Dodson, 1984). *Sine loc., E. Klaboch s.n*. (W-R! 48960, UGDA-DLSz!—drawing), *J. Hruby s.n*. (W-R! 48960), *Wilkes 87* (W-R! 48960).

***Cyrtochilum methonica*** (Rchb. f.) Kraenzl., Notizbl. Bot. Gart. Berlin-Dahlem 7: 91. 1917. ≡ *Oncidium methonica* Rchb. f., Linnaea 41: 21. 1877[1876]. TYPE: Bolivia. *Pearce s.n*. (holotype, W-R! 36960, UGDA-DLSz!—drawing).

**Figure 23:**
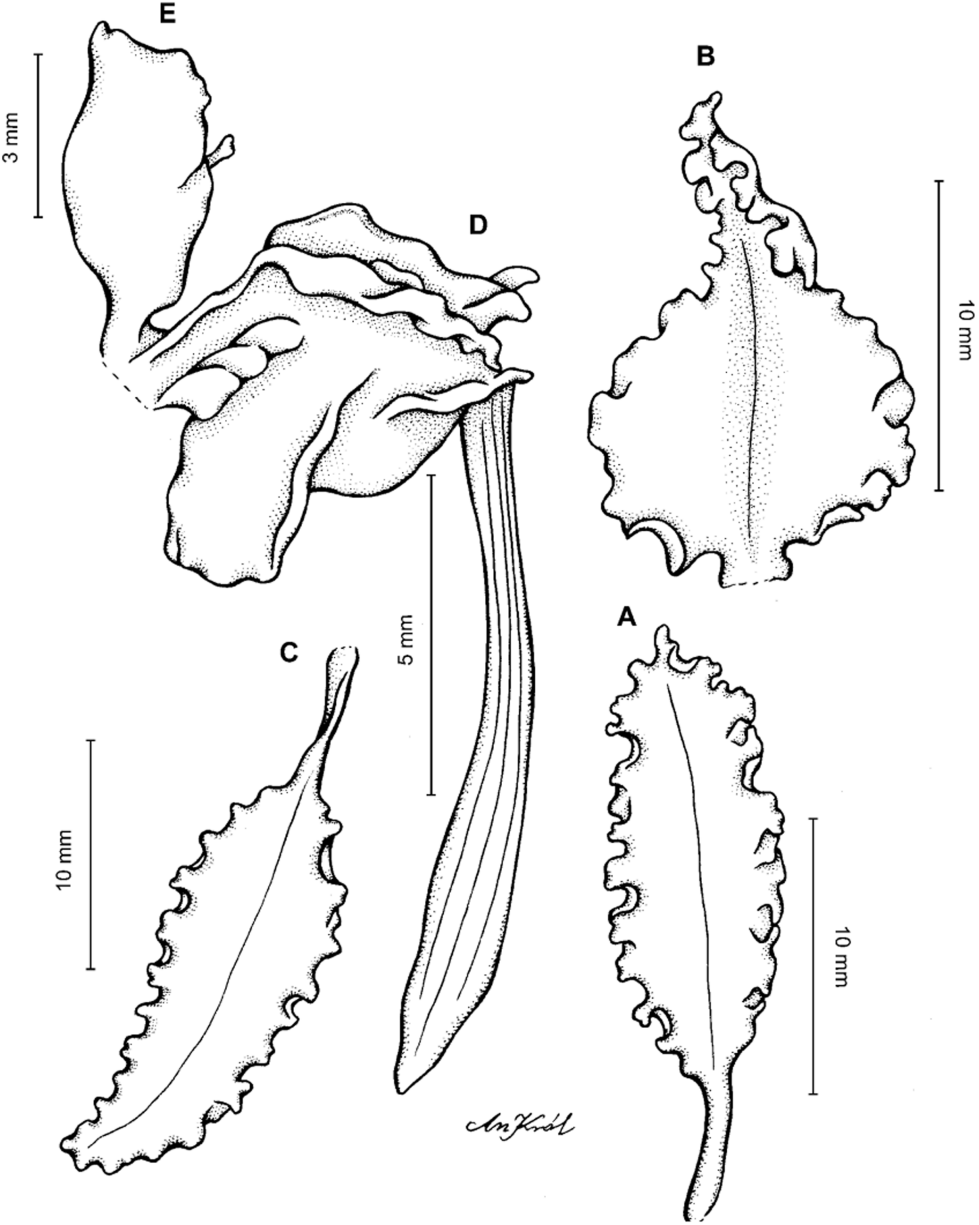
*Cyrtochilum methonica* (Rchb. f.) Kraenzl. (A) Dorsal sepal; (B) Petal; (C) Lateral sepal; (D) Lip; (E) Gynostemium, side view. Drawn by Anna Król from *Pearce s.n*. (W-R).

Rhizome creeping. Pseudobulbs more or less distant. Leaves not seen. Inflorescence several meters long, wiry, with well-spaced, few-flowered branches. Flowers large, conspicuous, showy, sepals bronzy-brown, petals yellow with brown spots, lip reddish-brown, with yellow apex, callus white and purple. Floral bracts 10 mm long. Pedicel and ovary 30 mm long. Dorsal sepal shortly clawed; claw about 5 mm long, narrow, canaliculate, wingless; blade 18–20 mm long, 6 mm wide, oblong elliptic, base cuneate, margins stringly undulate, apex subobtuse. Petals clawed; claw about 1–2 mm long, basally somewhat winged; blade 15–16 mm long, 7–8 mm wide when spread, somewhat oblique, ovate, attenuate towards obtuse or truncate apex, margins strongly undulate. Lateral sepals basally connate, prominently clawed; claw 5–7 mm long, narrow, wingless; blade about 20 mm long, 6–7 mm wide, obliquely ovate-lanceolate in general outline, basally cuneate, obtuse at the apex, margins strongly undulate. Lip 16–18 mm long in total, hastate in outline, curved in natural position exposing large and massive callus; basal part 4 mm long, about 12 mm wide across, broadly triangular, base truncate-subcordate, convex along the centre; callus consisting of high median ridge, more or less rhombic, flanked by two additional ridges; apical part 11 mm long, about 1.5–2 mm wide, linear, attenuate towards apex. Gynostemium 6 mm long, erect, connate basally with the lip, lateral appendages linear, reaching the anther base (Fig. 23).

*Ecology*: No data.

*Distribution*: Bolivia.

*Notes*: *Cyrtochilum methonica* is the only species of this group having narrow, oblong elliptic to ovate-lanceolate sepals. Dorsal sepal is about 3.5 times longer than wide, whereas in other species dorsal sepal is almost as long as wide.

*Representative specimen*: BOLIVIA. *G. Pearce s.n*. (W-R! 48960, UGDA-DLSz!—drawing).

***Cyrtochilum cryptocopis*** (Rchb.f.) Kraenzl., Notizbl. Bot. Gart. Berlin-Dahlem 7: 92. 1917. ≡ *Oncidium cryptocopis* Rchb.f., Gard. Chron. 1870: 827. 1870. TYPE: Peru. *Bull 789* (holotype, W-R! 48871; UGDA-DLSz!—drawing).

**Figure 24:**
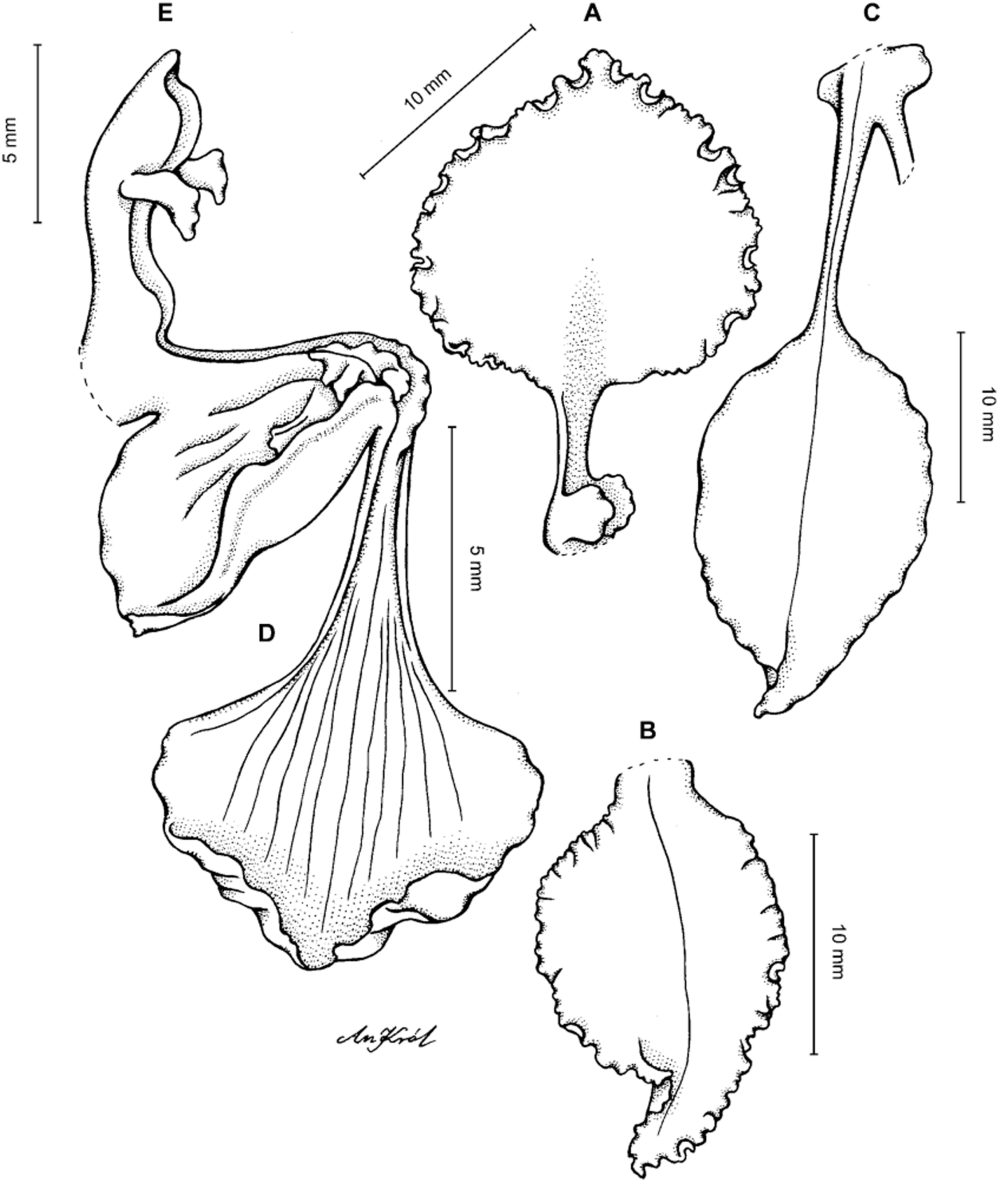
*Cyrtochilum cryptocopis* (Rchb.f.) Kraenzl. (A) Dorsal sepal; (B) Petal; (C) Lateral sepal; (D) Lip; (E) Gynostemium. Drawn by Anna Król from *Bull 789* (W-R).

Pseudobulbs aggregated, much compressed, 10–12.7 cm long, very narrowly ovoid to cylindric, bifoliate, enclothed at the base by several (up to 3) pairs of leaf-bearing sheaths. Leaves about 30 cm long, 3 cm wide, oblong-linear or lorate, slightly broader above the middle, acute. Inflorescence about 1–1.5 m or more long, twining above, paniculate, with remote branches which are short, twining and few- (often 1–) flowered. Flowers large, conspicuous, showy, pale chestnut-brown with the margins of the sepals and petals golden yellow and the mid-lobe of the lip yellow. Floral bracts 12 mm long. Pedicel and ovary about 45 mm long. Dorsal sepal clawed; claw 6–7 mm long, narrow, canaliculate, with prominent elliptic-pentagonal wings just above the base; blade 16 mm long and wide, suborbicular-ovate, base truncate, margins strongly and irregularly undulate, somewhat crenate, apex obtuse. Petals clawed; claw about 2 mm long; blade 17 mm long in total, 12 mm wide when spread, obliquely ovate-lanceolate, obtuse at the apex, margins irregularly undulate and crenate, the upper half of blade strongly recurved. Lateral sepals prominently clawed; claw 15–18 mm long, narrow, with prominent basal wing on the outer margin, connate along the inner margins in the basal 5 mm; blade 18–21 mm long, 13 mm wide, obliquely ovate in general outline with lanceolate and recurved acute apex, base cuneate, margins entire, non-undulate. Lip 17 mm long in total, curved in natural position exposing relatively small callus; basal part 6 mm long, about 8–9 mm wide, almost triangular, base truncate, convex along the centre, callus complicated, papillate, consisting of narrow central ridge, flanked by two ridges variously lobed, margins entire; apical part 11 mm long, about 8 mm wide, clawed in the lower part, gradually transforming above cuneate base into prominent flabellate blade, the apex rounded somewhat undulate. Gynostemium 9 mm long, subarcuate, connate basally with the lip, lateral appendages obliquely malleolate, with truncate apex, not reaching the anther base (Fig. 24).

*Ecology*: Epiphyte.

*Distribution*: Ecuador, Peru. Alt. 1,300–1,800 m.

*Notes*: *Cyrtochilum cryptocopis* has petals of similar form as the previously mentioned species, i.e. blade is strongly recurved in the upper part, and margins are irregularly undulate and crenate. It is unique in the genus by lip form. Its lower part is obtriangular, basally truncate, with relatively small callus, the middle lobe is flabellate and set on prominent, narrow claw, hence appearing as additional sepal.

*Representative specimens*: ECUADOR**. El Oro**: Pasaje-Zaruma, Inca Paccha, 1,800 m, *A. Hirtz et al. 3868* (RPSC), Ayapamba, Cerro las Palmas, 1,300 m, *Mármol 6* (SEL—Dalström, 2010), Buena Vista-Paccha, km 35, 1,685 m, *M. Whitten et al. 978* (QCNE—Dalström, 2010). **Imbabura**: Selva Alegre, 1,800 m, *A. Hirtz 338* (SEL—Dalström, 2010). PERU. *Sine loc., Bull 789* (W-R! 48871; UGDA-DLSz!—drawing).

***Cyrtochilum betancurii*** Giraldo & Dalström, Lankesteriana 12(3): 137. 2012. TYPE: Colombia. *Betancur 14882* (holotype, COL).

Pseudobulbs distant on a creeping, bracteate rhizome, oblong ovoid and slightly compressed, about 4.3 cm long, 2.1 cm wide, unifoliate or bifoliate, surrounded basally by 4–6 distichous foliaceous sheaths. Leaves subpetiolate, to about 40–50 cm long, 5–6 cm wide, conduplicate, oblanceolate, acute. Inflorescence axillary from the uppermost sheath, up to about 2.8 m long, paniculate, erect then wiry, straight to loosely flexuous, with a basal longer branch, and then several widely spaced, short, few-flowered lateral branches, carrying in total 16–18 flowers. Flowers stellate and showy. Floral bracts about 10 mm long, appressed, scale-like. Pedicel with ovary about 45 mm long. Dorsal sepal 36 mm long, 24 mm wide, basally auriculate, spathulate, broadly cordate, distinctly undulate, obtuse to acute and slightly oblique, dark brown with yellow border. Petals about 25 mm long, 18 mm wide, broadly linear and shortly spathulate, then truncate to cordate, distinctly undulate, obtuse to acute and slightly oblique, dark brown with a yellow border. Lateral sepals basally auriculate and connate for 6–8 mm, then spreading, about 74 mm long, 23 mm wide, elongate and narrowly spathulate, then cordate, slightly undulate, obtuse to rounded and slightly oblique, dark brown. Lip dark brown with yellow border and callus, rigidly attached to the base of the column through a narrow and terete claw, then truncate, distinctly pandurate with spreading, slightly oblique, broadly auriculate, slightly serrate lateral lobes, and a about 4 mm broad isthmus below the widely spreading and broadly dolabriform, obtuse to acute, emarginate, revolute middle lobe, 18 mm long and wide; callus complex and fleshy, emerging near the base of the lateral lobes and extending for about 5 mm, consisting of an erect, table-like, tricarinate structure with several lateral, spreading denticles, with an additional series of spreading tubercles or denticles on each side, and an apical, central, longitudinal and triangular keel, with spreading, dorsally flattened, fleshy, lateral keels. Gynostemium purplish brown, stout, erect in about 90° angle from the base of the lip then slightly curved towards the lip near the apex.

*Ecology*: Epiphytic in cloud forest.

*Distribution*: Colombia. Alt. 1,600–1,800 m.

*Notes*: While describing *C. betancurii* Giraldo & Dalström (2012) compared it to the Ecuadorian *C. cryptocopis* and *C. trifurcatum*, stated that it has a different ventral structure and much narrower wings of the column, and also by the much broader middle lobe of the lip. This species, however, is hard to confuse with any other *Cyrtochilum* species known so far, especially by peculiar lip form whose middle lobe resembles a kind of horseshoe. In similar *C. methonica* the apical part of the lip is linear, attenuate towards apex. The above characteristic of *C. betancurii* is based entirely on the protologue and illustration provided by Giraldo & Dalström (2012).

*Representative specimens*: COLOMBIA. **Antioquia**: Mun. Urrao. Parque Nacional Natural las Orquídeas, in cloud forest, 1,600–1,800 m, 2 February 2011, *J. Betancur 14882* (COL).

**(2) *Diceratum*-group**

Lip pandurate in general outline, expanded apically to form a wide plate, wider than basal part. Gynostemium connate with the lip at the base only, then abruptly upcurved.

Two species of this group are known mostly from Colombia, with *C. diceratum* found also in Ecuador.

### Key to the species

1 Blade of dorsal sepal as long as wide, or narrower, base cuneate*C. diceratum*
1* Blade of dorsal sepal wider than long, base cordate*C. englerianum*

***Cyrtochilum diceratum*** (Lindl.) Kraenzl., Notizbl. Bot. Gart. Berlin-Dahlem 7: 95. 1917. ≡ *Oncidium diceratum* Lindl., Fol. Orchid. 6: 6. 1855. TYPE: COLOMBIA. *Jameson s.n*. (holotype, K).

= *Cyrtochilum fallens* (Rchb.f.) Kraenzl., Notizbl. Bot. Gart. Berlin-Dahlem 7: 5. 1917. ≡ *Oncidium fallens* Rchb.f., Flora 69: 549. 1886. TYPE: COLOMBIA. *Lehmann 3615* (holotype, W).

**Figure 25:**
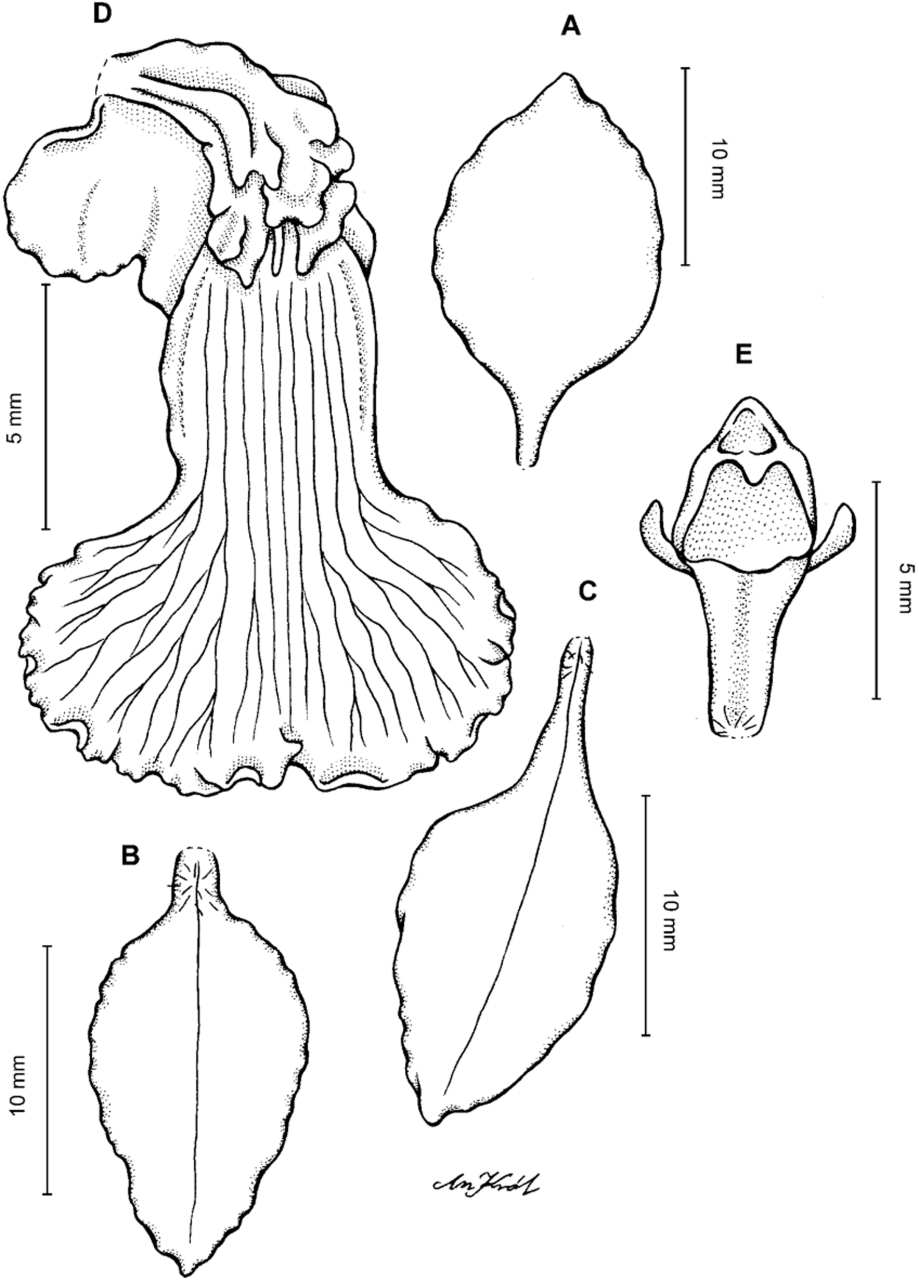
*Cyrtochilum diceratum* (Lindl.) Kraenzl. (A) Dorsal sepal; (B) Petal; (C) Lateral sepal; (D) Lip; (E) Gynostemium. Drawn by Anna Król from *Garay 468* (AMES).

**Figure 26:**
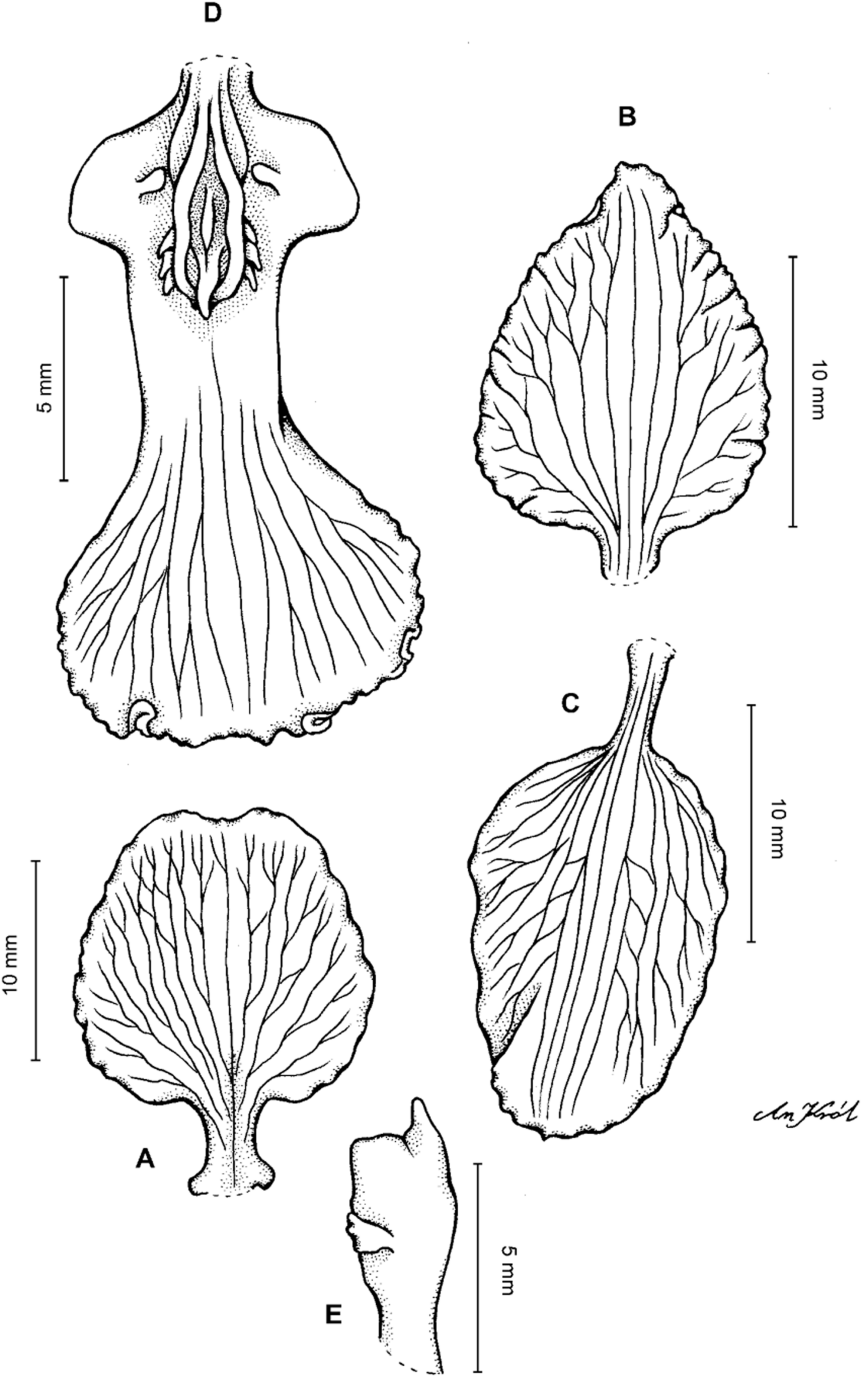
*Cyrtochilum diceratum* (Lindl.) Kraenzl. (A) Dorsal sepal; (B) Petal; (C) Lateral sepal; (D) Lip; (E) Gynostemium. Drawn by Anna Król from *Zarucchi & Echeverry 5159* (MO).

**Figure 27:**
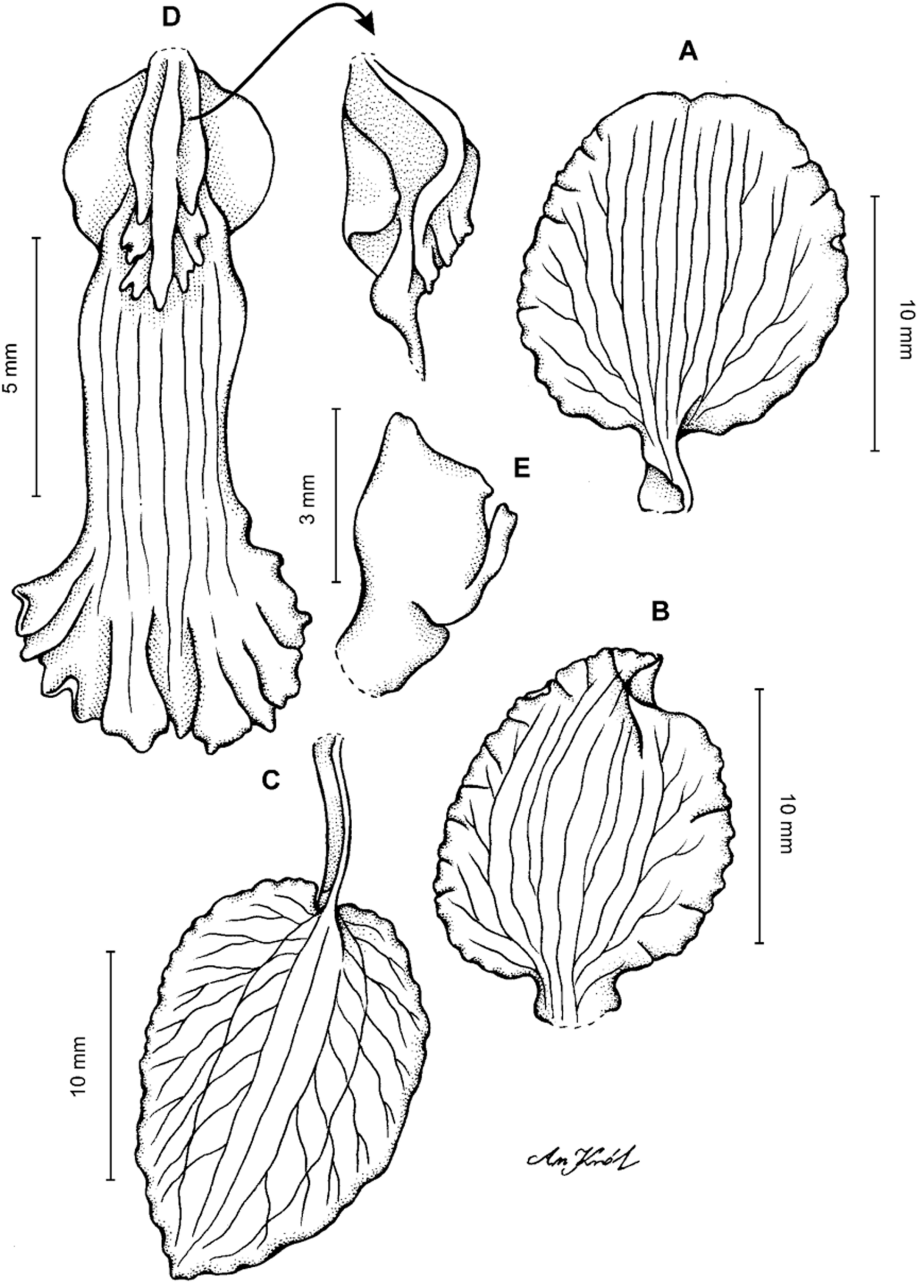
*Cyrtochilum diceratum* (Lindl.) Kraenzl. (A) Dorsal sepal; (B) Petal; (C) Lateral sepal; (D) Lip; (E) Gynostemium, side view. Drawn by Anna Król from *Torres et al. 2054* (COL).

Rhizome creeping. Pseudobulbs distant, 5–10 cm long, 1–2 cm in diameter, ovoid, supported basally by foliaceous sheaths, bifoliate. Leaves (15) 23–50 cm long, (1.5) 2–4 cm wide, linear-oblanceolate, acute. Inflorescence about 100 cm long, paniculate, wiry, with a few spaced few-flowered branches. Flowers large, conspicuous, showy, sepals brown, petals brown with a narrow yellow rim, lip brown with yellow apex of apical lobe, callus yellow. Floral bracts 13–15 mm long. Pedicel and ovary 32–50 mm long. Dorsal sepal clawed; claw 3–6 mm long, rather narrow, canaliculate, usually with prominent elliptic wings just above the base; blade 14–19 mm long, up to 19 mm wide, suborbicular-ovate to ovate, base cuneate, margins slightly irregularly undulate, apex subobtuse, truncate or emarginate. Petals clawed; claw 2–3 mm long, wide; blade 14–18 mm long, 12–17 mm wide when spread, obliquely elliptic-ovate, subobtuse at the apex, margins slightly undulate. Lateral sepals prominently clawed; claw 6–8 mm long, narrow, wingless; blade 16–22 mm long, 12–17 mm wide, obliquely elliptic in general outline, basally cuneate, obtuse at the apex, margins somewhat undulate. Lip 14–21 mm long in total, curved in natural position exposing massive callus; basal part 4–7 mm long, about 5–12 mm wide, suborbicular, broadly deltoid to hastate in outline, base cuneate, convex along the centre; callus complicated, consisting of 3 narrow ridges, flanked by some lobules on each side; apical part 10–14 mm long, ligulate in the basal half, then expanded into transversely elliptic to almost flabellate plate about 7–10 mm wide, more or less crispate and crenate along apical margin. Gynostemium 4.5–8 mm long, sigmoid, connate basally with the lip, lateral appendages obliquely triangular to oblong, upcurved, not reaching the anther base (Figs. 25–27).

*Ecology*: Terrestrial or epiphytic, growing on in humid montane forest, on steep embankments and rocky cliffs, also found on fallen rotten branch in disturbed subparamo vegetation.

*Distribution*: Colombia, Ecuador. Alt. 2,600–3,500 m.

*Notes*: Both species of this group are similar morphologically. In our opinion discriminative characters warranting a proper determination are form of dorsal sepal, especially its base (cuneate in *C. diceratum* vs cordate in *C. englerianum*) and lip apical lobe (crispate in the former vs non-undulate in the latter species). Further study should reveal if they are separate species.

*Representative specimens*: COLOMBIA. **Antioquia**: Mpio. Medellin. Cerro Padre Amaya. 8.4 km from Medellin-Santa Fe de Antioquia highway on road to summit. Disturbed subparamo vegetation. 6°16′N 75°14′W. Alt. 2,770 m. 30 March 1987. *J. Zarucchi & B. Echeverry 5159* (HUA, MO!, UGDA-DLSz!—drawing). **Caldas**: Carretera entre Manizales y el Hotel Termales del Ruiz. Alt. 3,000–3,500 m. 8 June 1966. *E. Forero, M. Murillo & N. Montenegro 559* (COL!). **Cauca**: Purace. Carretera de Popayán al PNN Purace. Alt. 3,200 m. 12 June 1983. *S. Hoyos & J. Santa 216* (MO!); Macizo Colombiano. Paramo de Las Papas. Entre Letreros y Santo Domingo. Alt. 3,350 m. 16 September 1958. *J. Idrobo, J.M. Pinto & P. Bischler 3388* (COL!). **Chocó**: Macizo del Tamana. Campamento Las Pavas. Margen oriental del rio Tatama. Alt. 2,800 m. 19 February 1983. *J. Torres, O. Rangel, P. Franco, A. Cleef & S. Salamanca 2054* (COL!, UGDA-DLSz!—drawing). **Cundinamarca**: San Francisco. Vereda El Vino. Finca La Carbonera. Alt. 2,800 m. 24 July 1990. *E. Linares, R. Sanchez & C. Escallon 3161* (COL!, UGDA-DLSz!—drawing); Mpio. Bojaca. Vereda de San Antonio. La Merced en faja de robledales proximo a la carretera que conduce de Mosquera a La Mesa. Alt. 2,600–2,700 m. July 1964. *J. Torres & G. Lozano 96* (COL!—sterile); Mun. Facatativá. Hacienda Cuatro Esquinas. Alt. 2,900 m. 21 July 1948. *M. Schneider 52* (AMES!). **Putumayo**: Cordillera Portachuelo, along the main road from Pasto to Mocoa, between Santiago and Pepino, on rocky cliffs. Alt. 1,900–2,400 m. 2 August 1961. *L.A. Garay, C.E. McClennan & A. Kapuler 468* (AMES!, UGDA-DLSz!—drawing). **Tolima**: Entre Fresno y paramo de Letras. Alt. 2,990 m. 26 April 1961. *H. Schmidt-Mumm 56* (COL!). ECUADOR. **Carchi**: 4–8 km W of Caramelo, along road from Tulcan to Alegria via Caramelo and Santa Barbara. Alt. 2,900 m. 5 February 1982. *C. Dodson & A. Gentry 12081* (MO!); Carretera Julio Andrade-El Carmen, Km 18, about 0°38′N 77°40′W. Bosque húmedo nublado. Alt. 3,200 m. 16 May 1982. *H. Balslev, L. Luteyn, B. Boom 2544* (AMES! *ex* QCA, UGDA-DLSz!—fragment, photo, drawing).

***Cyrtochilum englerianum*** (Kraenzl.) Kraenzl., Notizbl. Bot. Gart. Berlin-Dahlem 7: 95. 1917. ≡ *Oncidium englerianum* Kraenzl., Beibl. Bot. Jahrb. Syst. 117: 32. 1916. TYPE: Colombia. *Lehmann 6005* (holotype, K!; isotype, AMES!, UGDA-DLSz!—drawing).

**Figure 28:**
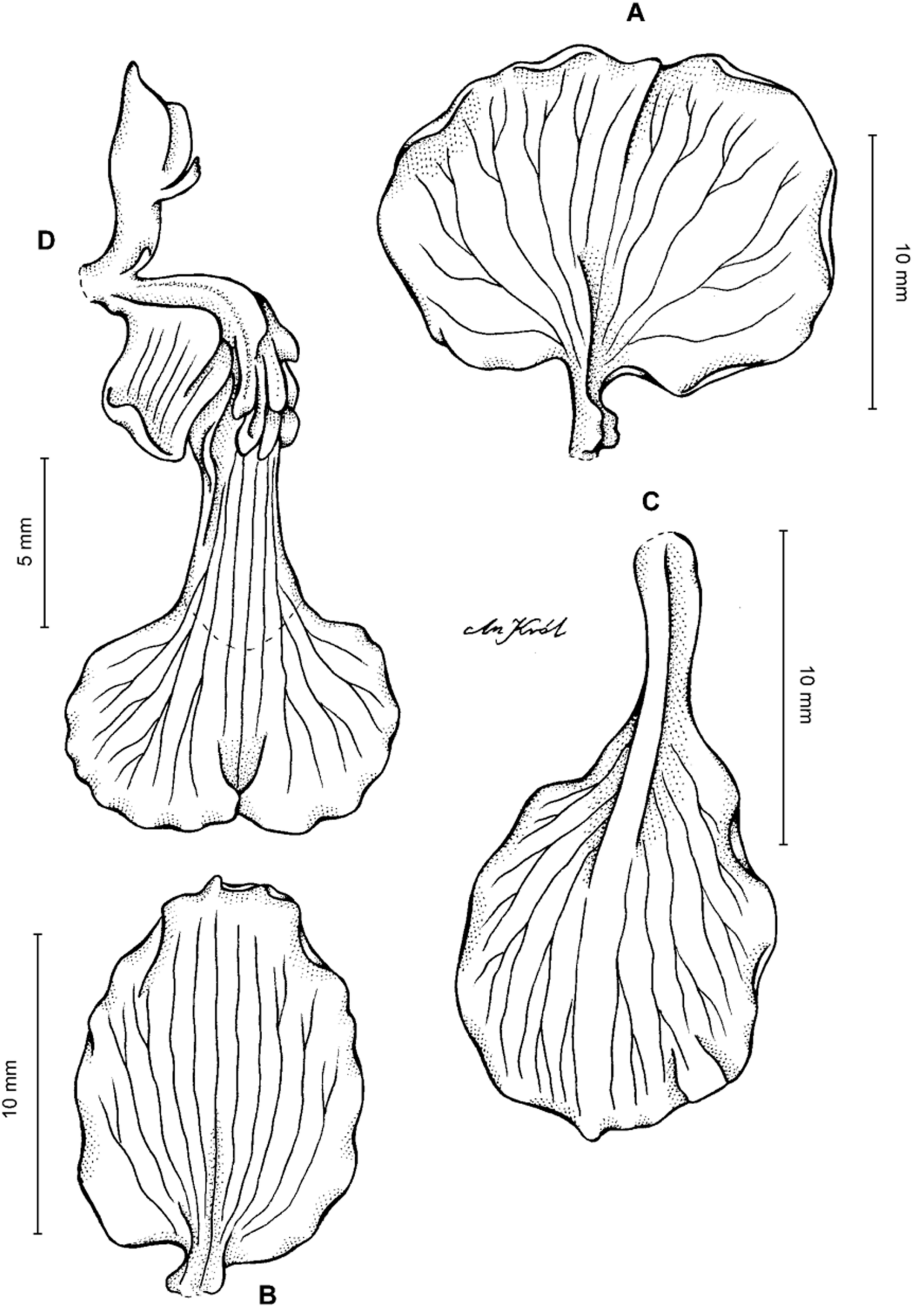
*Cyrtochilum englerianum* (Kraenzl.) Kraenzl. (A) Dorsal sepal; (B) Petal; (C) Lateral sepal; (D) Lip and gynostemium. Drawn by Anna Król from *Ewan 15751* (US).

**Figure 29:**
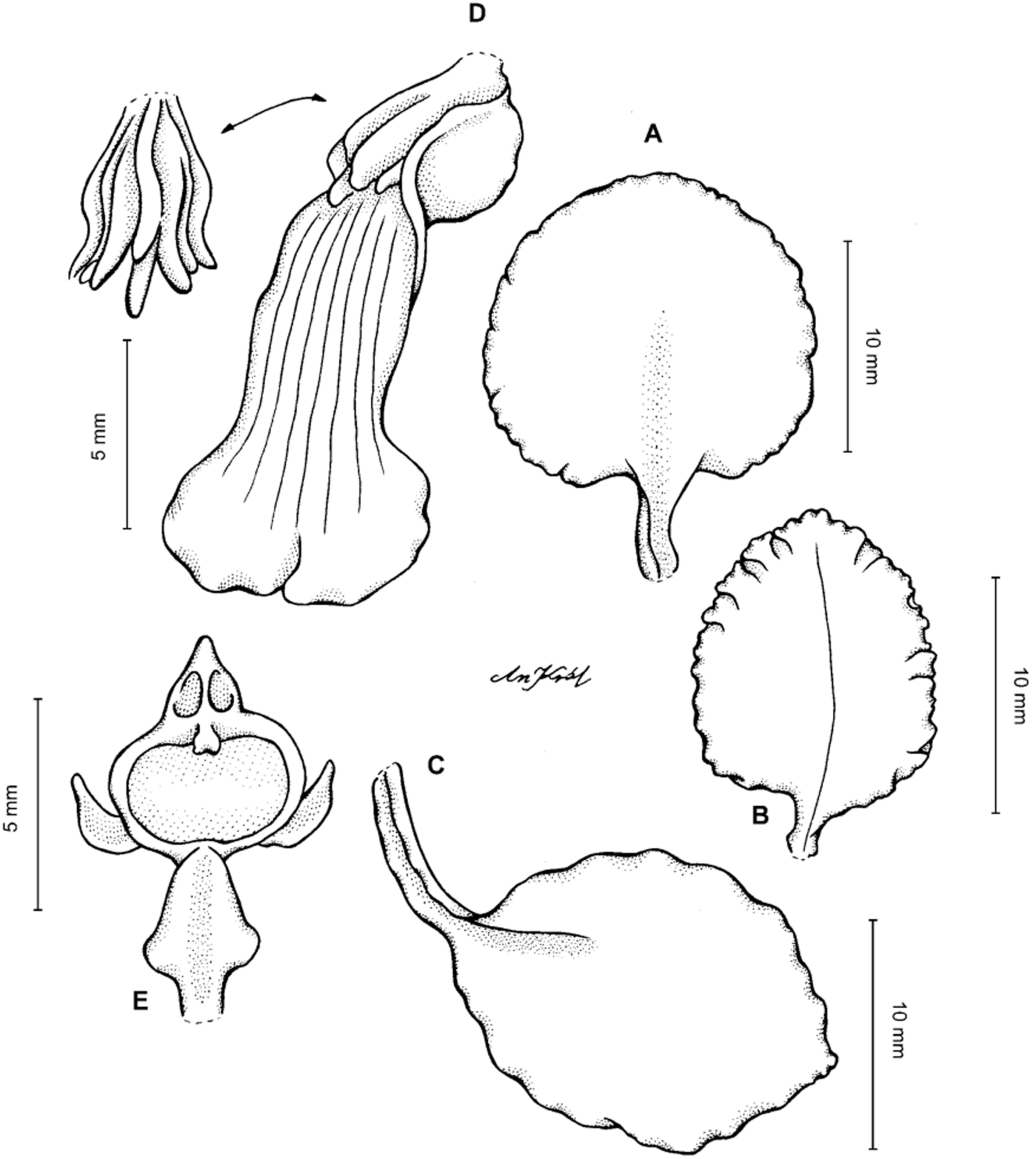
*Cyrtochilum englerianum* (Kraenzl.) Kraenzl. (A) Dorsal sepal; (B) Petal; (C) Lateral sepal; (D) Lip; (E) Gynostemium. Drawn by Anna Król from *Lehmann 6005* (AMES).

Pseudobulbs about 5 cm long, 1.5 cm in diameter, oblong-ovoid to almost cylindric, subtended by several sheaths, the uppermost foliaceous, bifoliate. Leaves about 30–40 cm long, up to 3 cm wide, oblanceolate, acute. Inflorescence about 75 cm long, branching, branches 3–7-flowered. Flowers brown with yellow apices, lip brown up to the apical part, callus yellow. Floral bracts 13–20 mm long, ovate, cucullate. Ovary with pedicel up to 30 mm long. Dorsal sepal clawed; claw about 3 mm long, wingless; blade 12–14 mm long, about 11–14 mm wide, suborbicular to transversely elliptic, obtuse to truncate, base subcordate, margins distantly undulate. Petals clawed; claw about 2 mm long, wingless; blade 12–13 mm long, 8–10.5 mm wide, ovate, acute, margins distantly undulate. Lateral sepals clawed; claw about 6 mm long, wingless; blade 15–18 mm long, 8–10 mm wide, ovate to obovate, acute, margins distantly undulate. Lip up to 15 mm long in total, pandurate in outline, base truncate; basal part up to 4 mm long, about 8 mm wide, semicircular to rectangular; callus composed of numerous digitate projections; apical part up to 10 mm long, ligulate in the lower half, expanded above into a 5 mm long and 10 mm wide transversally elliptic plate, apex emarginated, margins non-undulate. Gynostemium about 6 mm long, with a pair of oblanceolate, upcurved appendages (Figs. 28 and 29).

*Ecology*: No data.

*Distribution*: Colombia. Alt. 2,000–2,740 m.

*Notes*: cf. *C. diceratum*. ?? please explain.

*Representative specimens*: COLOMBIA. **Antioquia**: Alto Robletita above Sonson, via Sonson-Rio Sirgua camino, 2,740 m, 26–28 May 1944, *J. Ewan 15751* (US!, UGDA-DLSz!—drawing). **Cauca**: Andes of Popayán, 2,500 m, *F.C. Lehmann 6005* (AMES!, K!, UGDA-DLSz!—drawing). [**Nariño**]. Zwishen Pasto und Barbacoas, June 1922, 2,000 m, *W. Hopp 202* (Schlechter, 1917).

**(3) *Tetracopis-*group**

Basal lip part rhombic or deltoid, apical part ligulate, callus with large median keel. Dorsal sepal with cordate base, lamina wider than long. Gynostemium only basally connate with the lip, than abruptly upcurved.

This group embraces about 13 species. Most of them were described from Colombia, with only few species known in Ecuador and Venezuela.

### Key to the species

1 Gynostemium strongly sigmoid, lateral appendages digitate, rudimentary*C. xanthodon*
1* Not above combination of characters2
2 Lip covered with series of irregular outgrows running up to the lip apex*C. stegasaurum*
2* Not as above3
3 Lip callus consisting of a prominent, median ridge, papillate on the upper surface expanding apically into flabellate, dissected plate*C. ustulatum*
3* Lip callus does not form flabellate, dissected plate4
4 Dorsal sepal basally deeply cordate, basal auricles overlapping*C. klabochii*
4* Dorsal sepal with truncate or cordate base but not overlapping basally5
5 Petals cohering apically6
5* Petals not touching each other8
6 Lip callus consisting of a narrow, simple central ridge flanked by two ridges longer than the middle one*C. baldeviamae*
6* Lip callus complicated7
7 Gynostemum wings on the base of the stigma small, obscure, subulate, lip callus occupying the basal half of the lip*C. pozoi*
7* Gynostemium wings digitate, lip callus occupying the basal third of the lip length*C. steyermarkii*
8 Lip callus with a prominent, median ridge much elevated at the apex, which is rhombic-rectangular while observed from the side, much exceeding the other callus segments*C. garayi*
8* Lip callus not as above9
9 Lip callus glabrous*C. grandiflorum*
9* Lip callus papillate or ciliolate on the upper surface10
10 Gynostemium appendages linear, narrow*C. aemulum*
10* Gynostemium lateral appendages oblanceolate11
11 Basal lip part about twice longer than apical one*C. tetracopis*
11* Basal lip part about as long the apical one*C. monachicum*

***Cyrtochilum tetracopis*** (Rchb.f.) Kraenzl., Notizbl. Bot. Gart. Berlin-Dahlem 7: 93. 1917. ≡ *Oncidium tetracopis* Rchb.f., Gard. Chron. 1873: 915. 1873. TYPE: Colombia [New Grenada] *Bull 234* (holotype, W-R! 48799, UGDA-DLSz!—drawing).

= *Cyrtochilum ludens* (Rchb. f.) Kraenzl., Notizbl. Bot. Gart. Berlin-Dahlem 7: 93. 1917. ≡ *Oncidium ludens* Rchb. f., Gard. Chron. 23: 756. 1885. TYPE: *Veitch cult*. (holotype, W-R! 16854, UGDA-DLSz!—drawing).

**Figure 30:**
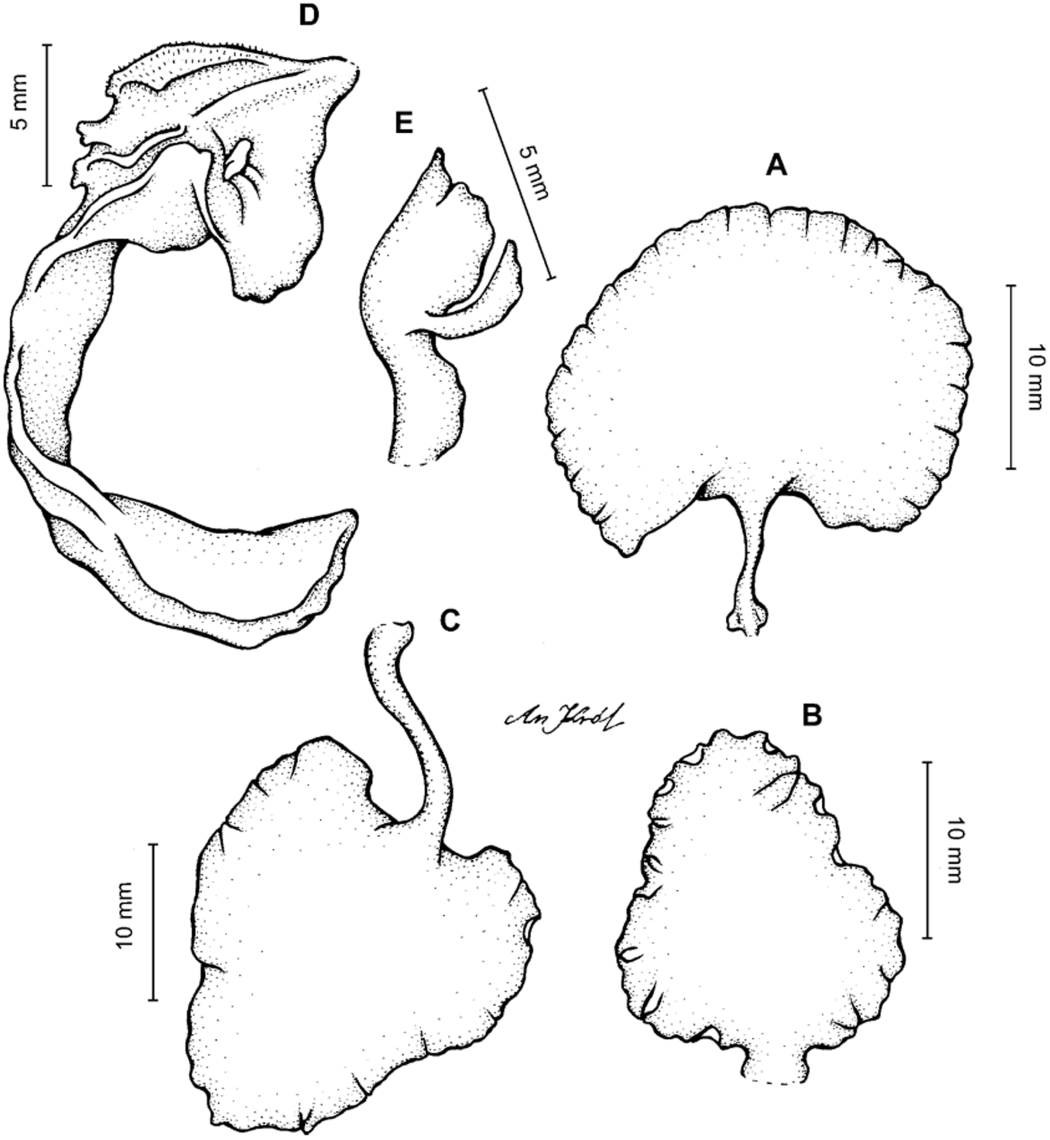
*Cyrtochilum tetracopis* (Rchb.f.) Kraenzl. (A) Dorsal sepal; (B) Petal; (C) Lateral sepal; (D) Lip; (E) Gynostemium, side view. Drawn by Anna Król from *Bull 234* (W-R).

**Figure 31:**
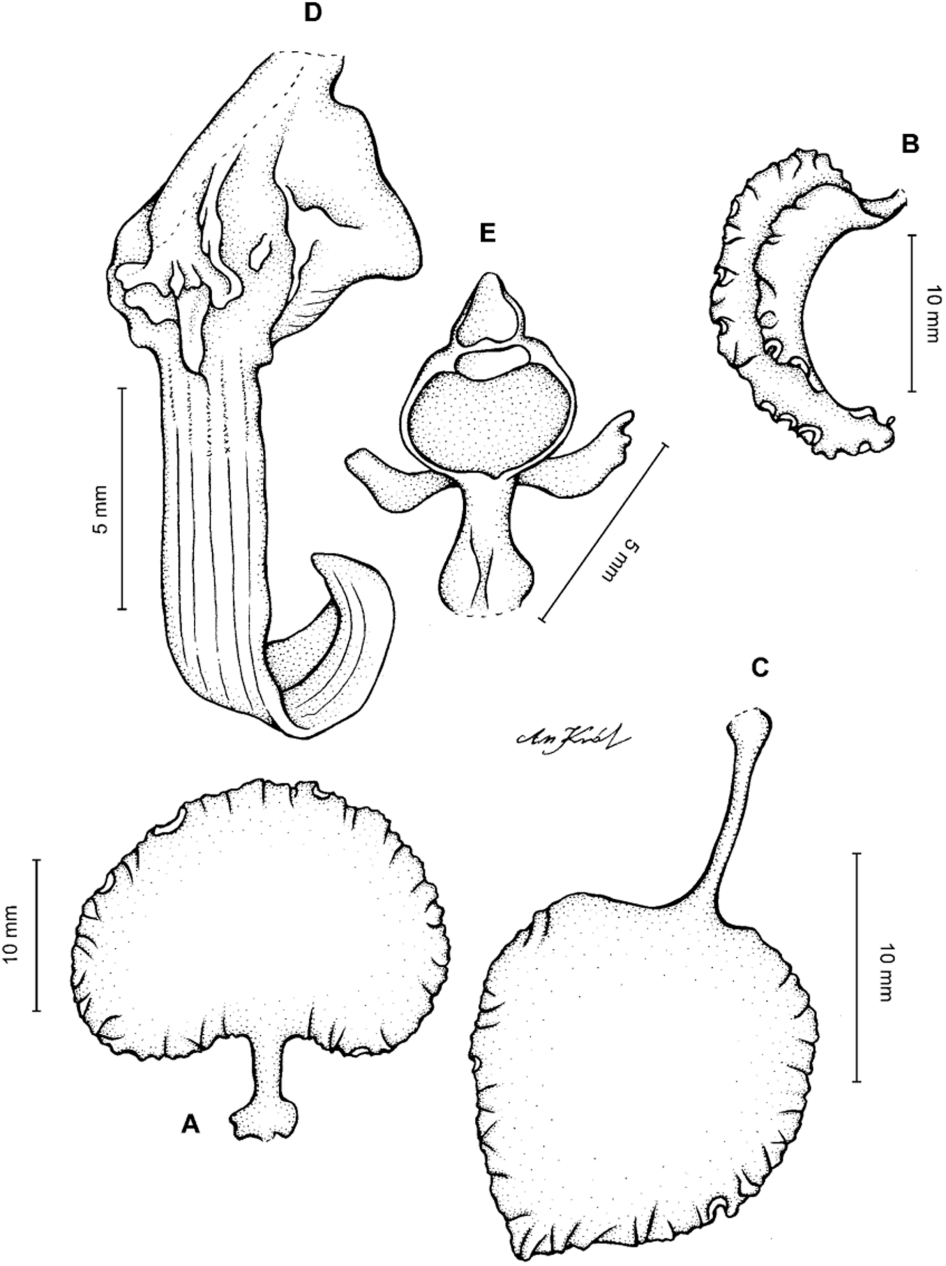
*Cyrtochilum tetracopis* (Rchb.f.) Kraenzl. (A) Dorsal sepal; (B) Petal; (C) Lateral sepal; (D) Lip; (E) Gynostemium, front view. Drawn by Anna Król from *Veitch s.n*. (W-R)—type of *Cyrtochilum ludens* (Rchb. f.) Kraenzl.

Rhizome repent. Pseudobulbs approximate or fairy spaced, up to 11 cm long, 5 cm wide, slightly compressed, bifoliate with several sheathing leaves. Leaves up to 65 cm long, 4.5 cm wide, oblong-elliptic to oblong-oblanceolate, acute. Inflorescence suberect to subpendent; peduncle up to 100 cm long, panicle up to 150 cm long with branches up to 50 cm long. Flowers medium-sized, conspicuous. Floral bracts 20 mm long. Pedicel and ovary 27 mm long. Dorsal sepal clawed; claw 6–7 mm long, narrow, canaliculate, with prominent rhombic wings just above the base; blade 18–20 mm long, 22–23 mm wide, reniform, margins entire, more or less undulate, base cordate, apex rounded. Petals clawed; claw about 2 mm long, wide; blade 18–20 mm long, 12–18 mm wide when spread, obliquely triangular-ovate, strongly falcate, base truncate, apex obtuse, margins strongly undulate. Lateral sepals prominently clawed; claw 10–14 mm long, narrow, with rather obscure wing on the outer margin; blade 20–27 mm long, 18–23 mm wide, obliquely cordate-ovate in general outline, apex obtuse, margins entire, slightly undulate. Lip 16–19 mm long in total, gently curved in natural position; basal part 6–7 mm long, 10–11 mm wide, rhombic in general outline, base broadly cuneate; callus consisting of a prominent, median ridge, ciliolate on the upper surface with front margins variously dissected, with an additional thickenings beyond the main callus; apical part 10–13 mm long, 3–5 mm wide, ligulate, apex obtuse. Gynostemium 8–10 mm long, gently sigmoid, connate basally with the lip, lateral appendages oblanceolate, upcurved, reaching the anther base (Figs. 30 and 31).

*Ecology*: Terrestrial, rarely lithophytic.

*Distribution*: Colombia, Venezuela (Dunsterville & Garay, 1979). Alt. 1,800–2,400 m.

*Notes*: There are four species of this group having papillate or ciliolate lip callus, i.e. *C. ustulatum*, *C. aemulum*, *C. monachicum* and *C. tetracopis*. From the aforementioned species, *C. ustulatum* is distinguished by the unique lip callus which forms a kind of flabellate, dissected plate. The gynostemium of *C. aemulum* is adorned with a pair of relatively short, linear appendages (vs appendages oblanceolate, reaching anther base). *Cyrtochilum tetracopis* appears to be similar to *C. monachicum*, but both species can be distinguished by the lip details—the basal lip part is about twice shorter than apical one in *C. tetracopis*, and similar in length in *C. monachicum*. Further study should reveal if they really deserve a status of separate taxa.

*Representative specimens*: COLOMBIA. **Cundinamarca**: Waldem uber Pacho, 1,800–2,400 m, March 1892, *F.C. Lehmann 7299* (AMES!, UGDA-DLSz!—fragment, photo, drawing). [New Grenada], *W. Bull 234* (W-R!, UGDA-DLSz!—drawing).

***Cyrtochilum monachicum*** (Rchb.f.) Kraenzl., Notizbl. Bot. Gart. Berlin-Dahlem 7: 93. 1917. ≡ *Oncidium monachicum* Rchb.f., Gard. Chron., n.s., 19: 368. 1883. TYPE: [Colombia]. New Grenada. Cult. *Williams s.n*. (holotype, W-R! 9415, UGDA-DLSz!—drawing).

**Figure 32:**
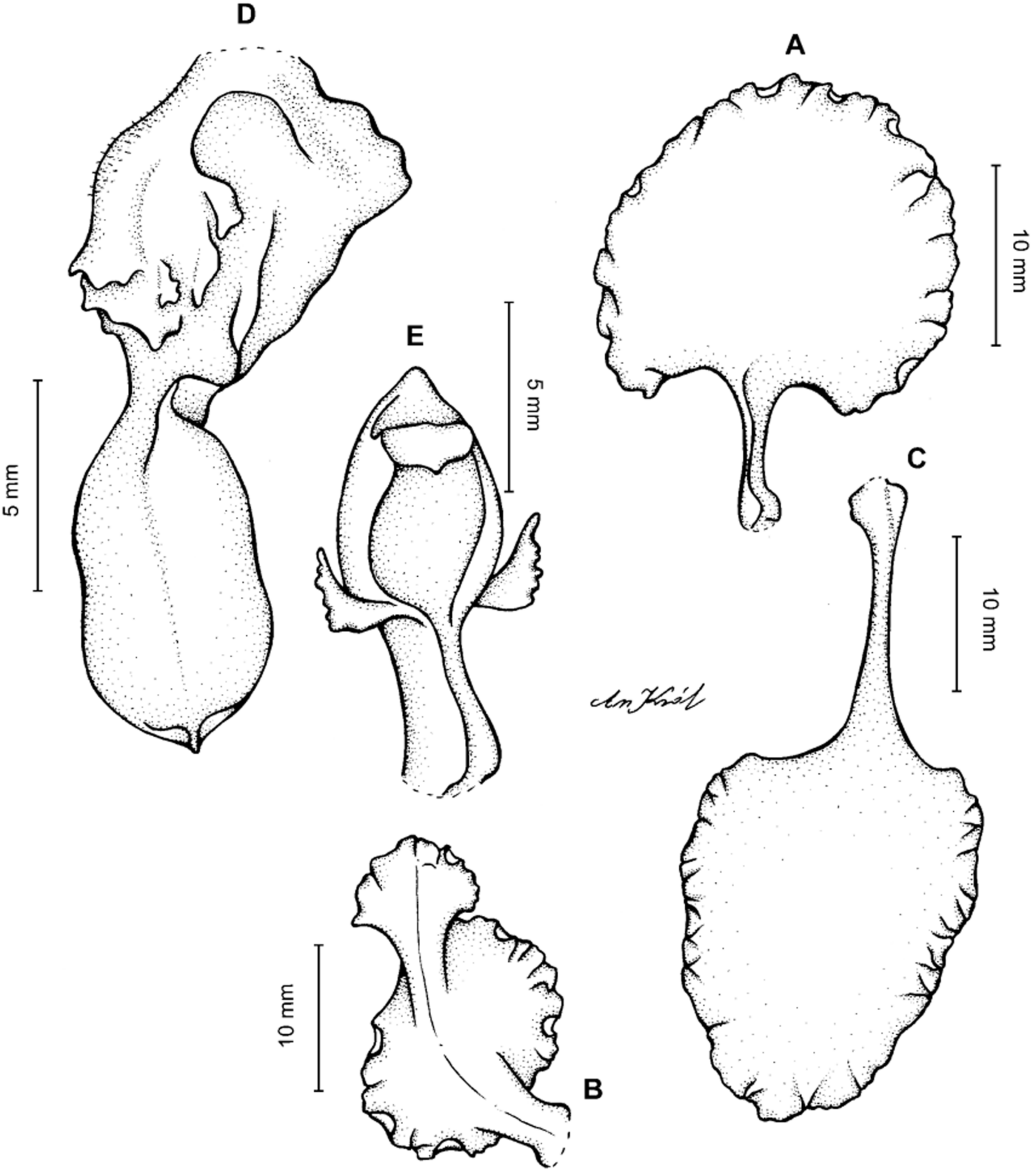
*Cyrtochilum monachicum* (Rchb.f.) Kraenzl. (A) Dorsal sepal; (B) Petal; (C) Lateral sepal; (D) Lip; (E) Gynostemium. Drawn by Anna Król from *Williams s.n*. (W-R).

Pseudobulbs enclothed basally with several distichous, foliaceous sheaths, bifoliate. Inflorescence up to 3 m long, flexuose. Flowers large, conspicuous, showy, purple-red with white margins on tepals. Floral bracts 18 mm long. Pedicel and ovary 42 mm long. Dorsal sepal clawed; claw 6 mm long, narrow, canaliculate, with triangular-ovate wings just above the base; blade 18 mm long, 22 mm wide, reniform, base subcordate, margins entire, undulate, apex rounded. Petals clawed; claw about 3 mm long, wide; blade 20–21 mm long, 15 mm wide when spread, oblique, elliptic-ovate, strongly falcate, recurved, base truncate, obtuse at the apex, margins strongly undulate. Lateral sepals prominently clawed; claw 17–20 mm long, narrow, with basal obscure wings; blade 22–25 mm long, 22 mm wide, obliquely elliptic-ovate in general outline, basally cuneate-subcordate, apex obtuse, margins slightly undulate. Lip 18 mm long in total, curved in natural position just above the base, somewhat constricted near the middle; basal part 9 mm long, about 10 mm wide, broadly deltoid, convex in the centre; callus large, consisting of a flat, papillate, pad-like tissue in the base irregularly dissected along apical margin, with some additional projections on each side; apical part 9 mm long, about 5 mm wide, ligulate-elliptic, rounded at the apex. Gynostemium 11–12 mm long, subarcuate, connate basally with the lip, lateral appendages obliquely oblanceolate, lower margins irregularly dentate, not reaching the anther base (Fig. 32).

*Ecology*: Epiphytic in montane cloud forest.

*Distribution*: Colombia, Ecuador (Dodson & Dodson, 1980). Alt. 1,500–2,230 m.

*Notes*: Dalström (2001) considered this species conspecific with *C. annulare*, but the lip base in this species is oblong-elliptic (vs broadly deltoid) and its callus consists of a narrow central ridge flanked by two ridges with dissected margins, papillate on the upper surface (vs pad-like tissue in the base irregularly dissected along apical margin, with some additional projections on each side). In our opinion *C. monachicum* is similar to *C. tetracopis* from which it differs in the details of the lip morphology (see note provided under *C. tetracopis*).

*Representative specimen*: COLOMBIA. New Grenada. Cult. *B.S. Williams s.n*. (W-R! 9415).

***Cyrtochilum klabochii*** Szlach. & Kolan., ***sp. nov***. TYPE: Colombia. *Klaboch s.n*. (holotype, W-R! 15128; UGDA-DLSz!—drawing).

Diagnosis: The *species is distinguished by the orbicular dorsal sepal with deeply cordate base, and overlapping basal auricles, not found in any other representative of Cyrtochilum. Other characteristic traits of the new species are elliptic-orbicular lateral sepals with cordate base. This species resembles somewhat C. baldeviamae from which it differs also in almost rhombic basal lip part and callus formed of narrow central ridge, variously lobed in the apical part, flanked by two ridges apically dissected into few parts*.

**Figure 33:**
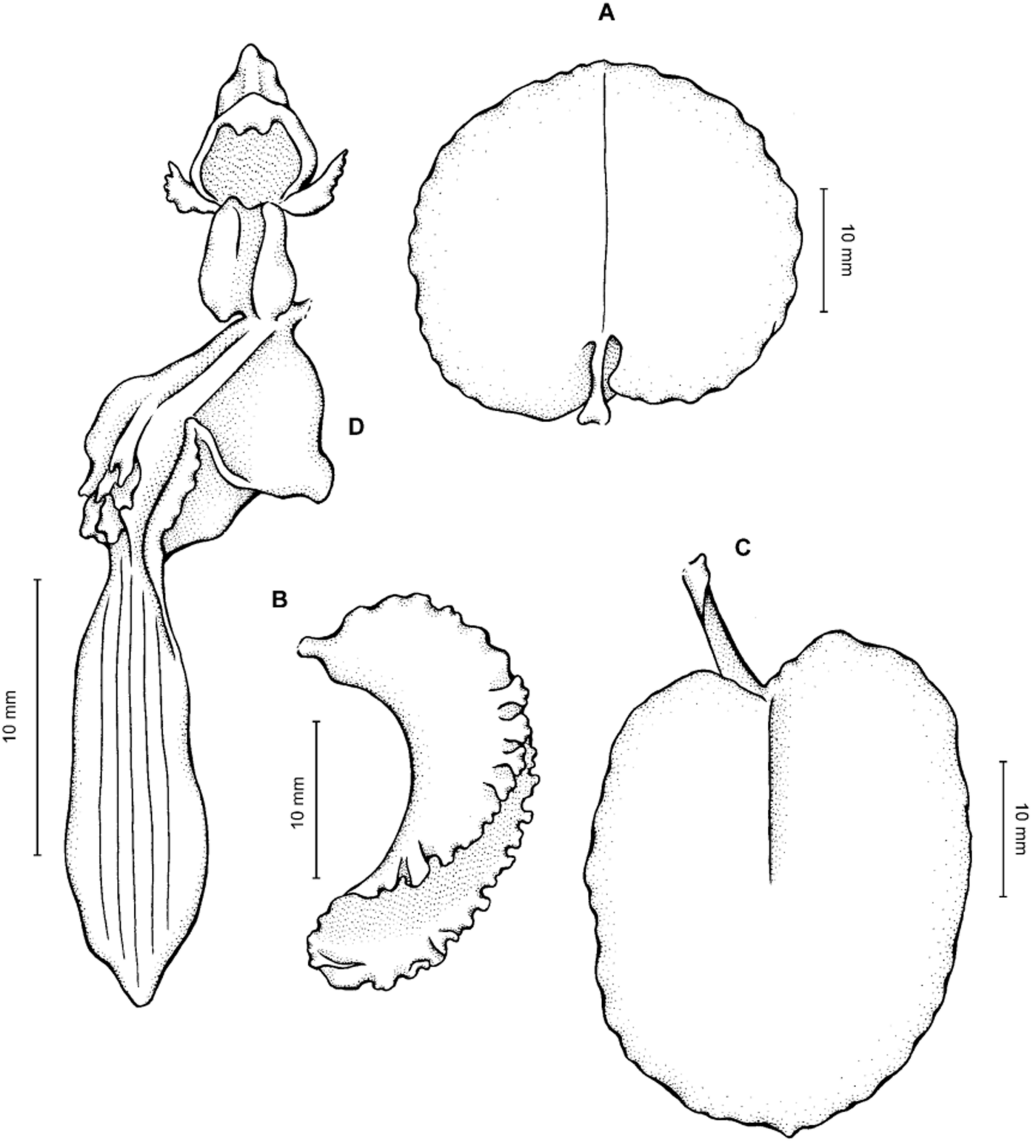
*Cyrtochilum klabochii* Szlach. & Kolan. (A) Dorsal sepal; (B) Petal; (C) Lateral sepal; (D) Gynostemium and lip, side view. Drawn by Anna Król from *Klaboch s.n*. (W-R).

Vegetative parts unknown. Dorsal sepal shortly clawed; claw 5 mm long, narrow, canaliculate, with obscure elliptic-rhombic wings just above the base; blade 30 mm long and wide, orbicular, base deeply cordate, basal auricles overlapping, margins somewhat crenate, irregularly and distantly undulate, apex rounded. Petals clawed; claw about 3 mm long, basally somewhat winged; blade 25 mm long, 17 mm wide when spread, obliquely elliptic, obtuse at the apex, margins strongly and irregularly undulate, free from one another. Lateral sepals prominently clawed; claw 12 mm long, narrow, with basal wing on the outer margin; blade 43 mm long, 28 mm wide, obliquely elliptic-orbicular in general outline, basally cordate, obtuse at the apex, margins very slightly undulate. Lip 24 mm long in total, curved in natural position exposing massive callus; basal part 7 mm long, 14 mm wide, almost rhombic, base broadly cuneate, convex along the centre, callus complicated, glabrous, consisting of narrow central ridge, variously lobed in the apical part, flanked by two ridges apically dissected into few parts; apical part 17 mm long, about 5 mm wide, ligulate, attenuate towards apex. Gynostemium 9 mm long, subarcuate, connate basally with the lip, lateral appendages oblique, oblanceolate, upcurved, margins denticulate, not reaching the anther base (Fig. 33).

*Etymology*: In honour of the collector of the type specimen.

*Ecology*: No data.

*Distribution*: Colombia.

*Notes*: The unique character of this entity is orbicular dorsal sepal with deeply cordate base and overlapping basal auricles, not found elsewhere in the genus. The lateral sepals are also exceptional being elliptic-orbicular in general outline, with basally cordate base. Cyrtochilum *baldeviamae* differs from the new species in having broadly obovate basal lip part (vs almost rhombic) and callus consisting of a narrow, simple central ridge flanked by two ridges longer than the middle one (vs callus formed of narrow central ridge, variously lobed in the apical part, flanked by two ridges apically dissected into few parts).

*Representative specimen*: COLOMBIA. Reña, 1883, *E. Klaboch s.n*. (W-R!; UGDA-DLSz!—drawing).

*Conservation status:* This species is described based on material collected in 1883. We cannot assess the current threats for this taxon hereby the “data deficient” (DD) category should be applied according to IUCN (2001).

***Cyrtochilum aemulum*** (Rchb.f. & Warsz.) Kraenzl., Notizbl. Bot. Gart. Berlin-Dahlem 7: 93. 1917. ≡ *Oncidium aemulum* Rchb.f. & Warsz., Bonplandia (Hannover) 2: 102. 1854. TYPE: Colombia. *Warszewicz s.n*. (holotype, W-R! 48937; isotypes: AMES!, W-R! 48931 & W-R! 48941, UGDA-DLSz!—drawing).

= *Cyrtochilum luerorum* Dalström, Lankesteriana 17(2): 281–283. 2017. TYPE: Ecuador. Carchi. Above Maldonado along the road to Tulcan, terrestrial in dense shrubs close to the road, alt. about 2,500 m. Mar. 1992. *Dalström et al. 1563* (holotype, SEL—flower in alcohol).

**Figure 34:**
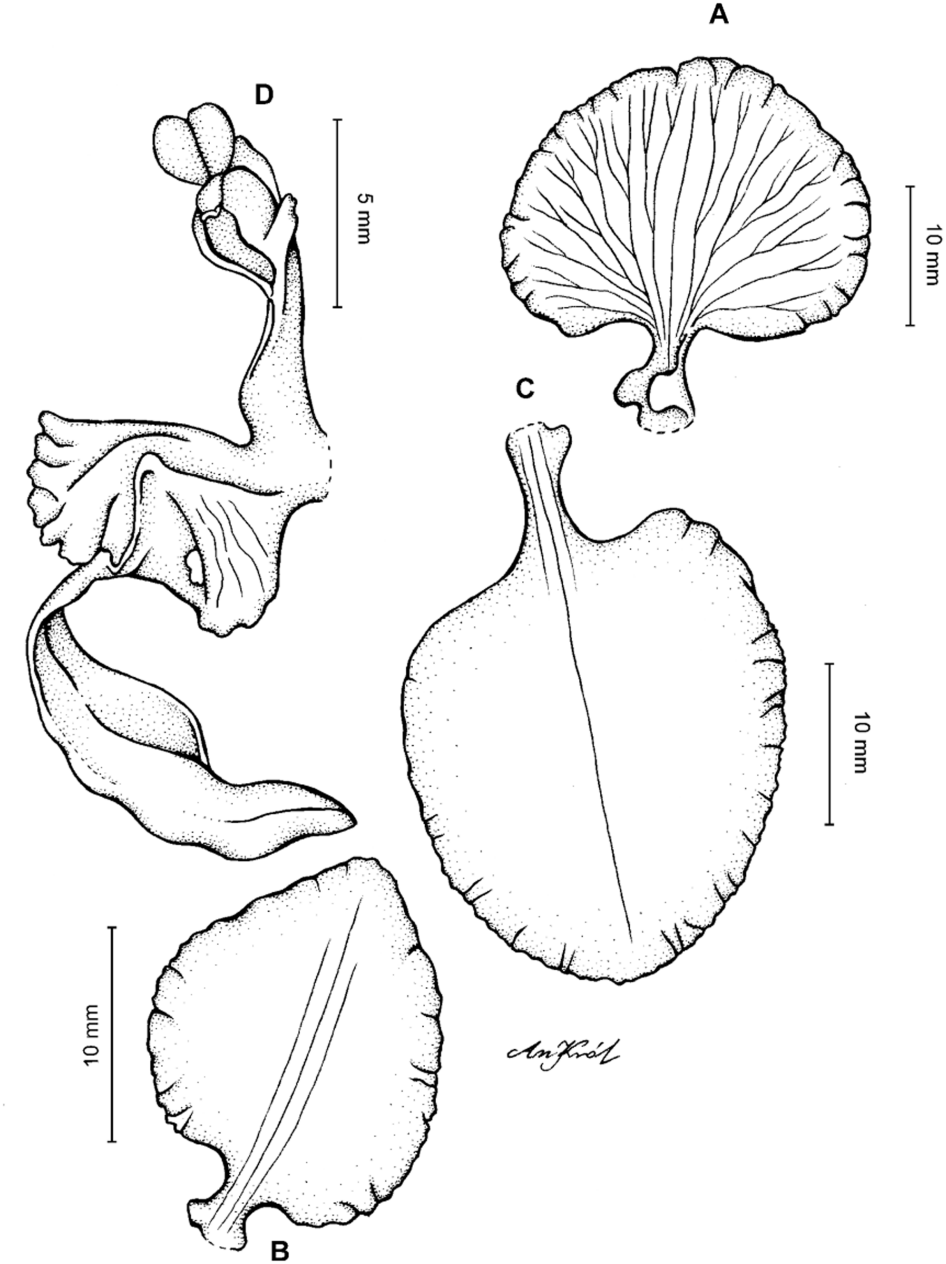
*Cyrtochilum aemulum* (Rchb.f. & Warsz.) Kraenzl. (A) Dorsal sepal; (B) Petal; (C) Lateral sepal; (D) Gynostemium and lip, side view. Drawn by Anna Król from *Warszewicz s.n*. (W-R).

**Figure 35:**
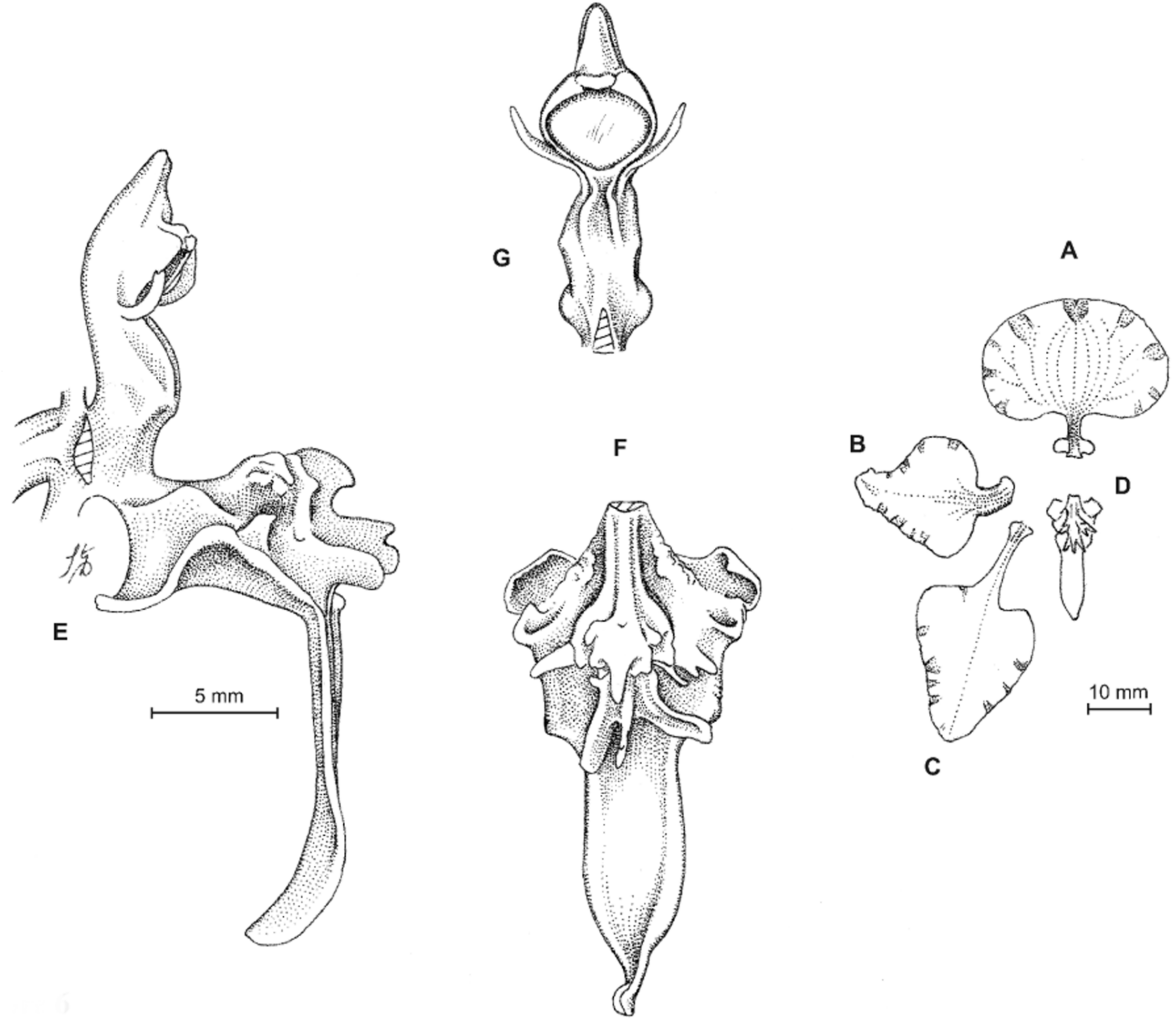
*Cyrtochilum aemulum* (Rchb.f. & Warsz.) Kraenzl. (A) Dorsal sepal; (B) Petal; (C) Lateral sepal; (D) Lip; (E) Gynostemium and lip, side view; (F) Lip, front view; (G) Gynostemium. Drawn by Stig Dalström from the holotype of *Cyrtochilum luerorum* Dalström, *Dalström et al. 1563* (SEL).

Rhizome elongate, creeping with remotely spaced pseudobulbs. Pseudobulbs fusiform-ovoid, elliptic in cross section, smooth, supported basally by few leafy bracts. Leaves linear-oblanceolate, acute. Inflorescence several meters long, paniculate, wiry, with widely spaced few-flowered branches. Flowers large, conspicuous, showy, sepals brown with white edge, petals basally yellow, then brown, apically white, spotted with red, lip reddish brown with white apex. Floral bracts 15–26 mm long. Pedicel and ovary 30–47 mm long. Dorsal sepal shortly clawed; claw 4–8 mm long, rather narrow, canaliculate, with prominent elliptic-rhombic wings just above the base; blade 18–23 mm long, 23–28 mm wide, reniform, base subcordate, margins somewhat crenate, irregularly undulate, apex rounded. Petals clawed; claw about 3–6 mm long, basally somewhat winged; blade 20–25 mm long in total, 14–20 mm wide when spread, obliquely cordate-ovate, obtuse at the apex, margins irregularly undulate. Lateral sepals prominently clawed; claw 8–12 mm long, narrow, with basal wing on the outer margin; blade 20–34 mm long and wide, obliquely elliptic-ovate in general outline, basally truncate, obtuse at the apex, margins very slightly undulate. Lip 15–16 mm long in total, curved in natural position exposing massive callus; basal part 6–8 mm long, 9–10 mm wide, almost rhombic, base truncate, convex along the centre, callus very complicated, more or less papillate on the upper surface, consisting of narrow central ridge, variously lobed in the apical part, flanked by two ridges with diverging apices, margins entire; apical part 8–13 mm long, about 4 mm wide, ligulate, attenuate towards apex. Gynostemium 9–12 mm long, subarcuate, connate basally with the lip, lateral appendages oblique, linear, upcurved, not reaching the anther base (Figs. 34 and 35).

*Ecology*: Terrestrial or epiphytic in cloud forest.

*Distribution*: Colombia, Ecuador. Alt. 2,500–2,800 m.

*Notes*: This showy species can be easily recognized by the narrow, linear and upcurved gynostemium lateral appendages—these characters separate *C. aemulum* from two other species having papillate lip callus, *C. tetracopis* and *C. monachicum*.

Examination of protologues of Dalström’s *C. luerorum* and Reichenbach’s & Warszewicz’s *Oncidium aemulum* led us to the conclusion that they are conspecific as we could not detect their distinctiveness. In fact, the petals of *C. luerorum* have longer claw, however, in our opinion the lip callus of both species is very similar. We did not find pubescence on the lip callus of the type specimen of *C. aemulum*.

*Representative specimens*: COLOMBIA. **Norte de Santander**: Ocaña, *J. Warszewicz s.n*. (W-R!; UGDA-DLSz!—drawing); Ocaña, *L. Schlim 413* (AMES!; UGDA-DLSz!—drawing). **Santander**: Epiphytic in cloud forest near Alto de San Francisco, above Villacaro, 2,800 m, 11 May 1984, *C. Luer, J. Luer, R. Escobar & E. Valencia 10264* (MO!, UGDA-DLSz!—drawing). ECUADOR. **Carchi**: Above Maldonado, ca. 2,500 m, *S. Dalström et al. 1563* (Dalström, 2010).

***Cyrtochilum grandiflorum*** (Rchb.f.) Kraenzl., Notizbl. Bot. Gart. Berlin-Dahlem 7: 94. 1917. ≡ *Oncidium grandiflorum* Rchb.f., Gard. Chron., n.s., 15: 782. 1881. TYPE: Colombia. *Schmidt s.n*. (holotype, W-R! 48967, UGDA-DLSz!—drawing).

**Figure 36:**
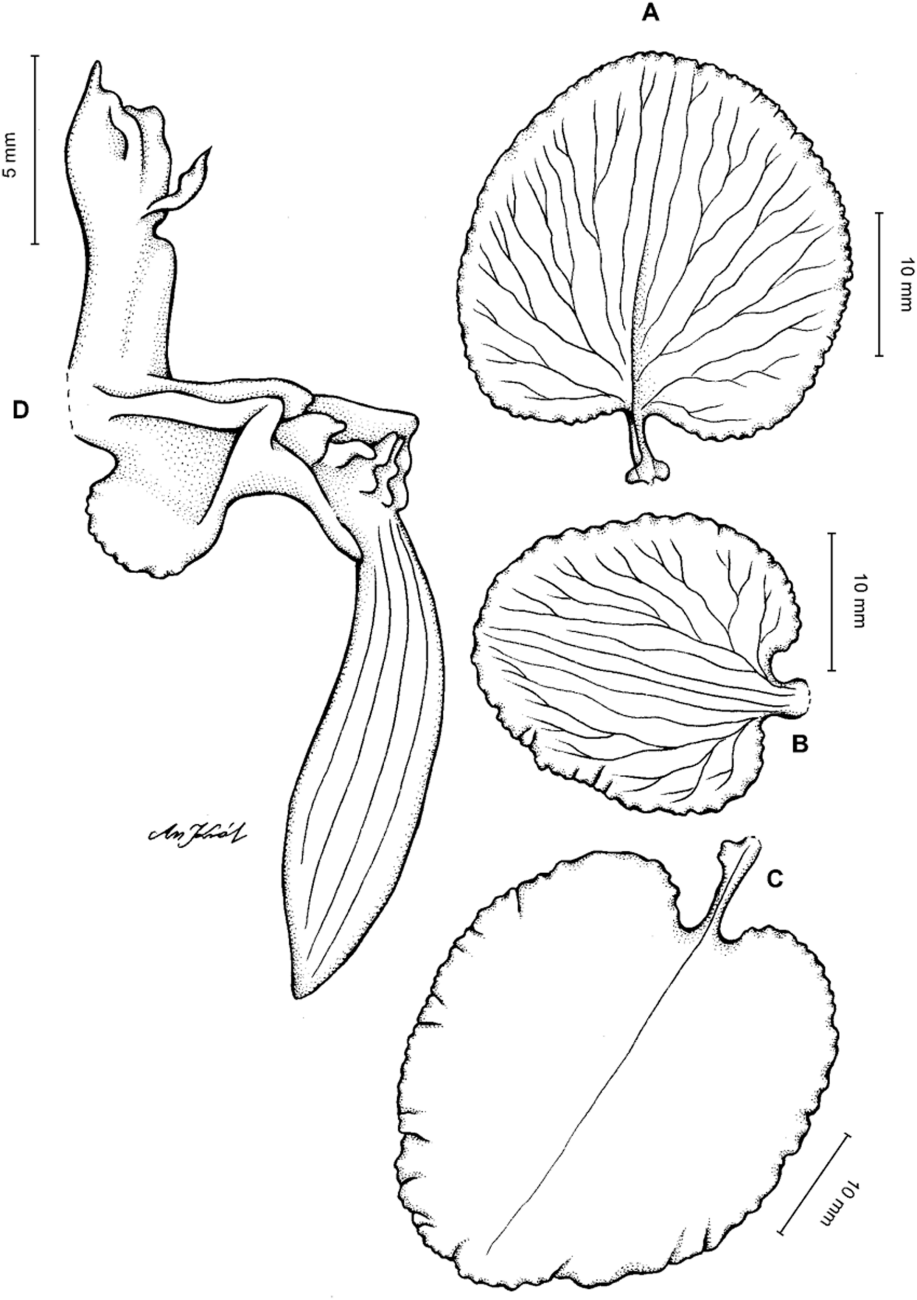
*Cyrtochilum grandiflorum* (Rchb.f.) Kraenzl. (A) Dorsal sepal; (B) Petal; (C) Lateral sepal; (D) Lip and gynostemium. Drawn by Anna Król from *Betancur & Asoc. Colomb. Bot. 12457* (COL).

**Figure 37:**
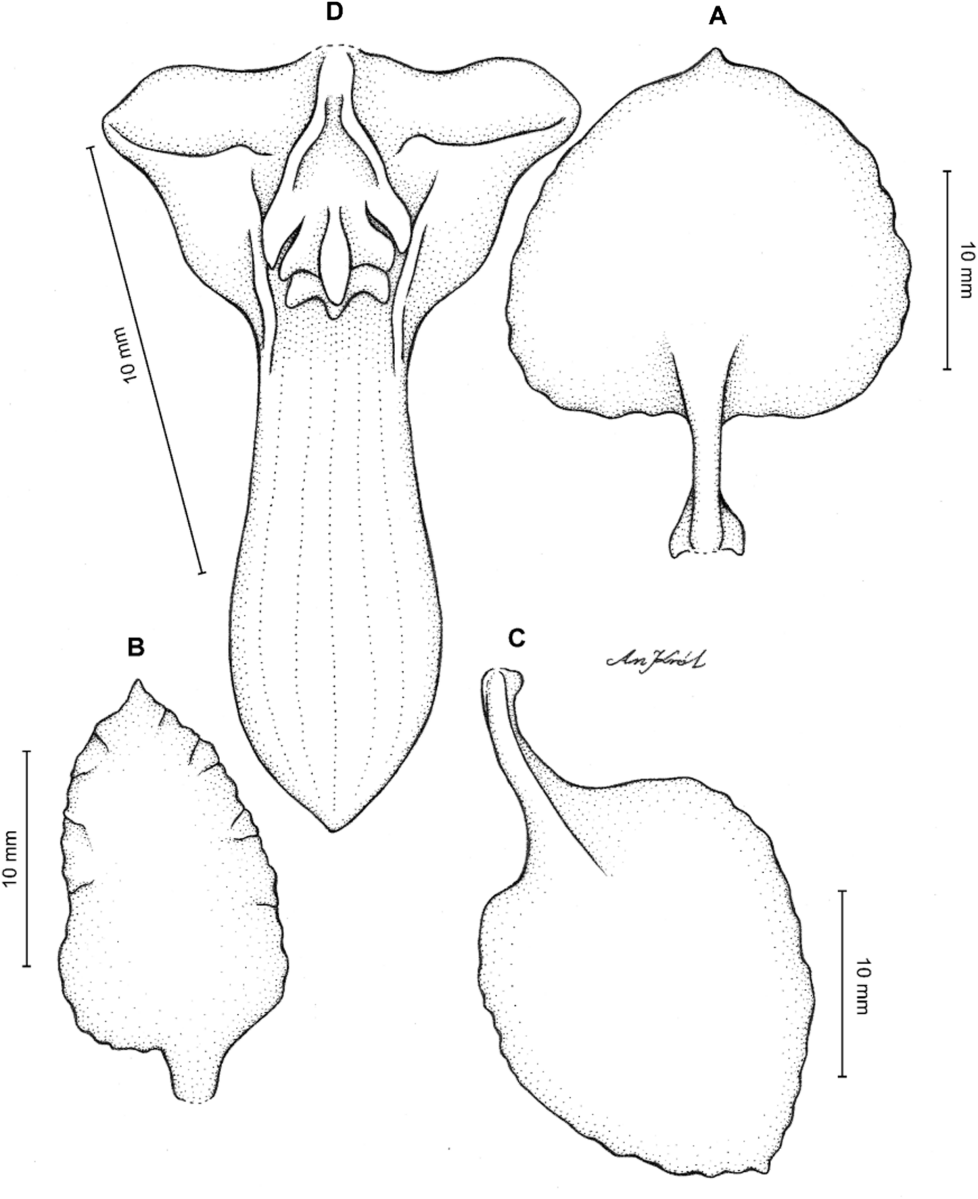
*Cyrtochilum grandiflorum* (Rchb.f.) Kraenzl. (A) Dorsal sepal; (B) Petal; (C) Lateral sepal; (D) Lip. Drawn by Anna Król from *Schmidt s.n*. (W-R).

Vegetative parts most probably similar to those of *C. steyermarkii*. Leaves up to 47 cm long and 6.5 cm wide, oblanceolate, acute. Flowers large, conspicuous, showy. Floral bracts 15 mm long. Pedicel and ovary about 42 mm long. Dorsal sepal clawed; claw 5–7 mm long, rather narrow, canaliculate, with prominent ovate-triangular wings just above the base; blade 17–30 mm long, 20–30 mm wide, reniform-suborbicular, base truncate, margins entire, apex shortly cuspidate to obtuse. Petals clawed; claw about 3 mm long; blade 17–24 mm long in total, 12–24 mm wide when spread, obliquely elliptic to elliptic-ovate, cuspidate at the apex, margins somewhat undulate. Lateral sepals prominently clawed; claw about 8 mm long, narrow, with basal wing on the outer margin; blade 21–34 mm long, 19–32 mm wide, obliquely ovate or elliptic in general outline, basally rounded to cordate, apex acute to obtuse, margins entire. Lip 18–23 mm long in total, curved in natural position exposing massive callus; basal part 6 mm long, about 9–10 mm wide, triangular-rhombic, base truncate, convex along the centre, callus relatively simple, consisting of central fleshy pad, divided apically into 6 lobules and flanked on each side by additional lobe; apical part 12–15 mm long, 4–6 mm wide, ligulate-oblanceolate, somewhat extended towards apex and than abruptly attenuate, subacute. Gynostemium 9 mm long, subarcuate, connate basally with the lip, lateral appendages oblique, oblong, not reaching the anther base (Figs. 36 and 37).

*Ecologyi*: Epiphyte.

*Distribution*: Colombia, Venezuela (Hokche, Berry & Huber, 2008). Alt. 2,440–2,600 m.

*Notes*: This species appears to be similar to *C. steyermarkii* and *C. baldeviamae*. It differs from the former by having much simpler callus structure and from the latter by free petals, not touching each others. From the other species of this group having truncate or cordate base of dorsal sepal but not overlapping basally and petals not cohering apically, as *C. aemulum*, *C. tetracopis* and *C. monachicum* this is the only one with glabrous lip callus. Cyrtochilum *garayi* is distinct in the lip callus which consists of a prominent, median ridge much elevated at the apex with small basal digitate outgrowth, papillate on the upper surface, flanked by shorter ridges, divided into 3 digitate projections.

*Representative specimens*: COLOMBIA. [**Cundinamarca**] : Bogotá, October 1882, *Schmidt s.n*. (W-R! 48967; UGDA-DLSz!—drawing). **Norte de Santander**: Mpio. Toledo. Carretera Chinacota-Toledo, alto de Mejue, 7°28′N 72°33′W, 2,440–2,600 m, 13 November 2006, *J. Betancur & Asoc. Colomb. Bot. 12457* (COL!, UGDA-DLSz!—drawing).

***Cyrtochilum steyermarkii*** (Foldats) Kolan. & Szlach., ***comb. nov*.**

Basionym: *Oncidium steyermarkii* Foldats, Acta Bot. Venez. 3: 366. 1968. TYPE: Venezuela. *Steyermark & Rabe 96993* (holotype, VEN).

**Figure 38:**
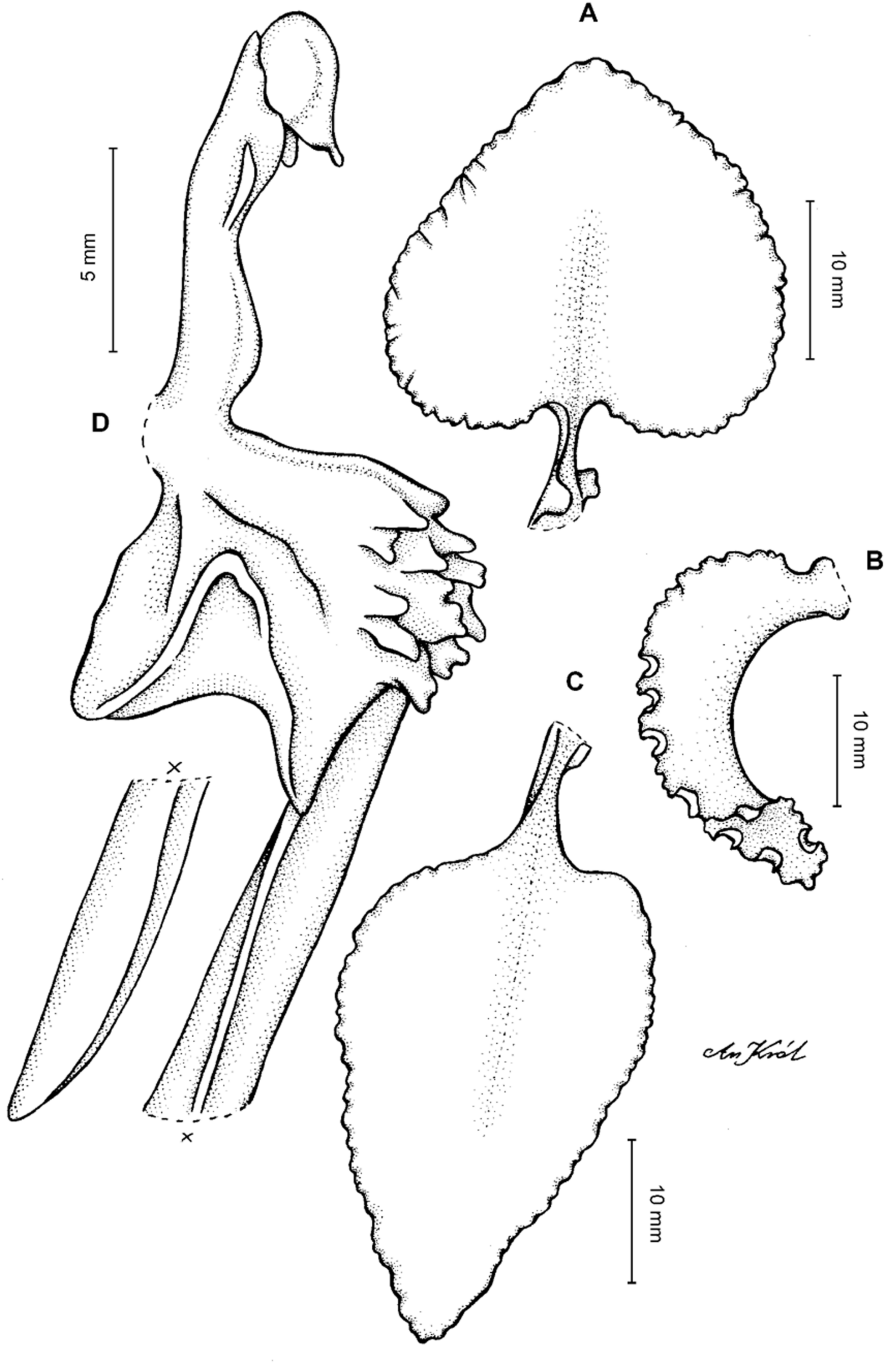
*Cyrtochilum steyermarkii* (Foldats) Kolan. & Szlach. (A) Dorsal sepal; (B) Petal; (C) Lateral sepal; (D) Lip and gynostemium, side view. Drawn by Anna Król from *Fendler 1376* (AMES).

Rhizome creeping. Pseudobulbs 5–12 cm long, 2.5–5 cm wide, narrowly ovoid, flattened, 1–3-leaved, laterally enclothed with various distichous, imbricating sheaths, 3 internal ones foliaceous. Leaves up to 63 cm long, 2.2–4.5 cm wide, oblong to oblong-lanceolate, attenuated in the base. Inflorescence over 200 cm long, paniculate, branches up to 50 cm long. Flowers with dark brown sepals, petals yellow with purple spots, lip brick-coloured with yellow callus and brown spots. Floral bracts 12–25 mm long, ovate. Pedicellate ovary 17–40 mm long. Dorsal sepal clawed; claw up to 7 mm long, narrow, with a pair of small appendages; blade 24–28 mm long, 19–23 mm wide, suborbicular to suborbicular-ovate, apex obtuse to emarginate, margins erose, undulate. Petals shortly clawed; claw wingless; blade 22–27 mm long, 13–14 mm wide, ovate, acute, margins erose. Lateral sepals clawed; claw up to 11 mm long, narrow, with a pair of small appendages; blade 29–34 mm long, 21–22 mm wide, broadly ovate to orbicular-ovate, apex obtuse, margins erose. Lip obscurely 3-lobed, 20–24 mm long in total, 8.4–15 mm wide, lateral lobes subquadrate, recurved in natural position; callus longitudinally tricarinate, multituberculate in apical part and here with two short keels; apical part up to 18 mm long and 4 mm wide, oblong-lanceolate, acute, reflexed in natural position. Gynostemium 8–10 mm long, lateral appendages oblong, upcurved (Fig. 38).

*Ecology*: Terrestrial.

*Distribution*: Venezuela. Alt. 2,400–2,500 m.

*Notes*: According to Foldats (1968) this species differs from *C. superbiens* in ecarinate lateral lobes of the lip, narrower gynostemium wings and acute petals. Cyrtochilum *steyermarkii* can be confused with both *C. grandiflorum* and *C. baldeviamae* which, however, are characterized by much more complicated lip callus.

Dalström (2001) considered this species to be conspecific with *C. grandiflorum*, but while in *C. steyermarkii* petals are cohering apically, in *C. grandiflorum* they are not touching each other.

*Representative specimens*: VENEZUELA. **Mérida**: Por debajo del Pmo. La Negra, carretera hacia Bailadores, 2,500 m, *J.A. Steyermark & M. Rabe 96993* (VEN); Pmo. La Negra, *Hno. Antonio 1052* (Foldats, 1968). **Táchira**: Las Copas, por debajo de Pata de Judío, 2,400 m, *J.A. Steyermark, G.C.K. Dunsterville & E. Dunsterville 101250* (Foldats, 1968), the same loc. *G.C.K. Dunsterville & E. Dunsterville 1040* (Foldats, 1968), Prope coloniam Tovar, *A. Fendler 1376* (AMES!, UGDA-DLSz!—drawing).

***Cyrtochilum baldeviamae*** (Rchb.f.) Kraenzl. [published as *Cyrtochilum baldewiamae* (Rchb.f.) Kraenzl.], Notizbl. Bot. Gart. Berlin-Dahlem 7: 93. 1917. ≡ *Oncidium baldeviamae* Rchb.f., Gard. Chron. 1873: 915. 1873. TYPE: Colombia. [New Grenada]. *Carder 134* (holotype, W-R! 48878, UGDA-DLSz!—drawing).

**Figure 39:**
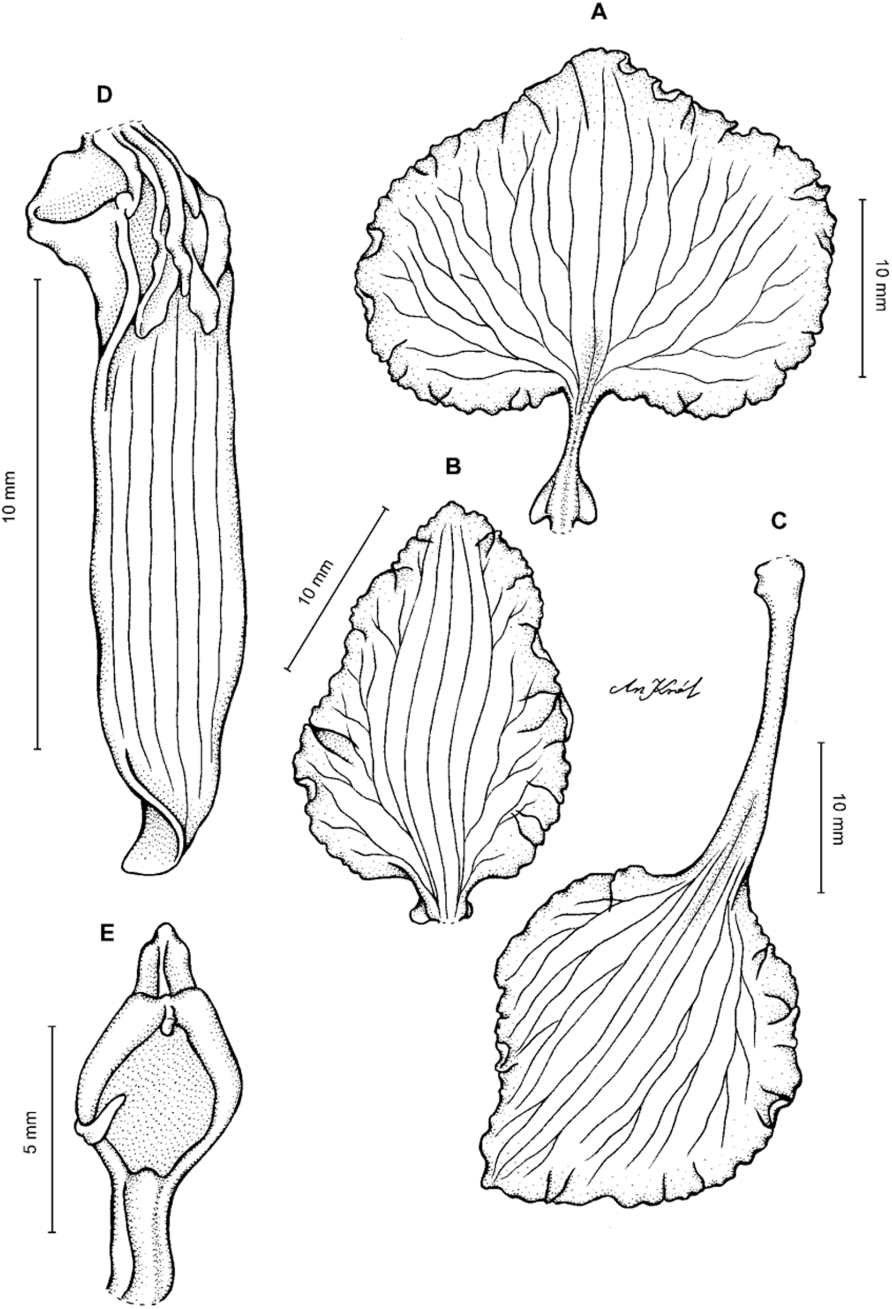
*Cyrtochilum baldeviamae* (Rchb.f.) Kraenzl. (A) Dorsal sepal; (B) Petal; (C) Lateral sepal; (D) Lip; (E) Gynostemium. Drawn by Anna Król from *Carder 134* (W-R).

Flowers large, conspicuous. Floral bracts 15–18 mm long. Pedicel and ovary 22–35 mm long. Dorsal sepal clawed; claw 6–8 mm long, rather narrow, canaliculate, with prominent triangular-ovate wings just above the base; blade 19–26 mm long, 17–25 mm wide, reniform, base truncate-subcordate, margins strongly and irregularly undulate and crenate, apex obtuse. Petals clawed; claw 2–3 mm long, basally winged; blade 18–22 mm long, 12–15 mm wide when spread, oblong-ovate, obtuse at the apex, falcate, margins undulate and crenate. Lateral sepals clawed; claw up to 20 mm long, narrow, with basal obscure wing; blade 20–26 mm long, 18–22 mm wide, broadly ovate in general outline, oblique, base broadly cuneate to subcordate, acute at the apex, margins slightly undulate and crenate. Lip 16–17 mm long in total, 3.5–8 mm wide, somewhat curved just above the base in natural position; basal part broadly obovate, convex; callus consisting of a narrow, simple central ridge flanked by two ridges longer than the middle one, margins entire; apical part ligulate-lanceolate, attenuate towards acute apex. Gynostemium 9–10 mm long, somewhat sigmoid, connate basally with the lip, lateral appendages digitate, not reaching the anther base (Fig. 39).

*Ecology*: Epiphyte.

*Distribution*: Colombia, Venezuela. Alt. 2,000–3,000 m.

*Notes*: By its strongly undulate and connate petals *C. baldeviamae* can be confused with *C. serratum*, but both species are easily separable by different lip form. In *C. serratum* the lip is hastate in outline, 3-lobed, with base cuneate, basal part is trapeziform in outline while the apical part is oblong obovate and minutely bifid at the apex. The other taxon easily misidentified with *C. baldeviamae* is *C. pozoi*. The latter, however, has much larger and complicated lip callus and acuminate petals.

*Representative specimens*: COLOMBIA. **Boyacá**: Alto Redondo. Somondoco, 12 October 1986, *M. Ospina H. 1173* (COL!). **Cauca**: El Tambo, La Romelia, desde la quebrada El Sopladero a la cabana del UAES PNN, 2,100–2,500 m, 10 October 1995, *N. Ruiz, G. Lozano & R. Sanchez 521* (COL!); PNN Munchique. El Tambo, vereda La Romelia, carretera de entrada al Parque, cerca de la cabana de Inderena, 2,600 m, 22 July 1993, *C. Barbosa & al. CB8558* (COL!). **Cundinamarca**: Cerros de los alrededores de Bogotá, 2,700–3,000 m, 11 June 1962. *C. Saravia 1287* (COL!, UGDA-DLSz!—drawing); Cordillera Oriental. 6 km N of Gutierrez, 2,835 m, 21 July 1944, *M. Grant 9624* (COL!, UGDA-DLSz!—drawing). [New Grenada]. *J. Carder 134* (W-R! 48878; UGDA-DLSz!—drawing). VENEZUELA. **Trujillo**: Bocono. Montanas de Misisi, carretera vieja Trujillo-Bocono, about 12 km by air NW of Bocono, 9°21′N 70°18′W, 2,000–2,400 m, 6 July 1990, *L. Dorr, L. Bernett & W. Diaz 7289* (MO!, UGDA-DLSz!—drawing).

***Cyrtochilum pozoi*** Königer, Arcula 2: 29. Königer (1994). TYPE: Ecuador. *Königer WK-26* (holotype, M; isotypes: K, QCA, herb. Königer).

Rhizome ascending to erect, internodes 8–10 cm long. Pseudobulbs up to 8 cm long, 1 cm wide, very narrowly ovoid, subcompressed, almost entirely concealed by sheaths, apically unifoliate at the apex, the base of the pseudobulbs with 4 leaf-bearing sheaths on each side, the blades narrowly ligulate-lanceolate, conduplicate towards the base, up to 40 cm long and 4.5 cm wide. Leaf up to 60 cm long and 6 cm wide, narrowly ligulate-lanceolate, coriaceous, conduplicate towards the base. Inflorescence 2–3 m long, arising from the axil of the sheath, twining, with several branches, each of them with 2–4 fragrant flowers; peduncle slender, with few close, amplexicaul sheaths. Flowers with yellow dorsal sepal that is densely speckled with light-brown, light-brown lateral sepals, yellow petals spotted with light-brown in the lower half and dark-brown lip edged with yellow, with middle lobe and callus yellow. Floral bracts 8 mm long, subinflate, cucullate, semiamplexicaul. Pedicel and ovary 4 cm long. Dorsal sepal clawed; claw 5 mm long, longitudinally furrowed within, with small auricles at the base; blade 25 mm long, 20 mm wide, orbicular-ovate, subcordate at base, apex obtuse, margins undulate. Petals shortly clawed; claw 4 mm long, broad-margined on both sides; blade 23 mm long, 14 mm wide, narrowly ovate, apex acuminate and cohering, convex towards the base, crisped at the margin. Lateral sepals slightly united at the base, clawed; claw 7 mm long, with a narrow margin on both sides, externally indistinctly auriculate at the base; blade 28 mm long, 17 mm wide, obliquely triangular-ovate, subundulate at the margin, unequally narrowed at the base. Lip 20 mm long, 7 mm wide at the base; basal part trapeziform, broader at the back, lateral lobes deflexed, fleshy, rigid, broader and acutely angled towards the base; middle lobe oblong-lanceolate, acuminate; basal callus oblong, microscopically cellular-pubescent, with a stout, apically lowering longitudinal lamella, at the base on both sides with a tuberculae in front, at the back with a serrate, oblique lamella on both sides, in the anterior portion with a lamella tuberculate in front on both sides, crossing arch-like into the midline lamella. Gynostemium about 7 mm long, wings on the base of the stigma small, obscure, subulate, ascending.

*Ecology*: Epiphytic in cloud forest.

*Distributioni* Ecuador. Alt. 2,400 m.

*Notes*: This species with cohering petals can be confused with *C. baldeviamae*, but unlike the latter it has larger and much complicated lip callus and acuminate petals. Cyrtochilum *pozoi* differs from *C. steyermarkii* in the form of gynostemium wings (obscure, subulate in *C. pozoi* vs digitate in *C. steyermarkii*) and lip callus (occupying basal half of the lip vs occupying basal third of the lip). Somewhat similar to *C. pozoi* is *C. xanthodon*. The latter species, however, has bifoliate, small pseudobulbs ca. 1 cm long, smaller flowers with segments being dark brown with yellow margins. Dorsal sepal blade is 12 × 9 mm with cuneate base, and lateral sepals have similar size. Petals are also quite small ca. 10 × 6 mm with cuneate base. Lip is 12 mm long and ca. 6-7 mm wide, with distinct callus form.

Dalström (2001) considered this species as conspecific with *C. detortum*, however, in the latter species the base of dorsal sepal is cuneate and its lamina is longer than wide.

*Representative specimens*: ECUADOR. Molleturo, Luz Maria, 2,400 m. Collected by A. Pozo in 1986, cultivated and flowered by *W. Königer WK-26* (K, M, QCA, herb. Königer). Collected and cultivated by A. Pozo, 20 November 1989, *C. Dodson & al. 17724* (RPSC—Dalström, 2010).

***Cyrtochilum xanthodon*** (Rchb.f.) Kraenzl., Notizbl. Bot. Gart. Berlin-Dahlem 7: 94. 1917. ≡ *Oncidium xanthodon* Rchb.f., Gard. Chron. 1868: 1338. 1868. TYPE: Colombia. [New Grenada]. *Backhouse & Sons 57* (holotype, W-R! 48977, UGDA-DLSz!—drawing).

= *Cyrtochilum phylloglossum* (Rchb. f.) Kraenzl., Notizbl. Bot. Gart. Berlin-Dahlem 7: 94. 1917. ≡ *Oncidium phylloglossum* Rchb. f., Gard. Chron., n.s.) 15: 169. 1881. TYPE: COLOMBIA. *Bull 551* (W).

= *Cyrtochilum plurituberculatum* D.E. Benn. & Christenson, Icon. Orchid. Peruv.: pl. 624. 2001. TYPE: PERU. *Meza T. ex Bennett 5090* (holotype, MOL).

= *Cyrtochilum confusum* D.E. Benn. & Christenson, Icon. Orchid. Peruv.: pl. 624. 2001. TYPE: PERU. *von Bismarck ex Bennett 761* (holotype, AMES).

**Figure 40:**
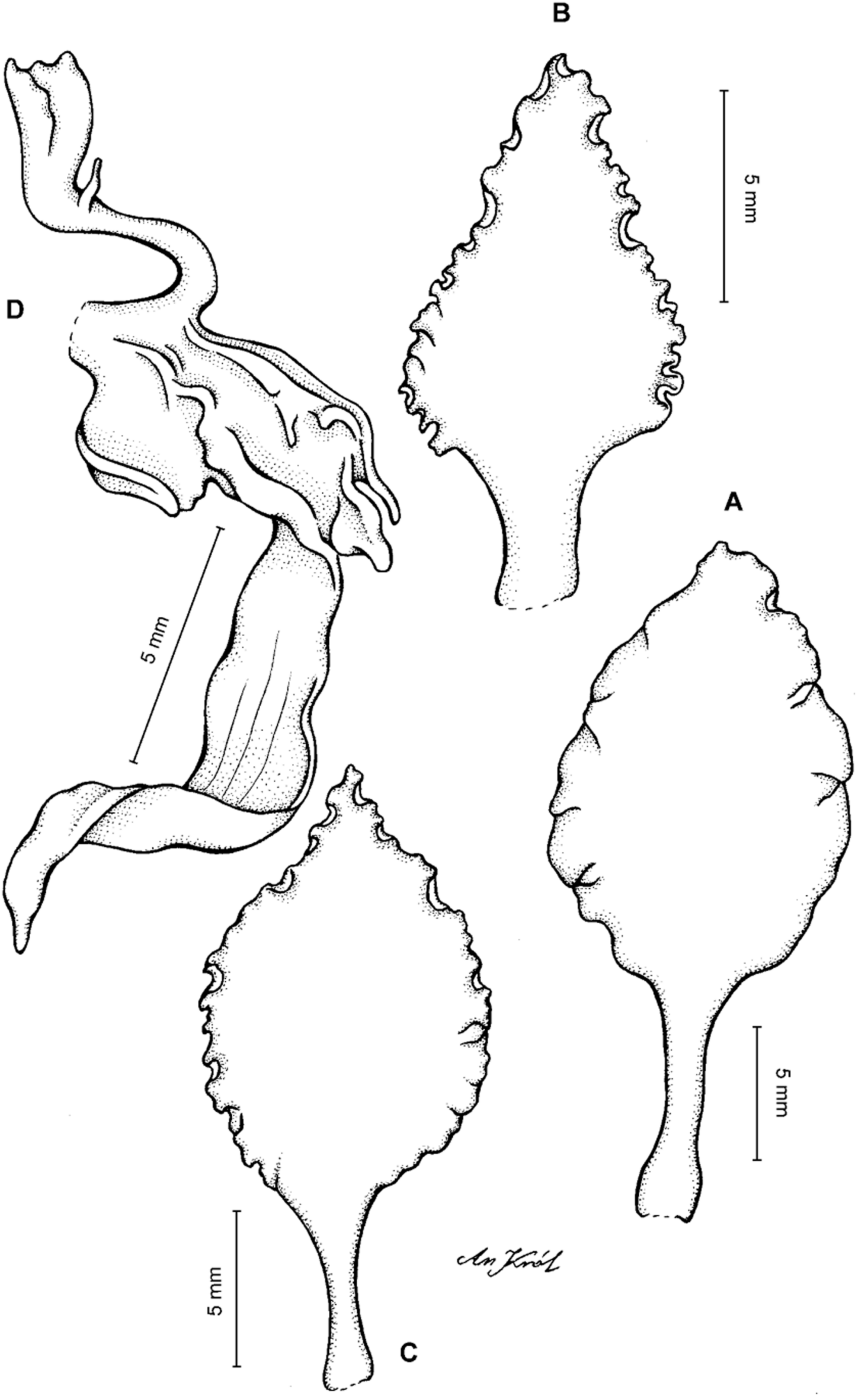
*Cyrtochilum xanthodon* (Rchb.f.) Kraenzl. (A) Dorsal sepal; (B) Petal; (C) Lateral sepal; (D) Lip and gynostemium, side view. Drawn by Anna Król from *Backhouse & Sons 57* (W-R).

Rhizome creeping. Pseudobulbs small, about 1 cm long according to illustration provided by Bennett & Christenson (2001), ovoid, sulcate with age, subtended by foliaceous sheaths subequal to the leaves. Leaves 2, up to 18 cm long and 3 cm wide, linear-lanceolate, acute-acuminate. Inflorescences elongate, vining panicles, branches 3–4-flowered. Flowers medium-sized, conspicuous, flower segments dark brown with yellow margins. Floral bracts 8 mm long. Pedicel and ovary 40 mm long. Dorsal sepal clawed; claw 8 mm long, narrow, canaliculate, with obscure rhombic wings just above the base; blade 12 mm long, 9 mm wide, ovate, base cuneate, apex obtuse, margins entire, somewhat undulate. Petals clawed; claw about 3–4 mm long, wide; blade 10 mm long, 6 mm wide when spread, obliquely triangular-ovate, base cuneate, apex obtuse, margins entire, strongly undulate-serrate. Lateral sepals prominently clawed; claw 7 mm long, narrow, with obscure wings at the base; blade 13 mm long, 9 mm wide, obliquely elliptic-ovate in general outline, apex attenuate, base cuneate, margins undulate-crenate. Lip 12 mm long in total, gently curved in natural position; basal part 6 mm long, 6–7 mm wide, rectangular-deltoid in general outline, base truncate, callus prominent consisting of a prominent plate with truncate apex and additional growths flanking main callus; apical part 6 mm long, 2 mm wide, ligulate, attenuate towards blunt apex. Gynostemium 6 mm long, strongly sigmoid, connate basally with the lip, swollen above the base, lateral appendages digitate, rudimentary, upcurved (Fig. 40).

*Ecology*: Terrestrial in open shrub-covered slopes surrounded by wet montane forest.

*Distribution*: Colombia, Ecuador (Dalström, 2010), Peru (Schweinfurth, 1961).

*Notes*: *Cyrtochilum xanthodon* can be easily distinguished from any other species of this group by having a strongly sigmoid gynostemium with digitate, rudimentary lateral appendages. As mentioned above, this species is somewhat similar to *C. pozoi*, but has larger flowers differently coloured. The dorsal sepal of *C. pozoi* is yellow, densely speckled with light-brown, lateral sepals are light-brown, petals are yellow spotted with light-brown in the lower half and lip is dark-brown edged with yellow, with yellow middle lobe and callus. The dorsal sepal is 25 × 20 mm with subcordate base, lateral sepals are somewhat longer and narrower, petals are 23 × 14 mm, and lip 20 × 7 mm.

*Cyrtochilum plurituberculatum* proposed by Bennett & Christenson (2001) from Peru appears to be conspecific with *C. xanthodon*. In our opinion it differs from the latter by the more undulate sepals only. According to Bennett & Christenson (2001) *C. confusum* is distinguished from *C. plurituberculatum* by acute lip and broader column wings, however, these differences in our opinion are not sufficient to consider it as a separated species.

*Representative specimens*: COLOMBIA. [New Grenada], *Backhouse & Sons 57* (W-R! 48977, UGDA-DLSz!—drawing). PERU. **Amazonas**: Chachapoyas, *J. Meza T. ex D.E. Bennett 5090* (MOL—flower in spirit). **Cajamarca**: Santa Cruz. Taulis, above Palmito, 2,900 m, *K. von Bismarck ex D.E. Bennett 761* (AMES—Bennett & Christenson, 2001).

***Cyrtochilum stegasaurum*** D.E. Benn. & Christenson, Icon. Orchid. Peruv.: pl. 625. 2001. TYPE (Trujillo, 2014): Peru. *Jara P. ex D.E. Bennett 6441* (holotype, MOL†); *Campoverde s.n. ex Bennett 6444* (lectotype, MOL).

Rhizome creeping, short, pseudobulbs appearing almost caespitose. Pseudobulbs 9–10 cm long, up to 7 cm wide, oblongoid to oblongoid-ovoid, somewhat compressed, unifoliate, new growths enclothed with several pairs of foliaceous sheaths. Leaves absent in type material. Inflorescence about 180–200 cm long, sparsely branching, branches loosely few-flowered. Flowers rather large, conspicuous. Floral bracts 10 mm long. Pedicel and ovary 30 mm long. Dorsal sepal clawed; claw 3 mm long, relatively wide, canaliculate, without any basal wing; blade about 22 mm long, 18–19 mm wide, broadly ovate, base truncate, apex attenuate, obtuse, reflexed, margins entire, delicately undulate. Petals almost sessile; blade 18 mm long, about 12–13 mm wide when spread, obliquely broadly ovate, base cuneate, apex obtuse, reflexed, margins undulate, apically somewhat serrate. Lateral sepals prominently clawed; claw 5–6 mm long, broad, without any basal wing, connate together; blade 20–22 mm long, 18–19 mm wide, obliquely ovate in general outline, apex attenuate, base cuneate, margins delicately undulate. Lip 12–13 mm long in total, almost straight in natural position; basal part 6–7 mm wide, deltoid in general outline, base truncate, callus consisting of a prominent plate with truncate apex and additional growths flanking main callus; apical part 4–5 mm wide, broadly elliptic, attenuate towards blunt apex, covered by series of irregular outgrowths running up to the lip apex. Gynostemium 6 mm long, erect, connate basally with the lip, somewhat swollen above the base, lateral appendages missing.

*Ecology*: Epiphyte in wet montane forest (Bennett & Christenson, 2001).

*Distribution*: Peru. Alt. 1,000–3,000 m.

*Notes*: No doubt, this species is similar to *C. xanthodon*, from which it can be separated by relatively shortly clawed tepals, broadly elliptic lip apical part, but most of all by series of irregular outgrowths running up to the lip apex. Gynostemium of *C. stegasaurum* is erect, and not sigmoid as in *C. xanthodon*.

*Representative specimens*: PERU. [**Piura**]: Huancabamba, 15–20 km NE from Huancabamba city, 3,000 m, 22 September 1993, *J. Campoverde s.n. ex D.E. Bennett 6444* (MOL—flower in spirit); 6 km SE Tingo Maria, 1,000–1,150 m, *E. Jara P. ex D.E. Bennett 6441* (MOL—Bennett & Christenson, 2001).

***Cyrtochilum ustulatum*** (Rchb.f.) Kraenzl., Notizbl. Bot. Gart. Berlin-Dahlem 7: 94. 1917. ≡ *Oncidium ustulatum* Rchb.f., Gard. Chron., n.s., 19: 340. 1883. TYPE: Colombia. *Carder ex Shuttleworth s.n*. (holotype, W-R! 16085, UGDA-DLSz!—drawing).

**Figure 41:**
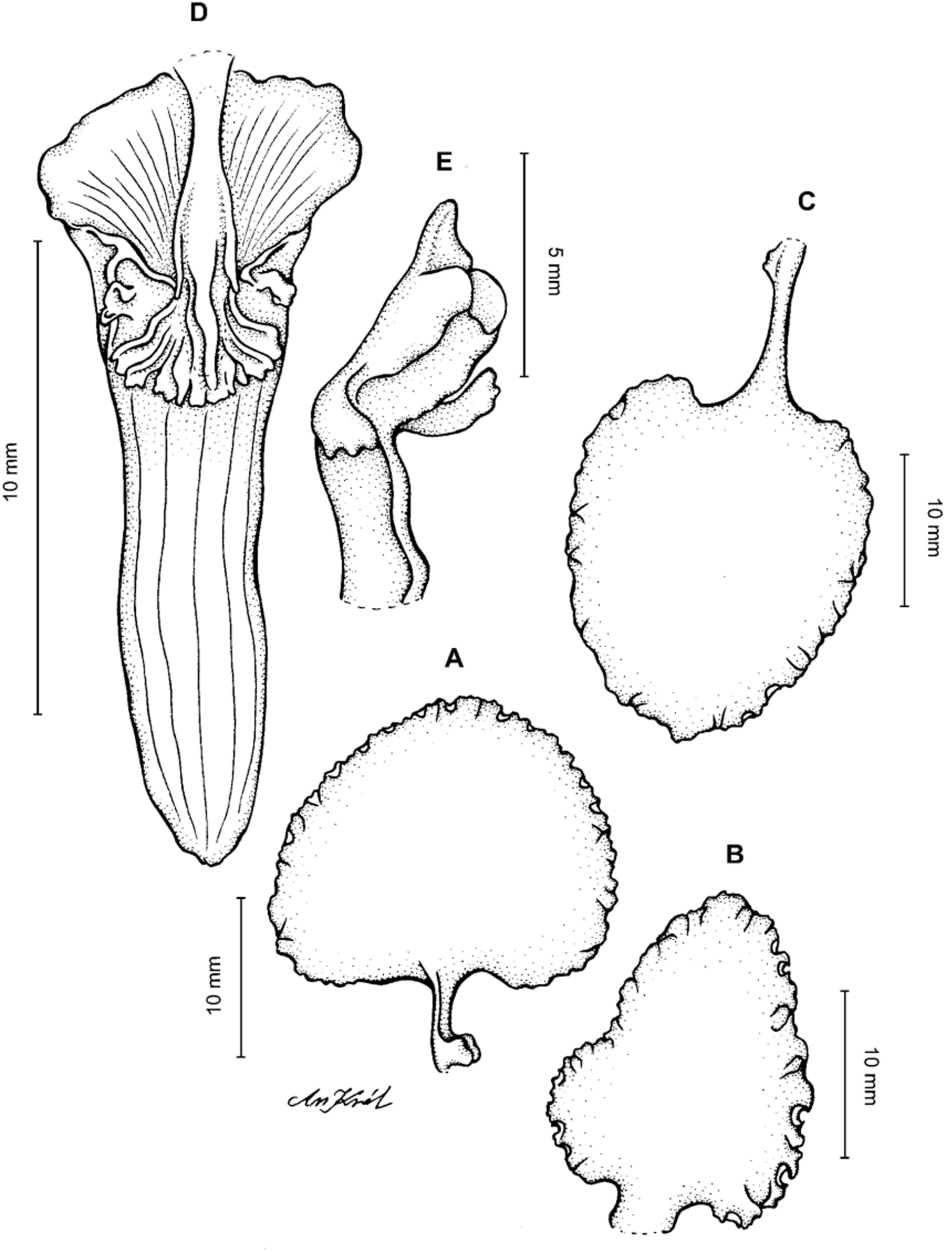
*Cyrtochilum ustulatum* (Rchb.f.) Kraenzl. (A) Dorsal sepal; (B) Petal; (C) Lateral sepal; (D) Lip; (E) Gynostemium, side view. Drawn by Anna Król from *Carder ex Shuttleworth s.n*. (W-R).

Vegetative parts unknown. Flowers large, showy, conspicuous. Dorsal sepal clawed; claw 4–5 mm long, narrow, canaliculate, with prominent rhombic wings just above the base; blade 18–19 mm long, 21 mm wide, reniform, margins strongly undulate and crenate, base truncate, apex rounded. Petals clawed; claw about 2 mm long; blade 20 mm long, 15 mm wide when spread, obliquely elliptic-ovate, base truncate, apex rounded, margins strongly serrate-undulate. Lateral sepals prominently clawed; claw 12 mm long, narrow, with rather obscure wing on the outer margin; blade 23 mm long, 22 mm wide, obliquely cordate-ovate in general outline, apex obtuse, margins slightly undulate. Lip 17 mm long in total, gently curved in natural position; basal part about 5 mm long, 6.5 mm wide, transversely elliptic-rhombic in general outline, base truncate, callus prominent consisting of a prominent, median ridge, papillate on the upper surface expanding apically into flabellate, dissected plate, with some additional thickenings beyond the main callus; apical part 10–12 mm long, 3–3.5 mm wide, ligulate, apex blunt. Gynostemium 9 mm long, sigmoid, connate basally with the lip, lateral appendages linear-obtriangular, obscurely 3–lobed at the apex, upcurved, not reaching the anther base (Fig. 41).

*Ecology*: No data.

*Distribution*: Colombia.

*Notes*: The unique character of this species is lip callus, forming a kind of flabellate, dissected plate. In other species having papillate or ciliolate lip callus as *C. aemulum*, *C. tetracopis* and *C. monachicum* its shape is different and never forms such a plate.

*Representative specimens*: COLOMBIA. *J. Carder ex Shuttleworth s.n*. (W-R! 16085; UGDA-DLSz!—drawing).

***Cyrtochilum garayi*** Szlach. & Kolan., ***sp. nov***. TYPE: Ecuador. *Garay 1029* (holotype, AMES! 116667, UGDA-DLSz!—drawing).

Diagnosis: *This is the only species of Tetracopsis-group with completely reduced lateral gynostemium appendages. This species is distinguished by the lip callus consisting of a prominent, median ridge much elevated at the apex, which is rhombic-rectangular when observed from the side, much exceeding the other callus segments. It is papillate on the upper surface and flanked by shorter ridges, divided into 3 digitate projections*.

**Figure 42:**
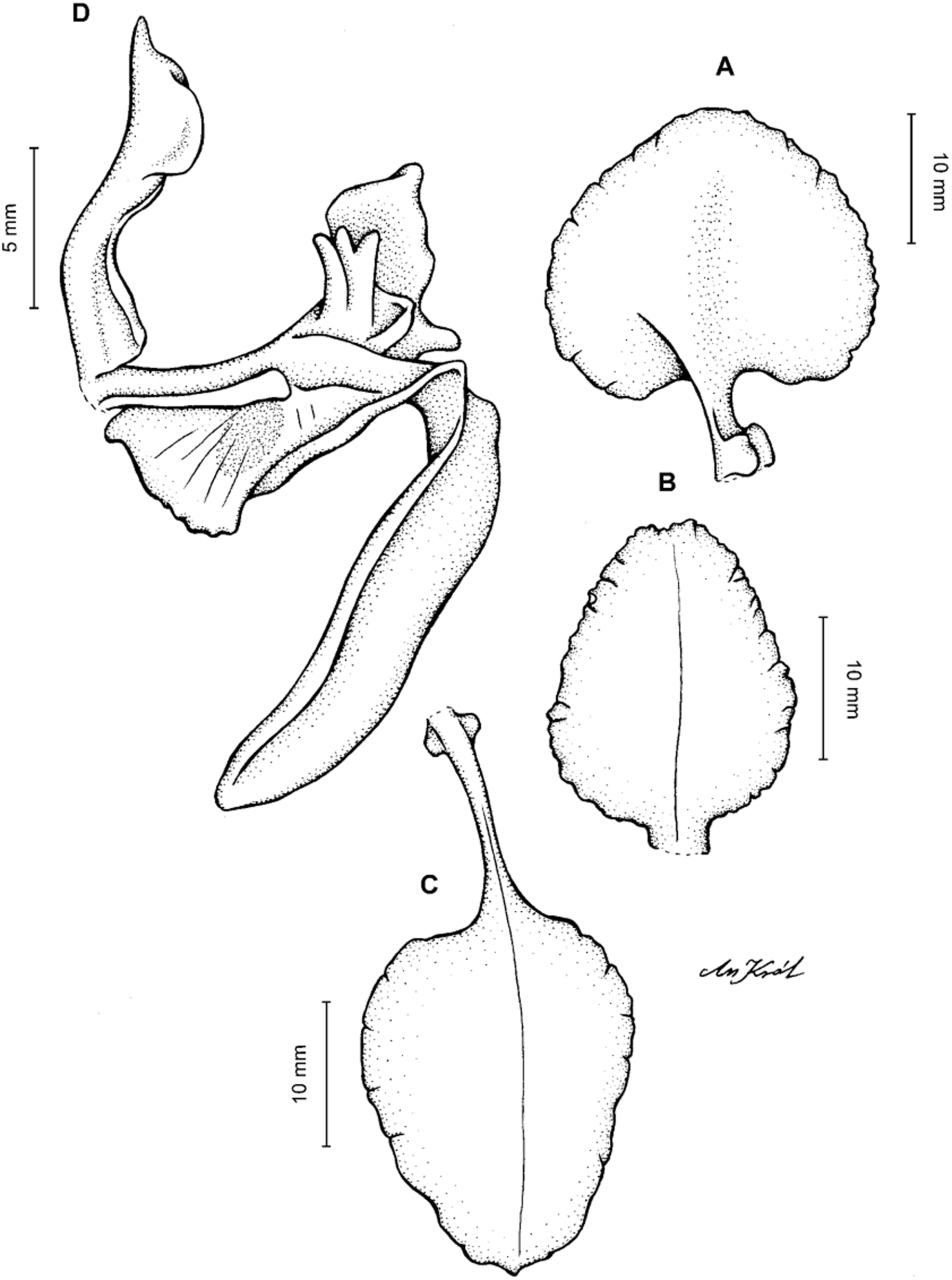
*Cyrtochilum garayi* Szlach. & Kolan. (A) Dorsal sepal; (B) Petal; (C) Lateral sepal; (D) Lip and gynostemium. Drawn by Anna Król from *Garay 1029* (AMES).

Vegetative parts unknown. Flowers large, showy, conspicuous. Floral bracts 23–25 mm long. Pedicel and ovary 45 mm long. Dorsal sepal clawed; claw 7 mm long, narrow, canaliculate, with prominent rhombic wings just above the base; blade 22 mm long, 23 mm wide, reniform, margins somewhat undulate and crenate, base subcordate, apex rounded. Petals clawed; claw about 2 mm long, wide; blade 23 mm long, 19 mm wide when spread, obliquely elliptic-ovate, base cuneate, apex emarginate, margins somewhat crenate and undulate. Lateral sepals prominently clawed; claw 11 mm long, narrow, with a pair of wings at the base; blade 26 mm long, 19 mm wide, obliquely ovate in general outline, apex obtuse with short apiculus, margins slightly undulate. Lip 22 mm long in total, gently curved in natural position; basal part about 9 mm long and wide, broadly triangular or deltoid in general outline, base subcordate, callus consisting of a prominent, median ridge much elevated at the apex with small basal digitate outgrowth, papillate on the upper surface, flanked by shorter ridges, divided into 3 digitate projections; apical part 13 mm long, 3.5 mm wide, ligulate, apex obtuse. Gynostemium 11–12 mm long, somewhat sigmoid, connate basally with the lip, lateral appendages reduced (Fig. 42).

*Etymology*: In honour of the collector of the type specimen.

*Ecology*: No data.

*Distribution*: Ecuador. Alt. 2,300–3,000 m.

*Notes*: This species has unique lip callus. Additionally, this is the only representative of this group with completely reduced lateral gynostemium appendages. Both these characters separate *C. garayi* from other species having petals not touching each other. In the similar *C. ustulatum* the lip callus consists of median ridge which expands apically into a flabellate, dissected plate and lateral appendages of gynostemium are linear-obtriangular and obscurely 3-lobed at the apex.

*Representative specimen*: ECUADOR. Between Quito and Santo Domingo, 2,300–3,000 m, 6 January 1970, *L.A. Garay 1029* (AMES!, UGDA-DLSz!—drawing).

*Conservation status*: This species is described based on material collected in 1970. We cannot assess the current threats for this taxon hereby the “data deficient” (DD) category should be applied according to IUCN (2001).

**(4) *Detortum-*group**

Basal lip part rhombic or deltoid, apical part ligulate, callus with large median keel. Dorsal sepal with broadly cuneate base, lamina usually longer than wide. Lateral sepals basally more or less connate. Sepals and petals usually strongly undulate. Gynostemium basally connate with the lip, appendages usually simple, small.

The group includes about a dozen of species reported mostly from Ecuador, Colombia and Venezuela.

### Key to the species

1 Lip callus papillate on the upper surface*C. ventilabrum*
1* Lip callus glabrous2
2 Gynostemium sigmoid, connate in the basal fifth or quarter with the lip3
2* Gynostemium suberect, connate with the lip in less than basal fifth7
3 Gynostemium lateral appendages with fimbriate lower margins*C. mandibulare*
3* Gynostemium lateral appendages with entire margins4
4 Lateral sepals free to the base*C. cinereobrunneum*
4* Lateral sepals more or less connate basally5
5 Lip callus massive, consisting of central ridge, more or less wrinkled apically, flanked by lower keel on each side, with wrinkled upper surface*C. cuencanum*
5* Lip callus apically much elongate and free forming a kind of nose-like, truncate projection, flanked by additional, uneven keels***C. lehmannii***
7 Gynostemium appendages obscure, rudimentary8
7* Gynostemium appendages short or long, but prominent9
8 Gynostemium base with obscure, rounded wings*C. gyriferum*
8* Gynostemium base with prominent triangular outgrowths*C. insculptum*
9 Dorsal sepal suborbicular-ovate, as long as wide*C. detortum*
9* Dorsal sepal ovate to ovate-lanceolate, longer than wide10
10 Lateral sepals about 5 times longer than wide*C. crispitissimum*
10* Lateral sepals up to twice longer than wide11
11 Gynostemium lateral appendages oblong, obliquely truncate and crenate along lower part*C. falcipetalum*
11* Gynostemium lateral appendages obliquely obtriangular, thick*C. aciculatum*

***Cyrtochilum ventilabrum*** (Rchb.f. & Warsz.) Kraenzl., Notizbl. Bot. Gart. Berlin-Dahlem 7: 92. 1917. ≡ *Oncidium ventilabrum* Rchb.f. & Warsz., Bonplandia (Hannover) 2: 101. 1854. TYPE: Colombia or Peru? [New Grenada]. *Warszewicz s.n*. (holotype, W-R! 36984; isotype, AMES!, UGDA-DLSz!—drawing).

**Figure 43:**
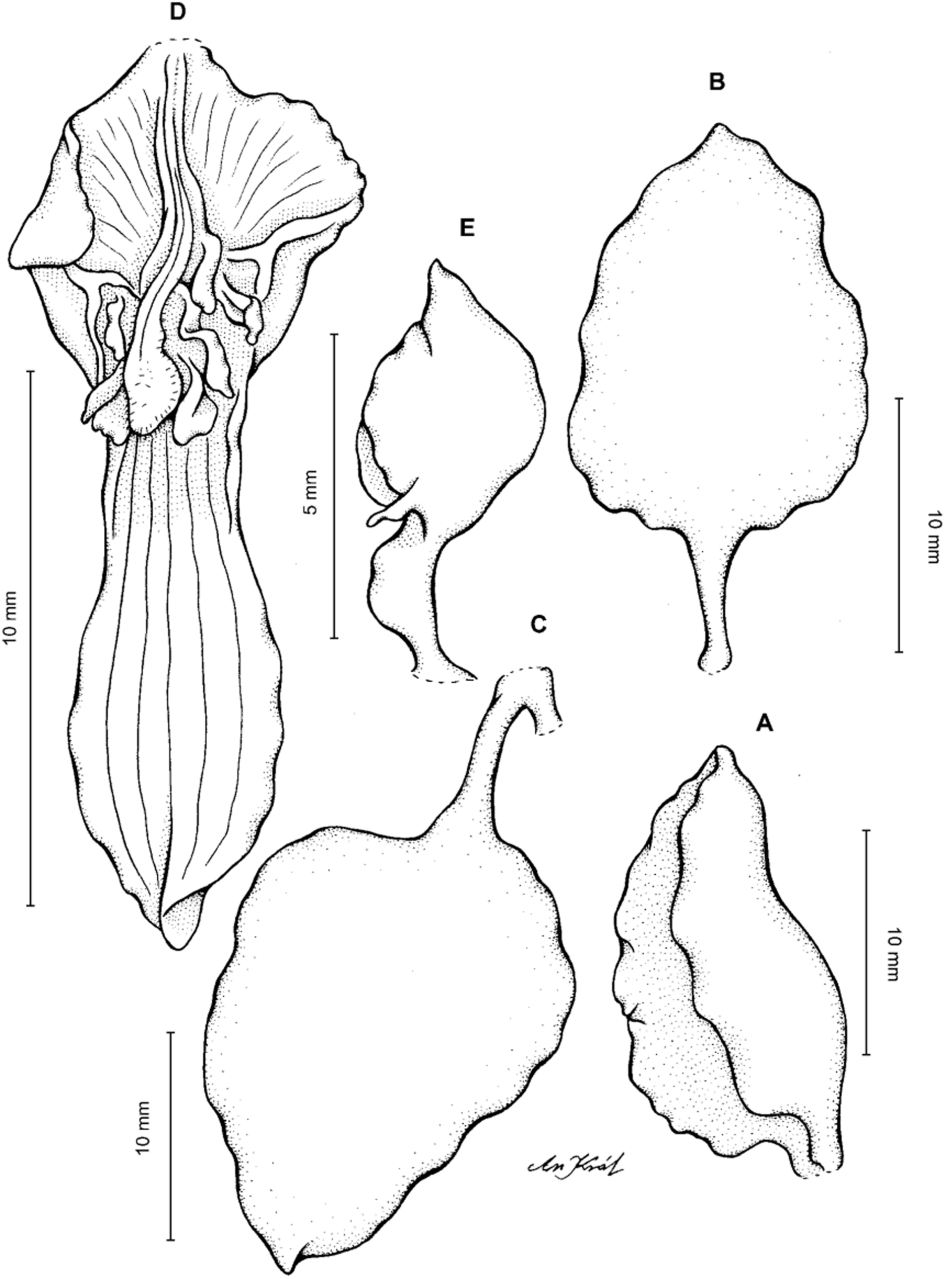
*Cyrtochilum ventilabrum* (Rchb.f. & Warsz.) Kraenzl. (A) Petal; (B) Dorsal sepal; (C) Lateral sepal; (D) Lip; (E) Gynostemium, side view. Drawn by Anna Król from *Warszewicz s.n*. (W-R).

Vegetative parts unknown. Inflorescence up to 4 m long, trailing. Flowers medium-sized, conspicuous, petals white with brown bands. Floral bracts 7 mm long. Pedicel and ovary 21 mm long. Dorsal sepal clawed; claw 4 mm long, narrow, canaliculate, wingless; blade 18 mm long, 11 mm wide, elliptic-ovate, margins entire, somewhat undulate, base broadly cuneate, apex obtuse. Petals clawed; claw about 2 mm long; blade 18 mm long, 13 mm wide when spread, obliquely elliptic-ovate, base truncate, apex obtuse, margins entire, slightly undulate. Lateral sepals prominently clawed; claw 6 mm long, narrow, wingless, basally connate to each other; blade 17 mm long, 16 mm wide, obliquely elliptic-ovate in general outline, base truncate, apex obtuse, margins entire, slightly undulate. Lip 17 mm long in total, gently curved in natural position; basal part 7 mm long and wide, deltoid in general outline, base broadly cuneate, callus consisting of a prominent, median ridge, papillate on the upper surface, flanked by 4-lobed ridges on each side, with an additional thickenings beyond the main calus; apical part 10 mm long, 3.5–4 mm wide, ligulate, somewhat expanded towards apex, then attenuate towards blunt apex. Gynostemium 7 mm long, sigmoid, much swollen at the apex, connate basally with the lip, lateral appendages digitate, rudimentary (Fig. 43).

*Ecology:* Terrestrial or lithophytic.

*Distribution*: Colombia (Idárraga-Piedrahita et al., 2011), Ecuador (Dalström *in* Dodson & Luer, 2010), Peru?, Venezuela. Alt. 1,800–3,000 m.

*Notes*: This is the only species of this group having a papillate lip callus, consisting of a prominent central ridge flanked by 4-lobed ridges on each side and relatively weakly undulate tepals.

*Representative specimens*: VENEZUELA. **Trujillo**: Distr. Boconó. Páramo de Guaramacal, SE of Boconó (ca. 9°15′N 70°16′W) on road from Boconó to Guaramacal, 1,800–3,000 m, 19 October 1990, *L.J. Dorr, L.C. Barnett & R. Rosales 7453* (AMES!, UGDA-DLSz!—drawing). COLOMBIA or PERU? [New Grenada]. *J. Warszewicz s.n*. (AMES!, W-R!, UGDA-DLSz!—drawing).

***Cyrtochilum detortum*** (Rchb.f.) Kraenzl., Notizbl. Bot. Gart. Berlin-Dahlem 7: 92. 1917. ≡ *Oncidium detortum* Rchb.f., Gard. Chron., ser. 3, 3: 392. 1888. TYPE: *Sander s.n*. (holotype, W-R! 48969, UGDA-DLSz!—drawing).

Rhizome creeping, bracteate. Pseudobulbs distant, well hidden by the large, distichous, folicaceous sheaths. Leaves subpetiolate, conduplicate, obovate, acute to acuminate. Inflorescence up to about 2 m long, from the base of uppermost sheath, wiry, paniculate with widely spaced few-flowered side branches. Flowers large, conspicuous, showy, sepals dark brown, dorsal sepal with yellow margin, petals brown with yellow reticulation and apical area, lip basally brown, apically yellow, callus yellow with brown spots and a white basal patch. Floral bracts 15 mm long. Pedicel and ovary about 45 mm long. Dorsal sepal clawed; claw 7 mm long, rather narrow, canaliculate, with prominent elliptic-rhombic wings just above the base; blade 14 mm long and wide, suborbicular-ovate, base rounded, margins strongly irregularly undulate and crenate, apex obtuse. Petals clawed; claw about 2–3 mm long; blade 20 mm long, 11 mm wide when spread, obliquely elliptic-ovate, obtuse at the apex, margins strongly undulate-serrate. Lateral sepals prominently clawed; claw 7 mm long, narrow, with basal wing on the outer margin; blade 23 mm long, 9 mm wide, obliquely oblong elliptic in general outline, basally cuneate, obtuse at the apex, margins strongly undulate. Lip 14 mm long in total, curved in natural position exposing massive callus; basal part 5 mm long, about 10 mm wide, almost rhombic in outline, base truncate, convex along the centre; callus complicated consisting of narrow central ridge, flanked by some lobules on each side, and by two ridges with apices prominently exceeding the middle ridge, margins entire; apical part 9 mm long, about 2 mm wide, ligulate, attenuate towards acute apex. Gynostemium 8 mm long, subarcuate, connate basally with the lip, lateral appendages oblique, oblong, unequally bilobed, not reaching the anther base.

**Figure 44:**
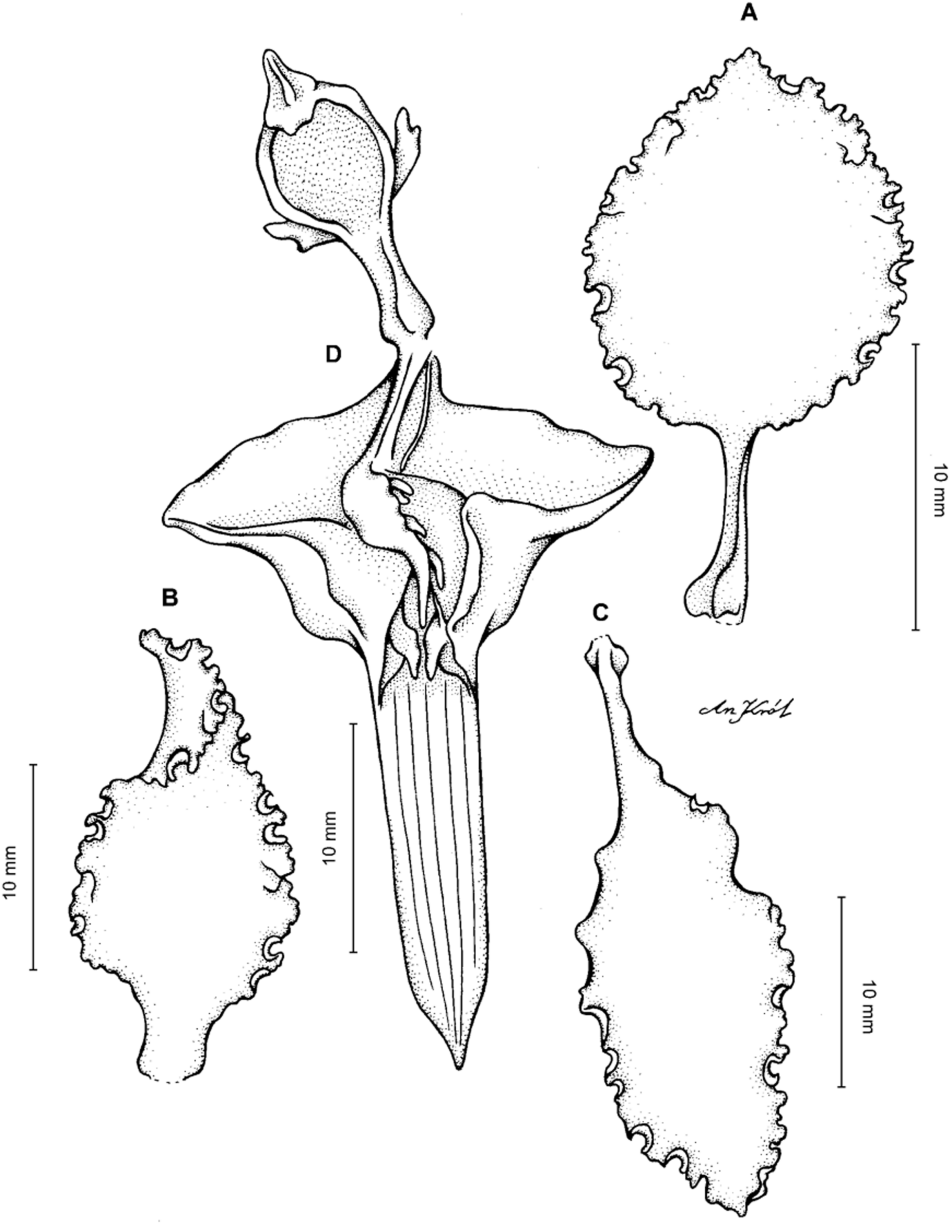
*Cyrtochilum detortum* (Rchb.f.) Kraenzl. (A) Dorsal sepal; (B) Petal; (C) Lateral sepal; (D) Lip and gynostemium. Drawn by Anna Król from *Sander s.n*. (W-R).

*Ecology*: Terrestrial, epiphytic or lithophytic (Fig. 44).

*Distribution*: Ecuador (Dodson & Luer, 2010), Peru (Zelenko & Bermúdez, 2009), Venezuela (Dunsterville & Garay, 1979; Dorr, 2014).

*Notes*: By the form of lip callus this species can be confused with *C. ventilabrum*. Unlike the latter, however, the callus is glabrous, tepals are strongly undulate and the dorsal sepal is almost orbicular. From the similar *C. crispitissimum*, *C. detortum* differs also in form of the dorsal sepal which is suborbicular-ovate, as long as wide in the latter species and ovate to ovate-lanceolate, longer than wide in *C. crispitissimum*.

According to Dalström (2010) *Oncidium sanderianum* Rolfe [≡ *Cyrtochilum sanderianum* (Rolfe) Königer] is conspecific with *C. detortum*. We did not examine any material representing *O. sanderianum* hereby we are not able to confirm this recognition.

*Representative specimen*: ORIGIN UNKNOWN. *F. Sander s.n*. (W-R!).

***Cyrtochilum cuencanum*** Kraenzl., Notizbl. Bot. Gart. Berlin-Dahlem **7**: 92. 1917. TYPE: Ecuador. cult. *Sander* (holotype, W-R! 13462, UGDA-DLSz!—drawing).

**Figure 45:**
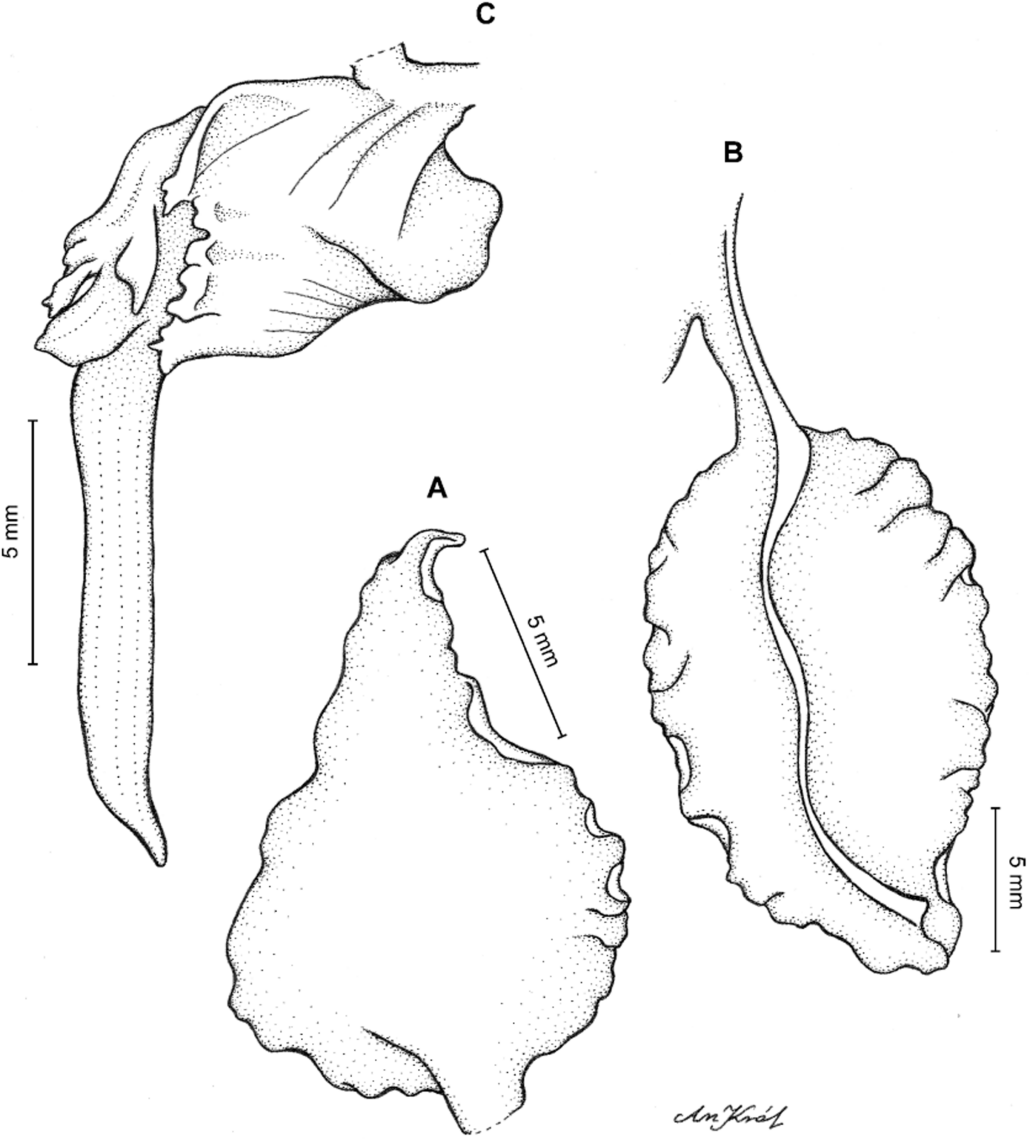
*Cyrtochilum cuencanum* Kraenzl. (A) Petal; (B) Lateral sepal; (C) Lip. Drawn by Anna Król from *Sander s.n*. (W-R).

Rhizome creeping, bracteate. Psudobulbs distant. Leaves and inflorescence not seen. Flowers large, conspicuous, showy. Floral bracts 15 mm long. Pedicel and ovary about 25 mm long. Dorsal sepal clawed; claw 4 mm long, rather narrow, canaliculate, wingless; blade 16 mm long, 12 mm wide, oblong ovate, base rounded, margins irregularly undulate, apex obtuse to emarginate. Petals clawed; claw about 2 mm long; blade 20 mm long, 11 mm wide when spread, obliquely triangular-ovate, acute at the apex, margins undulate. Lateral sepals prominently clawed; claw 7–8 mm long, narrow, wingless, in the basal half connate together; blade 24 mm long, 9 mm wide, obliquely oblong ovate in general outline, basally cuneate, obtuse at the apex, margins strongly undulate. Lip 16 mm long in total, curved in natural position exposing massive callus; basal part 7 mm long, about 10 mm wide, almost rhombic-obtriangular in outline, base truncate, convex along the centre; callus complicated consisting of massive, central ridge, more or less wrinkled apically, flanked by lower keel on each side, with wrinkled upper surface; apical part 10 mm long, about 2.5 mm wide, ligulate, attenuate towards acute apex. Gynostemium about 8 mm long, sigmoid, connate basally with the lip, lateral appendages very obscure (Fig. 45).

*Ecology*: Terrestrial, epiphytic or lithophytic in cloud forest.

*Distribution*: Ecuador, Peru (Zelenko & Bermúdez, 2009), Venezuela (Dunsterville & Garay, 1979; Dorr, 2014). Alt. 1,200–2,700 m.

*Notes*: This species along with *C. insculptum*, *C. gyriferum* and *C. lehmannii* can be characterized by very obscure or missing gynostemium appendages. The gynostemium of *C. insculptum* and *C. gyriferum* is erect or suberect, only basally connate with the lip. In *C. cuencanum* and *C. lehmannii* gynostemium is strongly sigmoid, connate with the lip along its basal fifth or quarter. Cyrtochilum *cuencanum* has larger flowers than *C. lehmannii*, with claw of lateral sepals ca. 3 times shorter than blade (vs claw being ca. 2/3 of blade length), and basal lip part wider than long (vs longer than wide).

*Representative specimens*: ECUADOR**. Tungurahua**: Slopes of volcán Tungurahua, 2,700 m, *S. Dalström 2761* (SEL, color transparency—Dalström, 2010); Río Topo, 1,200–1,400 m, collected and flowered in cultivation by Hierro, 21 March 1996, *S. Dalström 2189* (SEL—Dalström, 2010). **Azuay**: cult. at Ushupud, *C. Dodson 15493* (MO—Dalström, 2010).

***Cyrtochilum falcipetalum*** (Lindl.) Kraenzl., Notizbl. Bot. Gart. Berlin-Dahlem 7: 92. 1917. ≡ *Oncidium falcipetalum* Lindl., Orchid. Linden.: 14. 1846. TYPE: Venezuela. *Linden 626* (holotype, K-L; isotypes: BM, W!).

**Figure 46:**
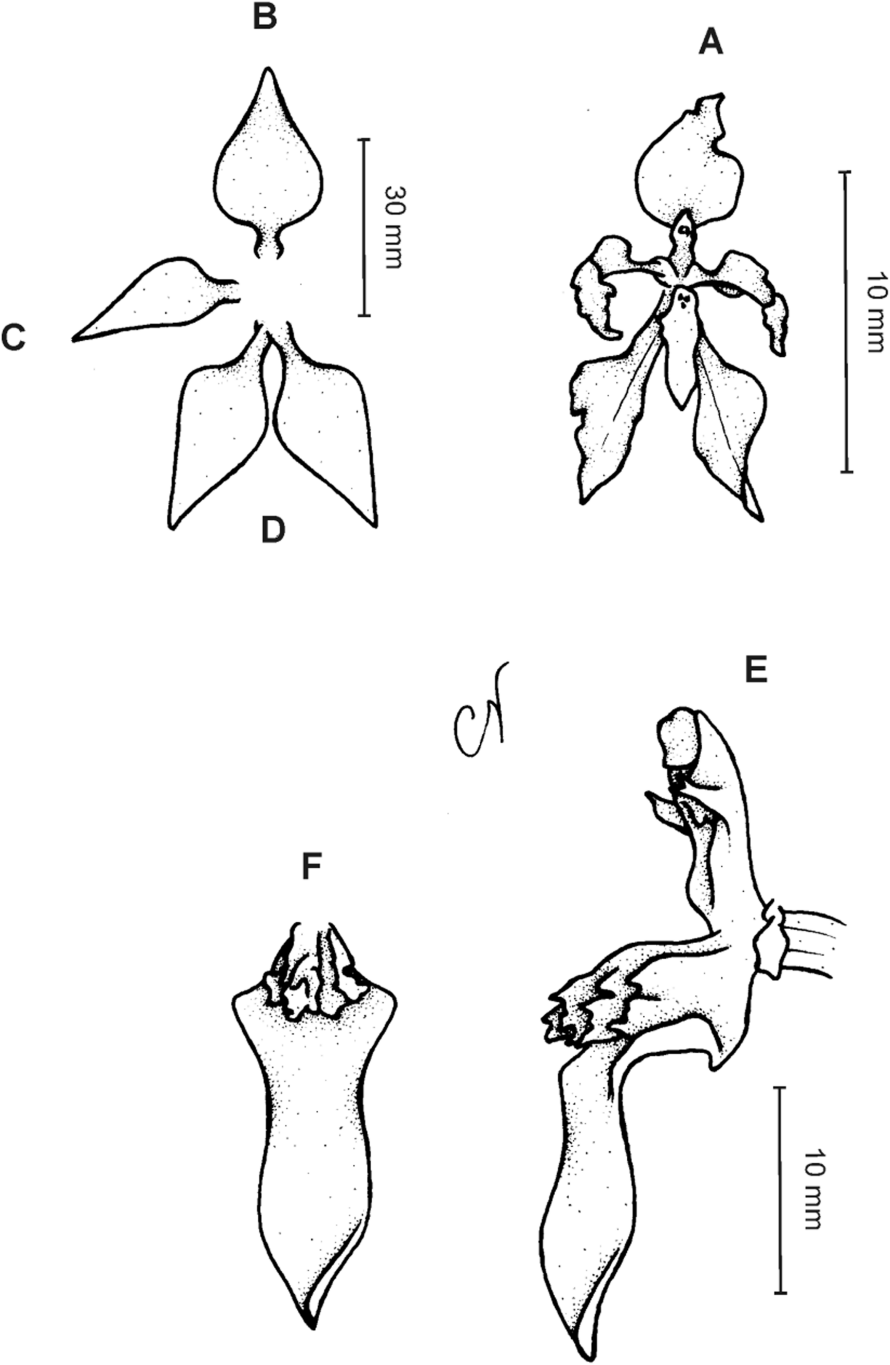
*Cyrtochilum falcipetalum* (Lindl.) Kraenzl. (A) Flower; (B) Dorsal sepal; (C) Petal; (D) Lateral sepals; (E) Gynostemium and lip, side view; (F) Lip, front view. Redrawn by Natalia Olędrzyńska from Dunsterville & Garay (1959).

Rhizome ascending, rather short. Pseudobulbs up to 10 cm long and 3 cm in diameter, oblong-ovoid, smooth, slightly compressed, enclothed basally with 3–4 leafy sheaths. Leaf 1, about 50 cm long and 4.5 cm wide, linear-oblanceolate, acute to shortly acuminate. Inflorescence 3–4 m long, climbing, distantly branching, branches loosely few-flowered. Flowers brown, petals and lip marked with yellow. Floral bracts 17 mm long. Pedicel and ovary 30 mm long. Dorsal sepal clawed; claw 5 mm long, with a pair of subrectangular appendages; blade 24 mm long, 21 mm wide, ovate, acute, margin crenate and slightly undulate. Petals clawed; claw 3 mm long, with a pair of semicircular appendages; lamina 24 mm long, 16 mm wide, obliquely ovate, acute, margin undulate. Lateral sepals clawed; claw 7 mm long, with a small appendage at the base; lamina 25 mm long, 18–20 mm wide, ovate, cuneate at the base, truncate at the apex with a small apiculus, margin slightly undulate. Lip obscurely 3-lobed at the base; basal part 6 mm long and wide, triangular, cuneate at base; callus basal, consisting of 3 oblong projections, the middle one the longest, with a series of digitate appendages in the apical part; middle lobe 17 mm long, 4.2 mm wide, ligulate, acute. Gynostemium 12 mm long, suberect, lateral appendages oblong, obliquely truncate and crenate along lower part (Fig. 46).

*Ecology*: Terrestrial in montane forest.

*Distribution*: Colombia (Idárraga-Piedrahita et al., 2011), Venezuela. Alt. 2,000–2,400 m.

*Notes*: In flower morphology *C. falcipetalum* appears to be similar to *C. cuencanum*. Both species can be easily distinguished by the gynostemium architecture. It is sigmoid with very obscure lateral appendages in the latter species, unlike *C. falcipetalum*, where it is suberect with oblong, truncate and crenate lateral appendages. Cyrtochilum *falcipetalum* differs from similar *C. aciculatum* in the form of gynostemium appendages. These are oblong, obliquely truncate and crenate along lower part in *C. falcipetalum* and obliquely obtriangular, thick in *C. aciculatum*.

*Representative specimens*: VENEZUELA. **Trujillo**: Distr. Boconó. Montañas de Misisí, carretera vieja Trujillo-Boconó, about 12 km. by air NW of Boconó, 2,000–2,400 m, 6 July 1990, *L.J. Dorr 7289* (MO!); *Sine loc*., *J. Linden 626* (BM, K, W!).

***Cyrtochilum insculptum*** (Rchb.f.) Kraenzl., Notizbl. Bot. Gart. Berlin-Dahlem 7: 94. 1917. ≡ *Oncidium insculptum* Rchb.f., Gard. Chron. 1872: 1035. 1872. TYPE: Ecuador. *Day s.n*. (holotype, W-R! 37003, UGDA-DLSz!—drawing).

**Figure 47:**
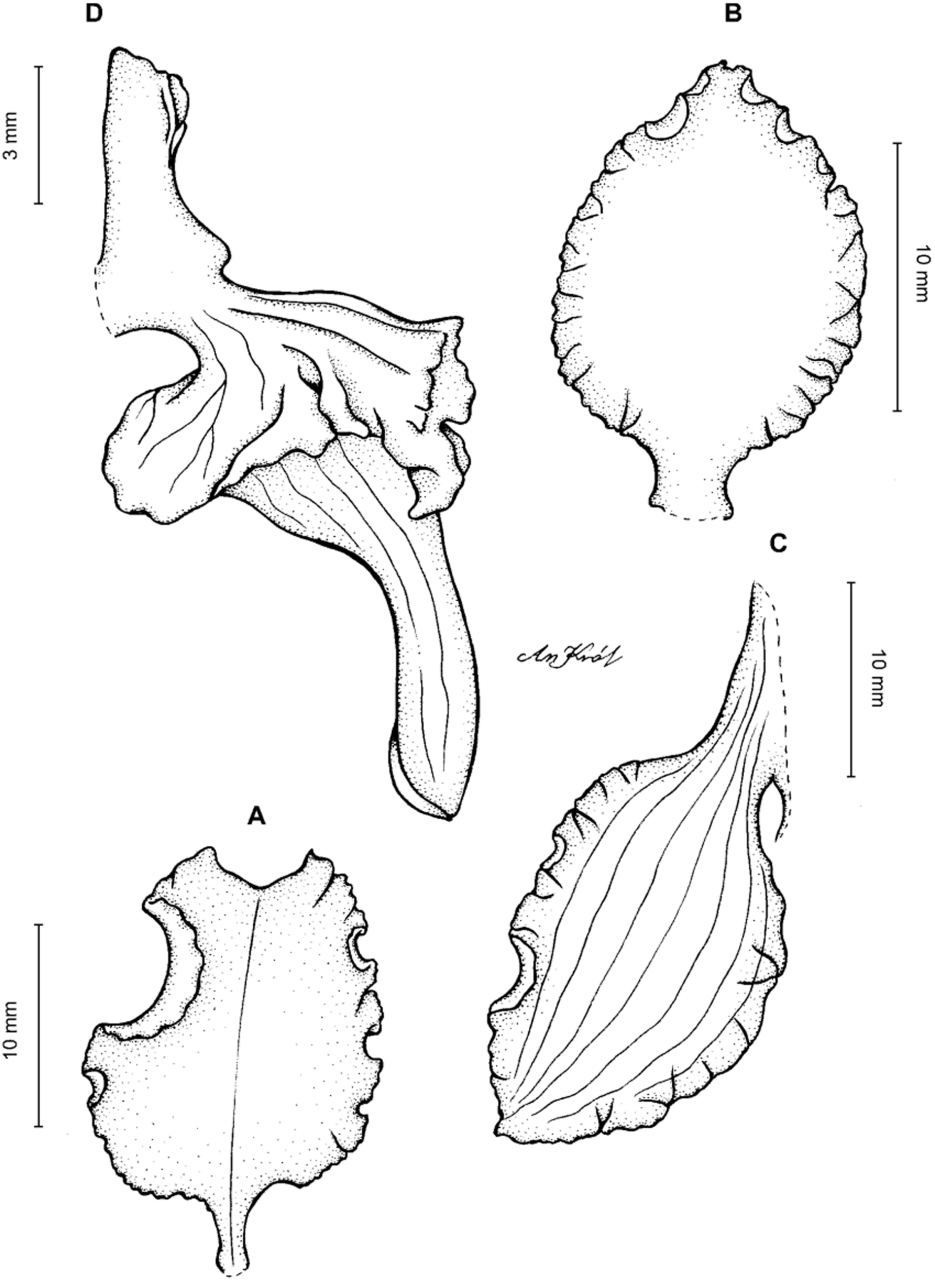
*Cyrtochilum insculptum* (Rchb.f.) Kraenzl. (A) Dorsal sepal; (B) Petal; (C) Lateral sepal; (D) Lip and gynostemium. Drawn by Anna Król from *Day s.n*. (W-R).

Pseudobulbs caespitose, surrounded basally by several foliaceous sheaths. Inflorescence 50–60 cm long, erect, then arching, paniculate with widely spaced, flexuous few- to many-flowered branches. Floral bract 6 mm long. Pedicel and ovary about 30 mm long. Dorsal sepal clawed; claw about 3 mm long; blade 17–18 mm long, 12–13 mm wide, ovate to elliptic-ovate, base rounded, apex obtuse, margin strongly undulate. Petals clawed; claw 2 mm long, wide; blade 15 mm long, 12–13 mm wide, ovate to elliptic, apex obtuse, margin undulate. Lateral sepals connate for basal 10 mm, free part shortly clawed; blade 24 mm long, 10 mm wide, obliquely ovate to oblong ovate, subacute, margin undulate. Lip about 12 mm long in total, base truncate, 3-lobed; basal part 3 mm long, 6–7 mm wide, transversely elliptic-obtriangular, apex of lateral lobes obtuse, with crenate margins; callus massive, occupying half of the lip length, in the form of central ridge with wrinkled apex, flanked by irregular outgrowths; apical part 7 mm long, 4 mm wide, ligulate, obtuse. Gynostemium 5 mm long, suberect, lateral appendages rudimentary, with prominent basal triangular outgrowths (Fig. 47).

*Ecology*: Epiphyte or terrestrial, growing in upper montane cloud forest.

*Distribution*: Ecuador. Alt. 2,400 m.

*Notes*: *Cyrtochilum insculptum* can be confused with *C. gyriferum* as in both of them rudimentary gynostemium appendages can be observed. The former however has wider tepals, obtuse dorsal sepal and petals (vs acuminate), and prominent triangular outgrowths on the gynostemium base (vs obscure, rounded).

*Representative specimens*: ECUADOR. **Loja**: Near Loja in mountains, 2,400 m, 10 July 1982, collected and cultivated in Cuenca by Dr. Benigno Malo, *C. Dodson & A. Embree 13172* (SEL—Dodson & Dodson, 1984). *Sine loc., Day s.n*. (W-R). *Sine loc. Sine coll*. (W-R 2.1882!).

***Cyrtochilum cinereobrunneum*** D.E. Benn. & Christenson, Icon. Orchid. Peruv.: pl. 622. 2001. TYPE: Peru. *Bennett 5976* (holotype, Herb. Bennettianum).

Rhizome, short, creeping. Pseudobulbs caespitose, 10–13 cm long, 3–4.5 cm wide, ovoid to ellipsoid-ovoid, surrounded basally by several foliaceous sheaths 26–30 cm long, 5.2–5.5 cm wide. Leaves 46–48 cm long, 2.4–3 cm wide, linear-oblanceolate, acute. Inflorescence 200–300 cm long, wiry, paniculate with widely spaced, flexuous few- to many-flowered branches. Dorsal sepal clawed; claw about 4–5 mm long, narrow, without any basal wings; blade 14–15 mm long, 10 mm wide, elliptic-ovate, base cuneate, apex acute, margin undulate. Petals clawed; claw 2 mm long, wide; blade 15 mm long, 9 mm wide, oblong ovate to elliptic, apex obtuse, margin crenate-undulate. Lateral sepals free to the base; claw 5 mm long, narrow, without any basal wings; blade 16–17 mm long, 9–10 mm wide, obliquely ovate to oblong ovate, subacute, margin undulate. Lip 12 mm long in total, base truncate, curved to expose callus; basal part 6 mm long, 5 mm wide, obovate-obtriangular, apex of lateral lobes obtuse; callus massive and very complicated, in the form of central, nose-like ridge, flanked by a pair of rhombic wings, and some additional outgrowths towards the base; apical part 5–6 mm long, 3–4 mm wide, ligulate-elliptic, rounded, shortly apiculate. Gynostemium 8 mm long, sigmoid, lateral appendages rudimentary.

*Ecology*: Epiphyte or terrestrial on cloud forest.

*Distribution*: Peru. Alt. 1,800 m.

*Notes*: This Peruvian species resembles *C. insculptum* described above, however, lateral sepals of *C. cinereobrunneum* are free to the base (vs basally connate for about 10 mm), all sepals are acute (vs dorsal sepal obtuse, lateral ones subacute), petals are very strongly undulate and distinctly twisted (vs undulate, not twisted), the lip main callus is flanked by very large, prominent wing-like projections (vs callus not flanked by such projections), lip apex smooth (vs wrinkled), sigmoid gynostemium (vs erect), tegula attenuate towards apex (vs tegula truncate at apex) and clavate (vs ellipsoid) pollinia.

*Representative specimen*: **PERU**. Sector Chipes near San Vicente mine, 1,800 m, *D.E. Bennett 5976* (Herb. Bennettianum).

***Cyrtochilum gyriferum*** Kraenzl., Notizbl. Bot. Gart. Berlin-Dahlem 7: 94.1917. TYPE: Ecuador. *Krause 6* (holotype, W). ≡ *Oncidium gyriferum* Rchb.f., Gard. Chron., n.s., 9: 558. 1878, *nom. nud*.

**Figure 48:**
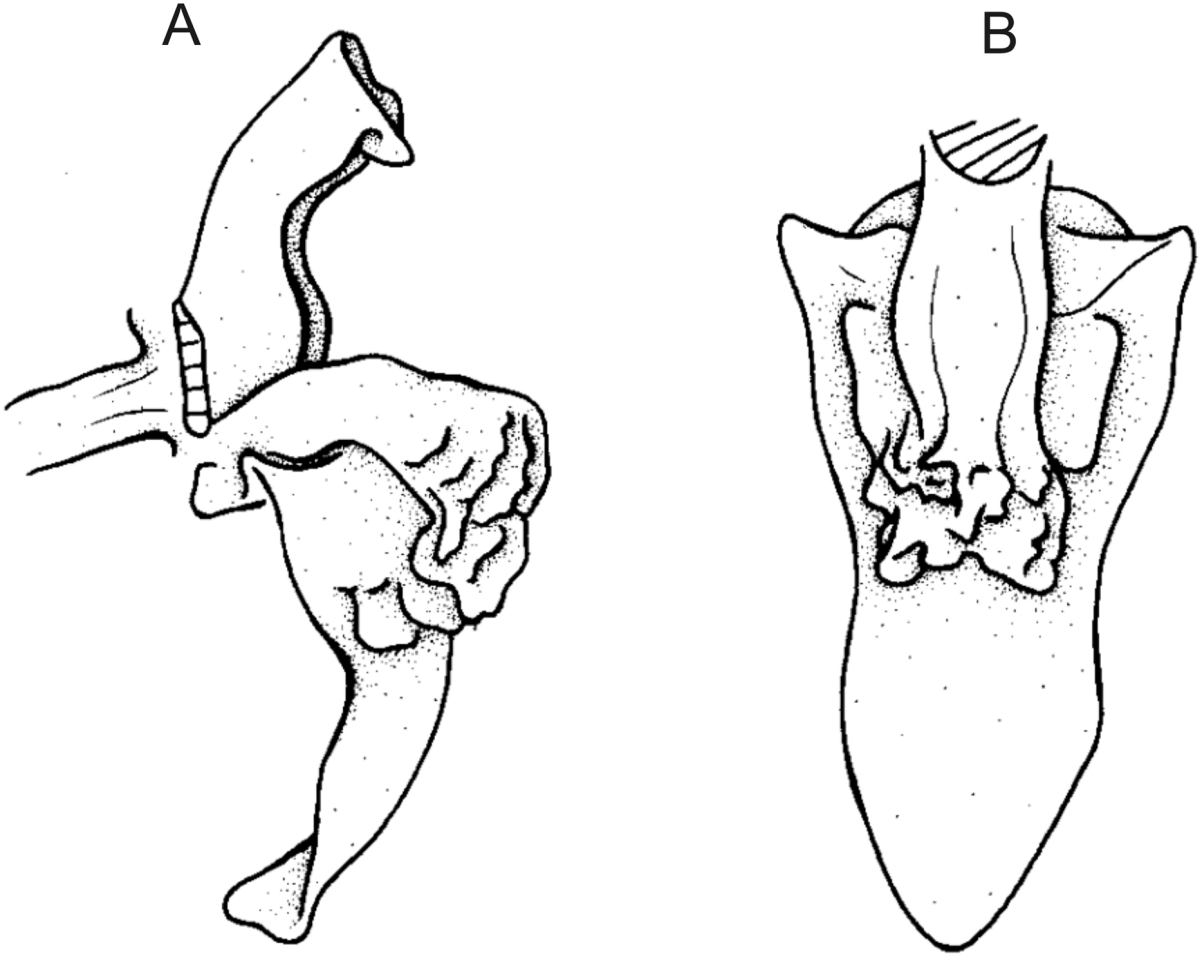
*Cyrtochilum gyriferum* Kraenzl. (A) Gynostemium and lip, side view; (B) Lip, front view. Redrawn by Natalia Olędrzyńska from Dalström (2010).

Pseudobulbs caespitose or distant on a creeping, bracteate rhizome, about 10 cm long, 4 cm wide, elongate, narrowly ovoid to oblong, surrounded basally by distichous sheaths, the uppermost foliaceous. Leaves 2–3, about 30–40 cm long, 2–3 cm wide, subpetiolate, elliptic to obovate, acuminate. Inflorescence up to 150 cm long, creeping-scandent, paniculate, branched widely spaced, almost straight to flexuous, loosely many-flowered. Flowers with dark brown tepals with yellow apices, pink to purple lip with white callus. Dorsal sepal clawed; claw 4 mm long, narrow, wingless; blade 16 mm long, 10 mm wide, elliptic or ovate, somewhat undulate, base cuneate, apex acuminate and recurved. Petals clawed; claw about 2 mm long, wingless; blade 16 mm long, 8–10 mm wide, ovate, base cuneate, apex acuminate, somewhat undulate. Lateral sepals clawed; claw about 5 mm long, connate together for about 4 mm, wingless; blade 20 mm long, 6–7 mm wide, basally oblique, elliptic to obovate, base cuneate, apex acuminate, margins undulate. Lip 13–15 mm long in total, 5–7 mm wide, cuneate, then hastate, 3-lobed with acute side lobes, middle lobe slightly elliptic, broadly ligulate, rounded to acute; callus fleshy, central, longitudinal, rounded ridge extending to the half of lamina, apex truncate, tuberoid, strongly wrinkled. Gynostemium about 8 mm long, suberect, lateral lobes missing, basal projections rounded, obscure (Fig. 48).

*Ecology*: Epiphytic in upper montane cloud forest.

*Distribution*: Ecuador, Peru (Zelenko & Bermúdez, 2009). Alt. 1,500–2,500 m.

*Notes*: *Cyrtochilum gyriferum* can be easily separated from *C. insculptum* by wider, obtuse tepals and the lack of prominent, triangular outgrowths at the gynostemium base. It can be equally easy to distinguished from *C. mandibulare* by the gynostemium appendages which have fimbriate lower margins.

*Representative specimens*: ECUADOR. **El Oro**: Curtincapa-Guagra Uma, km 8, 1,500 m, *J. Steyermark 53890* (AMES, RPSC—Dalström, 2010). **Loja**: *Krause 6* (W); Mt. Cajanuma, 12 km south of Loja, 2,500 m, 25 February 1982, collected and cultivated in Cuenca by Alfonso Pozo, *C. Dodson 12871* (SEL—Dodson & Dodson, 1984); Sozoranga S of Cariamanga, *C. Dodson & A. Embree 13290* (SEL—Dalström, 2010).

***Cyrtochilum mandibulare*** (Linden & Rchb.f.) Kraenzl., Notizbl. Bot. Gart. Berlin-Dahlem 7: 92. 1917. ≡ *Oncidium mandibulare* Linden & Rchb.f., Bonplandia (Hannover) 2: 279 (1854). TYPE: Colombia [New Granada]. *Schlim 1179* (holotype, W-R! 36968, UGDA-DLSz!—drawing).

**Figure 49:**
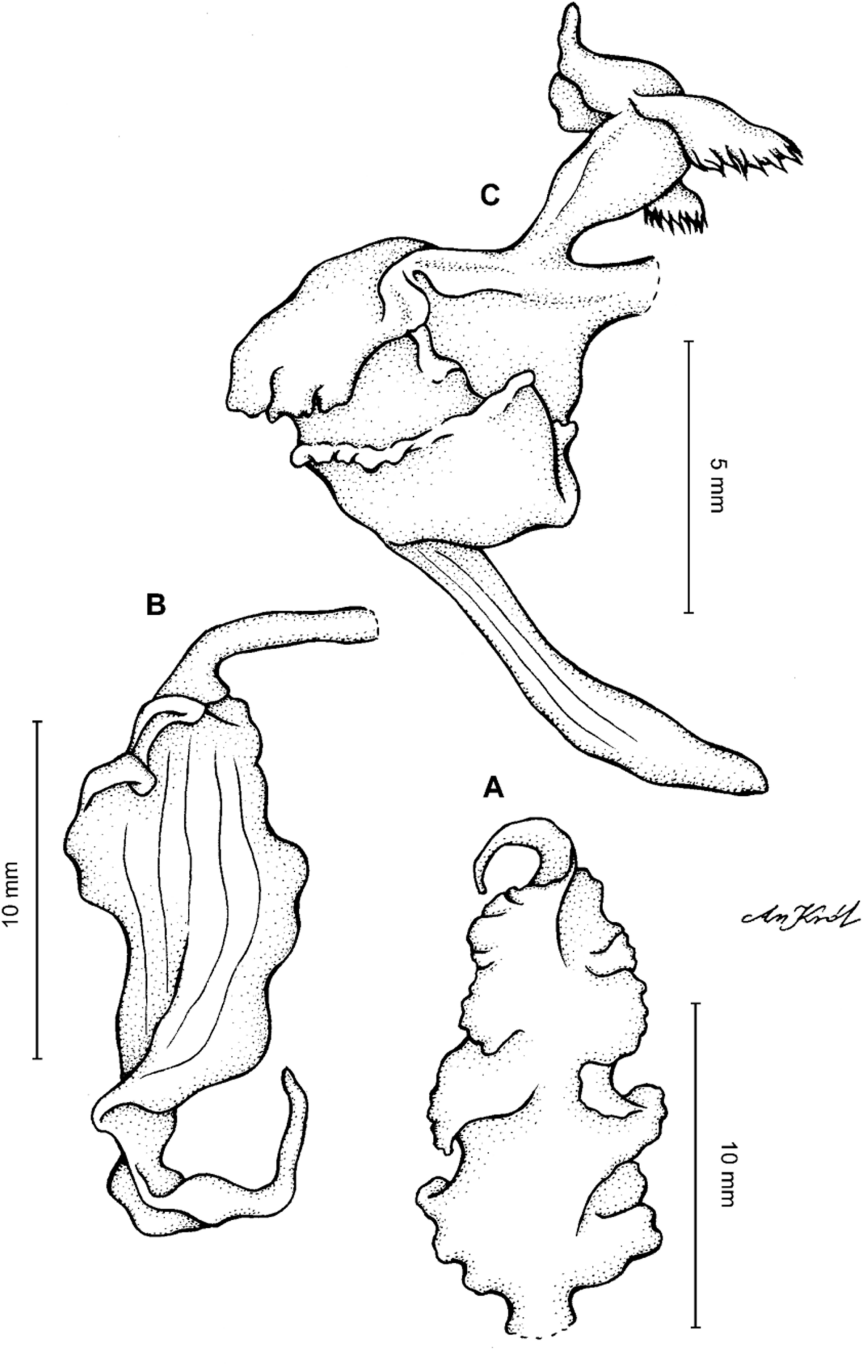
*Cyrtochilum mandibulare* (Linden & Rchb.f.) Kraenzl. (A) Dorsal sepal; (B) Lateral sepal; (C) Lip and gynostemium, side view. Drawn by Anna Król from *Schlim 1179* (W-R).

Vegetative parts unknown. Flowers large, conspicuous, showy. Floral bracts 20 mm long. Pedicel and ovary 35 mm long. Dorsal sepal not seen. Petals clawed; claw about 2 mm long, wide; blade 15 mm long, 8.5 mm wide when spread, somewhat oblique, oblong elliptic-ovate, base broadly cuneate, apex acuminate and strongly recurved, margins strongly irregularly undulate and crenate. Lateral sepals prominently clawed; claw 12 mm long, narrow; blade 31 mm long, 7 mm wide, obliquely lanceolate-ovate in general outline, basally cuneate, apex acuminate and strongly recurved, margins undulate, entire. Lip 20–21 mm long in total, curved in natural position exposing large callus; basal part 8–8.5 mm long, about 15 mm wide, broadly rhombic, convex in the centre; callus large, consisting of a flat, glabrous, pad-like tissue dissected into some lobules and some additional thickenings beyond the main callus; apical part 12 mm long, about 3 mm wide, linear-ligulate, attenuate towards obtuse apex. Gynostemium 4 mm long, sigmoid, connate basally with the lip, lateral appendages obliquely oblanceolate, with fimbriate lower margins, not reaching the anther base (Fig. 49).

*Ecology*: No data.

*Distribution*: Colombia.

*Notes*: The unique character of this species is shape of the lateral appendages of gynostemium which are obliquely oblanceolate, with fimbriate lower margins. Additionally, the lip callus is very peculiar and consists of large, flat, glabrous, pad-like tissue dissected into some lobules and some additional thickenings beyond the main callus.

Dalström (2001) considered this species as conspecific with *C. divaricatum* whose basal lip partis transversely elliptic in outline (vs broadly rhombic in *C. mandibulare*), apical part is oblong-obtriangular, subacute (vs linear-ligulate) and its gynostemium appendages have irregular lower margin (vs lower margin fimbriate).

*Representative specimen*: COLOMBIA. [New Granada], *L. Schlim 1179* (W-R!).

***Cyrtochilum aciculatum*** Königer, Arcula 3: 58. 1995. TYPE: Peru. *Königer WK 37* (holotype, M; isotypes, K, USM, herb. Königer).

Rhizome creeping, internodes 6–8 cm long, about 2 cm in diameter, concealed by numerous, large, imbricating, foliaceous sheaths. Pseudobulbs up to 12 cm long, 6 cm wide, narrowly ovoid, subcompressed, wrinkled-furrowed, 2-leaved apically. Leaves up to 50 cm long, 2.8 cm wide, linear-lanceolate, acuminate apically, complicate towards the base, pseudobulbs on both sides with 4–5 large, articulate. Inflorescence 200–300 cm long, arising from the axil of a sheath, twining, stout, with few bracteoles, paniculate, racemose above, with distant, 4–6-f1owered branches 8–17 cm long. Flowers medium-sized, tepals yellowish, densely covered with very fine irregular brown streaks, petals with few additional yellow spots, lateral lobes of the lip yellow, marked with brown, the tip yellow, callus yellow, dotted and marked with brown. Floral bracts 15 mm long, amplexicaul, acuminate. Pedicellate ovary about 40 mm long. Dorsal sepal clawed; claw about 4 mm long, basally auriculate, canaliculate on the inner side; blade 16 mm long and 12 mm wide, ovate, acute, fairly flat, undulate at the margins, the midvein thickened dorsally. Petals clawed; claw about 4 mm long, broadly margined to auriculate; blade 14 mm long and 9 mm wide, ovate, subacuminate, strongly undulate at the margins. Lateral sepals clawed; claw about 6 mm long, semiterete, canaliculate on the inner side, margined on both sides, on one side auriculate at the base; blade 20 mm long, 12 mm wide, obliquely obovate, acute, fairly flat, undulate at the margins, becoming obliquely cuneate towards the claw, the midvein thickened dorsally. Lip 18 mm long in total, sessile, oblong; basal part about 8–9 mm long, 4 mm wide, lateral lobes broadly triangular, deflexed, rigid, with a transverse fold, the posterior margins touching below the lip; callus at the base of the lip elevated, the midline lamella very large, subcurvate apically, truncate and subconcave in front, laterally in the lower half on both sides with a spreading lamella, in the anterior half on both sides many gibbous projections, both processes in front large, tooth-like, extrorsely directed, in the middle on both sides with a remote hump, the callus highly connate with the column at the base; apical part narrowly oblong-elliptical, acute. Gynostemium about 8 mm long, erect, wings conspicuous, obliquely obtriangular, thick.

*Ecology*: Epiphyte.

*Distribution*: Peru.

*Notes*: *Cyrtochilum aciculatum* is characterized by a prominent, large lip callus combined with conspicuous, obliquely obtriangular gynostemium appendages. In the similar *C. falcipetalum* the gynostemium appendages are oblong, obliquely truncate and crenate along lower part. Somewhat similar to *C. aciculatum* is *C. crispatissimum*, which however have strongly undulate sepals and petals with cuneate base and short, apically bifid gynostemium lateral appendages.

Dalström (2001) considered this species as conspecific with *C. gargantua* but the lip of *C. gargantua* is deeply hastate in general outline, with prominent lateral lobes.

*Representative specimen*: PERU. *Sine loc., W. Königer WK 37* (K, M, USM, herb. Königer).

***Cyrtochilum crispatissimum*** Kraenzl., Pflanzenr. (Engler) IV. 50: 38 (Fig. 9). 1922. TYPE: Bolivia. *Pearce 810* (holotype, W-R! 1450, UGDA-DLSz!—drawing).

**Figure 50:**
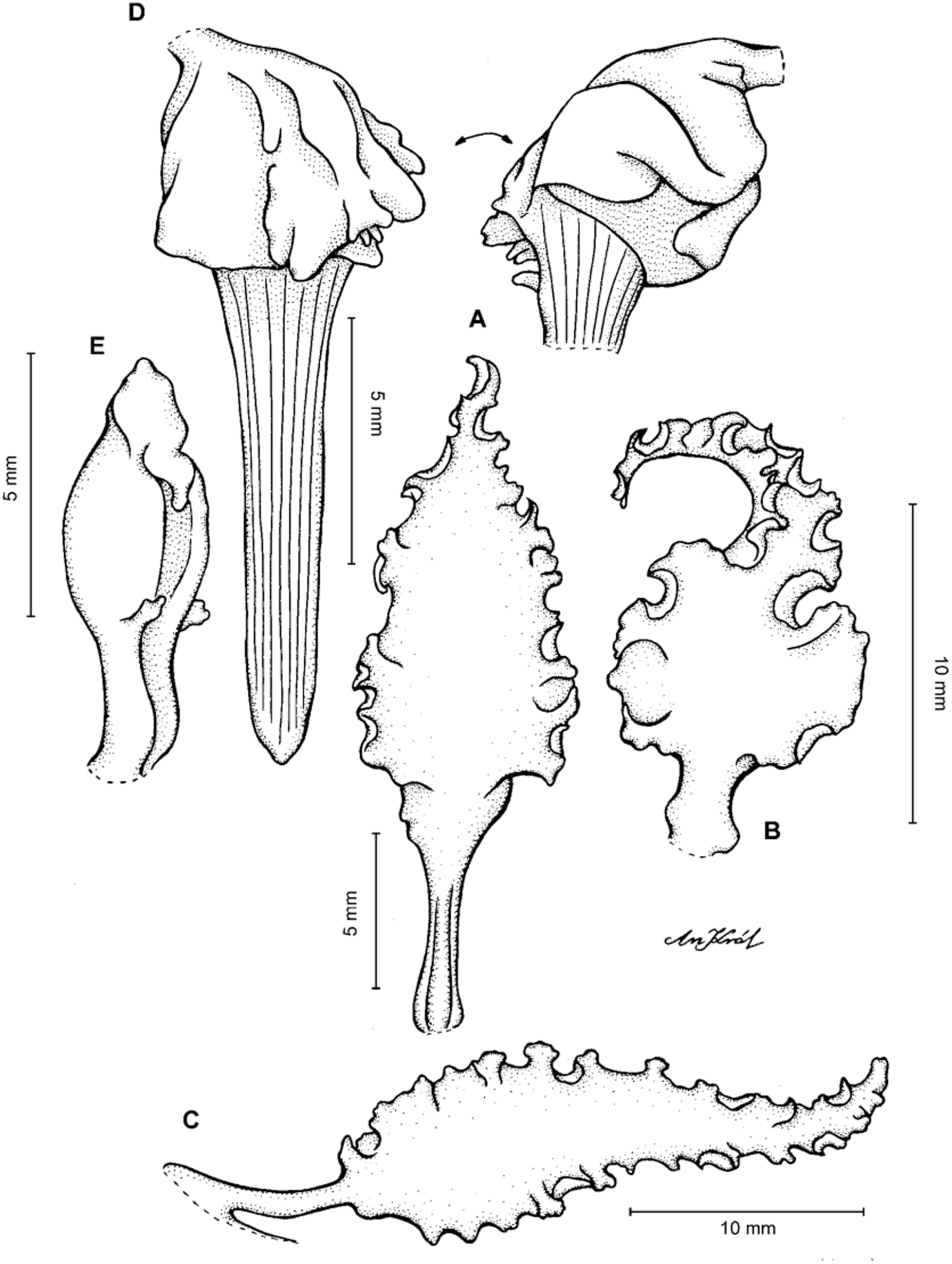
*Cyrtochilum crispatissimum* Kraenzl. (A) Dorsal sepal; (B) Petal; (C) Lateral sepal; (D) Lip; (E) Gynostemium. Drawn by Anna Król from *Pearce 810* (W-R).

Pseudobulbs caespitose or distant on a creeping, bracteate rhizome, about 10 cm long, 4 cm wide, elongate, narrowly ovoid to oblong, surrounded basally by distichous sheaths, the uppermost foliaceous. Leaves 2–3, about 30–40 cm long, 2–3 cm wide, subpetiolate, elliptic to obovate, acuminate. Inflorescence up to 150 cm long, creeping-scandent, paniculate, branches widely spaced, almost straight to flexuous, loosely many-flowered. Floral bracts 9 mm long. Pedicellate ovary 25 mm long. Dorsal sepal clawed; claw 6–7 mm long, narrow, wingless; blade 16 mm long, 9 mm wide, elliptic-lanceolate or ovate-lanceolate, strongly undulate, base cuneate, apex acuminate and recurved. Petals clawed; claw about 3 mm long, wingless; blade 16–17 mm long, 8 mm wide, ovate-lanceolate, strongly falcate and undulate, base broadly cuneate, apex acuminate. Lateral sepals clawed; claw about 6–7 mm long, connate together for about 3–4 mm, wingless; blade 28 mm long, 6 mm wide, basally oblique, lanceolate, base cuneate, apex acuminate, margins strongly undulate. Lip 16 mm long in total, cuneate, then hastate, 3-lobed; basal part about 6 mm long, 5–7 mm wide, with acute side lobes; callus a fleshy, central, longitudinal, rounded ridge extending to the third of lamina, apex truncate, flanked by additional keels; apical part 10 mm long, 2 mm wide, linear, obtuse. Gynostemium about 8 mm long, somewhat arcuate, lateral appendages short, apically bifid (Fig. 50).

*Ecology*: No data.

*Distribution*: Bolivia.

*Notes*: This species can be distinguished by very narrow lateral sepals (ca. 5 times longer than wide), strongly undulate along margins with acuminate apex, combined with obscure, apically bifid gynostemium appendages. Petals are strongly undulate as well and falcate being very difficult to flattened.

*Representative specimen*: BOLIVIA. *G. Pearce 810* (W-R!).

***Cyrtochilum lehmannii*** Szlach. & Kolan., ***sp. nov***. TYPE: Colombia. *Lehmann BT127* (holotype, AMES!, UGDA-DLSz!—fragment, drawing, foto).

Diagnosis: This s*pecies resembling C. cuencanum in its gynostemium architecture. The two taxa differ in the lip callus form which in C. lehmannii is apically much elongate and free forming a kind of nose-like, truncate projection, flanked by additional, uneven keels*.

**Figure 51:**
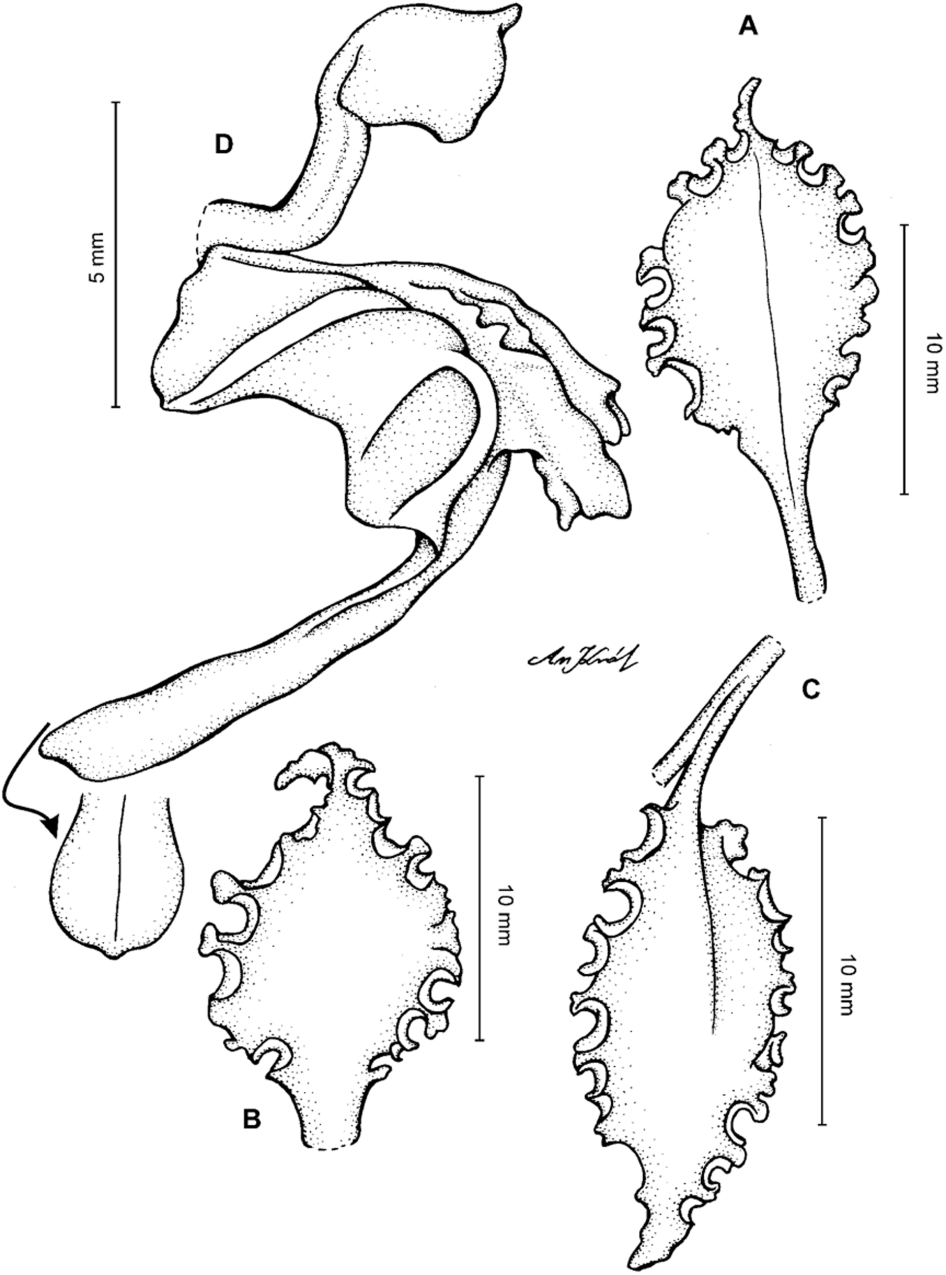
*Cyrtochilum lehmannii* Szlach. & Kolan. (A) Dorsal sepal; (B) Petal; (C) Lateral sepal; (D) Lip and gynostemium, side view. Drawn by Anna Król from *Lehmann BT 127* (AMES).

Leaves up to 40 cm long and 3.5 cm wide, linear-oblanceolate, acute. Inflorescence scandent, paniculate, branching, branches loosely 4–5-flowered. Floral bracts 15 mm long. Pedicellate ovary 50 mm long. Dorsal sepal clawed; claw 5 mm long, narrow, wingless; blade 15 mm long, 9 mm wide, elliptic, strongly undulate, base cuneate, apex acuminate and recurved. Petals clawed; claw about 2 mm long, wingless; blade 15 mm long, 8 mm wide, ovate-elliptic, falcate and strongly undulate, base cuneate, apex acuminate and recurved. Lateral sepals clawed; claw 10 mm long, connate together for about 5 mm, wingless; blade 15 mm long, 8 mm wide, oblique, ovate-lanceolate, base cuneate, apex acuminate, margins strongly undulate. Lip 16 mm long in total, truncate at base, obscurely 3-lobed; basal part about 8 mm long, 4–6 mm wide, pandurate in outline, margins rounded; callus a fleshy, central, longitudinal, rounded ridge extending to the middle of lamina, apex nose-like, truncate, flanked by additional, uneven keels; apical part 8 mm long, 2.6 mm wide, linear-ligulate, obtuse. Gynostemium about 6 mm long, sigmoid, lateral appendages missing (Fig. 51).

*Etymology*: In honour of the collector of the type specimen.

*Ecology*: No data.

*Distribution*: Colombia. Alt. 2,700–2,900 m.

*Notes*: By the general gynostemium architecture the new entity is similar to *C. cuencanum*. In both species the gynostemium is strongly sigmoid, connate with the lip along its basal fifth or quarter. The lip callus in both, however, is completely different. In *C. lehmannii* it is apically much elongate and free forming a kind of nose-like, truncate projection, flanked by additional, uneven keels. In *C. cuencanum* the lip callus is massive, consisting of central ridge, more or less wrinkled apically, flanked by lower keel on each side, with wrinkled upper surface. Moreover, flowers of *C. cuencanum* are larger than in *C. lehmannii*, with claw of lateral sepals being ca. 3 times shorter than blade, and basal lip part wider than long.

*Representative specimen*: COLOMBIA. Vulcan Galeras[?], 2,700–2,900 m, *F.C. Lehmann BT127* (AMES!, UGDA-DLSz!—fragment, drawing, foto).

*Conservation status*: This species is described based on material collected in the XIX century. We cannot assess the current threats for this taxon hereby the “data deficient” (DD) category should be applied according to IUCN (2001).

**(5) *Zebrinum*-group**

Lip narrow, basal part obscurely deltoid, apical part attenuate towards acute to obtuse apex, callus palmate or a bunch of digitate projections, the median keel usually elevated.

The group embraces about 14 mostly Colombian-Ecuadorian species.

### Key to the species

1 Lip callus papillate2
1* Lip callus glabrous3
2 Gynostemium lateral appendages obliquely oblanceolate, entire*C. lucescens*
2* Gynostemium lateral appendages wing-like or obtriangular, apically dissected into 3–4, unequal lobes*C. biscellum*
3 Blade of dorsal sepal as long as wide or wider than long …4
3* Blade of dorsal sepal longer than wide7
4 Base of dorsal sepal truncate or cordate5
4* Base of dorsal sepal cuneate6
5 Gynostemium lateral appendages obliquely obtriangular*C. andreettae*
5* Gynostemium lateral appendages lanceolate, falcate and upcurved*C. renisepalum*
6 Lip callus consisting of two ridges in the basal part, with shallow groove between them, then divided into some, irregular, thickenings, additional thickenings being observed along the bending line of basal wings, gynostemium lateral appendages obliquely oblong-triangular, falcate, directed forwards*C. guariniae*
6* Lip callus very complicated, apically dissected into 12 lobules, more or less wrinkled, base with entire margins, thick, gynostemium basally with chin-like projections, lateral appendages transformed into wings flanking gynostemium*C. brachypterum*
7 Lip callus large, prominent, occupying basal half of the lip8
7* Lip callus small, confined to the basal third of the lip11
8 Lip callus massive, dissected into numerous lobes of various forms and sizes radiating from the centre*C. zebrinum*
8* Lip callus not as above9
9 Gynostemium lateral appendages digitate*C. carderi*
9* Gynostemium appendages with truncate apex10
10 Lip callus high, apically on both sides with a tall, narrow, obliquely stretched forward lamella 2-apiculate apically, below with a few, low, longitudinal, variable gibbous lamellae, above with 2 obliquely spreading, approximate teeth, in the middle with a large, triangular tooth, thereafter on both sides with a small, low, horizontally spreading, rounded lamella*C. cumandae*
10* Lip callus consisting of central longitudinal, fleshy, faintly 3-partite, low keel, turning abruptly into a raised, butterfly-shaped, spreading, lobulate and angulate structure, basally flanked by low, fleshy, spreading ridge*C. flavostellulare*
11 Lip base truncate12
11* Lip base cordate13
12 Lip callus consisting of a prominent, median, nose-like keel, flanked by obliquely rhombic wings and several, irregular additional thickenings beyond the main callus*C. tenense*
12* Lip callus forming rather narrow pad, with rather uneven, irregular surface, additional thickenings appearing along the line of curvation of basal lip lobes*C. microcallosum*
13 Claws of lateral sepals connate in the basal half*C. microphysium*
13* Claws of lateral sepals free*C. christensonianum*

***Cyrtochilum carderi*** (Rchb.f.) Kraenzl., Notizbl. Bot. Gart. Berlin-Dahlem 7: 94. 1917. ≡ *Oncidium carderi* Rchb.f., Gard. Chron., n.s., 3: 748. 1875. TYPE: Colombia [New Grenada]. *Carder s.n*. (holotype, W-R! 36997; UGDA-DLSz!—drawing).

**Figure 52:**
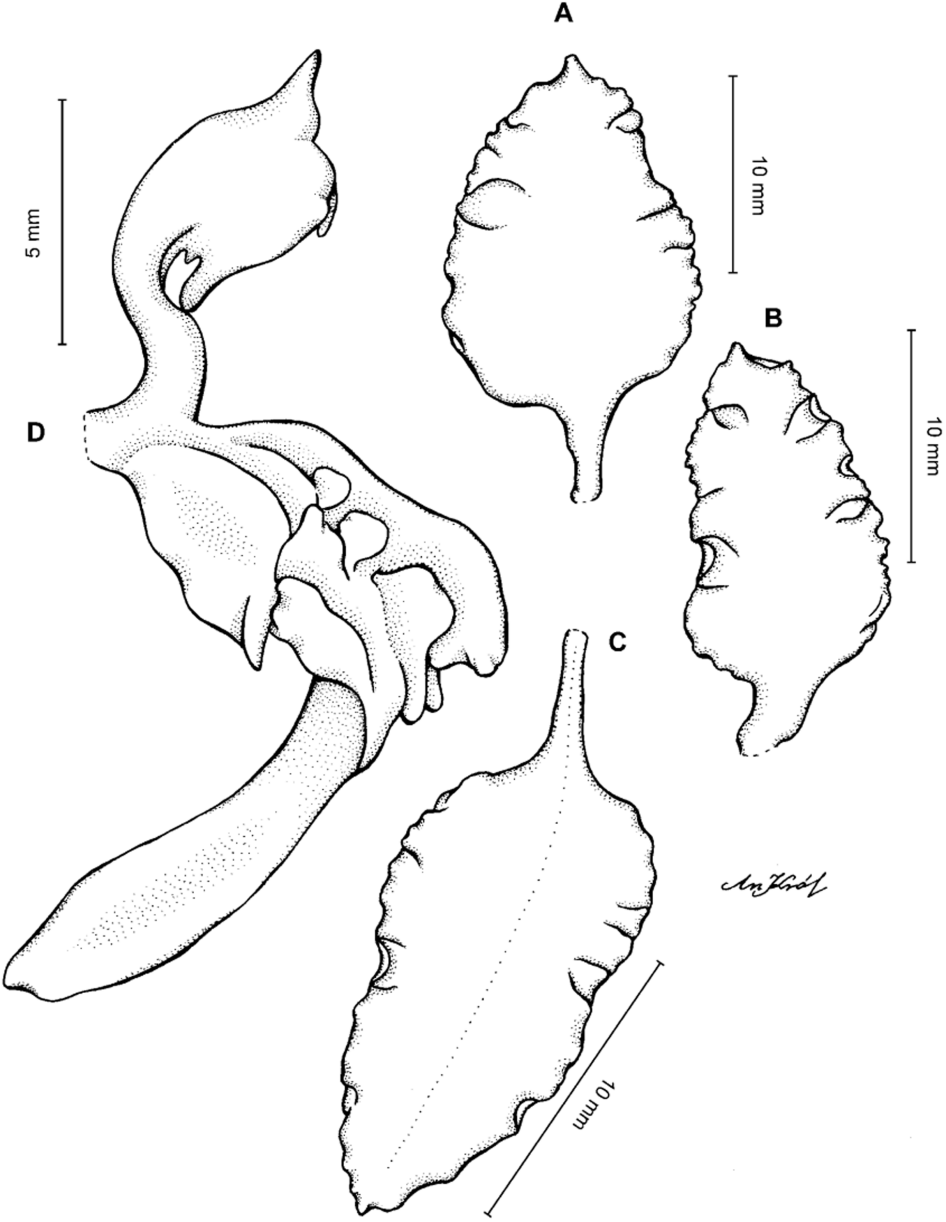
*Cyrtochilum carderi* (Rchb.f.) Kraenzl. (A) Dorsal sepal; (B) Petal; (C) Lateral sepal; (D) Gynostemium and lip, side view. Drawn by Anna Król from *Lehmann 8566* (AMES).

**Figure 53:**
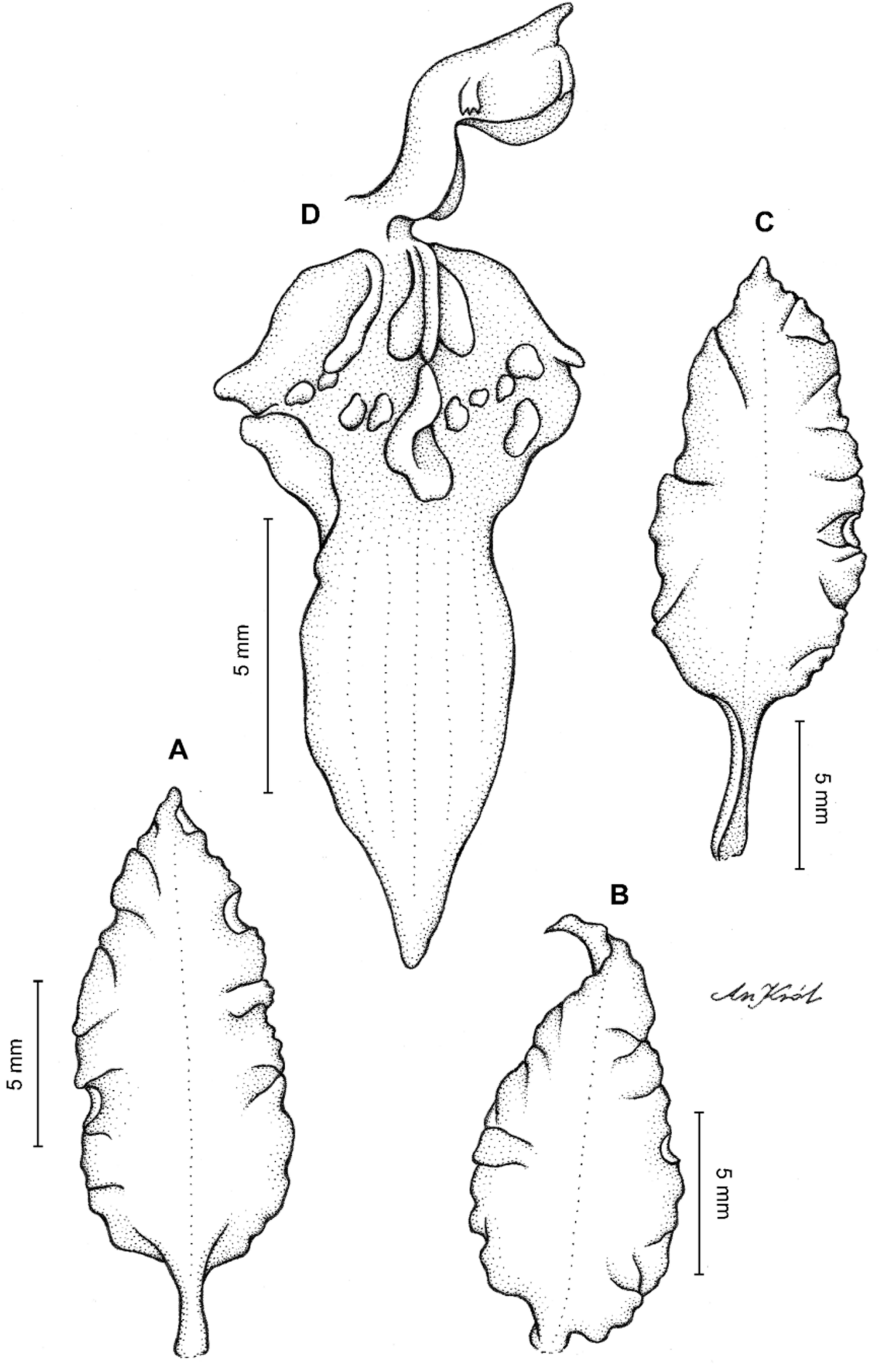
*Cyrtochilum carderi* (Rchb.f.) Kraenzl. (A) Dorsal sepal; (B) Petal; (C) Lateral sepal; (D) Gynostemium and lip, side view. Drawn by Anna Król from *Steyermark s.n*. (AMES).

Pseudobulbs up to 10 cm long and 2.5 cm wide, oblong, somewhat compressed, bifoliate. Leaves up to 45 cm long and 5.5 cm wide, oblanceolate, acute. Inflorescence up to 3 m long, climbing or pendent. Flowers medium-sized, conspicuous, lip deep purple, lateral petals purple within, brown without, sepals chocolate-brown both sides, column dark purple in upper half, brown-black in lower half. Floral bracts 6–11 mm long. Pedicel and ovary 18–29 mm long. Dorsal sepal clawed; claw 4–6 mm long, narrow, canaliculate, wingless; blade 13–17 mm long, 8–12 mm wide, elliptic, margins entire, somewhat undulate, base broadly cuneate, apex obtuse. Petals clawed; claw about 2 mm long; blade 12–14 mm long, 6–9.5 mm wide when spread, obliquely ovate, more or less recurved, base cuneate, apex obtuse, margins entire, undulate. Lateral sepals prominently clawed; claw 4–6 mm long, narrow, wingless; blade 15–19 mm long, 10–12.5 mm wide, obliquely elliptic-ovate in general outline, base cuneate, apex subobtuse to acute, margins entire, slightly undulate. Lip 12 mm long in total, gently curved in natural position; basal part 5 mm long, 7–8 mm wide, transversely elliptic in general outline, base cuneate, callus glabrous, very complicated, spread all over the basal part of the lip, consisting of a prominent, median ridge, flanked by simple or 2-lobed keels on each side, with numerous, additional outgrowths of various forms and sizes beyond the main callus; apical part 5–7 mm long, 4 mm wide, ligulate, blunt at apex. Gynostemium 6–8 mm long, sigmoid, much swollen at the apex, connate basally with the lip, lateral appendages digitate, rudimentary (Figs. 52 and 53).

*Ecology*: Terrestrial or epiphytic on roadbank, in disturbed wet montane vegetation and cloud forest.

*Distribution*: Colombia, Venezuela. Alt. 1,700–3,100 m.

*Notes*: The most distinguishing character of this species is lip callus. From the similar species, *C. cumandae* and *C. flavostellulare*, *C. carderi* differs in having digitate lateral appendages of gynostemium (vs appendages with truncate apex).

Dalström (2001) considered this species as conspecific with *C. ventilabrum*, but unlike the latter it has a glabrous lip callus.

*Representative specimens*: COLOMBIA. **Antioquia**: Mpio. Frontino. Km 12 of road Nutibara-Murri. Disturbed wet/very wet montane vegetation, roadside, 6°45′N 76°22′W, 2,010 m, 23 September 1987, *J.L. Zarucchi, A.E. Brant & C.J. Castaño 5701* (MO!, UGDA-DLSz!—drawing); Rio Negro, 2,300 m, *G. Wallis s.n*. (W-R! 36938, UGDA-DLSz!—drawing); La Ceja. Cerro San Jose, 2,600 m, 16 November 2002, *M. Ospina H. 1572* (COL!, UGDA-DLSz!—drawing); *Sine loc.*, 21 August 1915, *W. Kalbreyer 1749* (AMES!, UGDA-DLSz!—drawing); Rio Medellin, 2,450 m, *M. Ospina H. 163* (AMES!, UGDA-DLSz!—fragment, drawing, photo). **Cauca**: Popayán, 1,800–2,400 m, *F.C. Lehmann 6758* (AMES!, UGDA-DLSz!—fragment, drawing, foto); Popayán, 1,800–2,400 m, *F.C. Lehmann 8439* (AMES!, UGDA-DLSz!—drawing); Waldem am Pasto, 2,000–3,000 m, January 1892, *F.C. Lehmann 7293* (AMES!, UGDA-DLSz!—fragment, drawing, foto); Cordillera Central, vertiente occidental, entre Popayán y Puracé, Quebrada de Aguarregada, 2,050–2,100 m, 30 December 1942, *J. Cuatrecasas 13773* (AMES!, UGDA-DLSz!—drawing). **Chocó**: Cordillera Occidental. Almansa, entre Bolivar (Antioquia) y Carmen de Atrato (Chocó), cerca del limite con Antioquia, 2,150 m, 30 July 1944, *H. Garcia-Barriga 11154* (COL!, US!, UGDA-DLSz!—drawing). [New Grenada]. **Cundinamarca**: Epiphytisch mit windender Inflorezenzachse, im Nebelwald (Bergwald) oberhalb Monterredondo, zwischen Bogotá und Villavicenio, 1,900 m, 2 December 1955, *St. Vogel 0134* (AMES!, UGDA-DLSz!—fragment, drawing, foto). **Magdalena**: Cerro Pintado, Sierra Perija, 3,100 m, 3–6 Jul. 1942, *M. Carriker 51* (US!, UGDA-DLSz!—drawing). **Nariño**: Near Mayasquer, edge of cliff above stream, 2,600 m, 2 August 1935, *Y. Mexia 7567* (AMES!, UGDA-DLSz!—fragment, drawing, foto). **Tolima**: Cordillera Central. Cano del Rio Anaime. Hacienda Santa Rita, 1,800–1,900 m, 25–26 December 1973, *H. Garcia-Barriga 20393* (GH *ex* COL!, US!, UGDA-DLSz!—fragment, drawing, foto). *Sine loc., J. Carder s.n*. (W-R! 36997; UGDA-DLSz!—drawing). *Sine loc., J. Triana 613* (US!, UGDA-DLSz!—drawing). Vulcan de Cumbal [?], 1,700–2,200 m, July and Aug, *F.C. Lehmann 8566* (AMES!, UGDA-DLSz!—drawing). VENEZUELA. **Tachira**: Laderas de selva nublada a lado de un riachuelo pedregoso, entre Michelena y Boca de Monte, oease te Zumbador, 1,700 m, 28 August 1966, *J.A. Steyermark & M. Rabe 96739* (AMES!, UGDA-DLSz!—drawing). *J.A. Steyermark s.n*. (AMES!, UGDA-DLSz!—fragment, drawing, photo).

***Cyrtochilum cumandae*** Königer, Arcula 4: 93. Königer (1995). TYPE: Ecuador. *Pozo & Königer WK-49* (holotype, M; isotypes: K, QCA, herb. Königer).

Plant epiphytic, erect, large for the genus; rhizome short; pseudobulbs narrowly ovoid, up to 11 cm high and 4 cm wide, smooth to subrugose, 2–3-leaved apically, with several, large, articulate, blade-bearing sheaths similar to the leaves on both sides. Leaves up to 54 cm long, 4 cm wide, linear-lanceolate, acute apically, complicate towards the base. Inflorescence 3–4 m long, arising from the axil of a sheath, twining, stout, paniculate, with numerous (up to 14), up to 5-flowered branches, racemose above. Flowers showy, dorsal sepal golden-yellow, densely, irregularly and broadly fasciate with chestnut-brown, lateral sepals similar in colour, the stripes confluent for the most part, petals golden-yellow, the basal half with numerous, irregular, chestnut-brown spots, basal half of the lip golden-yellow, anterior half of the lip brown, callus white. Floral bract 15 mm long, boat-shaped. Pedicel and ovary about 40 mm long. Dorsal sepal clawed; claw about 8 mm long, subcanaliculate within, on both sides margined, basally on both sides minutely auriculate; blade 28 mm long, 20 mm wide, broadly ovate, obtuse, fairly flat, subundulate at the margins, recurved apically. Petals spreading, clawed; claw about 5 mm long, on both sides margined; blade 22 mm long, 18 mm wide, ovate to elliptic-ovate, acute, strongly undulate marginally, the apex recurved. Lateral sepals spreading, divaricate, basally connate for 2 mm, clawed; claw about 10 mm long, subcanaliculate within, on both sides narrowly margined; blade 23 mm long, 16 mm wide, elliptic-ovate, rounded to obtuse-acute apically, strongly undulate marginally. Lip about 23 mm long in total, fleshy, rigid, the basal half spreading, more or less 5-angled; basal part 11 mm wide, lateral lobes more or less quadrate, descending, on both sides in front of them with a sharp-angled, descending, triangular, narrower lobe; callus glabrous, high, apically on both sides with a tall, narrow, obliquely stretched forward lamella 2-apiculate apically, below with a low, longitudinal, variably gibbous lamellae, above with 2 obliquely spreading, approximate teeth, in the middle with a large, triangular tooth, thereafter on both sides with a small, low, horizontally spreading, rounded lamella, the callus passing over into the column through a semiterete torus, on both sides of it with a distinct, longitudinal tumour. Gynostemium apical part about 12 mm long with wings almost square, obliquely stretched forward.

*Ecology*: Epiphyte.

*Distribution*: Ecuador. Alt. 2,800–3,000 m.

*Notes*: This species appears to be similar to *C. andreettae*, but has a larger lip callus and square gynostemium wings. Unlike in *C. cumandae* the lip callus of *C. flavostellulare* consists of central, longitudinal, low, faintly 3-partite keel that turns abruptly into a raised, butterfly-shaped, spreading, lobulate and angulate structure.

*Representative specimen*: ECUADOR. **Azuay**: El Arenal, 2,800–3,000 m, collected, cultivated and flowered by *A. Pozo & W. Königer WK-49* (K, M, QCA, herb. Königer).

***Cyrtochilum andreettae*** Königer & J. Portilla, Arcula 4: 90. 1995. TYPE: Ecuador. *Königer WK-48* (holotype, M; isotypes: K, QCA).

Rhizome elongate, creeping. Pseudobulbs 12 cm long, 4 cm in diameter, narrowly ovoid, transversely elliptic in cross section, smooth, supported basally by few leafy bracts. Leaves 2, up to 60 cm long and 5 cm wide, oblanceolate, acute. Inflorescence 2–4 m long, paniculate, wiry, with widely spaced few-flowered branches. Flowers large, conspicuous, showy, sepals dark brown, petals dark brown basally yellow, light brown apically with a yellow apex, lip brown with a yellow, brown-marked callus. Floral bracts 12 mm long. Pedicel and ovary about 30–45 mm long. Dorsal sepal shortly clawed; claw 5 mm long, rather narrow, canaliculate, basally winged; blade 25 mm long and wide, broadly ovate, base truncate, apex rounded, margins slightly undulate. Petals clawed; claw about 3 mm long, basally somewhat winged; blade 23 mm long, 20 mm wide when spread, obliquely cordate-ovate, acute, base truncate, margins irregularly and slightly undulate. Lateral sepals prominently clawed; claw 6–7 mm long, narrow, wingless, basally connate together; blade 36 mm long, 25 mm wide, obliquely elliptic-ovate in general outline, basally truncate, subacute at the apex, margins entire, very slightly undulate. Lip 20 mm long in total, curved in natural position exposing massive callus; basal part about 10 mm long, 8 mm wide, triangular-deltoid, base truncate, convex along the centre; callus very complicated consisting of tumoroid 3-dentate keel, the median tooth longer and irregularly angulate, flanked by longitudinal, dentate ridges; apical part 10 mm long, about 4 mm wide, ligulate, attenuate towards subacute apex. Gynostemium 6 mm long, sigmoid, apically swollen, connate basally with the lip, lateral appendages obliquely obtriangular, not reaching the anther base.

*Ecology*: Epiphyte.

*Distribution*: Ecuador. Alt. 1,400 m.

*Notes*: As stated above this species can be misidentified with *C. cumandae*, but has a smaller lip callus and obliquely obtriangular gynostemium appendages. Both species have differently colored flowers. In both they are showy, but in *C. cumandae* the sepals are golden-yellow, densely, irregularly and broadly fasciate with chestnut-brown (vs sepals dark brown), petals are golden-yellow, with numerous, irregular, chestnut-brown spots in the basal half (vs petals dark brown basally yellow, light brown apically with a yellow apex), basal half of the lip is golden-yellow, anterior half of the lip brown, callus white (vs lip brown with a yellow, brown-marked callus). The dorsal sepal of *C. andreettae* is as long as wide, and in *C. cumandae* it is longer than wide. Cyrtochilum *andreettae* differs from the similar *C. renisepalum* in the gynostemium appendages form (obliquely obtriangular in *C. andreettae*, lanceolate, falcate and upcurved in *C. renisepalum*). Furthermore the blade of dorsal sepal of *C. renisepalum* is wider than long, reniform with subcordate base, margins of both dorsal sepal and petals are strongly undulate and basal lip part is almost quadrate.

*Representative specimens*: ECUADOR**. Tungurahua**: *Sine loc., F. Kuhn s.n*. (SEL—Dalström, 2010). **Zamora-Chinchipe**: Cantón Yanzatza: E. Zarza. Alt. 1,400 m. Gesammelt von J. Portilla in Kultur in Blüte in München bei (word missing?, who collected at whoms place?), *W. Königer WK-48* (M, K, QCA, Herb. H. Königer).

***Cyrtochilum flavostellulare*** Dalström, Fl. Ecuador 87: 80. 2010. TYPE: Ecuador. *Steyermark 54159* (holotype, F).

**Figure 54:**
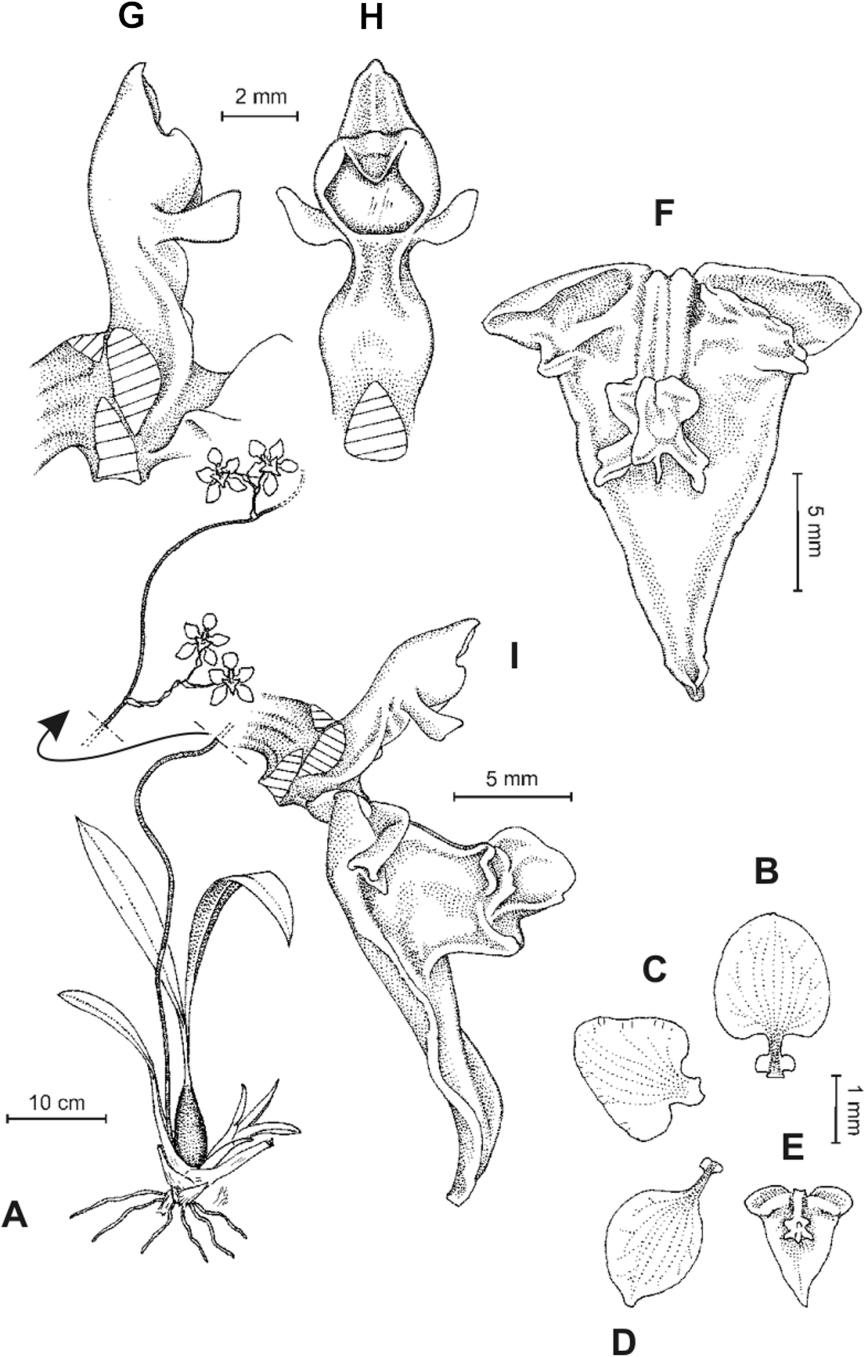
*Cyrtochilum flavostellulare* Dalström. (A) Habit and inflorescence fragment; (B) Dorsal sepal; (C) Petal; (D) Lateral sepal; (E and F) Lip; (G) Gynostemium, side view; (H) Gynostemium, front view; (I) Gynostemium and lip, side view. Drawn by Stig Dalström from *Steyermark 54159* (F).

Pseudobulbs caespitose, about 8–10 cm long, 3–4 cm wide, ovoid, bifoliate at the apex, basally surrounded by 4–6 foliaceous sheaths. Leaves 28–34 cm long, 3–4 cm wide, subpetiolate, conduplicate, elliptic, acuminate. Inflorescence up to 200 cm long, axillary, from the uppermost sheaths, erect, then wiry, paniculate, with widely spaced, flexuous, few-flowered branches. Flower with bronzy brown sepals, bright yellow petals with basal bronzy brown area and brownish lip with yellow callus. Floral bracts 10–20 mm long, involute-cucullate. Pedicel with ovary up to 40 mm long. Dorsal sepal clawed; claw about 7–8 mm long, auriculate; blade 23 mm long, 20 mm wide, base subcordate, broadly ovate to orbicular, rounded to acute at the apex. Petals clawed; claw 2–3 mm long, wingless; blade about 22 mm long, 20 mm wide, broadly and obliquely ovate, base cordate, obtuse to acute at apex, slightly undulate. Lateral sepals clawed; claw 7–8 mm long, auriculate; blade 22–23 mm long, 18 mm wide, obliquely broadly ovate to orbicular, acute, base cuneate. Lip 20 mm long and wide, triangular-hastate, 3-lobed at the base; lateral lobes slightly concave, entire, obtuse to acute; callus consisting of central longitudinal, fleshy, faintly 3-partite, low keel, turning abruptly into a raised, butterfly-shaped, spreading, lobulate and angulate structure, basally flanked by low, fleshy, spreading ridges; apical part triangular, slightly recurved, broadly acuminate, apically canaliculate. Gynostemium 8–9 mm long, erect, with large, truncate, subrectangular spreading wings on each side of stigmatic surface (Fig. 54).

*Ecology*: Epiphytic or terrestrial.

*Distribution*: Ecuador. Alt. 1,830–2,430 m.

*Notes*: Species resembling *C. macranthum* distinguished by the lip callus form. In *C. macranthum* the lip callus consists of an elevated keel apically 3-lobed and flanked basally by 3-lobed process. The lip callus of the somewhat similar *C. cumandae* consists of a tall, narrow, obliquely stretched forward lamella which is 2-apiculate apically and the lip lamina below is ornamented with a low, longitudinal, variably few-gibbous lamella, above with 2 obliquely spreading, approximate teeth, in the middle with a large, triangular tooth, thereafter on both sides with a small, low, horizontally spreading, rounded lamella. Unlike it, the lip callus of *C. flavostellulare* is formed of central longitudinal, 3-partite, low keel, turning abruptly into a raised, butterfly-shaped, spreading, lobulate and angulate structure, basally flanked by low, fleshy, spreading ridge.

*Representative specimen*: ECUADOR. **El Oro**: Between Paccha and Puente Grande, 1,830–2,430 m, 26 August 1943, *J.A. Steyermark 54159* (F).

***Cyrtochilum tenense*** (Rchb.f. & Warsz.) Kraenzl., Notizbl. Bot. Gart. Berlin-Dahlem 7: 95. 1917. ≡ *Oncidium tenense* Rchb.f. & Warsz., Bonplandia (Hannover) 2: 107. 1854. TYPE: Colombia. *Hartweg 1429* (?) (holotype, W-R! 5055, UGDA-DLSz!—drawing).

**Figure 55:**
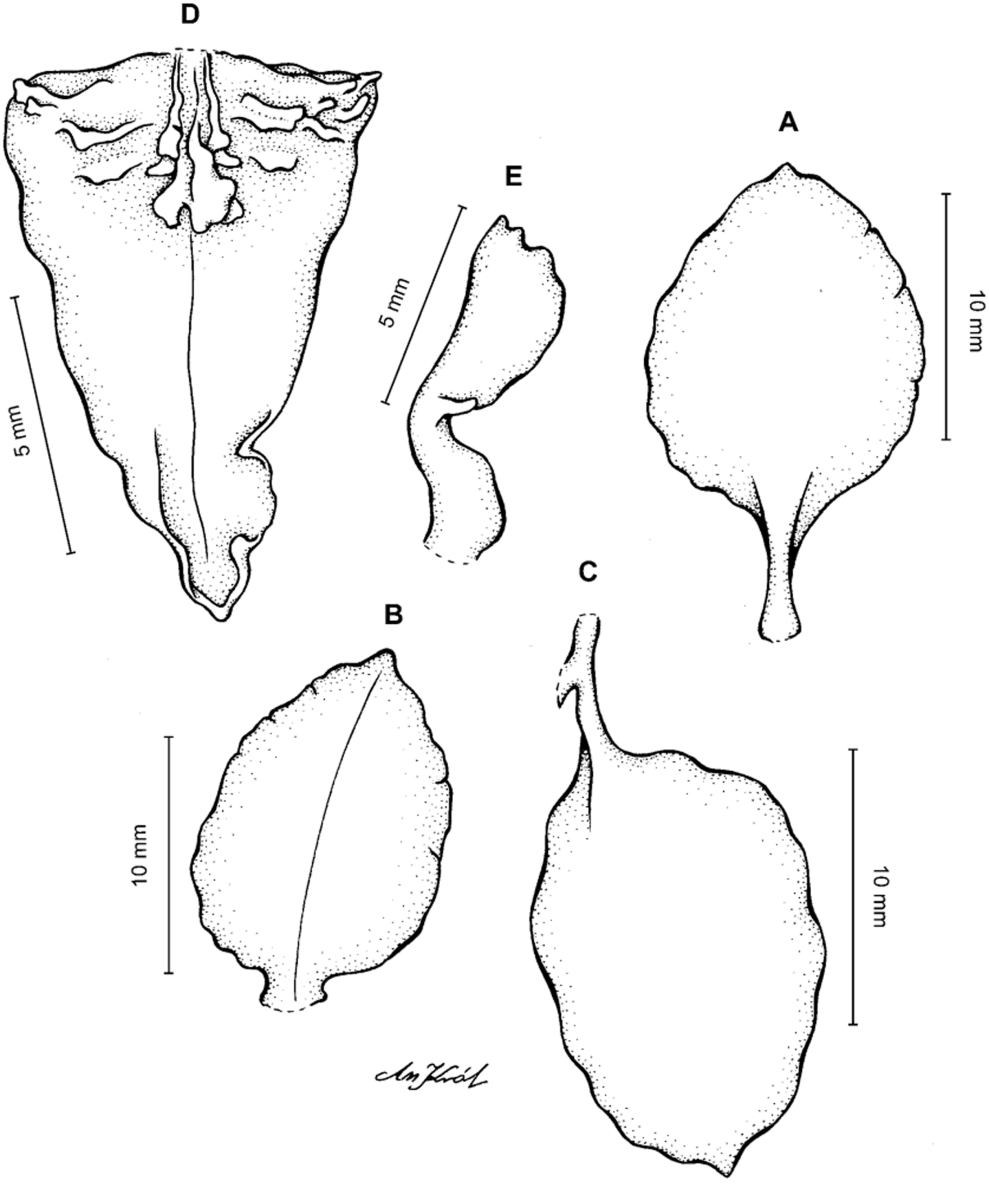
*Cyrtochilum tenense* (Rchb.f. & Warsz.) Kraenzl. (A) Dorsal sepal; (B) Petal; (C) Lateral sepal; (D) Lip; (E) Gynostemium, side view. Drawn by Anna Król from *Hartweg 1429* (W-R).

Vegetative parts unknown. Flowers medium-sized, conspicuous. Floral bracts 10 mm long. Pedicel and ovary 30 mm long. Dorsal sepal clawed; claw 5 mm long, narrow, canaliculate, wingless; blade 14 mm long, 12 mm wide, elliptic-ovate, margins entire, base cuneate, apex subobtuse. Petals clawed; claw about 2 mm long; blade 15 mm long, 11 mm wide when spread, obliquely elliptic-ovate, base rounded, apex subobtuse, margins entire, slightly undulate. Lateral sepals prominently clawed; claw 6 mm long, narrow, wingless, basally connate to each other; blade 16 mm long, 13 mm wide, obliquely elliptic in general outline, basally cuneate, apex subacute, margins entire. Lip 11 mm long in total, 6 mm wide at the base, oblong triangular, curved in natural position just above the base, base truncate, lateral lobes obtuse, apex subacute, canaliculate; callus relatively small, consisting of a prominent, median, nose-like keel, flanked by obliquely rhombic wings and several, irregular additional thickenings beyond the main callus. Gynostemium 8 mm long, subarcuate, connate basally with the lip, lateral appendages rudimentary, obliquely digitate, upcurved (Fig. 55).

*Ecology*: No data.

*Distribution*: Colombia.

*Notes*: This species differs from similar *C. microcallosum* in the lip callus form. In the latter it consists of rather narrow pad, with rather uneven, irregular surface and additional thickenings appearing along the line of curvation of basal lip lobes. Another species with which *C. tenense* can be confused is *C. guariniae*. The latter species, however, has undulate margins of petals and lateral sepals, irregularly crispate-undulate margins of dorsal sepal, strongly sigmoid gynostemium with obliquely oblong-triangular appendages and distinct lip callus. It consists in this species of two ridges in the basal part, with shallow groove between them, then divided into some, irregular, thickenings, with additional thickenings along the bending line of basal wings.

*Representative specimen*: COLOMBIA. [**Cundinamarca**]: Near the village of Tena, in the prov. of Bogotá, *C. Hartweg 1429* (?) (W-R! 5055).

***Cyrtochilum guariniae*** Szlach. & Kolan., Webbia 70: 83. 2015. TYPE: Colombia. *Guarín O. 89* (holotype, COL! 214684; UGDA-DLSz!—drawing).

**Figure 56:**
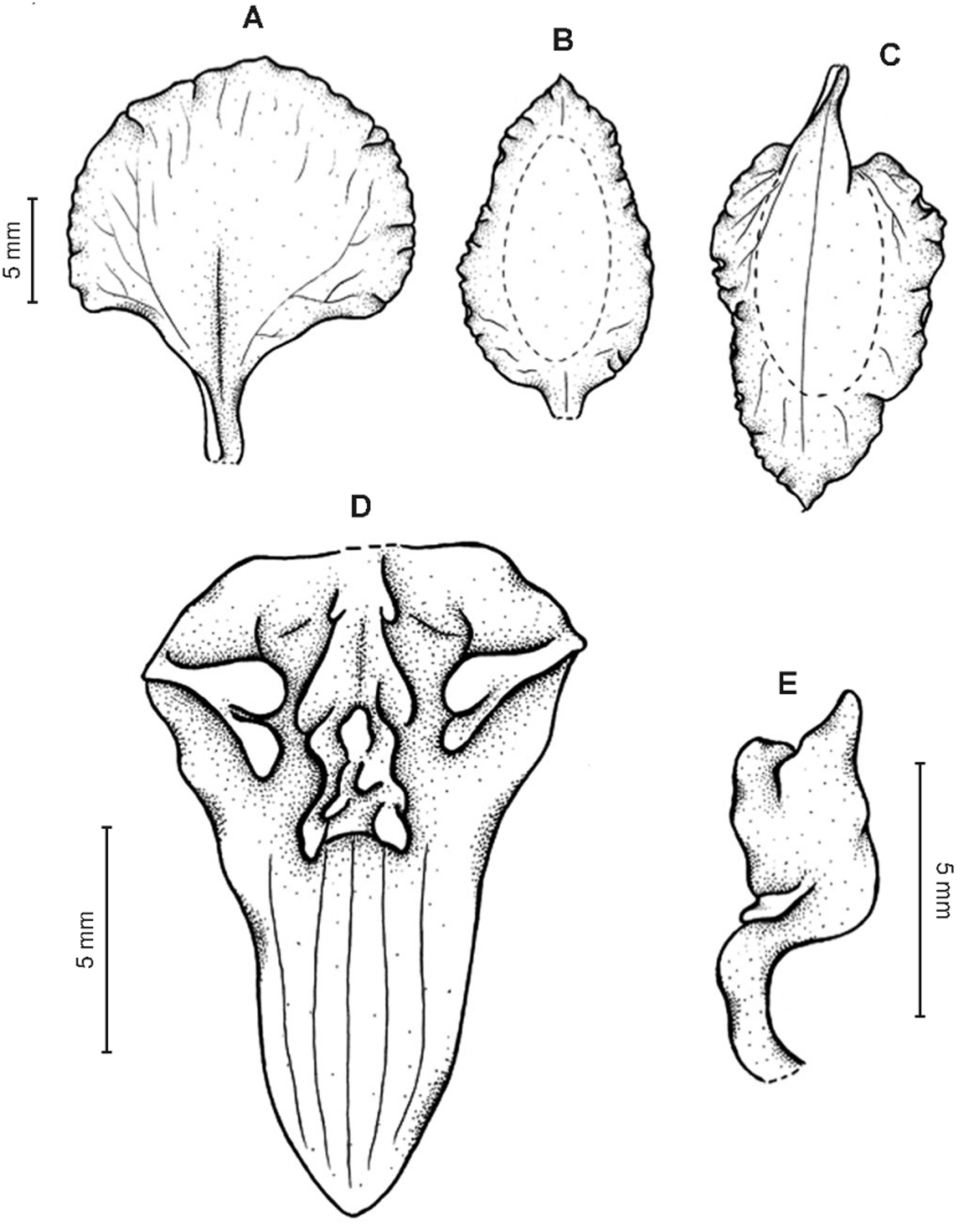
*Cyrtochilum guarinae* Szlach. & Kolan. (A) Dorsal sepal; (B) Petal; (C) Lateral sepal; (D) Lip; (E) Gynostemium. Drawn by Natalia Olędrzyńska from *Guarín O. 89* (COL).

Plants long-rhizomatous. Pseudobulbs 12 cm long, 1.5 cm in diameter, oblong-ovoid, somewhat compressed, elliptic in cross-section, basally concealed by leafy sheaths, bifoliate. Leaves up to 40 cm long and 4.5 cm wide, linear to linear-oblanceolate, acute. Inflorescence serpentine, branching. Flowers large, conspicuous. Floral bracts 10 mm long. Pedicel 15 mm long, ovary about 5 mm long. Dorsal sepal clawed; claw 6 mm long, narrow, canaliculate, wingless; blade 17 mm long and wide, flabellate, base attenuate towards claw, margins irregularly crispate, undulate, apex obtuse. Petals subsessile; claw up to 2 mm long, wingless; blade 16 mm long, 11 mm wide when spread, oblong-ovate, somewhat oblique, subacute at the apex, margins undulate. Lateral sepals clawed; claw 7 mm long, narrow, wingless; blade 20 mm long, 14 mm wide, oblong-ovate in general outline, subacute at the apex, subcordate at the base, margins undulate. Lip 15 mm long in total, 10 mm wide, slightly curved in natural position, undivided, oblong-triangular in general outline, base truncate, lateral lobes obtuse; callus consisting of two ridges in the basal part, with shallow groove between them, then divided into some, irregular, thickenings, with additional thickenings along the bending line of lateral lobes. Gynostemium 8 mm long, strongly sigmoid, connate basally with the lip, lateral appendages obliquely oblong-triangular, falcate, directed forwards (Fig. 56).

*Ecology*: No data.

*Distribution*: Colombia.

*Notes*: This species resembles somewhat *C. flavostellulare*, from which it differs by having undulate tepals (vs non-undulate sepals and somewhat undulate petals), flabellate dorsal sepal set on a wingless claw (vs elliptic blade set on a winged claw), subsessile petals (vs shortly clawed petals) with rounded base (vs petals obliquely truncate), and by the basal lip wings being as wide as lip lamina (vs basal wings much wider than lip lamina). The other taxon with which *C. guariniae* can be confused is *C. brachypterum*. The latter species has, however, a different form of lip callus. It is apically dissected into 12 lobules, more or less wrinkled, base has entire margins. Moreover, its gynostemium is basally adorned with chin-like projections, and lateral appendages are transformed into wings flanking gynostemium. We discuss differences between *C. guariniae* and *C. tenense* above.

*Representative specimen*: COLOMBIA. **Valle del Cauca**: La Nevera, 26 January 1980, *I. Guarín O. 89* (COL!; UGDA!—drawing of type).

***Cyrtochilum christensonianum*** Szlach. & Kolan., Webbia 70: 90. 2015. TYPE: Colombia. *Killip & Smith 533* (holotype, COL! 107147; UGDA-DLSz!—drawing).

**Figure 57:**
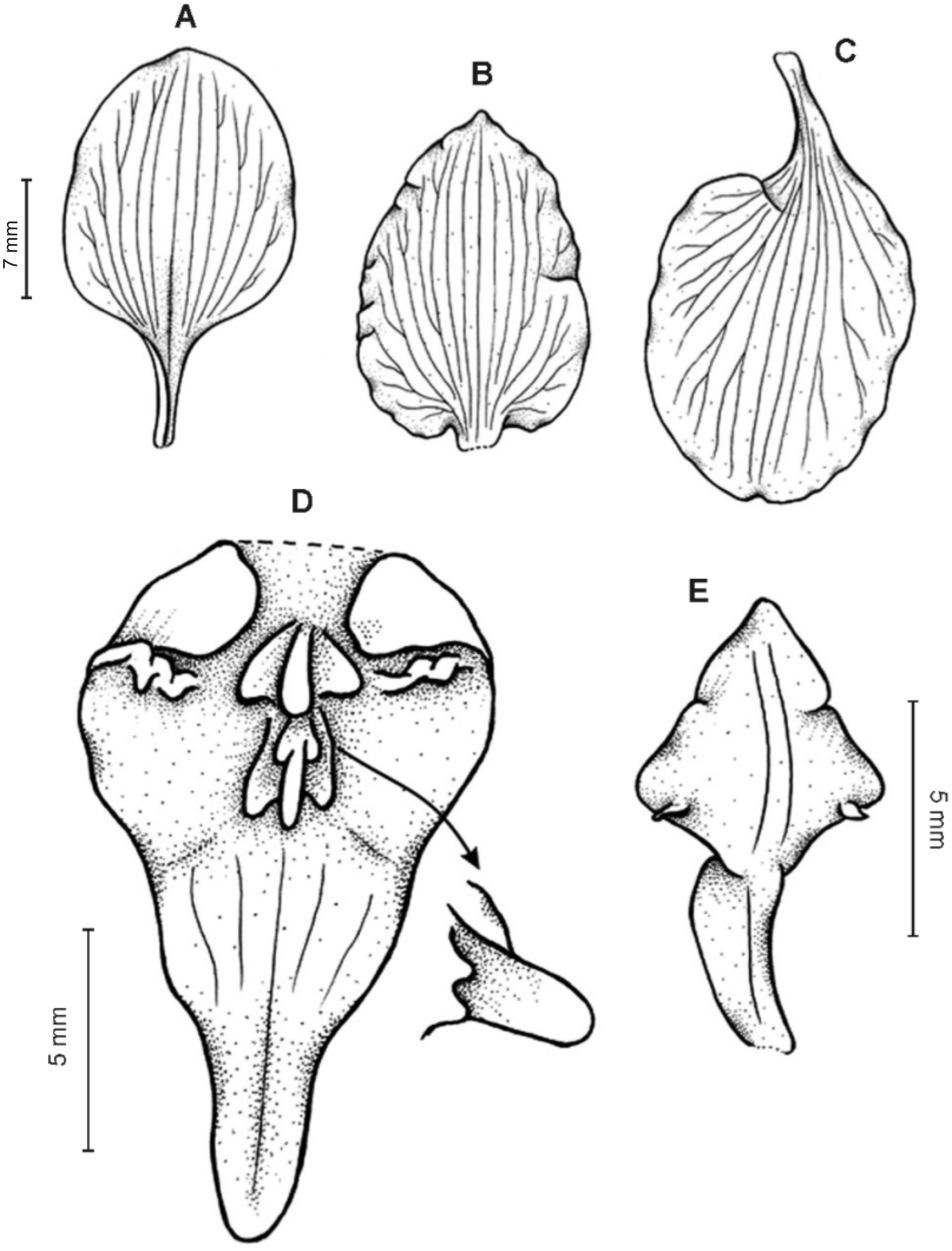
*Cyrtochilum christensonianum* Szlach. & Kolan. (A) Dorsal sepal; (B) Petal; (C) Lateral sepal; (D) Lip; (E) Gynostemium. Drawn by Natalia Olędrzyńska from *EPA 533* (COL).

Plants long-rhizomatous. Pseudobulbs incomplete in herbarium material, probably 10 cm long and 2.5–3 cm wide, basally concealed by leafy sheaths, bifoliate. Leaves to 45 cm long and 4.5 cm wide, linear to linear-oblanceolate, acute. Inflorescence serpentine, branching. Flowers large, conspicuous. Floral bracts 7 mm long. Pedicel 28 mm long, ovary about 8 mm long. Dorsal sepal clawed; claw 5 mm long, narrow, canaliculate, wingless; blade 23 mm long, 20 mm wide, elliptic-suborbicular, base attenuate towards claw, margins entire, apex obtuse. Petals subsessile; claw up to 2 mm long, wingless; blade 21 mm long in total, 17 mm wide when spread, elliptic-ovate, somewhat oblique, subacute at the apex, margins weakly undulate. Lateral sepals clawed; claw 6 mm long, narrow, wingless; blade 22 mm long, 19 mm wide, obliquely elliptic-suborbicular in general outline, rounded at the apex, subcordate at the base, margins entire. Lip 15 mm long in total, 10 mm wide, slightly curved in natural position, undivided, narrowly triangular in general outline, cordate at base, lateral lobes rounded; callus consisting of three ridges, terminating in 5 unequal lobes, additional thickenings along the bending line of basal wings. Gynostemium 10 mm long, sigmoid, connate basally with the lip, lateral appendages obscure, digitate (Fig. 57).

*Ecology*: No data.

*Distribution*: Colombia.

*Representative specimen*: COLOMBIA. **Cundinamarca**?: La Cabrera-Pandi, July 1930, *E.P. Killip & A.C. Smith 533* (COL!; UGDA!—drawing of type).

*Notes*: This species resembles *C. guariniae*, but it is easily distinguishable from this orchid by having non-undulate sepals (vs sepals undulate), elliptic-suborbicular dorsal sepal (vs flabellate), elliptic-suborbicular lateral sepals (vs oblong-ovate), lip with cordate base (vs truncate) and very obscure, digitate gynostemium projections (vs obliquely oblong-triangular). Cyrtochilum *christensonianum* differs from *C. microxiphion* in the connation of lateral sepals claws. In *C. microxiphion* claws are connate in the basal half (vs free in *C. christensonianum*).

***Cyrtochilum microxiphion*** (Rchb. f.) Kraenzl., Notizbl. Bot. Gart. Berlin-Dahlem 7: 95. 1917. *≡ Oncidium microxiphium* Rchb. f. Linnaea 41: 21. 1877[1876]. TYPE: Bolivia. *Pearce s.n*. (holotype, W-R! 5055, UGDA-DLSz!—drawing).

**Figure 58:**
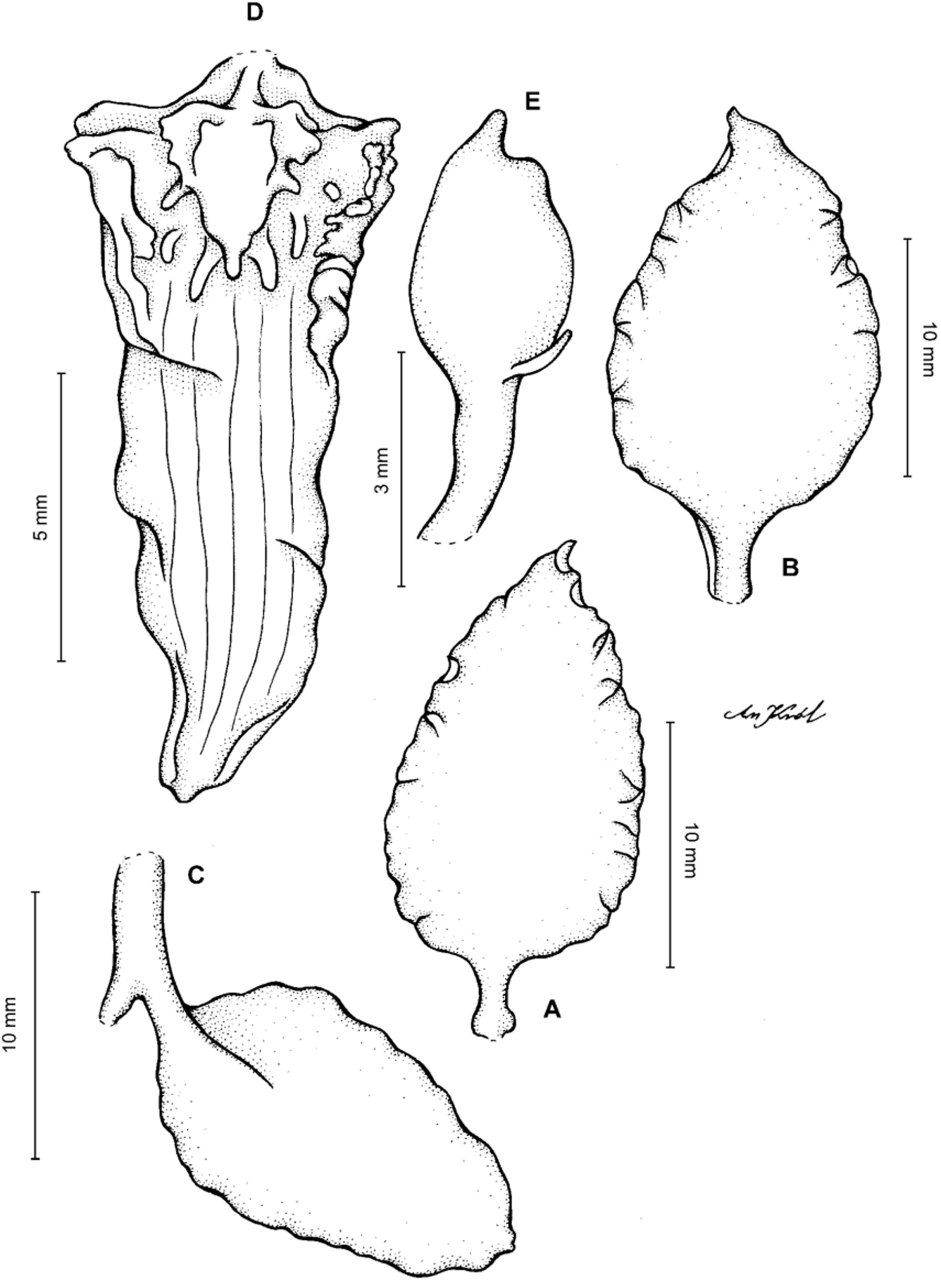
*Cyrtochilum microxiphion* (Rchb. f.) Kraenzl. (A) Dorsal sepal; (B) Petal; (C) Lateral sepal; (D) Lip; (E) Gynostemium, side view. Drawn by Anna Król from *Pearce s.n*. (W-R).

Plants long-rhizomatous. Pseudobulbs about 10 cm long and 2 cm wide, basally concealed by leafy sheaths, bifoliate. Leaves up to 45 cm long and 3 cm wide, linear to linear-oblanceolate, acute. Inflorescence 2–3 m long, serpentine, branching. Flowers large, conspicuous. Floral bracts 8–10 mm long. Pedicellate ovary 20–30 mm long. Dorsal sepal clawed; claw up to 5.5 mm long, narrow, canaliculate, wingless; blade 18–20 mm long, 8–14 mm wide, elliptic to broadly ovate, base cuneate, margins entire, more or less undulate, apex obtuse to subobtuse. Petals subsessile; claw up to 2 mm long, wingless; blade 17–19 mm long, 10–12.5 mm wide when spread, ovate, somewhat oblique, subacute at the apex, margins weakly undulate. Lateral sepals clawed; claw up to 6 mm long, narrow, wingless, connate in the basal half; blade 20–24 mm long, 9–14 mm wide, obliquely elliptic-ovate to oblong ovate in general outline, obtuse at the apex, cuneate at the base, margins entire. Lip up to 15 mm long in total and 5 mm wide, slightly curved in natural position, narrowly triangular-ligulate in general outline, truncate at base, lateral lobes auriculate; callus consisting of massive, central tissue, with more or less divided basal margins, flanked at apex by small ridges and some additional outgrowths. Gynostemium 5.5–8.5 mm long, suberect, connate basally with the lip, lateral appendages obscure, digitate (Fig. 58).

*Ecology*: Terrestrial or epiphytic on steep hillside in partial shade among *Pitcairnia sp*., also growing on rocks and mossy trees in upper elevation cloud forest.

*Distribution*: Colombia, Bolivia (Vásquez & Dodson, 1982), Venezuela. Alt. 1,650–2,500 m.

*Notes*: This species can be distinguished by a callus confined to the basal third or quarter of the lip and rather narrow tepals, only slightly undulate. It can be confused with *C. microcallosum* described below, but the latter species has distinctly wider lip (8–9 mm) with cordate base and different lip callus. It is small, forming narrow pad, with rather uneven, irregular surface, with additional thickenings appearing along the line of curvation of basal lip lobes.

*Representative specimens*: COLOMBIA. **Cundinamarca**: Between Chuscal and La Vega, 2,200 m, 21 January 1976, *T. C. Plowman, D. Vaughan & R. Jaramillo 5240* (AMES!, UGDA-DLSz!—fragment, photo, drawing); Between Tequendama Falls and Santandercito, on rocks, 5,500–7,000 ft, 12 July 1961, *L. A. Garay, C. E. McClennen, A. Kapuler 224* (AMES!, UGDA-DLSz!—drawing). VENEZUELA. **Tachira**: Along road between Rubio and Delicias, […] km above Rubio (SW), 23 August 1976, *G. Bunting & T. Croat 38455* (MO!, UGDA-DLSz!—drawing).

***Cyrtochilum microcallosum*** Szlach. & Kolan., J. Torrey Bot. Soc. 144(1): 89. 2017. TYPE: Colombia. *Uribe Uribe 6255* (holotype, COL! 115833, UGDA-DLSz!—drawing).

**Figure 59:**
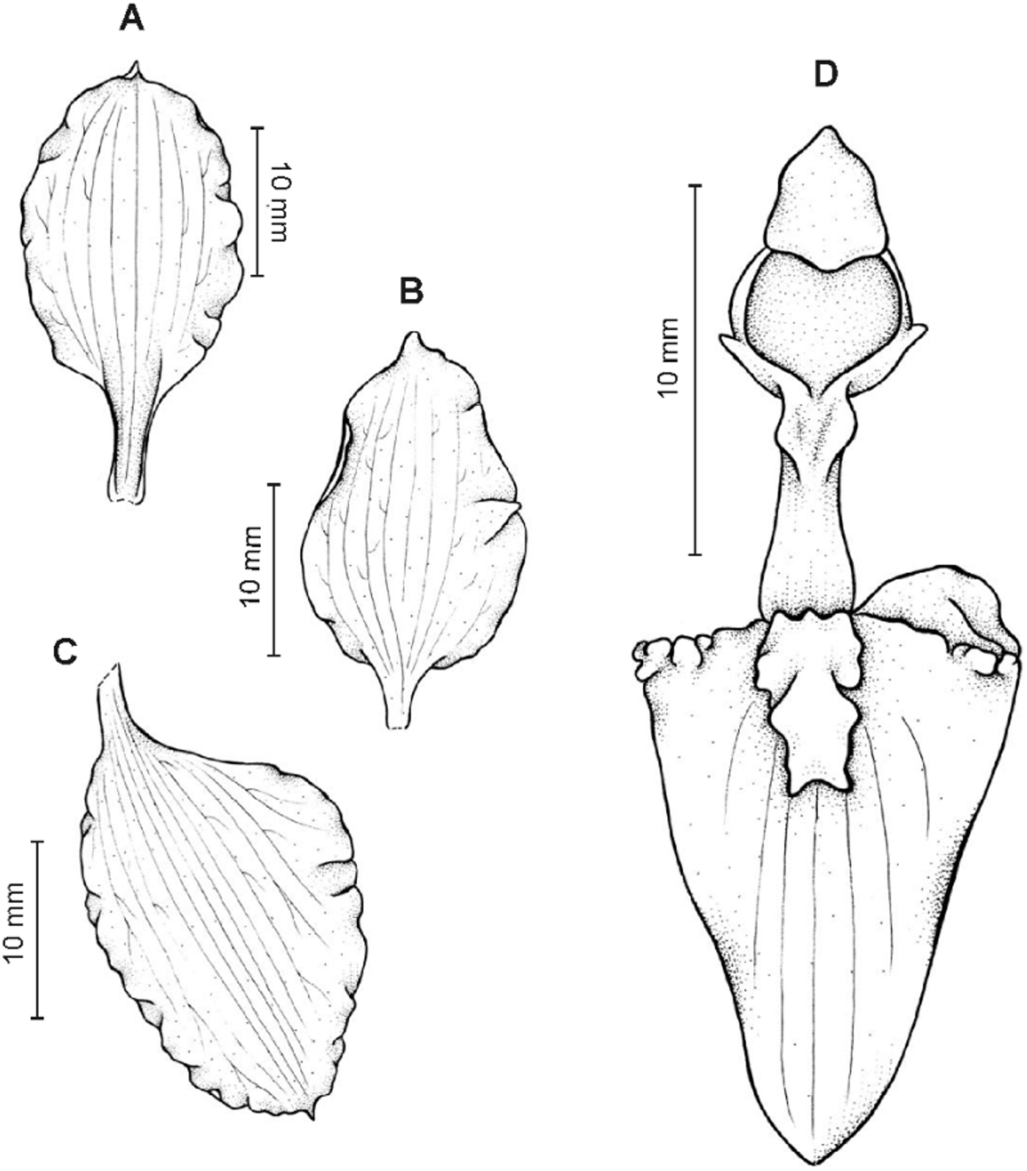
*Cyrtochilum microcallosum* Szlach. & Kolan. (A) Dorsal sepal; (B) Petal; (C) Lateral sepal; (D) Lip and gynostemium, front view. Drawn by Natalia Olędrzyńska from *Uribe Uribe 6255* (COL).

Plants long-rhizomatous. Pseudobulbs up to 13 cm long, about 1.3–1.5 cm in diameter, oblong, somewhat laterally compressed, more or less elliptic in cross section, concealed basally by leafy sheaths, bifoliate. Leaves about 50 cm long, 2.5–3 cm wide, linear-oblanceolate, acute. Inflorescence serpentine, branching. Flowers large, conspicuous. Floral bracts 10 mm long. Pedicel 25 mm long, ovary about 5–6 mm long. Dorsal sepal clawed; claw 6–7 mm long, rather narrow, canaliculate, without basal wings; blade 22 mm long, 15 mm wide, elliptic, base cuneate, margins gently undulate, apex shortly acuminate. Petals clawed; claw 3 mm long, wingless; blade 20 mm long, 14 mm wide when spread, oblong-ovate, acute at the apex, margins almost flat or gently undulate. Lateral sepals clawed; claw 6 mm long, narrow, without basal wings, connate together in the lower half; blade 25 mm long, 15 mm wide, obliquely elliptic-ovate in general outline, attenuate towards base, shortly apiculate at the apex, margins slightly undulate. Lip 15 mm long in total, 8–9 mm wide, curved in natural position, oblong-cordate or ovate-cordate when flattened in general outline, lateral lobes curved down, apical part subobtuse; callus forming small, rather narrow pad, with rather uneven, irregular surface, additional thickenings appear along the line of curvation of basal lip lobes. Gynostemium 13 mm long, sigmoid, connate basally with the lip, lateral appendages obscure, digitate (Fig. 59).

*Ecology*: Terrestrial in premontane and montane forest, also found in roadside and among scrub.

*Distribution*: Colombia. Alt. 1,850–2,500 m.

*Notes*: This species resembles *C. ventilabrum*, from which it is easily separable by the form of the lip callus. In *C. ventilabrum* the lip callus consists of large mass of the tissue occupying larger part of the basal lip base. The callus is highly complicated and consists of projections of various forms. Additionally, sepals and petals of *C. microcallosum* are weakly undulate, whereas in *C. ventilabrum* they are strongly undulate.

*Representative specimens*: COLOMBIA. **Boyacá**: Mpio. Pajarito. Corregimiento de Corinto, 2,200 m, 10 October 1967, *G. Lozano, F. Diaz & S. Diaz 779* (COL!). **Cundinamarca**: Mpio. Albán. Hacienda San Pablo, 1,850 m, 6 January 1958, *H. Schmidt-Mumm 9* (COL!); Cabrera, 1,900–2,000 m, 21 February 1969, *L. Uribe Uribe 6255* (COL!). **Magdalena**: Sierra Nevada de Santa Marta. Transecto del Alto Rio Buritaca. Cuchilla a 2,500 m, 29 July 1977, *R. Jaramillo, Th. van der Hammen, A. M. Cleef & O. Rangel 5325* (COL!).

***Cyrtochilum zebrinum*** (Rchb.f.) Kraenzl., Notizbl. Bot. Gart. Berlin-Dahlem 7: 94. 1917. ≡ *Odontoglossum zebrinum* Rchb.f., Linnaea 22: 849. 1850. TYPE: Venezuela. *Moritz 1615* (holotype, W-R! 36969; isotype: W-R! 36974). ≡ *Oncidium zebrinum* (Rchb.f.) Rchb.f., Bonplandia (Hannover) 2: 12. 1854.

= *Cyrtochilum engelii* (Rchb.f.) Kraenzl., Notizbl. Bot. Gart. Berlin-Dahlem 7: 92. 1917. ≡ *Oncidium engelii* Rchb.f., Linnaea 41: 22. 1877. TYPE: Venezuela. *Engel s.n*. (W).

**Figure 60:**
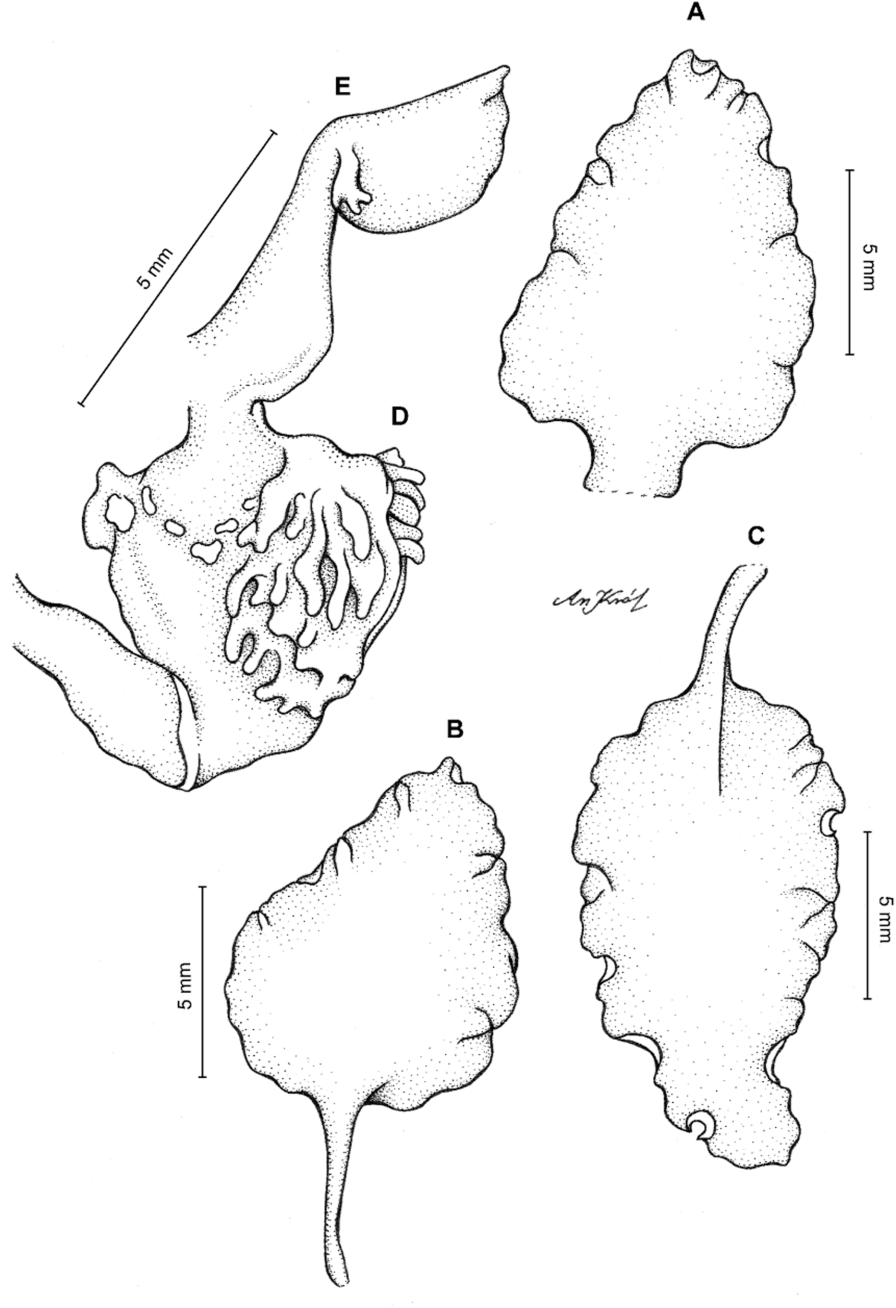
*Cyrtochilum zebrinum* (Rchb.f.) Kraenzl. (A) Dorsal sepal; (B) Petal; (C) Lateral sepal; (D) Lip; (E) Gynostemium, side view. Drawn by Anna Król from *Moritz 1615* (W-R).

Vegetative parts not seen. Flowers medium-sized, conspicuous. Floral bracts 7 mm long. Pedicel and ovary 30 mm long. Dorsal sepal clawed; claw 5–6 mm long, narrow, canaliculate, wingless; blade 13 mm long, 7 mm wide, elliptic-ovate, margins entire, somewhat undulate, base truncate, apex obtuse. Petals clawed; claw about 2 mm long; blade 12 mm long, 7 mm wide when spread, obliquely elliptic-ovate, base truncate, apex obtuse, margins entire, undulate. Lateral sepals prominently clawed; claw 4 mm long, narrow, wingless; blade 15 mm long, 6–7 mm wide, obliquely oblong elliptic-ovate in general outline, apex obtuse, base cuneate, margins entire, undulate. Lip 12 mm long in total, 5 mm wide at base, gently curved in natural position exposing large callus, oblong triangular in outline, base truncate, apex attenuate, subobtuse; callus massive, dissected into numerous lobes of various forms and sizes radiating from the centre, with some additional outgrowths beyond main callus. Gynostemium 6 mm long, arcuate, connate basally with the lip, swollen in the upper half, lateral appendages oblong, apically 3-lobed, pendent, not reaching the anther base (Fig. 60).

*Ecology*: Epiphyte or terrestrial on steep embankments in extremely wet montane forest.

*Distribution*: Colombia (?), Ecuador, Venezuela. Alt. 1,850 m.

*Notes*: Characteristic features of this species are its lip callus and its gynostemium appendages. It can be confused with *C. lucescens* and *C. brachypteron*, both with similar lip calli. In the former however, gynostemium appendages are unlobed and upper surface of lip calli is papillate. In the latter species gynostemium is devoid of any appendage.

Dalström (2001) amalgamated *C. engelii* with *C. zebrinum*. We were not able to trace *Engel s.n*. collection in W. Based on Dalström’s authority we accept this viewpoint.

*Representative specimens*: ECUADOR. **Carchi**: km 73, Tulcan to Maldonado, 1,850 m, July 1985, *C. Dodson & A. Embree 16177* (RPSC, QCNE—Dodson & Dodson, 1989); km 6, Maldonado to Tulcan, May 1987, *B. Stein 2893* (RPSC—Dodson & Dodson, 1989). VENEZUELA. Caracas, Sep., *K. Moritz 1615* (W-R!).

***Cyrtochilum lucescens*** (Rchb.f.) Kraenzl., Notizbl. Bot. Gart. Berlin-Dahlem 7: 93. 1917. ≡ *Oncidium lucescens* Rchb.f., Gard. Chron., ser. 3, 1: 799. 1887. TYPE: Cult. *Williams 3* (holotype, W-R! 3233; isotype, W-R! 48949; UGDA-DLSz!—drawing).

**Figure 61:**
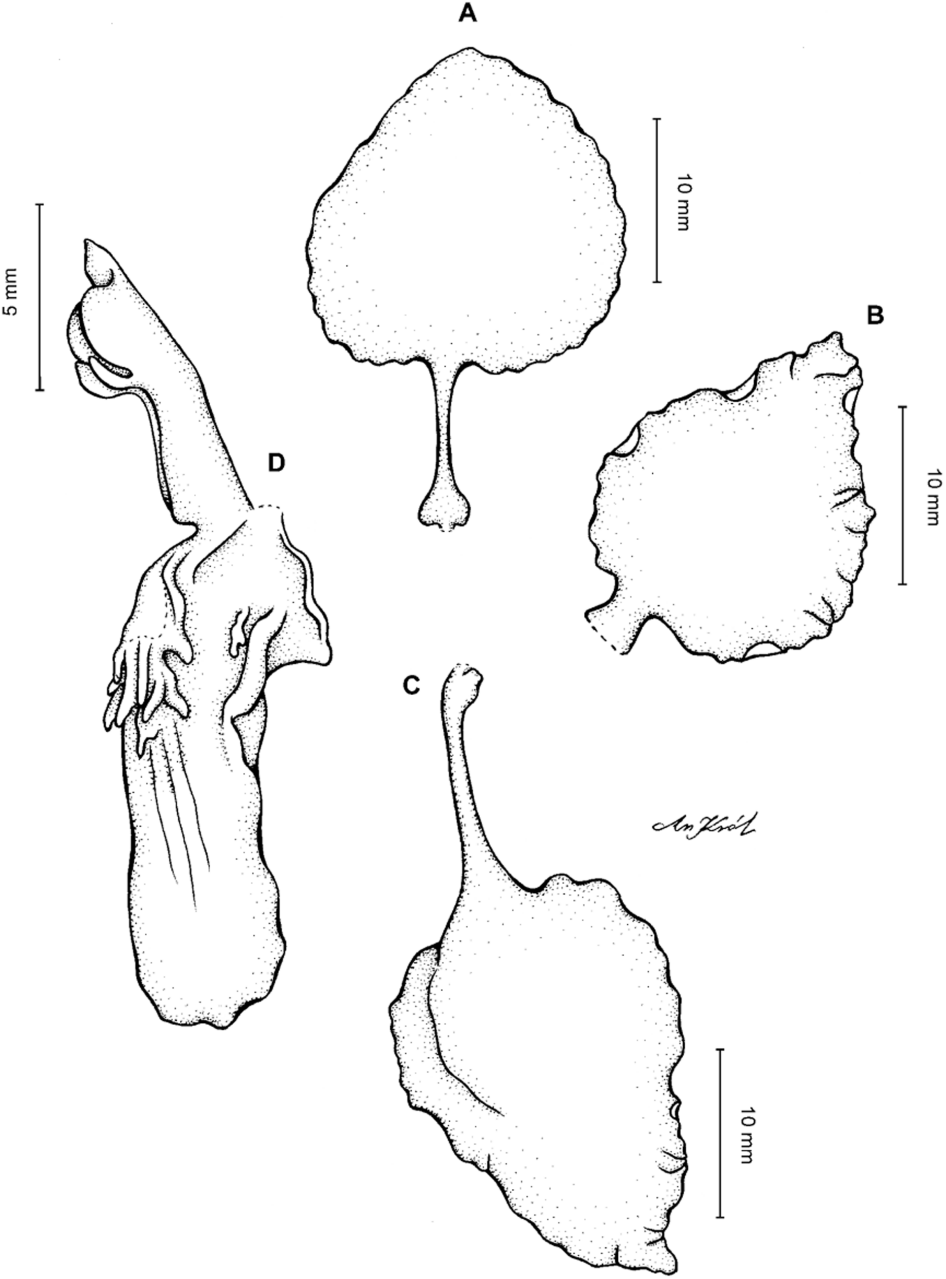
*Cyrtochilum lucescens* (Rchb. f.) Kraenzl. (A) Dorsal sepal; (B) Petal; (C) Lateral sepal; (D) Lip and gynostemium, side view. Drawn by Anna Król from *Williams 3* (W-R).

Vegetative parts unknown. Flowers large, conspicuous, showy. Floral bracts 15 mm long. Pedicel and ovary 35 mm long. Dorsal sepal clawed; claw 9 mm long, narrow, canaliculate, with triangular-ovate wings just above the base; blade 20 mm long and wide, broadly ovate-cordate, base truncate, margins entire, apex rounded. Petals clawed; claw about 3–4 mm long, wide; blade 20 mm long, 16 mm wide when spread, somewhat oblique, broadly ovate, base truncate, obtuse at the apex, margins irregularly undulate. Lateral sepals prominently clawed; claw 12–13 mm long, narrow, with basal obscure wing on the outer margin; blade 28–30 mm long, 17 mm wide, obliquely elliptic-ovate in general outline, basally cuneate-truncate, subacute at the apex, margins very slightly undulate. Lip 11–12 mm long in total, curved in natural position just above the base; basal part 4.5 mm long, about 7–8 mm wide, broadly rhombic, convex in the centre; callus large, consisting of a flat, papillate, pad-like tissue in the base terminated by palmate apical outgrowth and some additional projections beyond the main callus; apical part 7 mm long, about 3 mm wide, ligulate, rounded at the apex, prominently papillate-ciliolate along veins in the lower third. Gynostemium 9 mm long, subarcuate, connate basally with the lip, with chin-like outgrowth just above base, lateral appendages obliquely oblanceolate, not reaching the anther base (Fig. 61).

*Ecology*: No data.

*Distribution*: No data.

*Notes*: This species has very characteristic gynostemium morphology. Unlike in *C. biscellum* the gynostemium appendages of *C. lucescens* are obliquely oblanceolate (vs wing-like or obtriangular, apically dissected into 3–4, unequal lobes). Cyrtochilum *lucescens* appears to be similar to *C. brachypterum*, but in the latter base of the dorsal sepal is broadly cuneate, margins are broadly cuneate, blades of petals and lateral sepals are smaller, and the lip callus is glabrous and very complicated, i.e. consisting of tissue apically dissected into 12 lobules, which are more or less wrinkled. The gynostemium is strongly arcuate in the upper half.

*Representative specimen*: ORIGIN UNKNOWN. *Williams 3* (W-R!).

***Cyrtochilum brachypterum*** (Linden & Rchb. f.) Kraenzl. *in* Engler, Pflanzenr., Orchid.-Monandr.-Oncid. 319. 1922. ≡ *Oncidium brachypterum* Linden & Rchb. f., Ill. Hort. 29: 99. 1882. TYPE: Colombia. *Linden 72* (holotype, W-R! 48948; UGDA-DLSz!—drawing).

**Figure 62:**
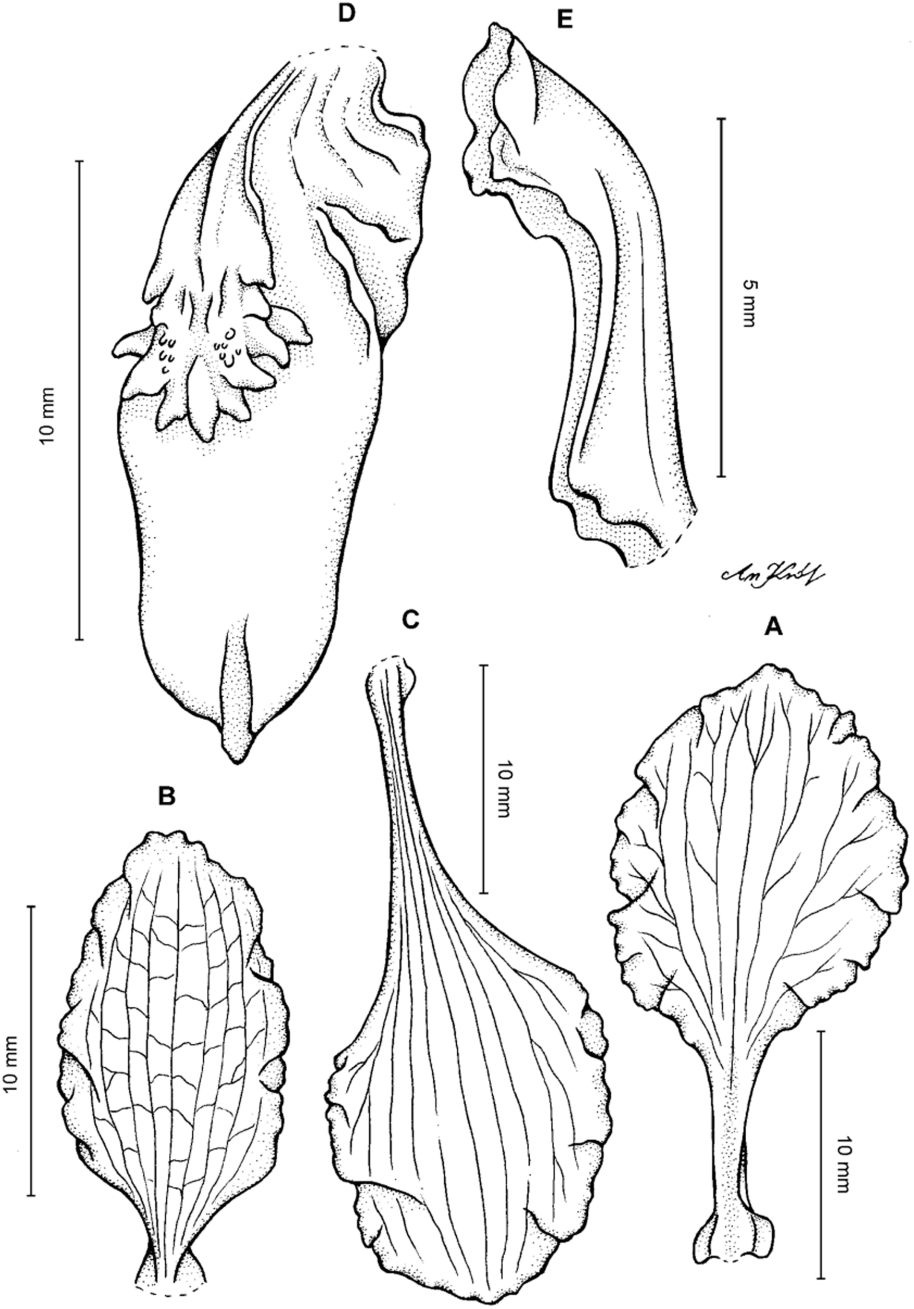
*Cyrtochilum brachypterum* (Linden & Rchb. f.) Kraenzl. (A) Dorsal sepal; (B) Petal; (C) Lateral sepal; (D) Lip; (E) Gynostemium. Drawn by Anna Król from *Linden 72* (W-R).

Vegetative parts not seen. Flowers rather large, showy. Floral bracts about 18 mm long. Pedicel and ovary about 30–35 mm long. Dorsal sepal clawed; claw 10 mm long, narrow, canaliculate, with prominent rhombic wings just above the base; blade 14 mm long and wide, elliptic-suborbicular, base broadly cuneate, margins undulate, entire, apex obtuse. Petals broadly clawed; claw about 3–4 mm long, winged; blade 15 mm long, 8 mm wide when spread, oblong elliptic, obtuse at the apex, almost symmetric, margins undulate, more or less entire. Lateral sepals prominently clawed; claw about 10 mm long, narrow, with rather obscure basal wing; blade about 20 mm long, 12 mm wide, obliquely elliptic-suborbicular to oblong flabellate in general outline, base cuneate, rounded at the apex, margins somewhat undulate. Lip 15 mm long in total, about 6 mm wide when spread, pendent just above the base, oblong-obovate in general outline, exposing large callus in the centre, base somewhat cordate, apex canaliculate, shortly acuminate; callus very complicated, apically dissected into 12 lobules, more or less wrinkled, base with entire margins, thick. Gynostemium 8 mm long, strongly arcuate in the upper half, connate basally with the lip, and just above it chin-like, lateral appendages transformed into wings flanking gynostemium (Fig. 62).

*Ecology*: No data.

*Distribution*: Colombia, Ecuador (?).

*Notes*: *Cyrtochilum brachypterum* is somewhat similar to both *C. zebrinum* and *C. lucescens*. Unlike the latter its callus is glabrous. The lip callus of *C. lucescens* consists of a flat, papillate, pad-like tissue in the base which is terminated by palmate apical outgrowth and some additional projections beyond the main callus. In *C. zebrinum* lip callus is dissected into numerous lobes of various forms and sizes radiating from the centre, with some additional outgrowths beyond main callus. Cyrtochilum *brachypterum* differs from *C. guariniae* in the form of the lip callus which in the latter species consists of two ridges in the basal part, with shallow groove between them, then divided into some, irregular, thickenings, additional thickenings along the bending line of basal wings, gynostemium lateral appendages obliquely oblong-triangular, falcate, directed forwards.

*Representative specimen*: COLOMBIA. [**Magdalena**]: Subäquatoriale andine Provinz. Sierra Nevada de Sta. Marta, *J. Linden 72* (W-R! 48948; UGDA-DLSz!—drawing).

***Cyrtochilum renisepalum*** Szlach. & Kolan., Syst. Bot. 40: 93. 2015. TYPE: Colombia. *Saravia 1287* (holotype, COL! 227326; UGDA-DLSz!—drawing).

**Figure 63:**
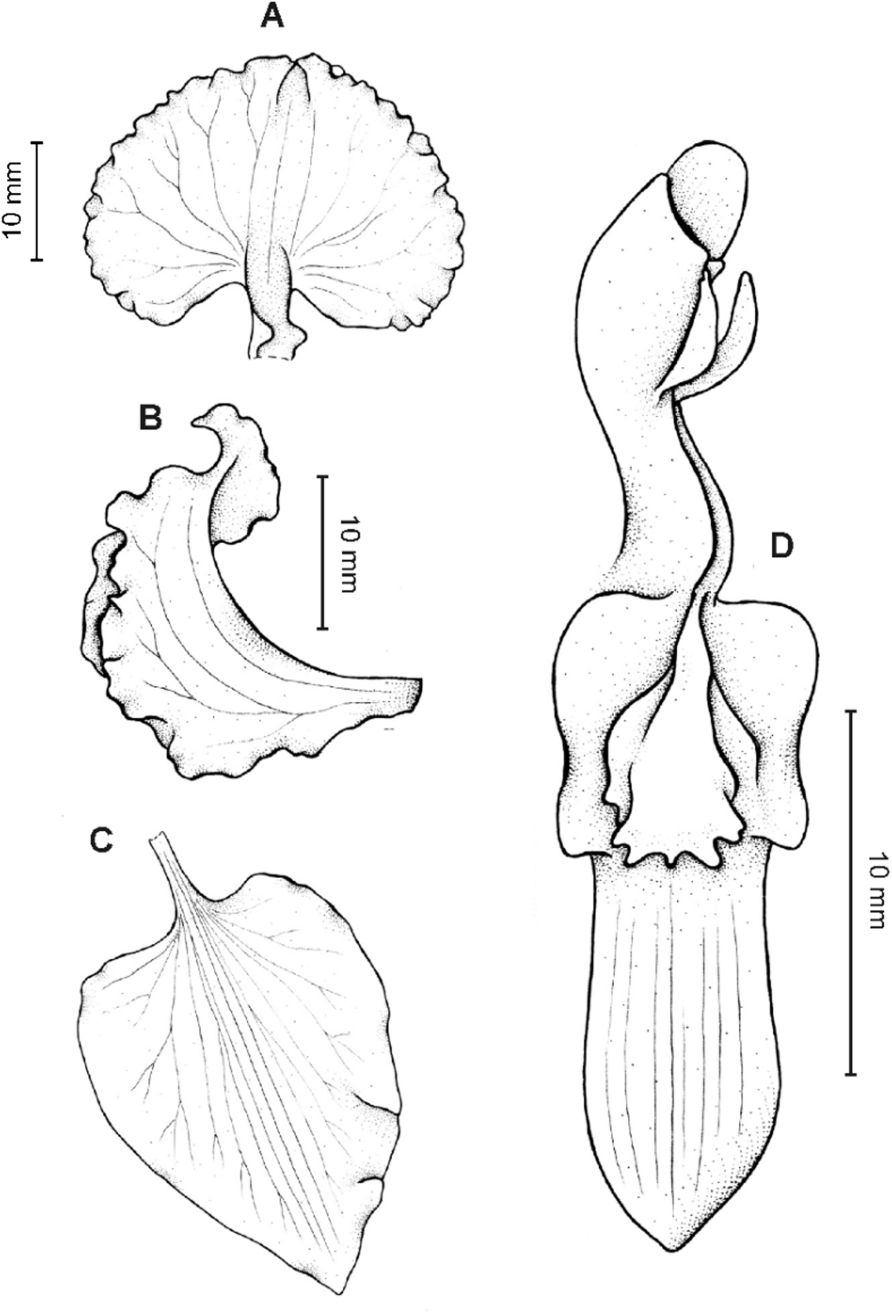
*Cyrtochilum renisepalum* Szlach. & Kolan. (A) Dorsal sepal; (B) Petal; (C) Lateral sepal; (D) Lip and gynostemium. Drawn by Natalia Olędrzyńska from *Saravia 1287* (COL).

Plants long-rhizomatous. Pseudobulbs 10–11 cm long, about 2 cm in diameter, somewhat laterally compressed, more or less elliptic in cross section, concealed basally by leafy sheaths, unifoliate. Leaf about 40 cm long, 5 cm wide, linear-oblanceolate. Inflorescence serpentine, branching. Flowers large, conspicuous. Floral bracts 15 mm long. Pedicel 30 mm long, ovary about 15 mm long. Dorsal sepal shortly clawed; claw 5 mm long, wide, canaliculate, with prominent elliptic-rhombic wings just above the base; blade 25 mm long, 30–32 mm wide, reniform, base subcordate, margins strongly undulate, irregular, apex obtuse, strongly recurved. Petals 26 mm long in total, 20 mm wide when spread, sessile, oblong-elliptic, acute at the apex, strongly falcate, touching each other, margins strongly undulate-serrate. Lateral sepals rather shortly clawed; claw 10 mm long, narrow, without basal wings; blade 35–37 mm long, 27 mm wide, broadly ovate in general outline, truncate basally, acute at the apex, margins almost flat. Lip 17 mm long in total, curved in natural position; basal part 6–7 mm long and wide, almost quadrate, basal part with rounded corners, apical lobes obliquely triangular, callus in the centre, prominent, convex, apically dissected into few projections; apical part 11 mm long, 5–6 mm wide, ligulate, triangular towards the obtuse apex. Gynostemium 11 mm long, sigmoid, connate basally with the lip, lateral appendages lanceolate, falcate and upcurved, not exceeding the anther base (Fig. 63).

*Ecology*: Terrestrial in montane forest.

*Distribution*: Colombia. Alt. 2,100–3,000 m.

*Notes*: *Cyrtochilum renisepalum* resembles *C. baldeviamae*, from which it differs in the structure of perianth segments and gynostemium. Its dorsal sepal is reniform, distinctly wider than long, obtuse at the apex (vs dorsal sepal broadly ovate, acute), lateral sepals are shortly clawed with blade being almost flat along margins (vs lateral sepals long-clawed, margins of blade undulate), lip callus consists of large mass of tissue apically dissected into few projections (vs callus dissected in numerous fragments, flanked by digitate projections) and gynostemium appendages are lanceolate, falcate and upcurved (vs appendages obtriangular, with shallow sinus between apical angles hence appearing bilobed). *Cyrtochilum renisepalum* is somewhat similar to *C. grandiflorum*, but its petals are strongly serrate along margins and touching each other in front of the flower. Additionally the dorsal sepal, lateral sepals and gynostemium appendages are different than in *C. grandiflorum*.

It can be confused with *C. hirtzii*. The latter species, however, has smaller flowers with blade of dorsal sepal 14–16 × 15 mm, and blade of lateral sepals being 20 × 13 mm only. Unlike *C. renisepalum*, the margins of dorsal sepal of *C. hirtzii* are only slightly undulate. The lip callus of *C. hirtzii* consists of central, longitudinal, fleshy, basally low then gradually raised and broadened, several-leveled, flattened structure ending in irregular digitate denticles, with the lateral edges irregularly denticulate and carnose, flanked basally by low, fleshy projecting rigdes.

*Representative specimens*: COLOMBIA. **Cauca**: El Tambo, La Romelia, desde la quebrada El Sopladero a la cabana del UAES PNN, 2,100–2,500 m, 10 October 1995, *N. Ruiz et al. 521* (COL!); PNN Munchique. El Tambo, vereda La Romelia, carretera de entrada al Parque, cerca de la cabana de Inderena, 2,600 m, 22 July 1993, *C. Acevedo et al. CB8558* (COL!). **Cundinamarca**: Cerros de los alrededores de Bogotá, 2,700–3,000 m, 11 June 1962, *C. Saravia 1287* (COL!; UGDA-DLSz!—drawing).

***Cyrtochilum biscellum*** Szlach. & Kolan., ***sp. nov***. TYPE: Colombia. *Lawrence 848* (holotype, AMES! 46827, UGDA-DLSz!—fragment, photo, drawing).

Diagnosis: *This species is somewhat similar to C. hirtzii, but has a papillate lip callus and prominent, 3- or 4-lobed gynostemium appendages. These characters differentiate the new species also from C. renisepalum*.

**Figure 64:**
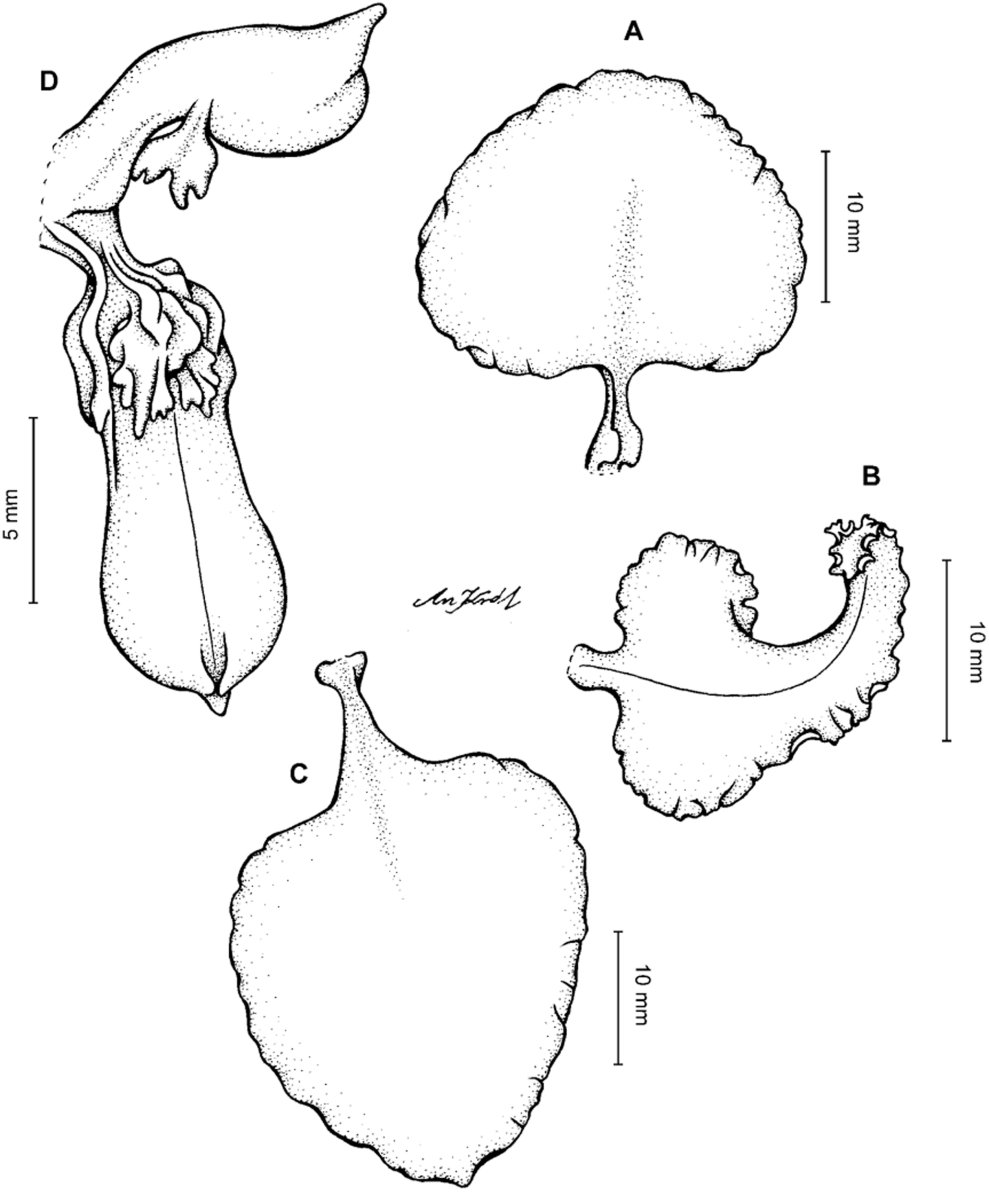
*Cyrtochilum biscellum* Szlach. & Kolan. (A) Dorsal sepal; (B) Petal; (C) Lateral sepal; (D) Lip and gynostemium, side view. Drawn by Anna Król from *Lehmann 8567* (AMES).

Plants long-rhizomatous. Pseudobulbs 10–11 cm long, about 2 cm in diameter, somewhat laterally compressed, more or less elliptic in cross section, concealed basally by leafy sheaths, unifoliate. Leaf about 40 cm long, 5 cm wide, linear-oblanceolate. Inflorescence serpentine, branching, branches loosely 3–4-flowered. Flowers large, conspicuous. Floral bracts 18–20 mm long. Pedicellate ovary 20–35 mm long. Dorsal sepal shortly clawed; claw 6–7 mm long, wide, canaliculate, with prominent elliptic-rhombic wings just above the base; blade 19–23 mm long, 20–32 mm wide, reniform, base truncate, margins undulate, irregular, apex rounded. Petals clawed; claw 3 mm long; blade 22–24 mm long, 11–15 mm wide when spread, oblong-elliptic-ovate, obtuse at the apex, strongly falcate, touching each other, margins strongly undulate-serrate. Lateral sepals clawed; claw 9–14 mm long, narrow, with basal wings; blade 23–30 mm long, 18–25 mm wide, orbicular-ovate in general outline, oblique, truncate basally, obtuse at the apex, margins almost flat. Lip 13–21 mm long in total, curved in natural position; basal part 5–8 mm wide, almost quadrate, with rounded corners; callus in the centre, prominent, papillate on the upper surface, consisting of several almost parallel keels variously wrinkled or divided into knob-like appendages; apical part 8–13 mm long, somewhat narrower than the basal part, ligulate, rounded towards the obtuse, shortly apiculate apex. Gynostemium 9–12 mm long, subarcuate, connate basally with the lip, lateral appendages wing-like or obtriangular, apically dissected into 3–4, unequal lobes (Fig. 64).

*Etymology*: *Ascella—*wing, in reference to the form of gynostemium appendages.

*Ecology*: Terrestrial in high, cold, forested ridges.

*Distribution*: Colombia. Alt. 2,000–3,000 m.

*Notes*: *Cyrtochilum biscellum* is somewhat similar to *C. hirtzii*. The latter is distinguished by glabrous callus and obliquely rhombic wings of gynostemium. The papillate lip callus and prominent, 3- or 4-lobed gynostemium appendages distinguish *C. biscellum* also from *C. renisepalum* which has glabrous lip callus and lanceolate, not dissected gynostemium wings.

*Representative specimens*: COLOMBIA. **Cauca**: Popayán, 2,600–2,900 m, June and July, *F.C. Lehmann 8567* (AMES!, UGDA-DLSz!—fragment, photo, drawing). **Santander**: Velez, 2,000–3,000 m, Novomber 1937, *A.E. Lawrence 848* (AMES!, UGDA-DLSz!—fragment, photo, drawing).

**(6) *Annulare-*group**

Lip narrow, more or less ligulate, apex emarginated.

Six species belonging to this group are known from Colombia and Ecuador.

### Key to the species

1 Blade of dorsal sepal longer than wide, margins of lip apical lobe erose*C. koenigerii*
1* Blade of dorsal sepal as long as wide, or wider, margins of lip apical lobe entire2
2 Lip callus papillate*C. metallicum*
2* Lip callus glabrous3
3 Gynostemium appendages linear, upcurved, acute, lip callus in the form of palmate ridges*C. palmatum*
3* Gynostemium appendages with truncate apex, callus not palmate4
4 Dorsal sepal broadly ovate, margin slightly undulate, gynostemium appendages obliquely rhombic*C. hirtzii*
4* Dorsal sepal reniform, strongly undulate, gynostemium appendages obliquely obtriangular5
5 Lip almost ribbon-like, slightly wider at the apex than at the base, callus consisting of a narrow central ridge flanked by two ridges verrucose along margins*C. orozcoi*
5* Lip oblong-ovate, distinctly wider at the apex than at the base, callus consisting of a massive central ridge, flanked by digitate projections perpendicular to the main ridge*C. annulare*

***Cyrtochilum hirtzii*** Dalström, Fl. Ecuador 87: 103. 2010. TYPE: Ecuador. *Hirtz 1363* (holotype, RPSC).

**Figure 65:**
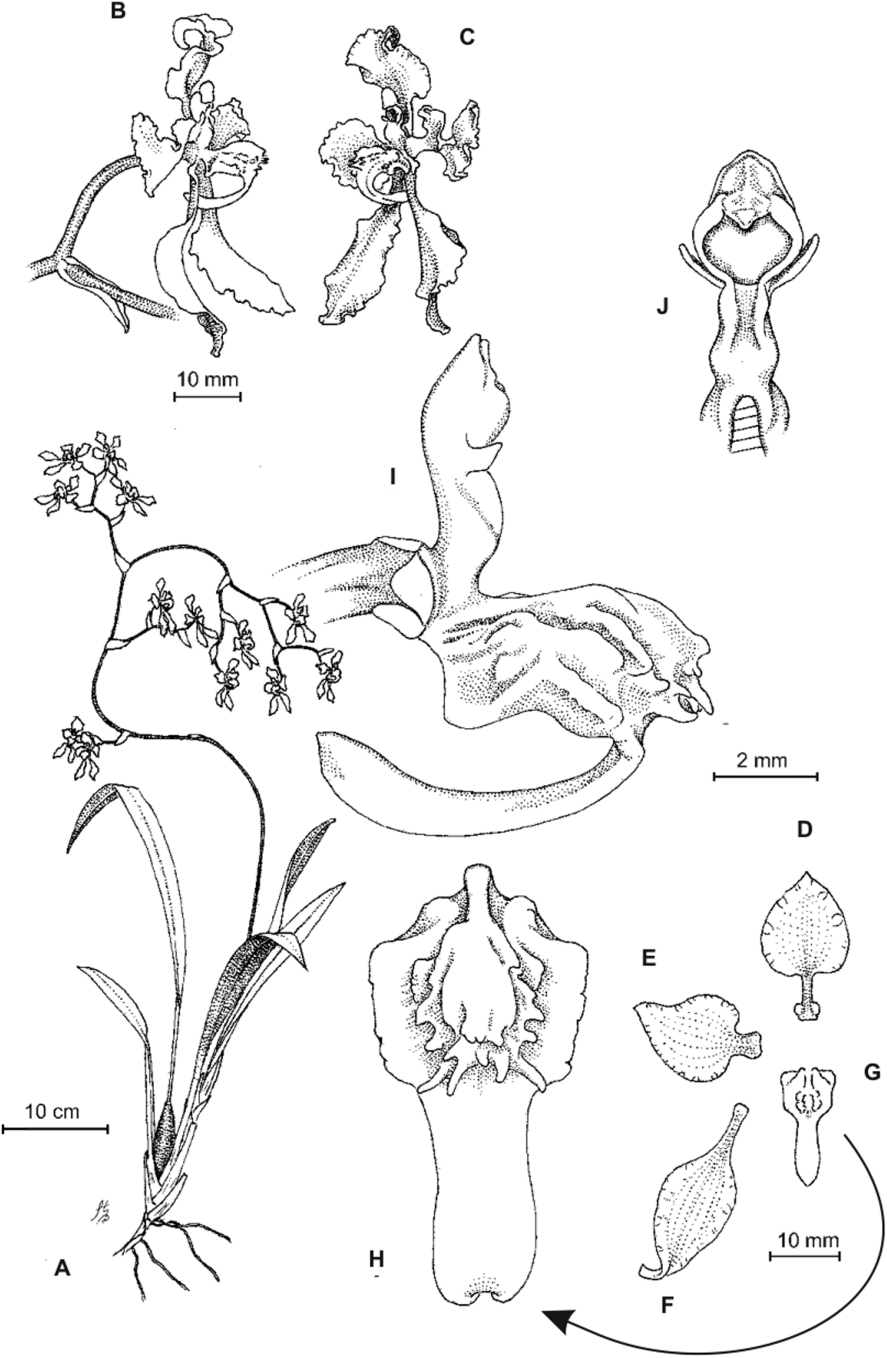
*Cyrtochilum hirtzii* Dalström. (A) Habit; (B) Flower, ovary and floral bract; (C) Flower, side view; (D) Dorsal sepal; (E) Petal; (F) Lateral sepal; (G and H) Lip; (I) Gynostemium and lip, side view; (J) Gynostemium, front view. Drawn by Stig Dalström from *Hirtz 1363* (RPSC).

Rhizome bracteate, creeping. Pseudobulbs 8 cm long, 2 cm wide, distant, oblong-ovoid, unifoliate, surrounded basally by 6–8 distichous sheaths, the uppermost foliaceous. Leaf about 40 cm long, 2 cm wide, subpetiolate, conduplicate, narrowly oblanceolate, acuminate. Inflorescence up to about 75 cm long, axillary, from the uppermost sheaths, erect, then wiry, flexuous, paniculate, with few, widely spaced, weakly flexuous, few-flowered branches. Flower with yellowish-white sepals covered with dark red-brown, whitish petals with irregular red–brown or purplish blotches on basal half, lip yellow at the base, then purple–brown with a white callus. Floral bracts 10–15 mm long, involute and cuculate. Pedicel and ovary 25–30 mm long. Dorsal sepal clawed; claw 6–7 mm long, auriculate; blade 14–16 mm long, 15 mm wide, broadly ovate, shortly acuminate–apiculate, base cordate to truncate, margin slightly undulate. Petals clawed; claw broad, widened at the base; blade up to 20 mm long, 12–14 mm wide, obliquely ovate, shortly acuminate, base cordate to truncate. Lateral sepals clawed; claw about 10 mm long, indistinctly auriculate; blade 20 mm long, 13 mm wide, ovate, slightly oblique, shortly acuminate, cuneate at the base, margin slightly undulate. Lip 18–20 mm long, 8–10 mm wide, cordate to truncate at base, weakly 3-lobed; basal part subrectangular, corners rounded, lateral lobes angular, projecting downwards, fleshy, ridged; callus a central, longitudinal, fleshy, basally low then gradually raised and broadened, several-leveled, flattened structure ending in irregular digitate denticles, with the lateral edges irregularly denticulate and carnose, flanked basally by low, fleshy projecting rigdes; apical part elongate, ligulate, strongly reflexed, slightly canaliculate and emarginate. Gynostemium 8 mm long, erect, clavate, slightly sigmoid, with a bilobular swelling above the base, stigmatic surface with a pair of lateral, obliquely rhombic wings (Fig. 65).

*Ecology*: Epiphytic or terrestrial in cloud forest.

*Distribution*: Ecuador. Alt. 2,600–2,800 m.

*Notes*: *Cyrtochilum hirtzii* is an attractive species, similar to the sympatric *C. halteratum*, but easily distinguished by the more complex, denticulate and digitate callus of the lip. The structure of the callus resembles the lip callus of the equally sympatric *C. cuencanum* and, at this time, the possibility of a natural hybrid origin for *C. hirtzii* cannot be ruled out. Cyrtochilum *cuencanum* differs from *C. hirtzii* by the nonauriculate sepals and the wingless column (Higgins & Dalström, 2011). It is somewhat similar to *C. orozcoi* from which it differs by the dorsal sepal (reniform in *C. orozcoi*), the lip callus (complicated consisting of narrow central ridge flanked by two ridges, apically more or less dissected in *C. orozcoi*) and the gynostemium appendages (obliquely obtriangular in *C. orozcoi*).

*Representative specimens*: ECUADOR. **Tungurahua**: Mt. Tungurahua, 2,800 m, Novomber 1983, *A. Hirtz 1363* (RPSC), Volcán Tungurahua. Alt. 2,600–2,800 m. *A. Hirtz 1362* (SEL—color transparency; Dalström, 2010).

***Cyrtochilum orozcoi*** Szlach. & Kolan., Syst. Bot. 40(1): 88. 2015. TYPE: Colombia. *Orozco et al. 1345* (holotype, COL! 434197; UGDA-DLSz!—drawing).

**Figure 66:**
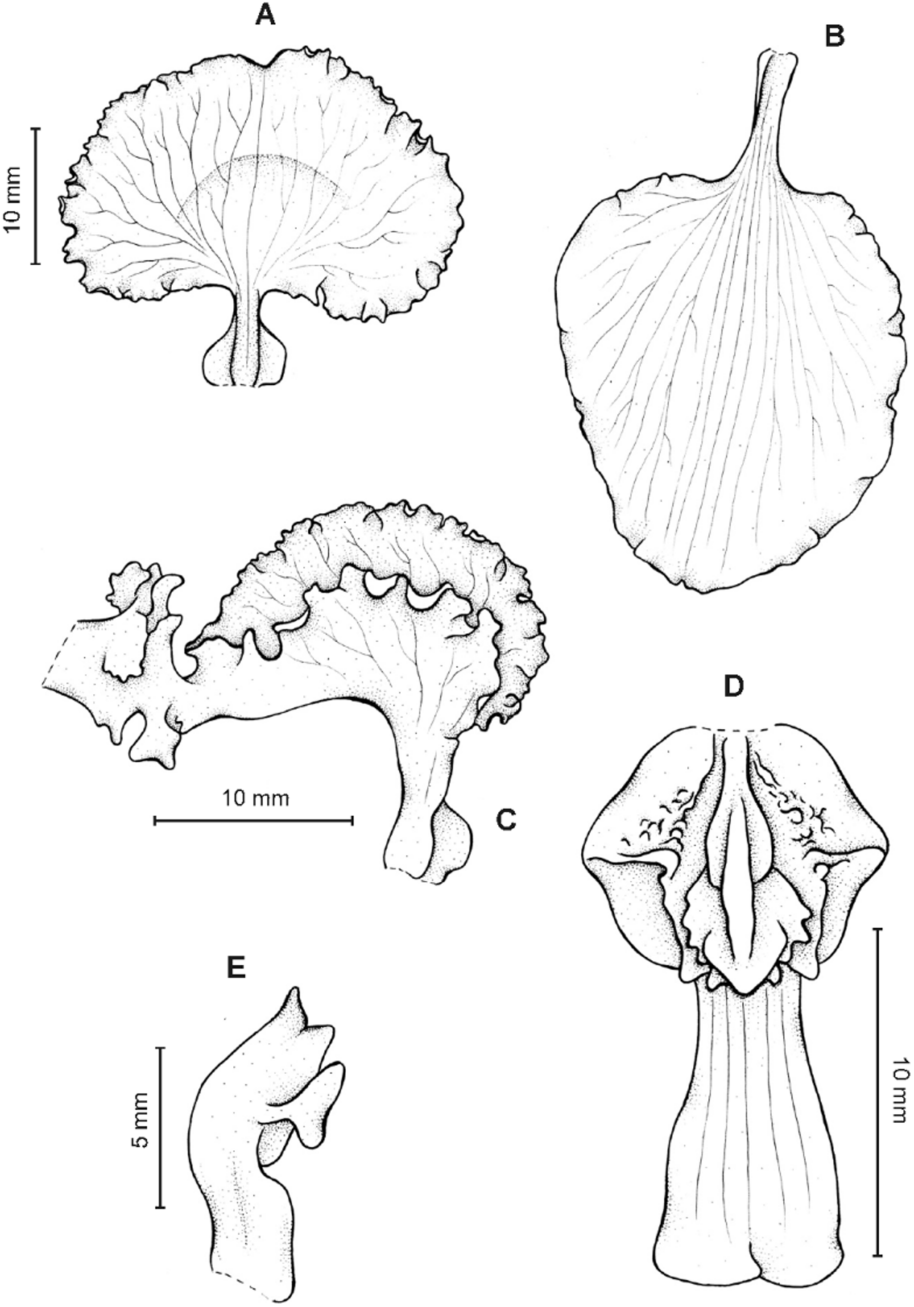
*Cyrtochilum orozcoi* Szlach. & Kolan. (A) Dorsal sepal; (B) Petal; (C) Lateral sepal; (D) Lip; (E) Gynostemium, side view. Drawn by Natalia Olędrzyńska from *Orozco et al. 1345* (COL).

Plants long-rhizomatous. Pseudobulbs up to 14 cm long, about 2.5 cm in diameter, somewhat laterally compressed, more or less elliptic in cross section, concealed basally by leafy sheaths, unifoliate. Leaf about 70 cm long, 5.5 cm wide, linear-oblanceolate, acute. Inflorescence serpentine, branching. Flowers large, conspicuous. Floral bracts 20 mm long. Pedicel 30 mm long, ovary about 10 mm long. Dorsal sepal shortly clawed; claw 6 mm long, rather narrow, canaliculate, with prominent elliptic-rhombic wings just above the base; blade 20–22 mm long, 30 mm wide, reniform, base truncate, margins strongly undulate-serrate, irregular, apex obtuse, strongly recurved. Petals clawed; claw 7 mm long, basally winged; blade 18 mm long, 18 mm wide when spread, oblong-elliptic, acute at the apex, strongly falcate, strongly touching each other, margins strongly undulate-serrate. Lateral sepals shortly clawed; claw 12 mm long, narrow, without basal wings; blade 30 mm long, 25–27 mm wide, obliquely elliptic-ovate in general outline, truncate basally, obtuse at the apex, margins slightly undulate. Lip 17 mm long in total, curved in natural position; basal part 8 mm long and wide, almost rhombic, convex; callus in the center, very complicated consisting of narrow central ridge flanked by two ridges, apically more or less dissected, verrucose along margins; apical part 9 mm long, 5 mm wide, ligulate, somewhat extending towards the apex, truncate, thick. Gynostemium 10 mm long, sigmoid, connate basally with the lip, lateral appendages obliquely obtriangular, not exceeding the anther base (Fig. 66).

*Ecology*: Terrestrial or epiphytic in montane forest.

*Distribution*: Colombia. Alt. 2,000–2,700 m.

*Notes*: *Cyrtochilum orozcoi* resembles *C. annulare*, from which it differs by the lip form. In *C. orozcoi* the lip is almost ribbon-like, slightly wider at the apex than at the base and its callus is very complicated, consisting of a narrow central ridge flanked by two ridges verrucose along margins. On the other hand, the lip of *C. annulare* is oblong-ovate, distinctly wider at the apex than at the base and the lip callus consists of a massive central ridge, flanked by digitate projections perpendicular to the main ridge. *Cyrtochilum orozcoi* is somewhat similar to *C. baldeviamae* from which it differs by the form of the lateral sepals (long-clawed in *C. baldeviamae*, claw up to 20 mm long), gynostemium appendadges (digitate in *C. baldeviamae*) and lip callus (callus side ridges dissected in C. *orozcoi*). Cyrtochilum *orozcoi* can be confused with *C. hirtzii*, but has blade of dorsal sepal much wider than long (vs almost as long as wide), more complicated lip calli verrucose along margins and gynostemium appendages (obliquely obtriangular vs obliquely rhombic).

*Representative specimens*: COLOMBIA. **Antioquia**: Mpio. Urrao. Via hacia Caicedo. Alto del Caicedo. Km 108. En talud a orilla de la carretera, 2,720 m, 4 December 1984, *C. Orozco, L. Tobon & G. Henao 1345* (COL!). Mpio. Salgar. Camino de ascenso a Cerro Plateado, quebrada La Liboriana, 2,200–2,240 m, 3 Novomber 1985, *P. Franco et al. 2260* (COL!). **Cauca**: El Tambo. La Romelia. Desde la quebrada El Sopladero a la cabana del UAES PNN, 2,100–2,500 m, 10 October 1995, *N. Ruiz, G. Lozano & R. Sanchez 521* (COL!). **Cundinamarca**: Bojacá. Vereda San Antonio, 2,600–2,700 m, 28 June 1967, *J. Idrobo 6021* (COL!); Pasca. Vereda de La Mesa, en una pequena ceja de selva, 2,600–2,700 m, 16 September 1967, *L. Uribe Uribe 5967* (COL!); Cabrera. Vereda de Penas Blancas, en el bosque de aserrio, 2,000 m, 21 February 1969, *L. Uribe Uribe 6247* (COL!). **Magdalena**: Sierra Nevada de Santa Marta. Quebrada Cebolleta, 10°55′N 73°57′W, 2,500–2,650 m, 4 August 1972, *J.H. Kirkbride & E. Forero 1880* (COL!, NY). **Santander**: Mpio. Bucaramanga. Via Bucaramanga-Berlin, entre El Divisio y La Corcova, 2,500 m, 29 October 1994, *J.L. Fernandez et al. 11669* (COL!). **Valle Del Cauca**: Palmira, 26 September 1980, *I. Guarin O. 97* (COL!).

***Cyrtochilum annulare*** (Rchb.f.) Kraenzl., Notizbl. Bot. Gart. Berlin-Dahlem 7: 93. 1917. ≡ *Oncidium annulare* Rchb.f., Gard. Chron., n.s., 3: 396. 1875. TYPE: Colombia [New Grenada]. *Charleston s.n*. (syntype: W-R! 48846; UGDA-DLSz!—drawing) & *Roezl s.n*. & *Shuttleworth 13* (syntype: W-R! 48846).

**Figure 67:**
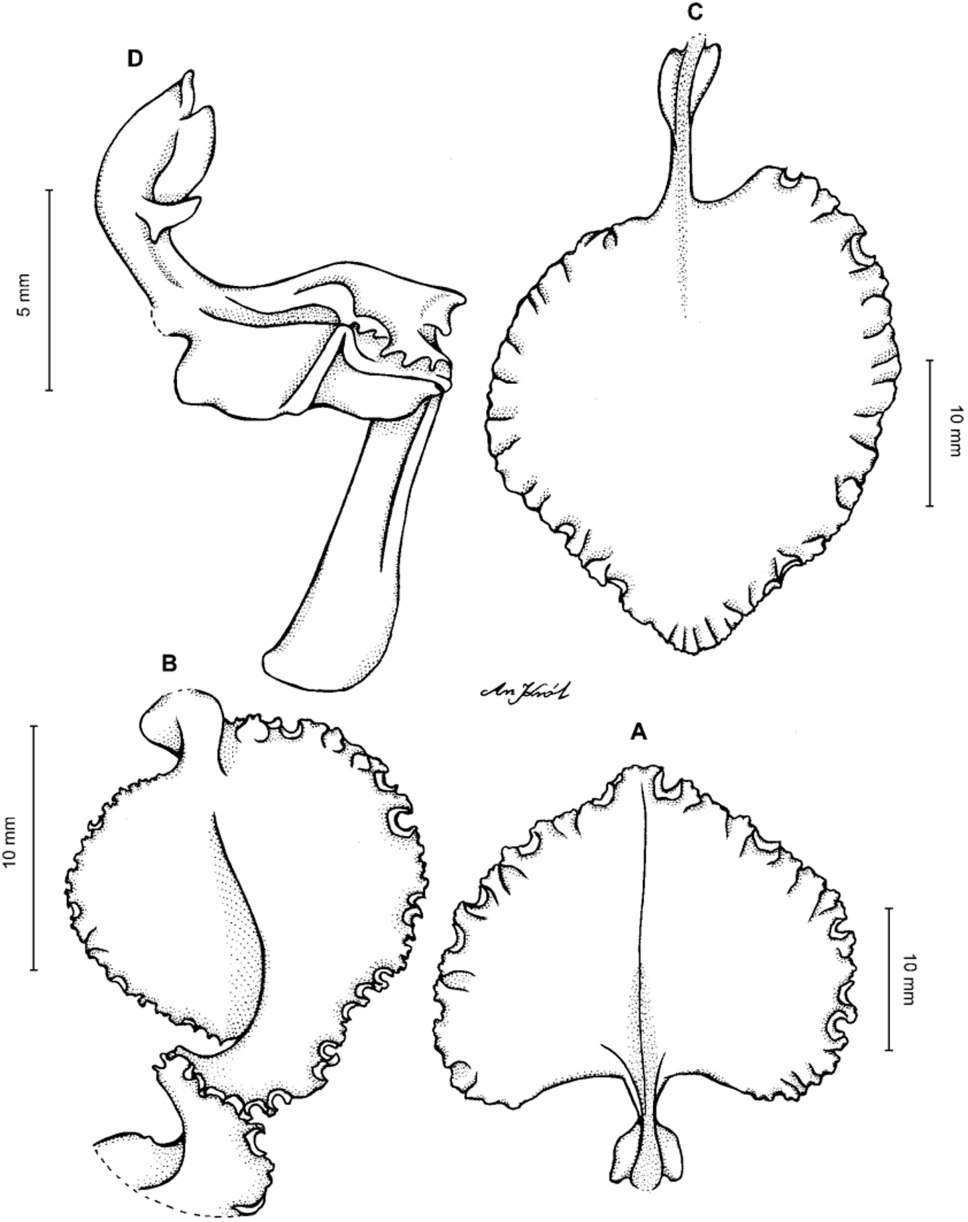
*Cyrtochilum annulare* (Rchb.f.) Kraenzl. (A) Dorsal sepal; (B) Petal; (C) Lateral sepal; (D) Gynostemium and lip, side view. Drawn by Anna Król from *Shuttleworth 13* (W-R).

Plant creeping. Pseudobulbs ovoid, smooth, somewhat compressed. Leaves up to 55 cm long and 4.5 cm wide, oblanceolate, acute. Flowers large, showy. Floral bracts about 15 mm long. Pedicel and ovary about 50 mm long. Dorsal sepal clawed; claw 5–6 mm long, rather narrow, canaliculate, with prominent rhombic wings just above the base; blade 24–30 mm long, 33–36 mm wide, reniform, base subtruncate, margins strongly undulate, irregularly crenate, apex obtuse. Petals broadly clawed; claw about 5–8 mm long, non-winged; blade 19–20 mm long, 15–17 mm wide when spread, oblong elliptic-ovate, sometimes obscurely 3-lobed, obtuse at the apex, strongly falcate, strongly touching each other, margins strongly undulate-serrate. Lateral sepals prominently clawed; claw 10 mm long, narrow, with rather obscure basal wings; blade 30–33 mm long, 28–30 mm wide, obliquely elliptic-cordate in general outline, basally truncate, obtuse at the apex, margins strongly undulate and irregularly crenate. Lip 14–15 mm long in total, curved in natural position to expose large callus; basal part 7 mm long, about 10 mm wide, oblong-elliptic with truncate base, convex; callus very complicated, consisting of a narrow central ridge flanked by two ridges with dissected margins, papillate on the upper surface; apical part 7–8 mm long, 5–6 mm wide, ligulate, somewhat extending towards the apex, truncate, thick. Gynostemium 9 mm long, strongly arcuate, connate basally with the lip, apically somewhat swallen, lateral appendages falcately elliptic-lanceolate, obscurely and obliquely bilobed, not reaching the anther base (Fig. 67).

*Ecology*: Terrestrial in secondary vegetation along exposed roadside or epiphytic in cloud forest.

*Distribution*: Colombia. Alt. 2,600–2,710 m.

*Notes*: From the similar *C. orozcoi* this species differs in the lip form and callus structure (see notes provided under characteristic of *C. orozcoi*).

*Representative specimens*: COLOMBIA. **Antioquia**: Mpio. Urrao. Road between Urrao and Caicedo, 21 km NE of Urrao, near high point on road, secondary vegetation along exposed roadside, 6°24′N 76°02′W, 2,710 m, 27 February 1989, *J. MacDougal, F. Roldan & J. Betancur 4252* (MO!, UGDA-DLSz!—drawing); Epiphytic in cloud forest, Raton Pelado, above Yarumal, 2,650 m, 1 May 1994, *C. Luer, J. Luer, R. Escobar & E. Valencia 10063* (MO!); Páramo de Sonsón. Hacia La Dorada, rastrojo al lado de la carretera, 2,600 m, 20 July 1977, *L. de Escobar & L. Uribe 500* (MO!). **Cauca**: *Sine loc., Charleston s.n*. (W-R!; UGDA-DLSz!—drawing), *B. Roezl s.n*. & *E. Shuttleworth 13* (W-R!).

***Cyrtochilum palmatum*** Szlach. & Kolan., ***sp. nov***. TYPE: Colombia. *Cruz 291* (holotype, COL!, UGDA-DLSz!—drawing).

Diagnosis: *This species is similar to C. hirtzii from which it differs in the broadly deltoid basal lip part and the lip callus consisting of a flat, glabrous, pad-like tissue in the base dissected into some palmate lobes, variously thickened beyond* the *main tissue*.

**Figure 68:**
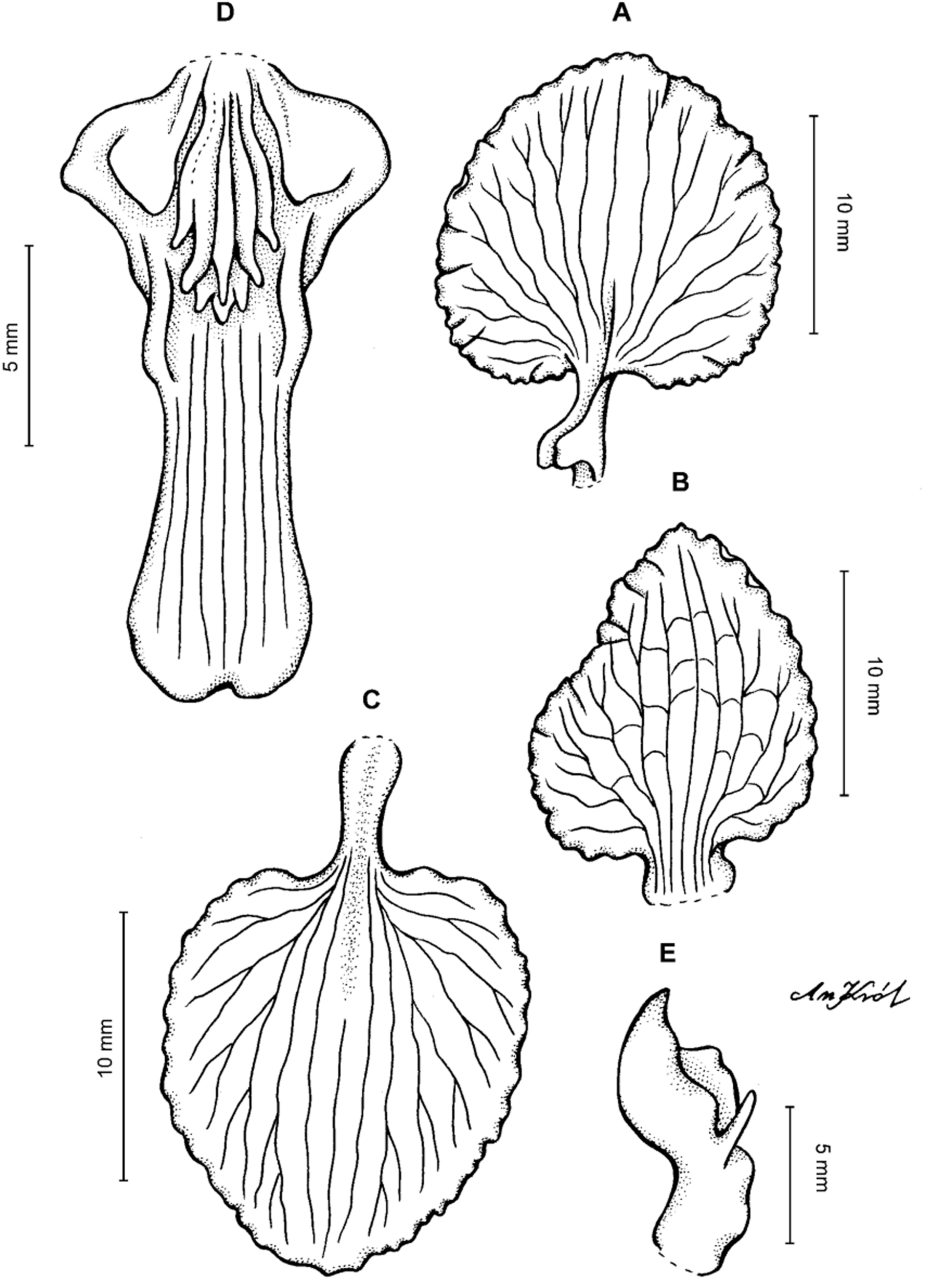
*Cyrtochilum palmatum* Szlach. & Kolan. (A) Dorsal sepal; (B) Petal; (C) Lateral sepal; (D) Lip; (E) Gynostemium, side view. Drawn by Anna Król from *Cruz 291* (COL).

Vegetative parts unknown. Flowers large, conspicuous, showy. Floral bracts 11 mm long. Pedicel and ovary 28 mm long. Dorsal sepal clawed; claw 4 mm long, narrow, canaliculate, with rhombic basal wings; blade 13 mm long, 15 mm wide, suborbicular-reniform, base subcordate, margins irregularly undulate, apex broadly rounded. Petals clawed; claw 3 mm long, wide; blade 15 mm long, 13 mm wide when spread, somewhat oblique, broadly ovate, recurved, base truncate, apex obtuse, margins undulate towards apex. Lateral sepals prominently clawed; claw 5 mm long, narrow, wingless; blade 15 mm long, 13–14 mm wide, obliquely broadly ovate in general outline, basally truncate, rounded at the apex, margins slightly undulate. Lip about 20 mm long in total, curved in natural position above the base; basal part about 7 mm long, about 10 mm wide, broadly deltoid, convex in the centre, corners rounded; callus large, consisting of a flat, glabrous, pad-like tissue in the base dissected into some palmate lobes, variously thickened beyond main tissue; apical part 12–13 mm long, ligulate in the lower part, much extended above to form a deltoid plate about 7 mm wide, truncate to somewhat emarginate at the apex. Gynostemium 5 mm long, sigmoid, connate basally with the lip, lateral appendages digitate, upcurved (Fig. 68).

*Etymology*: In reference to the form of lip callus.

*Ecology*: No data.

*Distribution*: Colombia. Alt. 3,570 m.

*Notes*: *Cyrtochilum palmatum* can be easily distinguished from any other species of this group by having palmate lip callus and digitate gynostemium appendages. In *C. hirtzii* the basal lip part is subrectangular (vs broadly deltoid) and its callus consists of a central, longitudinal, fleshy, basally low then gradually raised and broadened, several-leveled, flattened structure ending in irregular digitate denticles, with the lateral edges irregularly denticulate and carnose, flanked basally by low, fleshy projecting rigdes (vs callus consisting of a flat, glabrous, pad-like tissue in the base dissected into some palmate lobes, variously thickened beyond main tissue).

*Representative specimen*: COLOMBIA. **Boyacá**: Guican. Vereda Tabor, Loma de Llano de la Cruz, 6°27′45.7″ 72°23′24.3″W, 3,570 m, 19 October 1996, *A. Cruz 291* (COL!, UGDA-DLSz!—drawing).

*Conservation status*: This species is described based on material collected more than 20 years ago. We cannot assess the current threats for this taxon hereby the “data deficient” (DD) category should be applied according to IUCN (2001).

***Cyrtochilum metallicum*** (Rchb.f.) Kraenzl., Notizbl. Bot. Gart. Berlin-Dahlem 7: 94. 1917. ≡ *Oncidium metallicum* Rchb.f., Gard. Chron., n.s., 5: 394. 1876. TYPE: Colombia. *Wallis 412* (holotype, W-R! 48950, UGDA-DLSz!—drawing).

**Figure 69:**
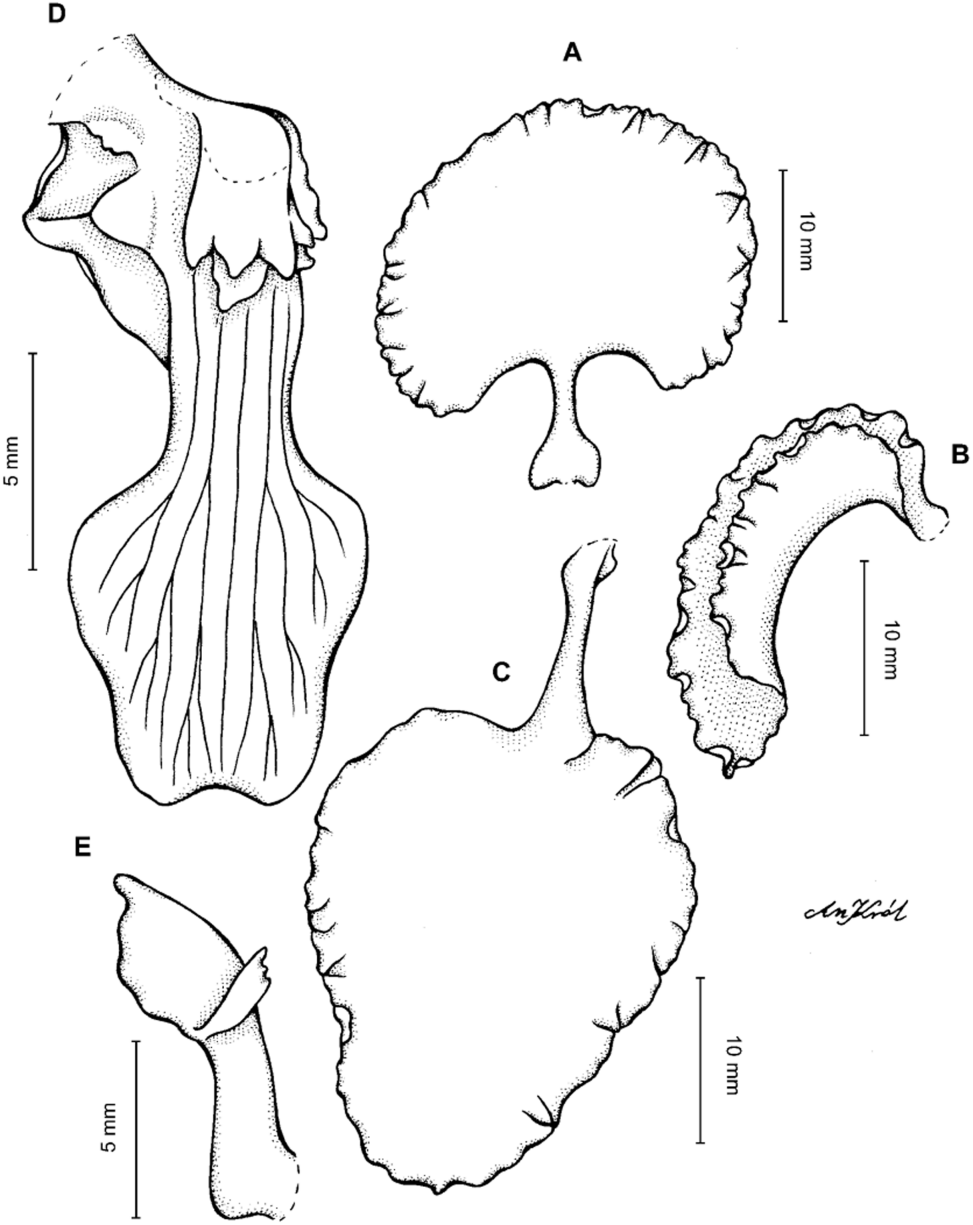
*Cyrtochilum metallicum* (Rchb.f.) Kraenzl. (A) Dorsal sepal; (B) Petal; (C) Lateral sepal; (D) Lip; (E) Gynostemium, side view. Drawn by Anna Król from *Wallis 412* (W-R).

Flowers large, conspicuous, showy. Floral bracts 15 mm long. Pedicel and ovary 50 mm long. Dorsal sepal clawed; claw 6 mm long, narrow, canaliculate, with triangular-ovate wings just above the base; blade 17 mm long, 24 mm wide, reniform, base subcordate, margins irregularly undulate, apex broadly rounded. Petals clawed; claw about 2–3 mm long, wide; blade 17–18 mm long, 14 mm wide when spread, somewhat oblique, broadly elliptic-ovate, recurved, base truncate, apex obtuse, margins irregularly undulate-serrate. Lateral sepals prominently clawed; claw 10 mm long, narrow, with basal obscure wings on both margins; blade 34 mm long, 23 mm wide, obliquely broadly ovate in general outline, basally truncate, rounded at the apex, margins slightly undulate. Lip about 20 mm long in total, curved in natural position above the base; basal part about 7 mm long, about 10 mm wide, broadly deltoid, convex in the centre, callus large, consisting of a flat, papillate, pad-like tissue in the base dissected into some apical lobes, variously thickened beyond main tissue; apical part 12–13 mm long, ligulate in the lower part, much extended above to form a deltoid plate about 7 mm wide, truncate to somewhat emarginate at the apex. Gynostemium 10 mm long, subarcuate, connate basally with the lip, lateral appendages recurved, obliquely oblanceolate, apex truncate, irregularly dentate, not reaching the anther base (Fig. 69).

*Ecology*: No data.

*Distribution*: Colombia.

*Notes*: This is the only species of this group with a papillate callus and irregularly dentate gynostemium appendages.

*Representative specimen*: COLOMBIA. *Sine loc., G. Wallis 412* (W-R! 48950, UGDA-DLSz!—drawing).

***Cyrtochilum koenigerii*** Szlach. & Kolan., ***sp. nov***. TYPE: Ecuador. *Neill & Cero 7485* (holotype, MO!, UGDA-DLSz!—drawing).

Diagnosis: *This species resembles C. metallicum from which it differs in the transversely elliptic lip base and glabrous lip callus consisting of a median keel flanked by 5–6 keels on each side and two wings at the apex*.

**Figure 70:**
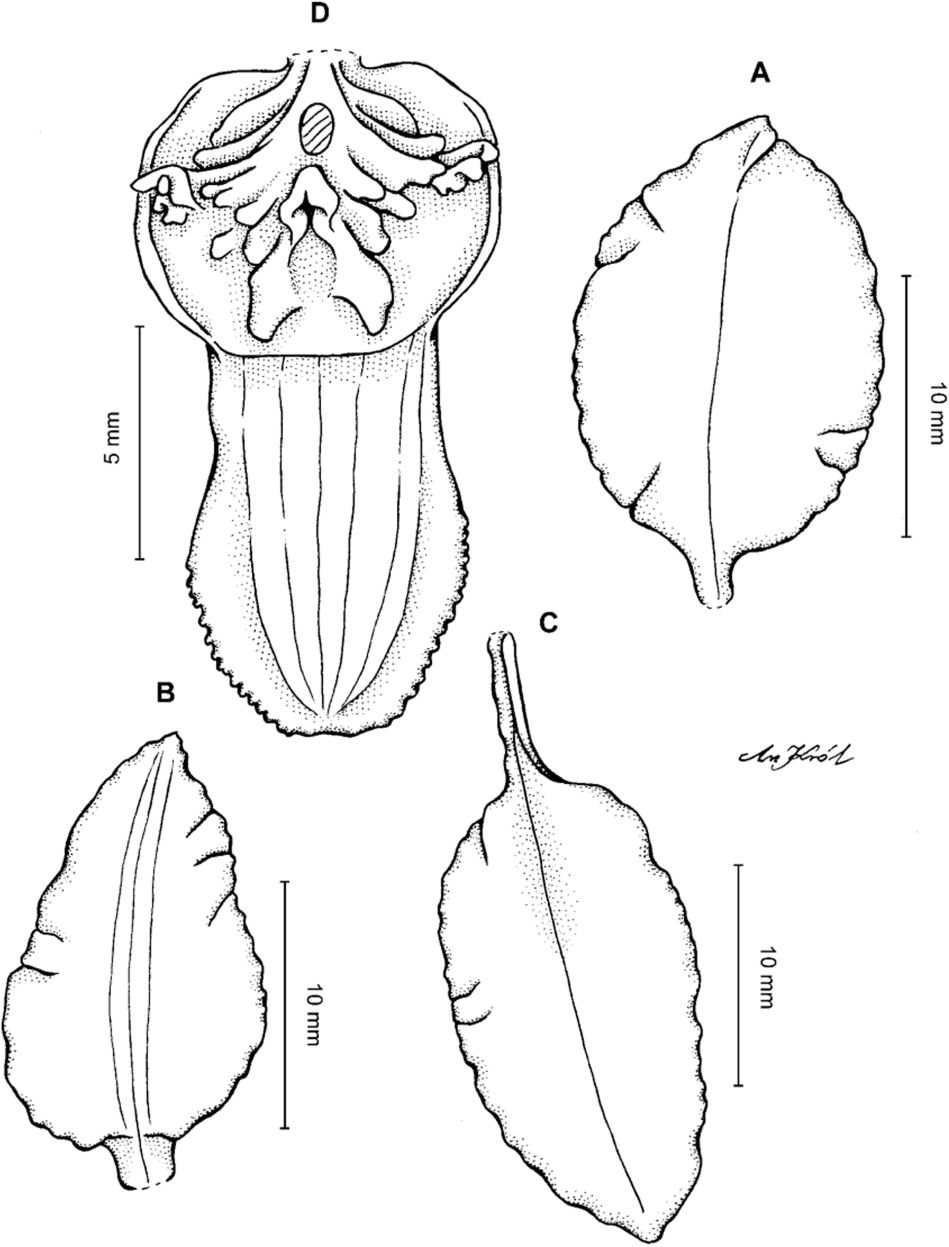
*Cyrtochilum koenigerii* Szlach. & Kolan. (A) Dorsal sepal; (B) Petal; (C) Lateral sepal; (D) Lip. Drawn by Anna Król from *Neill & Cero 7485* (MO).

Pseudobulbs 12 cm long, 1 cm wide, fusiform, enclothed basally by leafy sheaths, 3-foliate. Leaves up to 70 cm long and 3.5 cm wide, linear-oblanceolate, acuminate. Flowers large, conspicuous, showy. Floral bracts 7 mm long. Pedicel and ovary 25 mm long. Dorsal sepal clawed; claw 5 mm long, narrow, canaliculate, wingless; blade 19 mm long, 14 mm wide, elliptic, base cuneate, margins weakly undulate, apex obtuse. Petals clawed; claw 3 mm long, wide; blade 18 mm long, 12–13 mm wide when spread, somewhat oblique, elliptic-ovate, somewhat pandurate, base cuneate, apex obtuse, margins slightly undulate. Lateral sepals prominently clawed; claw 8 mm long, narrow, canaliculate, wingless; blade 22 mm long, 14 mm wide, obliquely oblong elliptic in general outline, basally cuneate, apex obtuse, margins slightly undulate. Lip about 15 mm long in total, curved in natural position above the base; basal part about 7 mm long, about 9 mm wide, transversely elliptic, convex in the centre, corners rounded, base truncate; callus large, consisting of a median keel flanked by 5–6 keels on each side and 2 wings at the apex, glabrous, additional outgrowths beyond main tissue; apical part 8 mm long, 7 mm wide, ligulate, truncate at the apex, apical margins erose. Gynostemium unknown (Fig. 70).

*Etymology*: Dedicated to Willibald Koniger, a German orchidologist, working on Oncidiinae.

*Ecology*: No data.

*Distribution*: Ecuador. Alt. 1,500 m.

*Notes*: The unique characters of this taxon are the elliptic dorsal sepal, oblong elliptic lateral sepals, elliptic-ovate, somewhat pandurate petals and erose margins of lip apical lobe. Unlike in the new species the lip of *C. metallicum* is broadly deltoid at the base (vs transversely elliptic), ornamented with a large callus consisting of a flat, papillate, pad-like tissue in the base dissected into some apical lobes, variously thickened beyond main tissue (vs callus consisting of a median keel flanked by 5–6 keels on each side and 2 wings at the apex, glabrous).

*Conservation status*: This species is described based on material collected more than 30 years ago. We cannot assess the current threats for this taxon hereby the “data deficient” (DD) category should be applied according to IUCN (2001).

*Representative specimens*: ECUADOR. **Zamora-Chinchipe**: 15 km above Zamora on road to Loja, 4°00′S 79°01′W, 1,500 m, 9 Novomber 1986, *D. Neill & C. Cero 7485* (MO!, UGDA-DLSz!—drawing).

**(7) *Trilamellatum*-group**

Lip oblong-ovate, callus consisting of 3 main lamellae variously shaped.

Over 10 species are known mostly from Colombian and Ecuadorian Andes.

### Key to the species

1 Lip callus consisting of 3 parallel lamellae similar in size2
1* Lip callus consisting of 3 lamellae, from which the median one is prominently larger than both laterals3
2 Lip base cuneate, callus apically enlarged and irregularly dissected with digitate outgrowth at the base, gynostemium lateral appendages digitate*C. mendax*
2* Lip base truncate, callus apically extended, flanked in the basal part by narrow keels with free apex, gynostemium lateral appendages obliquely oblanceolate, with truncate apex, unequally 3-lobulate*C. tucumanense*
3 Gynostemium appendages obscure4
3* Gynostemium appendages prominent6
4 Lip callus with the median keel terminated with extended nose-like process, branching basally into digitate lobules, with a series of additional, irregular thickenings on both sides of central callus*C. orgyale*
4* Lip callus somewhat nose-like, apically bilobulate, flanked by short, triangular keels on both sides, apically bilobed, with an additional thickenings beyond the main callus5
5 Lip subrectangular-ovate in general outline, apical part linear-triangular, attenuate towards subacute apex*C. undulatum*
5* Lip pandurate-elliptic, with weakly bilobed to minutely apiculate apical lobe*C. violaceum*
6 Gynostemium lateral appendages obovate, apically truncate and crenate*C. pastasae*
6* Gynostemium lateral appendages linear to lanceolate-falcate7
7 Main lip calli covered basally with two, incurved folds along fusion line between gynostemium and lip*C. geniculatum*
7* Lip calli without any basal folds8
8 Petals with a pair of basal wings*C. halteratum*
8* Petals wingless9
9 Dorsal sepal more or less reniform, base cordate to cordate-sagittate, blade wider than long, petals blade wider than long10
9* Dorsal sepal more or less ovate, base cuneate, longer than wide, petals longer than wide11
10 Lateral sepals subcordate-ovate, basal lip part as long as apical one*C. maasii*
10* Lateral sepals elliptic-ovate in outline, base truncate, basal lip part longer than apical one*C. kraenzlinianum*
11 Gynostemium appendages falcate-subulate, acute*C. portillae*
11* Gynostemium appendages obliquely oblanceolate, with oblique, truncate apex*C. plagianthum*

***Cyrtochilum mendax*** (Rchb.f.) Kraenzl., Notizbl. Bot. Gart. Berlin-Dahlem 7: 92. 1917. ≡ *Oncidium mendax* Rchb.f., Flora 69: 549. 1886. TYPE: Cult? *Lehmann s.n*. (holotype, W-R! 13874, UGDA-DLSz!—drawing).

**Figure 71:**
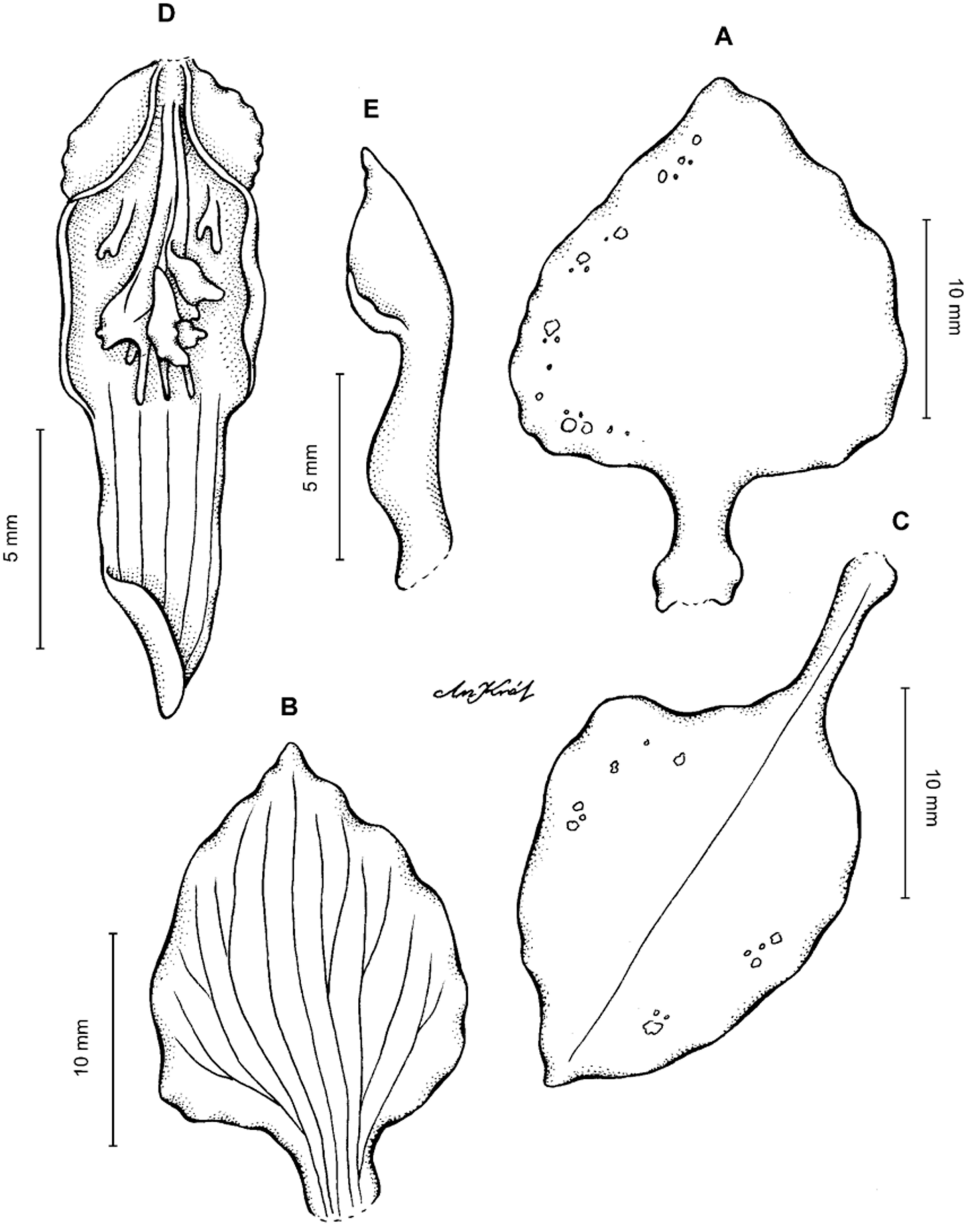
*Cyrtochilum mendax* (Rchb.f.) Kraenzl. (A) Dorsal sepal; (B) Petal; (C) Lateral sepal; (D) Lip; (E) Gynostemium, side view. Drawn by Anna Król from *Lehmann s.n*. (W-R).

Pseudobulbs up to 10 cm long and 3.5 cm wide, oblong ovoid, bifoliate. Inflorescence about 50 cm long, paniculate, wiry, with widely spaced few-flowered branches. Flowers rather large, conspicuous, showy, sepals whitish covered by brown, petals white, covered basally by pink, then with red-purple irregular blotches, lip whitish with pinkish-brown suffusion, callus pink-white. Floral bracts 15–17 mm long. Pedicel and ovary 20–25 mm long. Dorsal sepal clawed; claw 4–6 mm long, narrow, canaliculate, with rather obscure obliquely ovate wings just above the base; blade 16–17 mm long, 12–16 mm wide, broadly triangular-ovate to suborbicular-ovate, base truncate, margins entire with crystals, apex rounded to obtuse. Petals clawed; claw about 2 mm long, wide; blade 16 mm long, 12 mm wide when spread, somewhat oblique, broadly ovate-suborbicular, base truncate, attenuate towards the apex, margins entire. Lateral sepals prominently clawed; claw 5–6 mm long, narrow, with basal obscure wing on the outer margin; blade about 15–20 mm long, 11 mm wide, obliquely elliptic-ovate in general outline, basally cuneate-truncate, subacute at the apex, margins entire. Lip 15–18 mm long in total, lanceolate-ligulate, curved in natural position near the middle; basal part somewhat expanded about 4–5.6 mm wide, oblong elliptic, cuneate at base; callus consisting of 3 parallel lamellae apically enlarged and irregularly dissected with digitate outgrowths at the base, furnished with an additional projections beyond main callus; apical part 7 mm long, about 3 mm wide, ligulate, attenuate at the apex. Gynostemium 10–12 mm long, subarcuate, connate basally with the lip, lateral appendages obliquely digitate, upcurved, not reaching the anther base (Fig. 71).

*Ecology*: Epiphyte in forest, on rocky stream bank.

*Distribution*: Colombia, Ecuador. Alt. 1,600–3,000 m.

*Notes*: This species appears to be closely related to *C. tucumanense*, from which it can be separated by the cuneate lip base (vs truncate), a 3-lamellate callus apically enlarged and an irregularly dissected with digitate outgrowth at the base (vs 3 parallel lamellae apically extended, flanked in the basal part by narrow keels with free apex) and obliquely digitate lateral appendages (vs obliquely oblanceolate, with truncate apex, unequally 3-lobulate).

*Representative specimens*: COLOMBIA. **Caldas**: Rio San Rafael, below Cerro Tatama, 2,600–2,800 m, 7–11 September 1922, *F. W. Pennell 10368* (AMES!, UGDA-DLSz!—drawing); the same loc., *F. Pennell 10367* (AMES!, UGDA-DLSz!—drawing). **Cauca**: El Tambo, La Romelia, desde la quebrada El Sopladero a la cabana del UAES PNN, 2,100–2,500 m, 10 October 1995, *N. Ruiz, G. Lozano & R. Sanchez 521* (COL!); PNN Munchique. El Tambo, vereda La Romelia, carretera de entrada al Parque, cerca de la cabana de Inderena, 2,600 m, 22 July 1993, *C. Barbosa & al. CB8558* (COL!); Popayán, 2,000–2,600 m, *F.C. Lehmann 6243* (AMES!, UGDA-DLSz!—fragment, photo, drawing). **Cundinamarca**: Cerros de los alrededores de Bogotá, 2,700–3,000 m, 11 June 1962, *C. Saravia 1287* (COL!). Cult? *F.C. Lehmann s.n*. (W-R! 13874, UGDA-DLSz!—drawing). ECUADOR. **Chimborazo**: Sibambe, 1,600–2,200 m, July–September, *F.C. Lehmann 8565* (NY—Dalström, 2010).

***Cyrtochilum tucumanense*** (Rchb. f.) Kraenzl., Notizbl. Bot. Gart. Berlin-Dahlem 7: 94. 1917. ≡ *Oncidium tucumanense* Rchb.f., Linnaea 41: 23. 1877. TYPE: Argentina [Bolivia?]. *Pearce 803* (holotype, W-R! 48945, UGDA-DLSz!—drawing). ≡ *Cyrtochilum trilamellatum* Kraenzl., Notizbl. Bot. Gart. Berlin-Dahlem 7: 99. 1917. *nom. illeg. superfl*.

**Figure 72:**
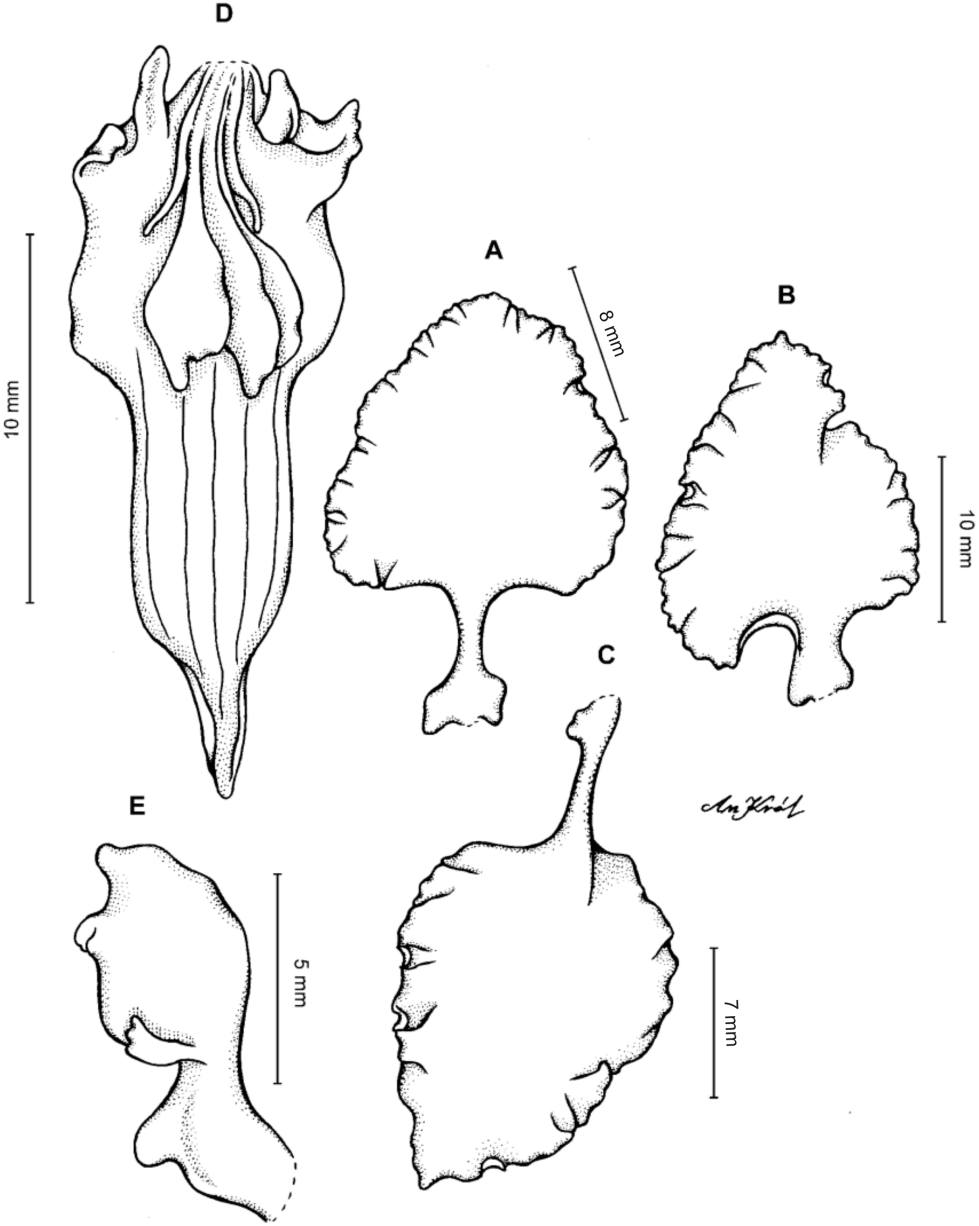
*Cyrtochilum tucumanense* (Rchb. f.) Kraenzl. (A) Dorsal sepal; (B) Petal; (C) Lateral sepal; (D) Lip; (E) Gynostemium, side view. Drawn by Anna Król from *Pearce 803* (W-R).

Flowers conspicuous, showy. Floral bracts 15–17 mm long. Pedicel and ovary 32–35 mm long. Dorsal sepal clawed; claw 6–7 mm long, narrow, canaliculate, with prominent rhombic wings just above the base; blade 13–14 mm long, 11–15 mm wide, elliptic-ovate to reniform, base truncate, margins undulate, crenate, apex rounded. Petals clawed; claw about 4 mm long, wide; blade 14–18 mm long, 11–12 mm wide when spread, somewhat oblique, broadly ovate, base truncate or subcordate, attenuate towards obtuse apex, margins crenate-undulate. Lateral sepals prominently clawed; claw 5–7 mm long, narrow, with basal rhombic wing on the outer margin; blade about 18 mm long, 13–15 mm wide, obliquely elliptic-ovate in general outline, basally truncate, subacute to subobtuse at the apex, margins crenate-undulate. Lip 20 mm long in total, 6–8 mm wide, lanceolate-ligulate, curved in natural position above the base; basal part somewhat expanded, rectangular, apical corners obtuse, basal corners auriculate, base truncate, with an additional ligulate projections; callus consisting of 3 parallel lamellae apically extended, flanked in the basal part by narrow keels with free apex; apical part ligulate, attenuate at the apex. Gynostemium 9–10 mm long, sigmoid, connate basally with the lip, lateral appendages obliquely oblanceolate, apex truncate, unequally 3-lobulate, with prominent knob-like outgrowth just below stigma (Fig. 72).

*Ecology*: Terrestrial or epiphyte.

*Distribution*: Colombia, Bolivia (or Argentina).

*Notes*: This species is easy to distinguish by peculiar gynostemium structure, i.e. oblanceolate lateral appendages with truncate, shallowly and unequally 3-lobulate apex and knob-like outgrowth just below stigma. It can be separated from similar *C. mendax* also by the lip form, which has truncate, auriculate base (vs cuneate) with additional ligulate projections and simpler callus.

*Representative specimens*: COLOMBIA. Bogotá, *H. Karsten s.n*. (W!). [New Grenada], *J. Warszewicz s.n*. (W-R! 36994, UGDA-DLSz!—drawing).

*Other specimens examined*: ARGENTINA [BOLIVIA?]. Taumana. *G. Pearce 803* (W-R).

***Cyrtochilum orgyale*** (Rchb.f. & Warsz.) Kraenzl., Notizbl. Bot. Gart. Berlin-Dahlem 7: 92. 1917. ≡ *Oncidium orgyale* Rchb.f. & Warsz., Bonplandia (Hannover) 2: 102. 1854. TYPE: Colombia [New Grenada]. *Warszewicz s.n*. (holotype, W-R! 36994, UGDA-DLSz!—drawing).

**Figure 73:**
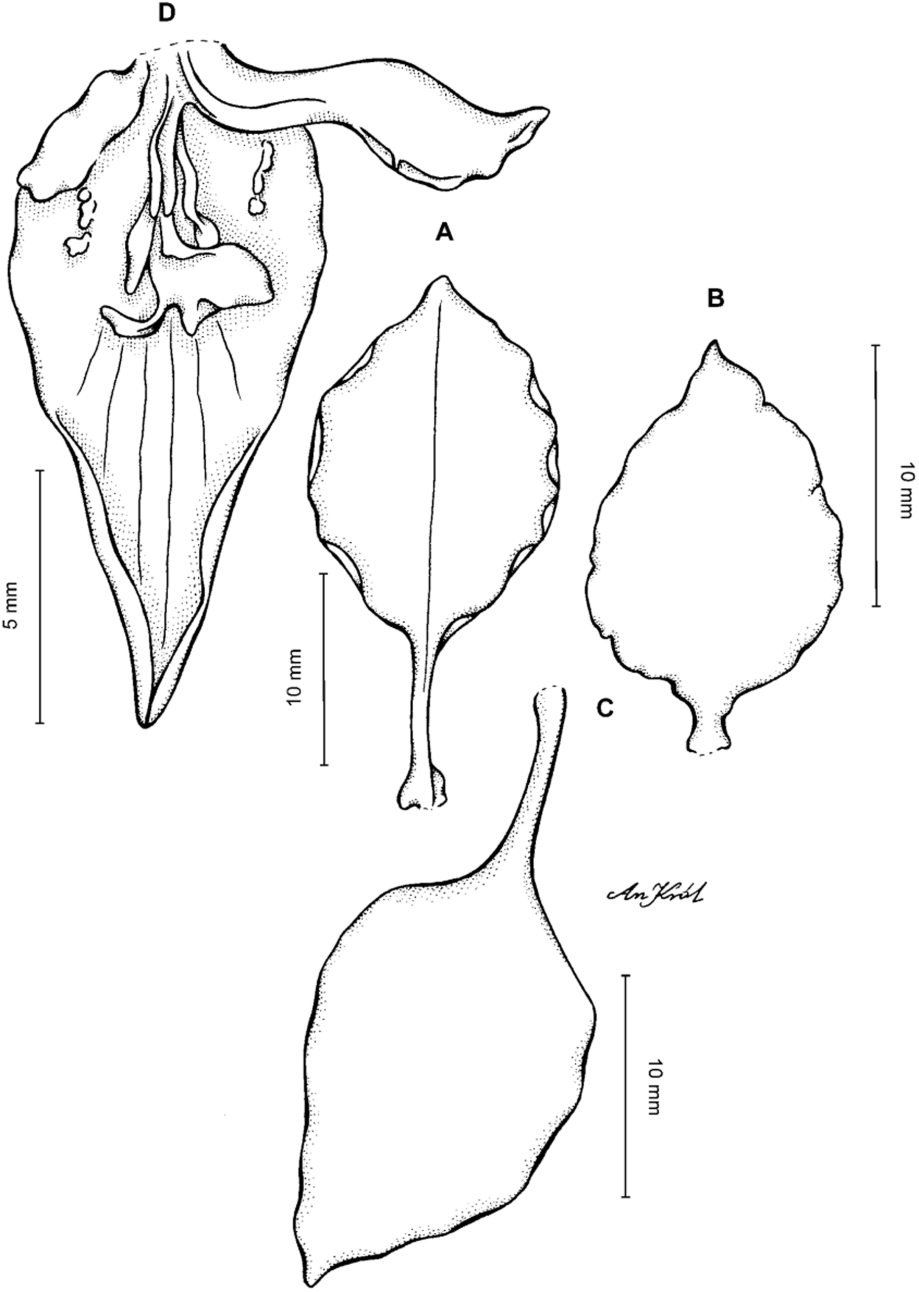
*Cyrtochilum orgyale* (Rchb.f. & Warsz.) Kraenzl. (A) Dorsal sepal; (B) Petal; (C) Lateral sepal; (D) Lip and gynostemium. Drawn by Anna Król from *Warszewicz s.n*. (W-R).

Pseudobulbs up to 13 cm long and 2–3 cm wide, fusiform to oblong ovoid, basally enclothed with leafy sheaths, bifoliate. Leaves up to 40 cm long and 2.5 cm wide, linear-oblanceolate, acuminate. Inflorescence wiry, branching, densely many-flowered. Flowers rather large, conspicuous, showy. Floral bracts 8–10 mm long. Pedicel and ovary 20–30 mm long. Dorsal sepal clawed; claw 6 mm long, narrow, canaliculate, with obscure triangular-ovate wings just above the base; blade 13–21 mm long, 9–17 mm wide, elliptic, base cuneate, margins entire, somewhat undulate, apex subacute. Petals clawed; claw about 2 mm long, wide; blade 12–18 mm long, 10–16 mm wide when spread, oblique, ovate, acute, base broadly cuneate, margins entire, distantly undulate. Lateral sepals prominently clawed; claw 5–6 mm long, narrow, wingless; blade 14–20 mm long, 8–16 mm wide, obliquely ovate in general outline, basally cuneate, apex subacute, margins entire. Lip 13–15 mm long in total, 5–7 mm wide at the base, gently curved in natural position just above the base, oblong ovate-lanceolate in general outline, base truncate, with small knob-like extension on both sides, attenuate towards the apex, acute, convex in the lower part; callus large, consisting of 3 parallel keels, of which the median one is terminated with extended nose-like process, branching basally into digitate lobules, with a series of additional, irregular thickenings on both sides of central callus. Gynostemium 7 mm long, subsigmoid, connate basally with the lip, lateral appendages rudimentary (Fig. 73).

*Ecology*: Terrestrial or epiphyte.

*Distribution*: Colombia.

*Notes*: This species can be easily identified from other taxa of this group by having rudimentary gynostemium lateral appendages and a callus with the median keel being the largest and extended in a nose-like shape. A similar gynostemium form with rudimentary appendages can be found also in *C. undulatum*. The species, however, has a different lip callus which is flanked by short, triangular keels on both sides, apically bilobed, with additional thickenings beyond the main callus.

*Representative specimens*: COLOMBIA. **Cundinamarca**: Bogotá, *H. Karsten s.n*. (W!); Above Salto de Tequendama, 23 July 1965, *F.A. Barkley & J.H. Torres-Romero 35341* (AMES!, UGDA-DLSz!—fragment, photo, drawing). [New Grenada], *J. Warszewicz s.n*. (W-R! 36994).

***Cyrtochilum undulatum*** Kunth *in* F.W.H. von Humboldt, A.J.A. Bonpland & C.S. Kunth, Nov. Gen. Sp. 1: 349. 1816. TYPE: Colombia [New Grenada]. *Humboldt s.n*. (holotype, P; fragment of type: W-R! 36987; UGDA-DLSz!—drawing).

**Figure 74:**
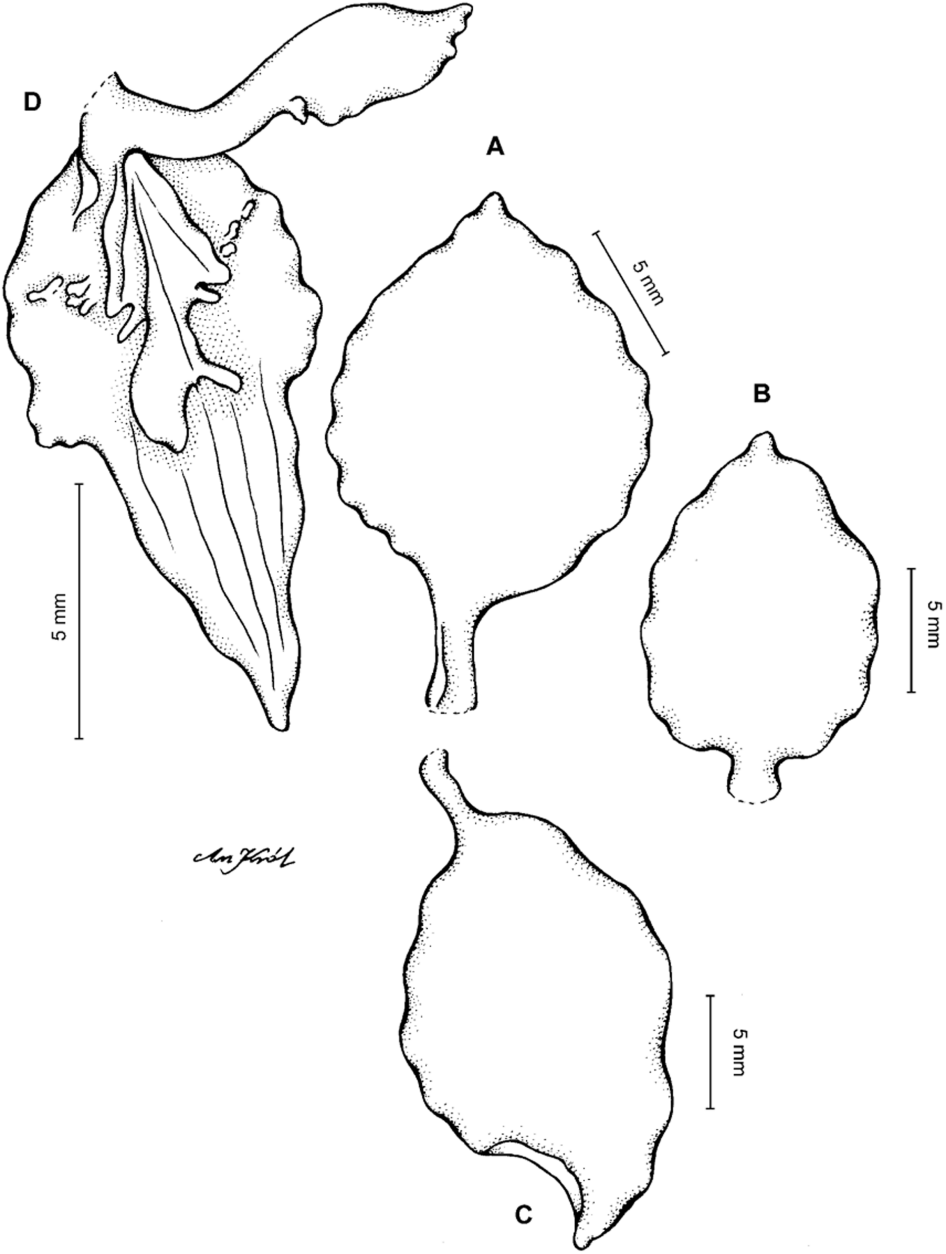
*Cyrtochilum undulatum* Kunth. (A) Dorsal sepal; (B) Petal; (C) Lateral sepal; (D) Lip and gynostemium. Drawn by Anna Król from *Humboldt s.n*. (W-R).

Flowers rather small. Floral bracts 18 mm long. Pedicel and ovary 38 mm long. Dorsal sepal clawed; claw 3–5 mm long, narrow, canaliculate, with obscure wings just above the base; blade 11–17 mm long, 9–12 mm wide, suborbicular-ovate, margins entire, somewhat undulate, base cuneate, apex subobtuse. Petals clawed; claw about 2 mm long, wide; blade 11–14 mm long, 8–9 mm wide when spread, obliquely elliptic-ovate, base truncate, apex obtuse, margins entire, somewhat undulate. Lateral sepals prominently clawed; claw 4–6 mm long, narrow, wingless; blade 15–16 mm long, 8–13 mm wide, obliquely elliptic-ovate in general outline, apex obtuse, margins entire, slightly undulate. Lip 12–13 mm long in total, gently curved in natural position; basal part 5–6 mm long and wide, subrectangular-ovate in general outline, base broadly cuneate; callus prominent consisting of a prominent, median keel, somewhat nose-like, apically bilobulate, flanked by short, triangular keels on both sides, apically bilobed, with additional thickenings beyond the main callus; apical part 6 mm long, 3.5 mm wide, linear-triangular, attenuate towards subacute apex. Gynostemium 8 mm long, subarcuate, connate basally with the lip, lateral appendages rudimentary, upcurved (Fig. 74).

*Ecology*: Epiphyte.

*Distribution*: Colombia.

*Notes*: *Cyrtochilum undulatum* can be misidentified with *C. orgyale*, but has somewhat smaller flowers and a different lip callus, composed of the median keel terminated with extended nose-like shape, branching basally into digitate lobules, with a series of additional, irregular thickenings on both sides of central callus. In *C. orygale* the large callus consists of 3 parallel keels, of which the median one is terminated with extended nose-like shape, branching basally into digitate lobules, with a series of additional, irregular thickenings on both sides of central callus.

*Representative specimens*: COLOMBIA. **Cauca**: Pomplayon-Popayán, *F.C. Lehmann 6243* (W-R! 7904, UGDA-DLSz!—drawing). [New Grenada], *A. Humboldt s.n*. (P, W-R! 36987; UGDA-DLSz!—drawing).

***Cyrtochilum violaceum*** Dalström, Lankesteriana 12: 143. 2012. TYPE: Colombia. Magdalena. *Jaramillo M. et al. 5366* (holotype, COL).

**Figure 75:**
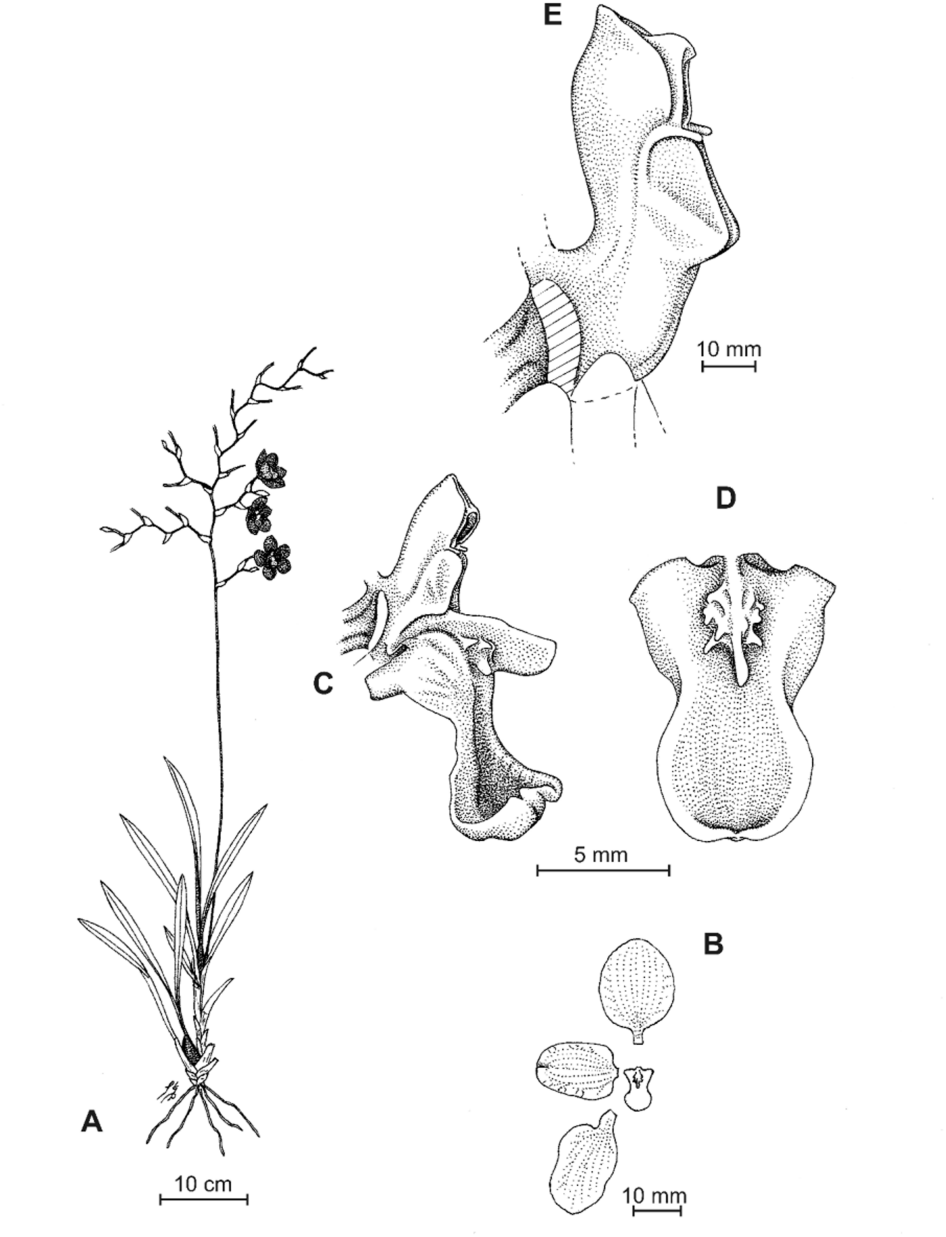
*Cyrtochilum violaceum* Dalström. (A) Habit; (B) Dissected perianth; (C) Gynostemium and lip, side view; (D) Lip, front view; (E) Gynostemium. Drawn by Stig Dalström from the holotype, *Jaramillo M. et al. 5366* (COL).

Pseudobulbs caespitose to creeping on a bracteate rhizome, about 5 cm long, 2 cm wide, ovoid, bifoliate, surrounded basally by 7–8 distichous sheaths, the uppermost foliaceous. Leaves about 16–17 cm long, 2 cm wide, subpetiolate, conduplicate, elliptic to slightly obovate, narrowly acute. Inflorescence up to about 70 cm long, axillary from the uppermost sheath, an erect to arching, loosely flexuous panicle with widely spaced 3–4 flexuous, 2- to 4-flowered side-branches (up to 6 or more flowers on 7 branches have been noted on an additional specimen). Floral bracts about 10–15 mm long, involute and cucullate. Pedicel with ovary 20–25 mm long. Flowers apparently open and stellate, tepals violet, lip callus yellow. Dorsal sepal about 25 mm long, 18 mm wide, shortly spathulate, then truncate and broadly ovate to elliptic laminate, obtuse, slightly undulate. Lateral sepals about 25 mm long, 15 mm wide, slightly obliquely spathulate, then obliquely cordate, broadly and weakly pandurate laminate, obtuse. Petals about 20 mm long, 13 mm wide, almost sessile, truncate to cordate, then broadly ovate and rounded obtuse with a canaliculate acute, almost folded apex. Lip about 10 mm long, 8 mm wide, truncate to cordate, pandurate-ovate with obtuse triangular lateral lobes, and a rounded and slightly concave, weakly bilobed to minutely apiculate apical lobe; callus in form of a fleshy denticulate structure emerging from the base and extending to almost half the length of the lip lamina, with several spreading lower lateral denticles and a dominating, projecting, laterally compressed, nose-like central keel. Gynostemium about 7 mm long, robust, gently bent back, with obscure, digitate appendages and prominent wings below them (Fig. 75).

*Ecology*: Epiphytic in forest.

*Distribution*: Colombia. Alt. 2,700–2,900 m.

*Notes*: *Cyrtochilum violaceum* resembles *C. undulatum* from which it differs in the form of the lip and gynostemium. Cyrtochilum *violaceum* has a pandurate lip lamina, the cleft and distinct frontal angles of the stout column, and the pair of digitate or narrowly clavate wings on each side below the stigmatic surface while *C. undulatum* has a triangular lip lamina, and a more slender and sigmoid column with short angular knobs only, or without wings altogether.

*Representative specimens*: COLOMBIA. **Magdalena**: Sierra Nevada de Sta. Marta, Transecto del Alto Rio Buritaca, Cuchilla, Lev. 29. Proyecto Desarrollo, 2900 m, 5 Aug. 1977, *R. Jaramillo M. et al. 5366* (COL), the same loc., Lev. 27. Proyecto Desarrollo, 2,700 m, 2 August 1977, *R. Jaramillo M. et al. 5352* (COL).

***Cyrtochilum pastasae*** (Rchb.f.) Kraenzl., Notizbl. Bot. Gart. Berlin-Dahlem 7: 93. 1917. ≡ *Oncidium pastasae* Rchb.f., Linnaea 41: 21. 1876. TYPE: Ecuador. *Spruce 5078* (holotype, W-R! 5058, UGDA-DLSz!—drawing).

**Figure 76:**
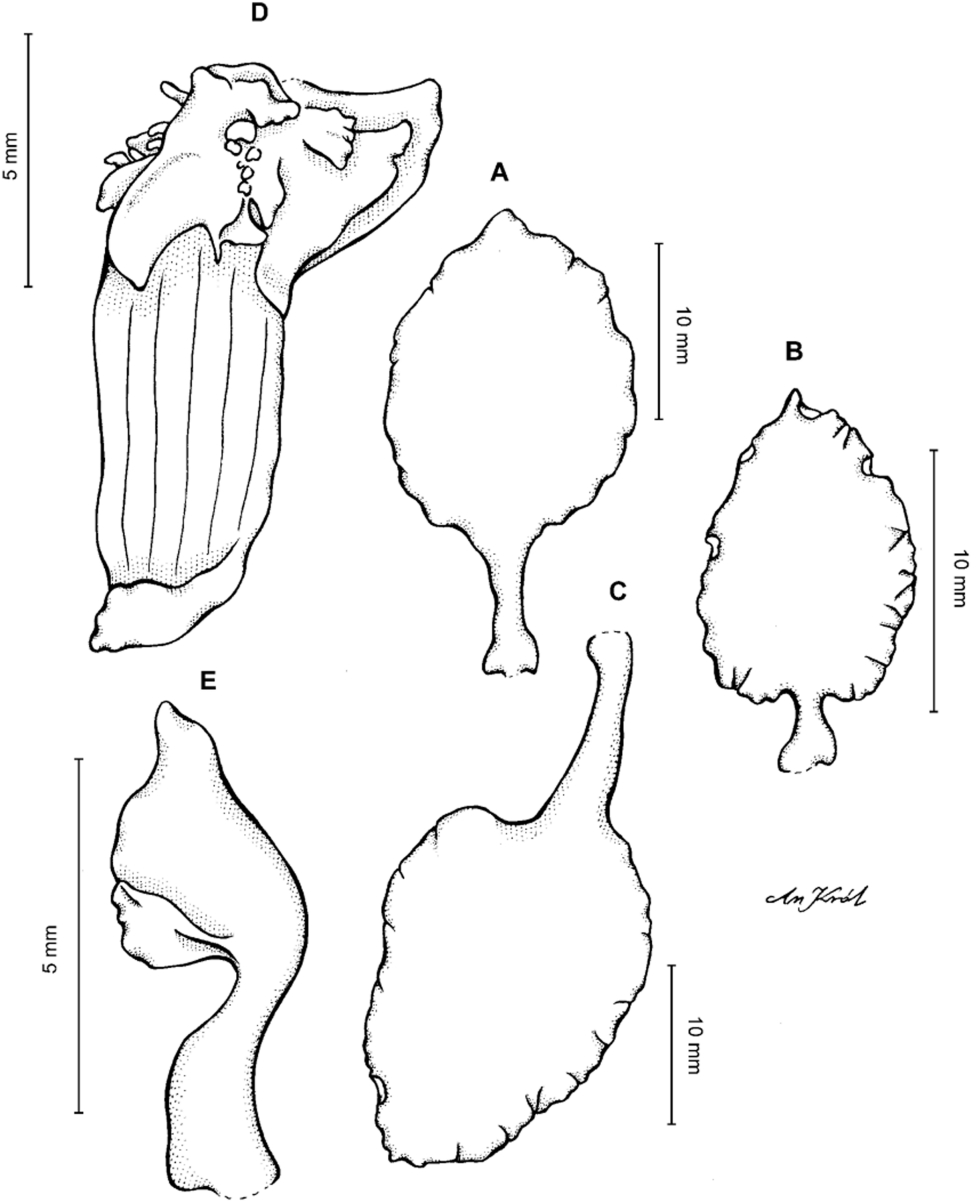
*Cyrtochilum pastasae* (Rchb.f.) Kraenzl. (A) Dorsal sepal; (B) Petal; (C) Lateral sepal; (D) Lip; (E) Gynostemium, side view. Drawn by Anna Król from *Spruce 5078* (W-R).

Pseudobulbs widely distant on a creeping, bracteate rhizome, surrounded basally and hidden by several distichous, folicaeous sheaths. Leaves subpetiolate, conduplicate, elongate-linear to narrowly obovate, acuminate. Inflorescence up to several meters long, axillary, an erect then wiry panicle with widely spaced, flexuous, few- to several-flowered branches. Floral bracts 8–10 mm long, appressed, scale-like. Pedicel with ovary 20–30 mm long. Flowers stellate, sepals reddish brown, petals basally brown then purple and apically white, lip basally brownish then white and apically lilac-purple, callus white to yellowish. Dorsal sepal clawed; claw up to 8 mm long, obscurely winged; blade 17–25 mm long, 15–21 mm wide, ovate to ovate-elliptic, obtuse, margin slightly undulate. Petals clawed; claw about 3–4 mm long, with two small appendages at the base; blade 10–21 mm long, 6–15 mm mm wide, ovate, obtuse, margin undulate. Lateral sepals clawed; claw up to 10 mm long, with a small appendage at the base; blade up to 20 mm long, 11–18 mm, ovate, minutely apiculate, margin slightly undulate. Lip 11.5–13 mm long, 7 mm wide, oblong ovate-triangular, obscurely 3-lobed; basal part deltoid, truncate, lateral lobes ovate, obtuse; callus consisting of a basal, central, longitudinal, projecting, laterally compressed, extended into nose-like keel, flanked by several layers of spreading, irregularly dentate to tuberculate ridges, flanked basally by fleshy, curved ridges and tubercles; apical part ovate-ligulate, attenuate towards obtuse apex. Gynostemium 7 mm long, arcuate, lateral appendages obovate, apically truncate and crenate (Fig. 76).

*Ecology*: Terrestrial or epiphytic on steep slopes in very wet montane forest or cloud forest.

*Distribution*: Colombia, Ecuador, Peru (Dalström, 2010). Alt. 1,600–3,200 m.

*Notes*: *Cyrtochilum pastazae* can be misidentified as *C. undulatum*, but the basal part of the lip in the latter species is subrectangular-ovate, broadly cuneate and widest towards the apex and gynostemium wings of this species are rudimentary.

*Representative specimens*: COLOMBIA. **Putumayo**: San Francisco, Vereda La Esperanza, entre Minchoy y Mocoa, ca. 1,700 m, 4 February 2005, cultivated in San Francisco by *R. Medina 740* (HPUJ—flower in alcohol; Ortiz Valdivieso, 2009). ECUADOR. **Morona-Santiago**: Chiguinda, Novomber 1980, *A. Andreetta s.n*. (SEL—Dalström, 2010); Gualaceo-Limón, Plan de Milagro, 1,700 m, 9 July 1982, *C. Dodson & A. Embree 13184* (SEL, US—Dalström, 2010). **Napo**: Above bridge of Rio Cosanga, 00°36′S 77°52′W, 2100 m, October 1990, *W. Palacios 6378* (QCNE—Dalström, 2010); Guacamayos Pass, 1,900 m, April 1983, *A. Hirtz 903* (SEL—Dalström, 2010). **Tungurahua**: Along Río Pastaza near Mount Tungurahua, *R. Spruce 5078* (W-R!), Km 14 between Baños and Puyo, 1,600 m, 8 October 1961, *C. Dodson & L. Thien 899* (SEL—Dodson & Dodson, 1982); east-facing slope of Mt. Tungurahua, 3,200 m, 23 October 1961, *C. Dodson & L. Thien 1086* (SEL, QCA—Dodson & Dodson, 1982); near Baños, 2,000 m. Flowered in cultivation, 29 April 1976, *J. & L. Kuhn 0–9* (SEL—Dodson & Dodson, 1982); the same loc., *A. Andreetta 0711* (SEL—Dodson & Dodson, 1982). **Zamora-Chinchipe**: Valladoid, on island river, 1,700 m, 1 April 1983, *S. Dalström 423* (RPSC—Dalström, 2010), 15 km above Zamora on road to Loja, 4°S 70°01′W, 9 Novomber 1986, *D. Neill & C. Cerón 7485* (MO—Dalström, 2010).

***Cyrtochilum geniculatum*** Königer, Orchidee (Hamburg) 42: 135. 1991. TYPE: Ecuador. *Königer & Königer K-164b* (holotype, M; isotypes: K, M, QCA).

**Figure 77:**
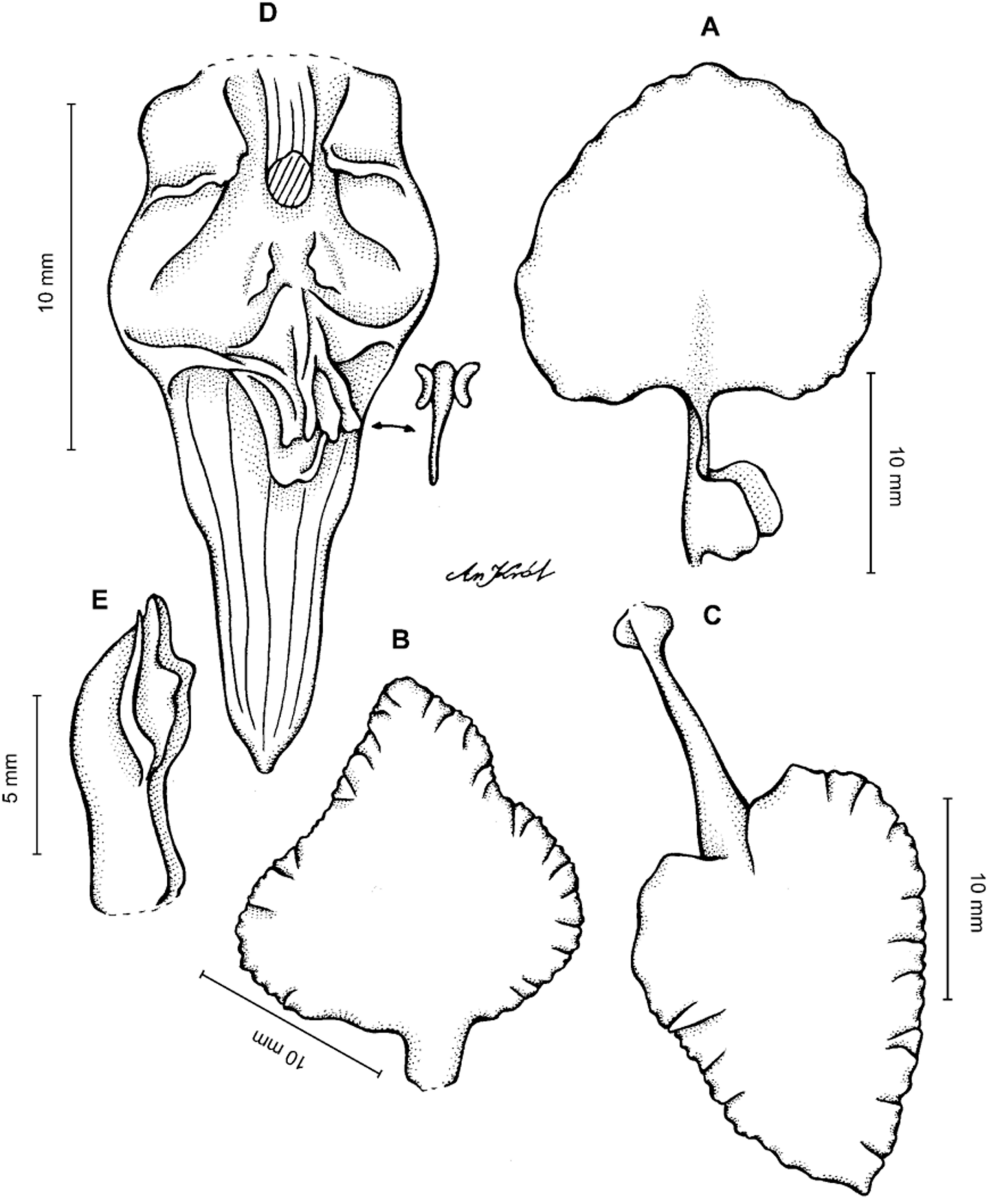
*Cyrtochilum geniculatum* Königer. (A) Dorsal sepal; (B) Petal; (C) Lateral sepal; (D) Lip; (E) Gynostemium. Drawn by Anna Król from *Jameson s.n*. (W-R).

Plants long-rhizomatous. Pseudobulbs 5–7 cm long, 1–2 cm in diameter, narrowly ovoid, laterally compressed, subtended basally by foliaceous sheaths. Leaves 20–25 cm long, 2–4 cm wide, oblanceolate, acuminate. Inflorescence up to 3 m long, serpentine, branching, branches several-flowered. Flowers medium-sized, conspicuous, tepals pale olive to red-brown or purple with white margins and apex, lip basally white, then brown or purplish. Floral bracts 15–23 mm long. Pedicel and ovary about 40 mm long. Dorsal sepal clawed; claw 8 mm long, narrow, canaliculate, with prominent rhombic basal wings; blade 17–19 mm long, 18–19 mm wide, reniform, base truncate, margins weakly undulate, entire, apex obtuse. Petals clawed; claw 2–4 mm long, wide; blade 17–18 mm long and wide when spread, triangular-ovate, somewhat oblique, obtuse at the apex, base truncate, margins undulate. Lateral sepals clawed; claw 11–15 mm long, narrow, basally winged; blade 17–19 mm long, 15 mm wide, obliquely elliptic-ovate in general outline, subobtuse at the apex, subcordate at the base, margins entire, undulate. Lip 20–21 mm long in total, 8–9 mm wide, slightly curved in natural position, undivided, ligulate-lanceolate in general outline, base truncate, apex attenuate; callus consisting of three ridges, terminating in 3, subequal lobes, additional thickenings along the bending line of basal wings, with two incurved folds along fusion line between gynostemium and lip. Gynostemium 10–11 mm long, subarcuate, connate basally with the lip, lateral appendages prominent, digitate, upwards, reaching the anther base (Fig. 77).

*Ecology*: Epiphyte.

*Distribution*: Ecuador. Alt. 1,800–2,335 m.

*Notes*: *Cyrtochilum geniculatum* is very a characteristic species with a complicated lip callus. It consists of three ridges, terminating in 3, subequal lobes. The main callus is covered basally with two, incurved folds along fusion line between gynostemium and lip. There are some additional thickenings along the bending line of basal wings. The gynostemium is also peculiar in having very long, digitate and upcurved projections. It can be occasionally confused with such species as *C. undulatum*, *C. pastazae* or *C. halteratum*. It can be easily recognised from both former species by the form of dorsal sepal which blade is reniform, nearly as long as wide with truncate base (vs suborbicular-ovate to ovate, longer than wide, with cuneate base), and petals which are more or less triangular, as long as wide (vs ovate, longer than wide). Cyrtochilum *halteratum* appears to be morphologically more similar to *C. geniculatum*, but the former has simplier lip callus.

*Representative specimens*: ECUADOR. **Bolívar**: Ventanas-Echeandea-Guaranda, km 60, 2,335 m, *C. Dodson et al. 18756* (MO—Dalström, 2010). **Carchi**: 12 km E of Maldonado on road to Tulcan, 2,230 m, 27 September 1979, *A. Gentry & G. Shupp 26643* (MO!). **Cotopaxi**: 4 km E of Macuchi, 1,800 m, 26 July 1979, *C. Dodson, P. Morgan & M. Fallen 8562* (MO!, UGDA-DLSz!—drawing). **Pichincha**: Bei Mindo, 2,200 m, 10 April 1988, *W. Königer & H. Königer K-164b* (M, K, QCA, Herb. Königer). *Sine loc., W. Jameson s.n*. (W-R! 14302, UGDA-DLSz!—drawing).

***Cyrtochilum halteratum*** (Lindl.) Kraenzl., Notizbl. Bot. Gart. Berlin-Dahlem 7: 93. 1917. ≡ *Oncidium halteratum* Lindl., Orchid. Linden.: 14. 1846. TYPE: Colombia [New Grenada]. *Linden 1289* (holotype, W-R! 48833; UGDA-DLSz!—drawing).

**Figure 78:**
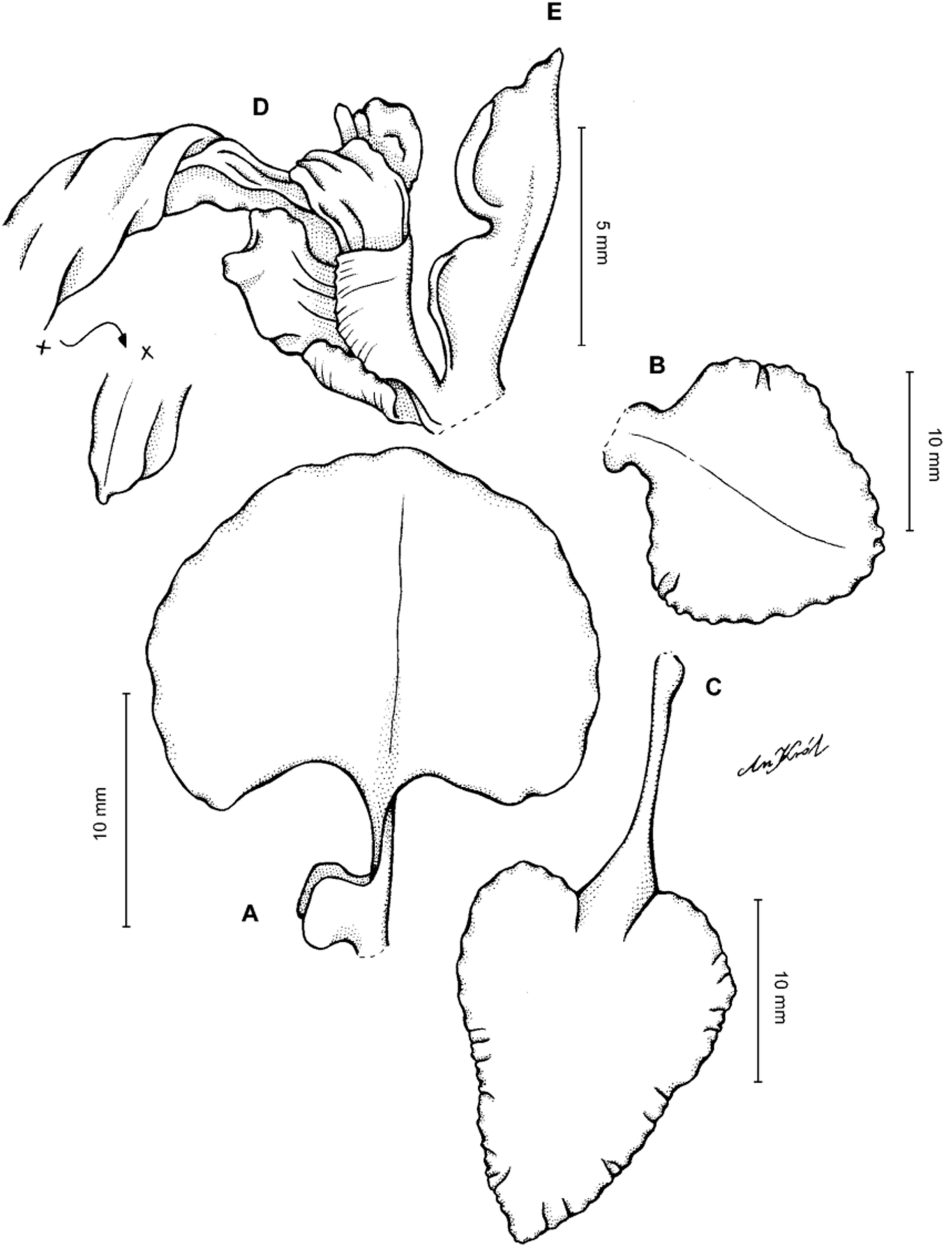
*Cyrtochilum halteratum* (Lindl.) Kraenzl. (A) Dorsal sepal; (B) Petal; (C) Lateral sepal; (D) Lip; (E) Gynostemium. Drawn by Anna Król from *Linden 1289* (W-R).

Pseudobulbs caespitose, 5–10 cm long, 2.5–3.5 cm in diameter, ovoid, laterally somewhat compressed, uni- or bifoliate. Leaves 20–30 cm long, 1.5–4 cm wide, oblanceolate, acuminate. Inflorescence up to 3 m long, paniculate, with widely spaced few-flowered branches. Flowers large, conspicuous, showy, sepals yellowish-white, basally red-brown, lip reddish-purple to brown. Floral bracts 15–18 mm long. Pedicel and ovary about 40–55 mm long. Dorsal sepal clawed; claw 6–8 mm long, rather narrow, canaliculate, with very prominent subrectangular wings just above the base; blade 16–23 mm long, 22 mm wide, broadly ovate to orbicular-ovate, base truncate, margins crenate, undulate, apex acute, recurved. Petals clawed; claw about 3–5 mm long, wide; blade 15–22 mm long in total, 16–17 mm wide when spread, obliquely cordate-suborbicular, base truncate, acute and recurved at the apex, margins irregularly undulate. Lateral sepals prominently clawed; claw 9–10 mm long, narrow, with basal, prominent wings; blade 20–27 mm long, 17–18 mm wide, obliquely oblong ovate in general outline, basally cuneate, subacute at the apex, margins undulate. Lip 15–22 mm long in total, curved in natural position exposing massive callus; basal part 7–8 mm wide, elliptic, base cuneate, convex along the centre; callus consisting of narrow central ridge much extended, more or less nose-like, flanked by smaller outgrowths on each side, glabrous; apical part about 4 mm wide, ligulate, attenuate towards blunt apex. Gynostemium 9–13 mm long, subarcuate, connate basally with the lip, lateral appendages oblique, digitate, reaching the anther base (Fig. 78).

*Ecology*: No data.

*Distribution*: Colombia, Ecuador (Dodson & Luer, 2010), Venezuela. Alt. 2,700 m.

*Notes*: This species has a rather simple lip callus of 3 ridges, with the median one being a little higher than both laterals. Petals are shortly clawed, claws of sepals are adorned with a pair of prominent basal wings.

*Representative specimens*: COLOMBIA. **Santander**: Bucamaranga. El Picacho, 2,700 m, 30 March 2002, *M. Ospina H. 1557* (COL!, UGDA-DLSz!—drawing). [New Grenada], *J. Linden 1289* (W-R! 48833; UGDA-DLSz!—drawing). VENEZUELA. San Pedro, 2,000 m, *H. Wagener s.n*. (W-R! 48834, UGDA-DLSz!—drawing).

***Cyrtochilum maasii*** Szlach. & Kolan., Nordic J. Bot. 32(6): 728. 2014. TYPE: Colombia. *Maas & Plowman 2111* (holotype, COL! 181940, UGDA-DLSz!—drawing).

**Figure 79:**
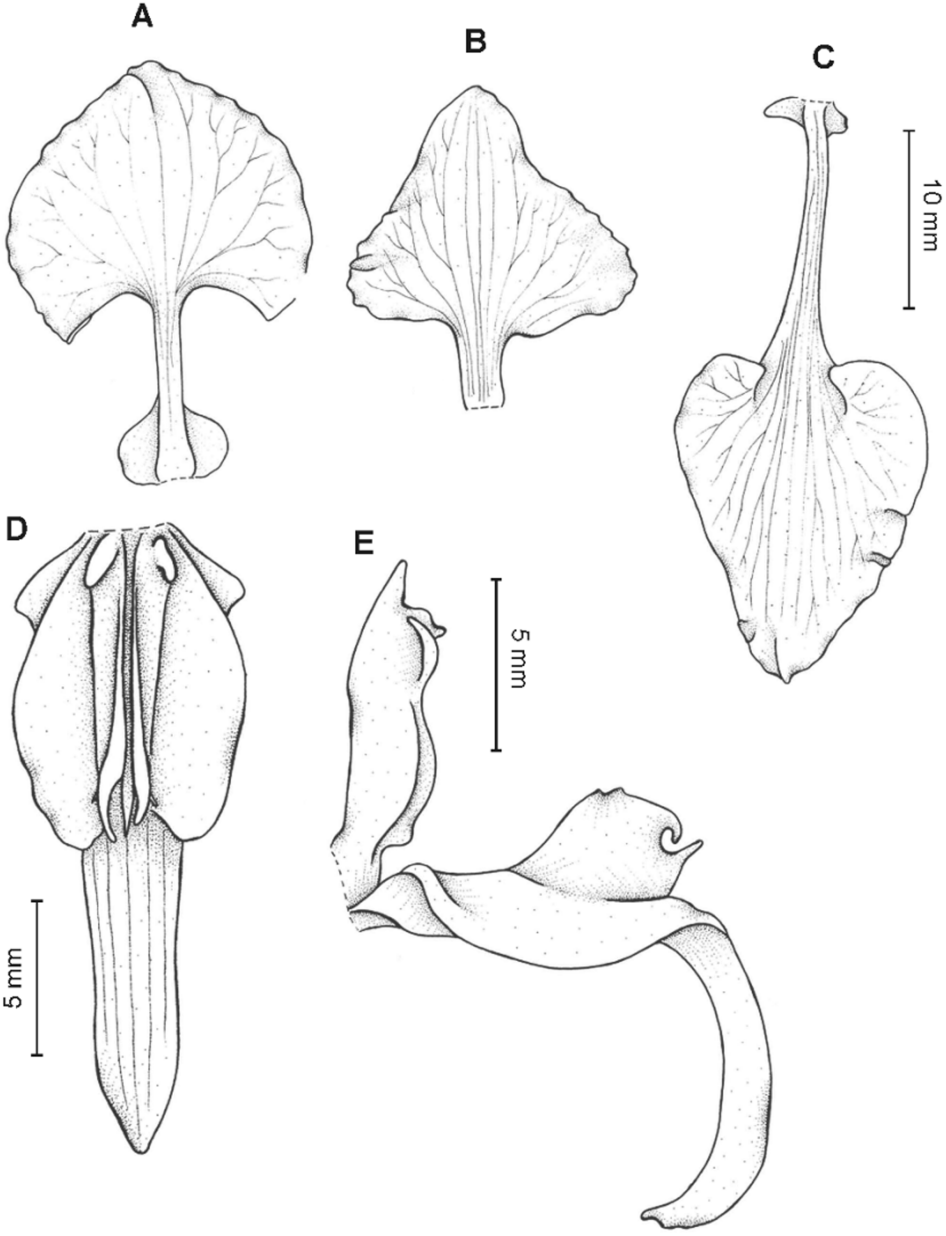
*Cyrtochilum maasii* Szlach. & Kolan. (A) Dorsal sepal; (B) Petal; (C) Lateral sepal; (D) Lip; (E) Gynostemium and lip, side view. Drawn by Natalia Olędrzyńska from *Maas & Plowman 2111* (COL).

Long-rhizomatous plants. Pseudobulbs about 8 cm long and 1 cm in diameter, oblong-ovoid, somewhat compressed, elliptic in cross-section, basally concealed by leafy sheaths. Leaves up to 50 cm long and 3.5 cm wide, linear to linear-oblanceolate, acute. Inflorescence serpentine, branching. Flowers large, conspicuous. Floral bracts 15 mm long. Pedicel about 25 mm long, ovary about 10 mm long. Dorsal sepal clawed; claw 10 mm long, narrow, canaliculate, with prominent, rhombic wings at the base; blade 19–21 mm long, 22–23 mm wide, cordate-reniform, base cordate-sagittate, margins weakly undulate, apex obtuse, somewhat recurved. Petals clawed; claw about 8 mm long, wingless; blade 16–18 mm long, 18–20 mm wide when spread, obliquely triangular, obtuse at the apex, truncate at the base, margins erose, slightly undulate. Lateral sepals clawed; claw about 15 mm long, narrow, with wings unequal in size at the base; blade 20 mm long, 16–17 mm wide, ovate-subcordate in general outline, subobtuse at the apex, margins almost entire, weakly undulate towards the apex. Lip 20–21 mm long in total, slightly curved in natural position; basal part 10 mm long, 8–9 mm wide, elliptic in outline, supported basally by oblique wings, somewhat convex with canaliculate basal part, callus a narrow ridge rising in the centre terminating with 3 lobes, each lobe acute, prominently hooked; apical part 10–11 mm long, 4 mm wide, ligulate, triangular towards subobtuse apex, canaliculate. Gynostemium 10 mm long, almost straight, connate basally with the lip, with basal, chin-like protuberance, lateral appendages obliquely lanceolate, strongly falcate, reaching the anther base (Fig. 79).

*Ecology*: Terrestrial in roadsides and páramo.

*Distribution*: Colombia. Alt. 2,100–3,000 m.

*Notes*: This species resembles both *C. halteratum* and *C. kraenzlinianum* described below. From the former *C. maasii* differs by having a sagittate base of the dorsal sepal (vs base truncate to cuneate), free lateral sepals with blade nearly as long as the claw (vs claw half as long as the blade), petals wider than long (vs longer than wide), a lip with equally long basal and apical parts (vs basal part longer than apical one), and a 3-hooked apex of the lip callus (vs lip callus truncate apically). It differs from *C. kraenzlinianum* by the form of the dorsal sepal (claw 6–7 mm long, wings rhombic in *C. kraenzlinianum*) and lip width/length ratio (0.45 in *C. kraenzlinianum*).

*Representative specimens*: COLOMBIA. **Boyacá**: Mpio. Arcabuco. La Cumbre, 2,300 m, 8 October 1980, *G. Lozano & J. Diaz 3717* (COL!). **Cauca**: Km 54 of road from Timbio to Veinte de Julio, roadside, 2,100–2,300 m, 14 October 1974, *P.J. Maas & T.C. Plowman 2111* (COL!). **Cundinamarca**: Macizo de Bogotá. Páramo de Chipaque, September 1941, *Schultes s.n*. (COL!); Páramo de Usaquén, 3,000 m, 1 Novomber 1953, *M. Schneider 123* (COL!); *Sine loc. F. Pombo s.n*. (COL!). **Santander/Boyacá**: Limites entre los Departamentos. Corregimiento de Virolin. Finca La Sierra, 2,500–2,600 m, 12 May 1976, *G. Lozano, J. Torres & S. Diaz 2367* (COL!).

***Cyrtochilum kraenzlinianum*** Szlach. & Kolan., Nordic J. Bot. 32(6): 726. 2014. TYPE: Colombia. *Zarucchi et al. 6210* (holotype, COL! 377036; isotype: MO!, UGDA-DLSz!—drawing).

**Figure 80:**
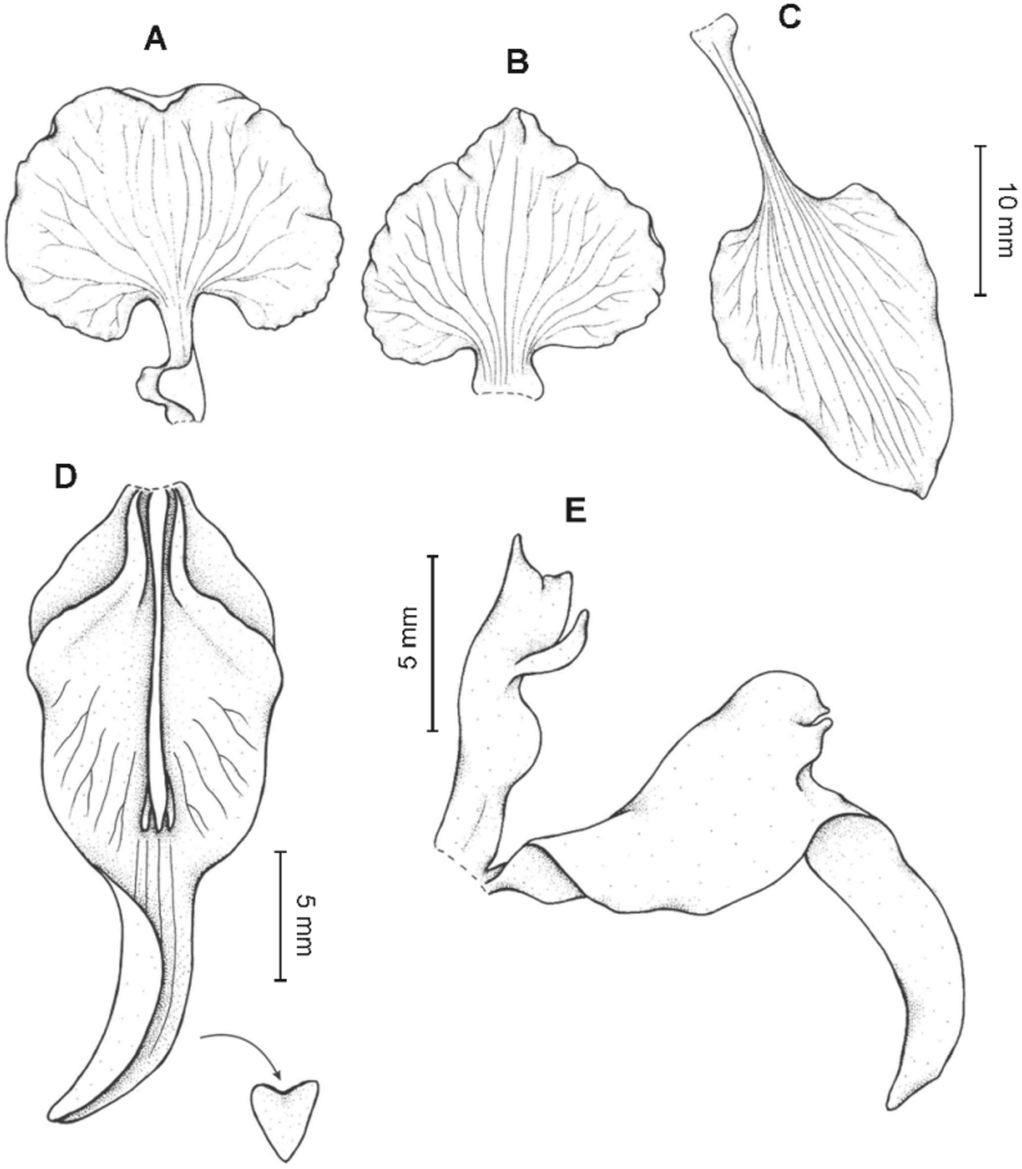
*Cyrtochilum kraenzlinianum* Szlach. & Kolan. (A) Dorsal sepal; (B) Petal; (C) Lateral sepal; (D) Lip; (E) Gynostemium and lip, side view. Drawn by Natalia Olędrzyńska from *Zarucchi et al. 6210* (COL).

Pseudobulbs and leaves absent in the herbarium material. Inflorescence serpentine, branching. Flowers large, conspicuous. Floral bracts about 20 mm long. Pedicel 30 mm long, ovary about 7–10 mm long. Dorsal sepal clawed; claw 6–7 mm long, rather narrow, canaliculate, with prominent elliptic wings just above the base; blade 18–20 mm long, 20–23 mm wide, reniform, base cordate, margins somewhat undulate and crispate, apex obtuse, somewhat recurved. Petals shortly clawed; claw 4 mm long, wingless; blade 15–16 mm long, about 20 mm wide when spread, transversely elliptic-cordate, subobtuse at the apex, somewhat oblique, margins slightly undulate. Lateral sepals clawed; claw 12–13 mm long, narrow, with basal wing on the outer margin; blade 21–23 mm long, 15–16 mm wide, obliquely elliptic-ovate in general outline, truncate basally, subacute at the apex, margins almost entire. Lip about 25 mm long in total, slightly curved in natural position; basal part 14–15 mm long, 10–11 mm wide, almost rectangular, somewhat convex with canaliculate basal part; callus a narrow ridge rising in the centre terminating with 3 lobes, each lobe being acute, somewhat curved; apical part 10–11 mm long, 4 mm wide, ligulate, thickened towards acute and recurved apex, and here triangular in cross section. Gynostemium 10 mm long, sigmoid, connate basally with the lip, lateral appendages obliquely lanceolate, falcate, not reaching the anther base (Fig. 80).

*Ecology*: Terrestrial in wet montane vegetation, as well as in the disturbed roadsides.

*Distribution*: Colombia. Alt. about 2,700 m.

*Notes*: This species resembles *C. halteratum*, from which it differs by the dorsal sepal and petals being wider than long (vs longer than wide), lateral sepals being free to the base (vs lateral sepals basally connate), lip apical part triangular in cross section (vs canaliculate) and lip callus lobes acute, somewhat curved (vs callus lobes truncate). Cyrtochilum *kraenzlinianum* differs from *C. maasii* by having elliptic-ovate lateral sepals (vs subcordate-ovate) and basal lip part longer than apical one (vs basal lip part as long as apical one).

*Representative specimens*: COLOMBIA. **Antioquia**: Mpio. Sonsón. Km 11 of road Sonsón-Nariño (25 km from Nariño), 5°42′N 75°15′W, 2,700 m, 3 October 1987, *J. Zarucchi, A. Brant & J. Roldán 6210* (COL!). **Santander**: Bucaramanga. El Picacho, 2,700 m, 30 March 2002, *M. Ospina H. 1556* (COL!).

***Cyrtochilum portillae*** Königer, Arcula 3: 61. 1995. TYPE: Ecuador. *Königer WJ-38* (holotype, M; isotypes: K, QCA, herb. Königer).

**Figure 81:**
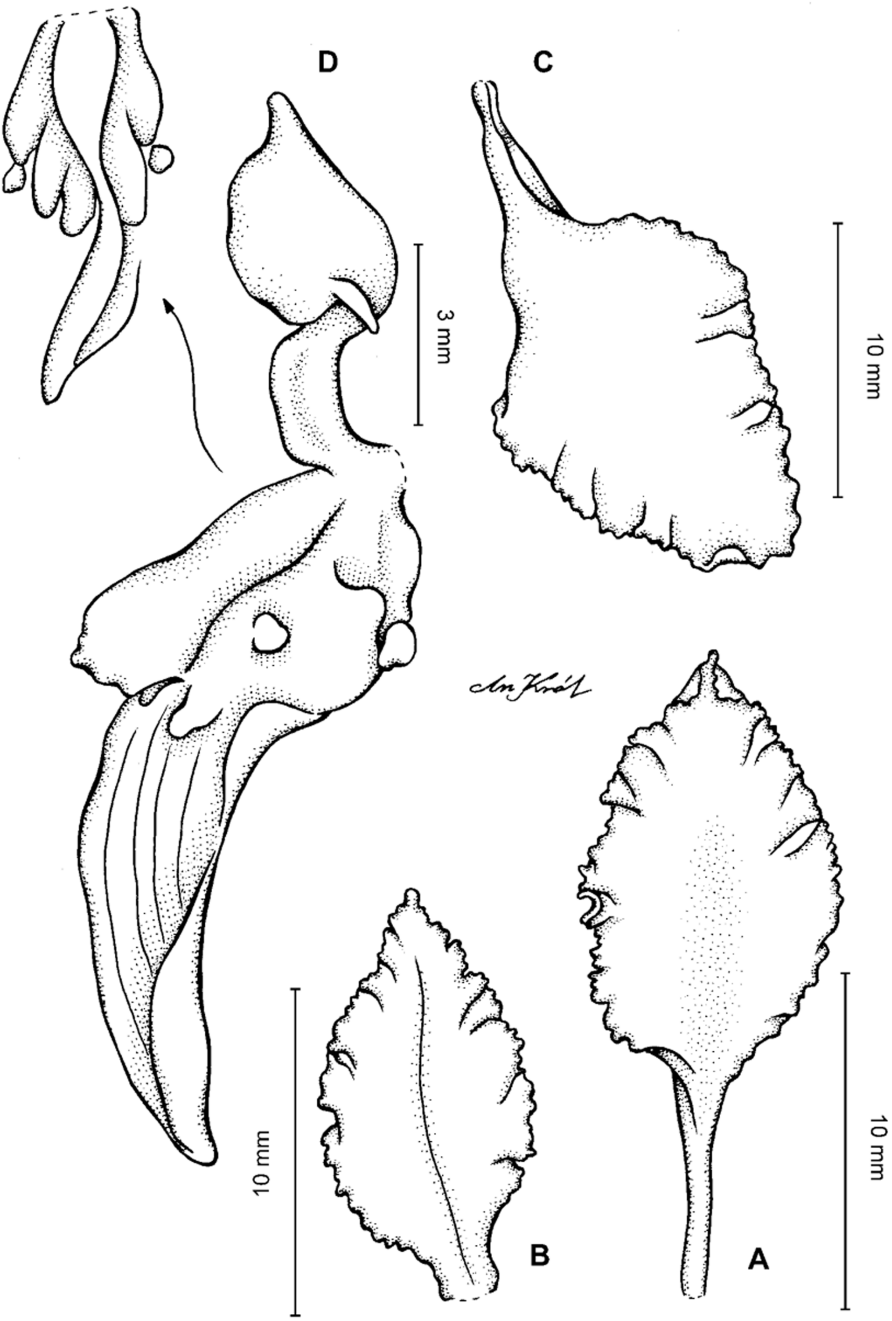
*Cyrtochilum portillae* Königer. (A) Dorsal sepal; (B) Petal; (C) Lateral sepal; (D) Lip and gynostemium, side view. Drawn by Anna Król from *Lehmann BT 128* (AMES).

Rhizome short. Pseudobulbs ovoid, subcompressed, up to 10 cm long, 4 cm wide, 2–leaved apically, with 1–2 leaf-bearing sheaths on both sides. Leaves up to 40 cm long, 4 cm wide, narrowly lanceolate, acuminate above, conduplicate towards the base. Inflorescence 1.5–2.5 m long, twining, slender, with 6–8 distant, short, loosely 2–3-flowering branches, peduncle about 30 cm long. Floral bracts tubular, 9–10 mm long. Pedicel and ovary 20–40 mm long. Flowers fragrant, dorsal sepal light-brown, very narrowly margined with yellow, auricles white, suffused with rose, lateral sepals light-brown, greenish-yellow towards the tips, the auricles white, suffused with rose, petals brown with yellow tips, lip white, suffused with light-purple towards the margins and in the apical half, callus white. Dorsal sepal clawed; claw 6–8 mm long, canaliculate within, at the base on both sides auriculate, the auricles large, rounded, incurved; blade 13–14 mm long, 7.5–11 mm wide, ovate to elliptic, subconcave, acute, subundulate marginally. Petals shortly, broadly clawed; claw about 3 mm long and 4 mm wide, geniculate, margined; blade 11–17 mm long, 6–13 mm wide, oblong ovate, flat, subundulate, acute at the apex, becoming broadly cuneate towards the claw basally. Lateral sepals long-clawed; claw about 10 mm long, canaliculate within, at the base on both sides auriculate, the auricles large, rounded; blade 12–18 mm long, 7–10 mm wide obliquely and narrowly elliptical, subconcave, subacute to obtuse apically, becoming cuneate towards the claw, margin undulate, the midvein thickened dorsally. Lip 14–20 mm long, basally 13 mm wide expanded, oblong-cuneate in outline, with distinct, broadly triangular lateral lobes at the base, subconvex in the posterior half, turned back in the anterior half, concave, the margins erose, rounded apically; the midline lamella of the callus cuneate, tall, extended towards the base into a low torus and joined with the column through a ligule, apically on both sides with an acute keel, at the base on both sides with a stout keel becoming lower towards the base. Gynostemium 6–8 mm high, subsigmoid, wings falcate-subulate, acute (Fig. 81).

*Ecology*: Epiphyte.

*Distribution*: Ecuador. Alt. 1,800–2,000 m.

*Notes*: This species is characterized by elevated a lip callus which consists of massive lamella which is triangular in cross section, flanked by few outgrowths on both sides. The gynostemium appendages are linear, upcurved. Cyrtochilum *portillae* differs from the similar *C. plagianthum* in form of gynostemium appendages which are obliquely oblanceolate, with oblique, truncate apex in the latter and falcate-subulate, acute in *C. portillae*. Plants collected by *Lehmann BT128* have prominently undulate-serrate sepals and petals.

Dalström (2001) considered this species as conspecific with *C. mendax* in which the lip lamellae are of similar size (vs median one much larger in *C. portillae*).

*Representative specimens*: ECUADOR. **El Oro**: Paccha-Pasaje, 1,800 m, 23 May 1988, *A. Hirtz 3869* (SEL, flower in alcohol—Dalström, 2010). **Loja**: Celica, 2,000 m, *W. Königer WJ-38* (K, M, QCA, herb. Königer); *F.C. Lehmann BT128* (AMES!, UGDA-DLSz!—fragment, photo, drawing).

***Cyrtochilum plagianthum*** (Rchb.f.) Kraenzl., Notizbl. Bot. Gart. Berlin-Dahlem 7: 93. 1917. ≡ *Oncidium plagianthum* Rchb.f., Gard. Chron. 1873: 915. 1873. TYPE: Colombia. *Carder 134*. (holotype, W-R! 36985).

**Figure 82:**
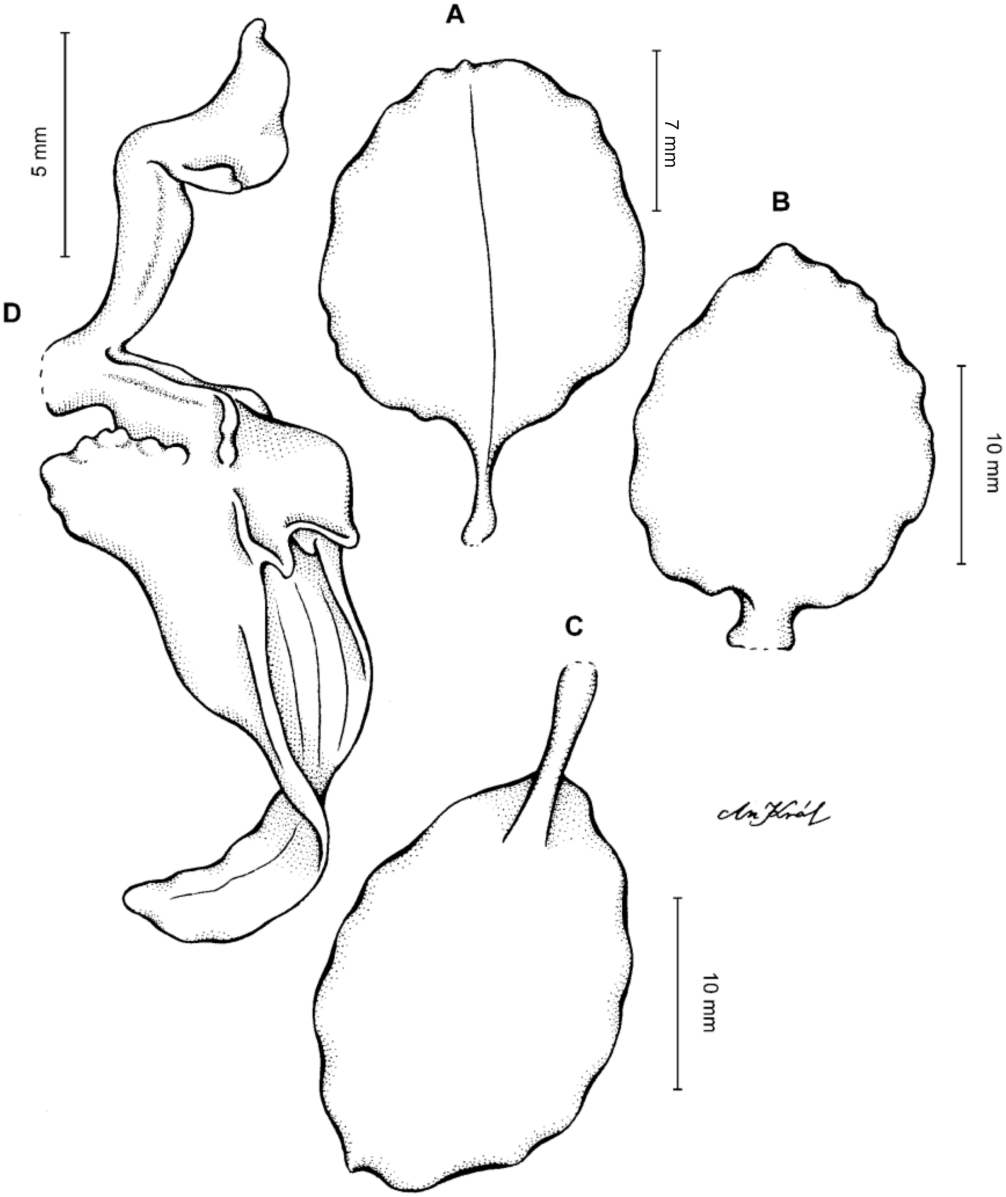
*Cyrtochilum plagianthum* (Rchb.f.) Kraenzl. (A) Dorsal sepal; (B) Petal; (C) Lateral sepal; (D) Lip and gynostemium, side view. Drawn by Anna Król from *Baldewian s.n*. (W-R).

Flowers medium-sized, conspicuous. Floral bracts 10 mm long. Pedicel and ovary 23 mm long. Dorsal sepal clawed; claw 5 mm long, narrow, canaliculate, wingless; blade 18 mm long, 13–14 mm wide, elliptic-suborbicular, margins entire, base cuneate, apex rounded. Petals clawed; claw about 2 mm long, wide; blade 15 mm long, 12 mm wide when spread, oblique, suborbicular-ovate, base truncate, apex obtuse, margins entire. Lateral sepals prominently clawed; claw 4 mm long, narrow, wingless; blade 19 mm long, 15 mm wide, obliquely broadly elliptic in general outline, basally cuneate, apex obtuse, margins entire. Lip 12 mm long in total, 10–11 mm wide at the base, curved in natural position just above the base, triangular-ovate in outline, base truncate, apex ligulate, attenuate; callus prominent consisting of median nose-like keel, flanked apically by small lateral outgrowth, and basally by two short keels, and additional thickenings beyond main callus. Gynostemium 8 mm long, sigmoid, connate basally with the lip, lateral appendages obliquely oblanceolate, obliquely truncate at apex, not reaching the anther base (Fig. 82).

*Ecology*: No data.

*Distribution*: Colombia.

*Notes*: This species can be confused with *C. portillae*, and both might be conspecific. In our opinion they can be separated based on the gynostemium appendages, which are falcate-subulate, acute in *C. portillae*, and obliquely oblanceolate, with oblique, truncate apex in *C. plagianthum*.

Dalström (2001) considered this species as conspecific with *C. xanthodon* in which the dorsal sepal is distinctly clawed, all tepals are undulate, and the lip callus constists of a prominent plate with truncate apex and additional growths flanking main callus.

*Representative specimens:* COLOMBIA. *Baldewian s.n*. (W-R! 36988, UGDA-DLSz!—drawing).

**(8) *Superbiens*-group**

Lip narrow, callus a single ridge.

Five species of this group are known so far exclusively from Colombian Andes.

### Key to the species

1 Gynostemium lateral appendages obliquely oblanceolate, upcurved, apical margin more or less dentate*C. superbiens*
1* Gynostemium lateral appendages obliquely lanceolate, falcate, non-dentate2
2 Lip without longitudinal ridge running obliquely along basal part*C. santanderensis*
2* Lip with more or less prominent oblique ridge running along basal part3
3 Dorsal sepal with sagittate base*C. rangelii*
3* Dorsal sepal with cordate base4
4 Claw of lateral sepals subequal in length to the blade, basal lip part subrectangular-elliptic, apical part oblong elliptic*C. ortizianum*
4* Claw of lateral sepals half as long as blade, basal lip part semiorbicular, apical part oblong triangular*C. boyacensis*

***Cyrtochilum superbiens*** (Rchb.f.) Kraenzl., Notizbl. Bot. Gart. Berlin-Dahlem 7: 93. 1917. ≡ *Oncidium superbiens* Rchb.f., Linnaea 22: 843. 1850. TYPE: Colombia [New Grenada]. *Funck & Schlim 1183* (holotype, W-R! 48908; isotype: W-R! 48910, UGDA-DLSz!—drawing).

**Figure 83:**
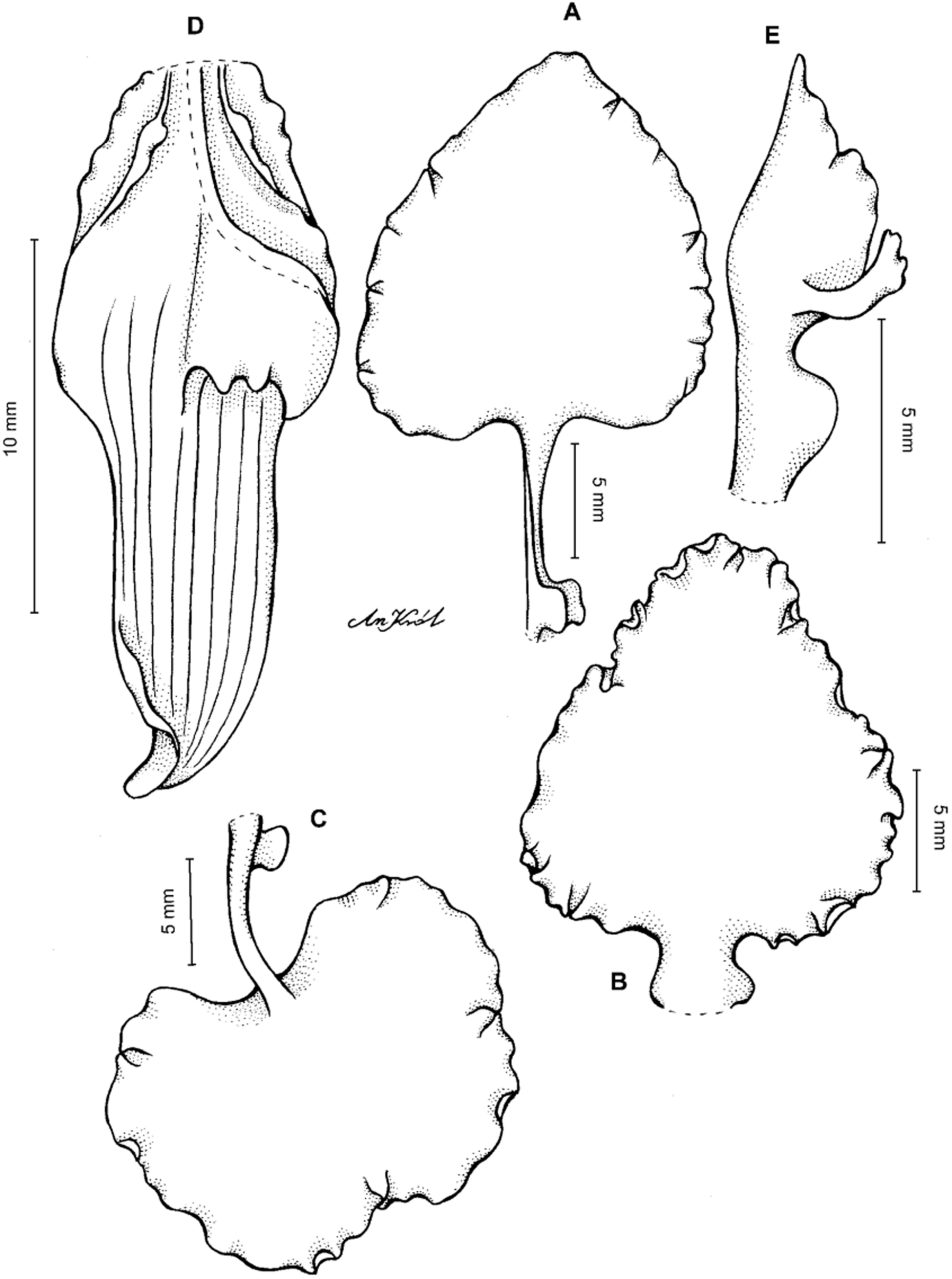
*Cyrtochilum superbiens* (Rchb.f.) Kraenzl. (A) Dorsal sepal; (B) Petal; (C) Lateral sepal; (D) Lip; (E) Gynostemium, side view. Drawn by Anna Król from *Schlim 1183* (W-R).

Pseudobulbs up to 13 cm long and 2.5 cm wide, fusiform-ovoid, enclothed basally with leafy sheaths. Leaves up to 50 cm long and 5.5 cm wide, oblanceolate, acuminate. Inflorescence wiry, branching, sparsely many-flowered. Flowers medium-sized, conspicuous. Floral bracts 15 mm long. Pedicel and ovary 38–45 mm long. Dorsal sepal clawed; claw 8 mm long, narrow, canaliculate, with prominent rhombic wings at the base; blade 18–20 mm long and wide, triangular-ovate to reniform, margins entire, more or less undulate, base truncate, apex rounded. Petals clawed; claw about 2–3 mm long, wide; blade 17–20 mm long and wide when spread, somewhat oblique, triangular-ovate, base truncate, apex obtuse, margins entire, strongly undulate. Lateral sepals prominently clawed; claw 7–10 mm long, narrow, with prominent wing at the base of outer margin; blade 15–25 mm long, 19–20 mm wide, reniform, triangular-ovate to pandurate-ovate in general outline, basally cordate to cuneate, apex truncate or emarginate, margins entire, undulate. Lip 20–25 mm long in total, curved in natural position just above the base; basal half about 7–11 mm wide, base cuneate, broadly ovate, more or less auriculate; callus consisting of a prominent, median, nose-like, papillate keel, with somewhat dissected frontal margin, additional thickened ridges running from the base of the lip to the middle of the basal part; apical part about 3.5–5 mm wide, ligulate, attenuate towards subobtuse apex. Gynostemium 10 mm long, subarcuate, connate basally with the lip, lateral appendages obliquely oblanceolate, upcurved, apical margin more or less dentate, reaching the anther base (Fig. 83).

*Ecology*: No data.

*Distribution*: Colombia, Ecuador (?), Peru (Schweinfurth, 1961; Zelenko & Bermúdez, 2009), Bolivia (Jørgensen, Nee & Beck, 2014), Venezuela (Foldats, 1968). Alt. 2,300–3,100 m.

*Notes*: This is the only species of this group having a gynostemium obliquely oblanceolate, upcurved lateral appendages with apical margin more or less dentate. By sharing longitudinal ridges running from the lip base to the middle of the basal lip part, *C. superbiens* is similar to *C. rangelii* and *C. boyacensis*. In both latter species, however, the blade of dorsal sepal is wider than long and lip callus is glabrous. Besides, the blade of petals of *C. rangelii* is also distinctly wider than long.

*Representative specimens*: COLOMBIA. **Antioqiua**: *G. Schmidtchen s.n*. (W-R! 10809, UGDA-DLSz!—drawing). Magdalena. Cerro Pintado, Sierra Perija, 3,100 m, 3–6 July 1942, *M. Carriker 50* (US!, UGDA-DLSz!—drawing). **Norte de Santander**: Pamplona, *N. Funck & Schlim 1433* (K). **Santander**: Eastern Cordillera. S slope of Mt San Martin, near Charta, 2,300–2,500 m, 10 February 1927, *E.P. Killip & A.C. Smith 19182* (US!, UGDA-DLSz!—drawing); Forested mountainside near La Cordoba, 2,830 m, 19 July 1965, *F.A. Barkley & F. Montoya 35260* (AMES!, UGDA-DLSz!—drawing).

***Cyrtochilum rangelii*** Szlach. & Kolan., Webbia 70(1): 90–94, f. 4 A-E. 2015. TYPE: Colombia. *Lozano et al. 4363* (holotype, COL! 277603; UGDA!—drawing).

**Figure 84:**
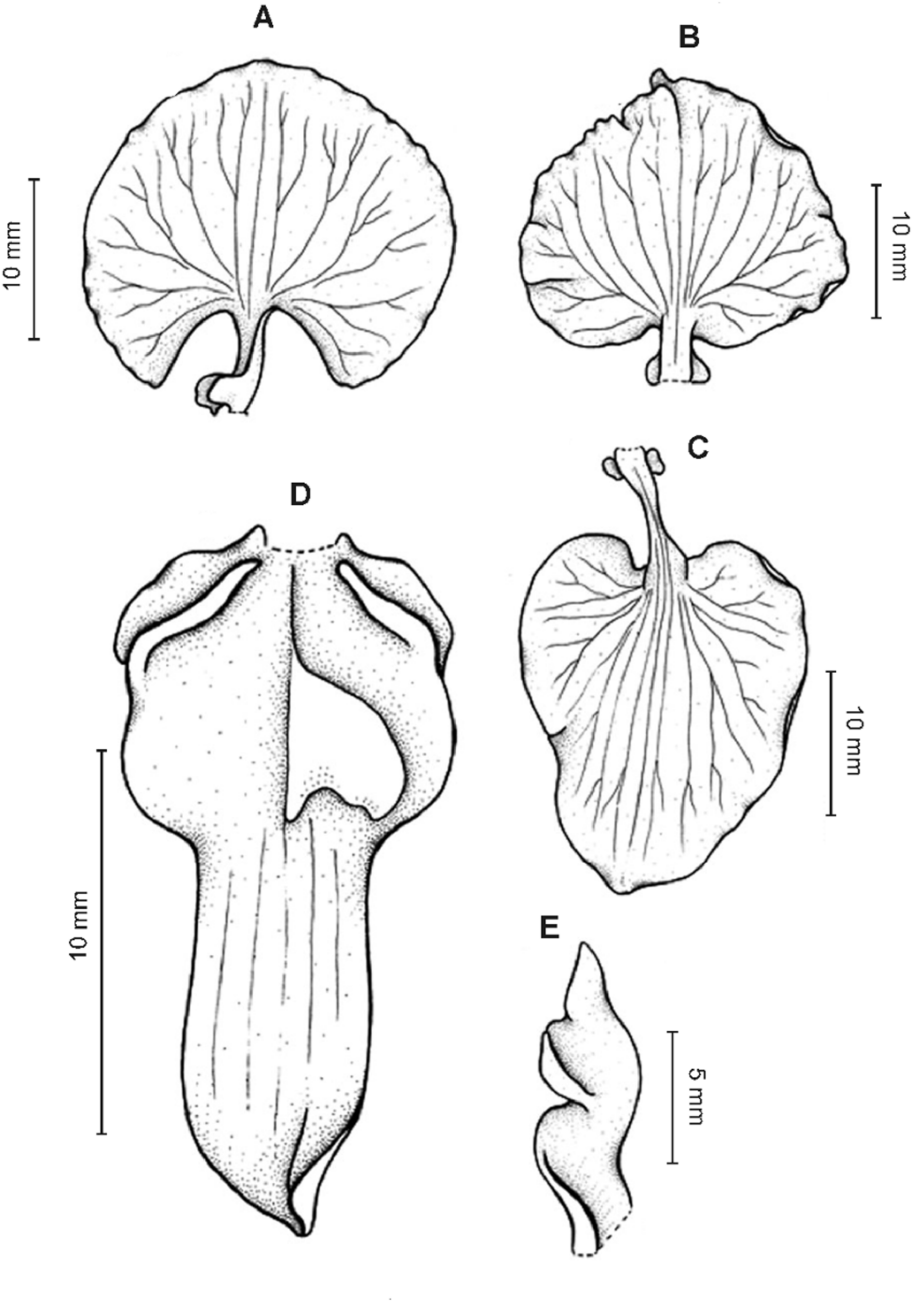
*Cyrtochilum rangeli* Szlach. & Kolan. (A) Dorsal sepal; (B) Petal; (C) Lateral sepal; (D) Lip; (E) Gynostemium, side view. Drawn by Natalia Olędrzyńska from *Lozano et al. 4363* (COL).

Plants long-rhizomatous. Pseudobulbs 11 cm long, 2 cm in diameter, oblong-ovoid, somewhat compressed, elliptic in cross-section, basally concealed by leafy sheaths. Leaves up to 50 cm long and 4.5 cm wide, linear to linear-oblanceolate, acute. Inflorescence serpentine, branching. Flowers large, conspicuous. Floral bracts 13–15 mm long. Pedicel 25 mm long, ovary about 5 mm long. Dorsal sepal clawed; claw 7 mm long, narrow, canaliculate, with prominent, rhombic wings at the base; blade 20 mm long, 26 mm wide, reniform, base sagitate, margins entire, apex obtuse. Petals clawed; claw 4 mm long, with rhombic wings; blade 18 mm long in total, 23 mm wide when spread, somewhat obliquely cordate, obtuse at the apex, margins slightly undulate. Lateral sepals clawed; claw 10 mm long, narrow, with wings at the base; blade 23 mm long, 20 mm wide, elliptic-ovate in general outline, subobtuse at the apex, cordate at the base, margins entire. Lip 18–19 mm long in total, slightly curved in natural position; basal part 8–9 mm long, 10 mm wide, transversely elliptic in outline, supported basally by oblique, thin wings, somewhat convex; callus a narrow ridge rising in the basal third being a reminiscent of a nose, with oblique thick ridges running from the lip base to the median part of lateral margins; apical part 10 mm long, 6 mm wide, ligulate, triangular towards subobtuse apex, somewhat canaliculate. Gynostemium 12 mm long, sigmoid, connate basally with the lip, lateral appendages obliquely lanceolate, falcate, reaching the anther base (Fig. 84).

*Ecology*: Terrestrial plant growing near peatbog in paramo.

*Distribution*: Colombia. Alt. 2,380 m.

*Notes*: While describing this species, we compared it with *C. mendax*, from which it differs in the form of the lip callus, which is rather simple, consisting of single ridge (vs callus composed, consisting of various segments and projections), almost the lack of undulation of the dorsal sepal and petals (vs dorsal sepal and petals prominently undulate) and the form of the dorsal sepal, which is long clawed with sagittate base (vs claw short, blade with subcordate base). It is similar to *C. superbiens* from which it can be separated by obliquely lanceolate lateral gynostemium appendages (vs obliquely oblanceolate), a sagittate base of dorsal sepal (vs truncate) and a glabrous lip callus (vs papillate). Cyrtochilum *rangelii* differs from both *C. ortizianum* and *C. boyacensis* in dorsal sepal form which is cordate at the base in these species (vs base sagittate in *C. rangelii*).

*Representative specimen*: COLOMBIA. **Huila**: Mpio. La Plata. Vereda Arrabal. Turbera de Paramo, 2,380 m, 1 October 1984, *G. Lozano, O. Rangel, De Turbay, A. Sanabria & N. Espejo 4363* (COL!; UGDA-DLSz!—drawing).

***Cyrtochilum boyacensis*** Szlach. & Kolan., Webbia 70(1): 94. 2015. TYPE: Colombia. *Diaz 1482* (holotype, COL! 105456; UGDA-DLSz!—drawing).

**Figure 85:**
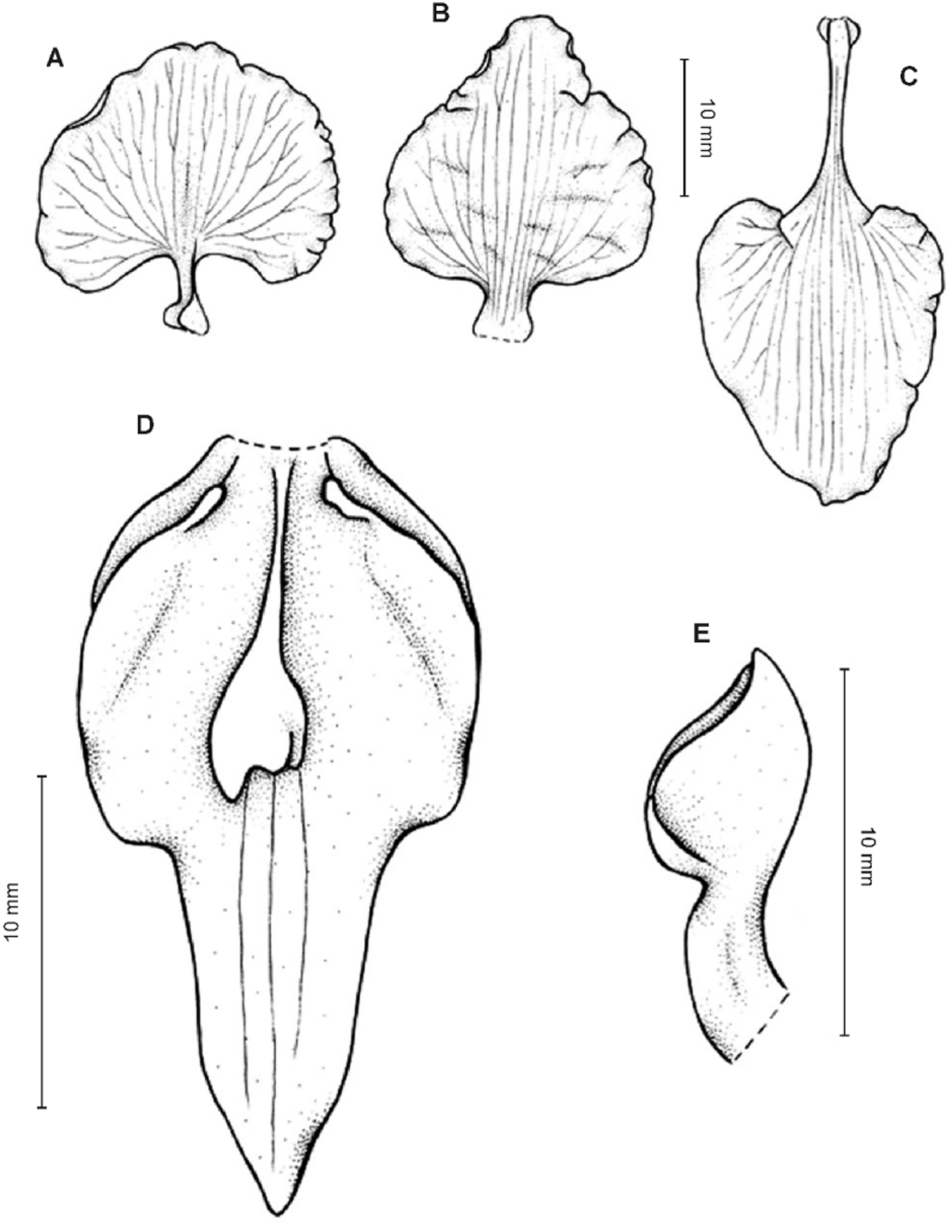
*Cyrtochilum boyacensis* Szlach. & Kolan. (A) Dorsal sepal; (B) Petal; (C) Lateral sepal; (D) Lip; (E) Gynostemium. Drawn by Natalia Olędrzyńska from *Diaz 1482* (COL).

Plants long-rhizomatous. Pseudobulbs 15 cm long, 4 cm in diameter, oblong-ovoid, somewhat compressed, elliptic in cross-section, basally concealed by leafy sheaths, bifoliate. Leaves up to 60 cm long and 6 cm wide, narrowly oblanceolate, acute. Inflorescence serpentine, branching. Flowers large, conspicuous. Floral bracts 13–15 mm long. Pedicel 25 mm long, ovary about 5 mm long. Dorsal sepal clawed; claw 8 mm long, narrow, canaliculate, with prominent, elliptic wings at the base; blade 23 mm long, 28 mm wide, reniform, base subcordate, margins undulate, apex obtuse. Petals clawed; claw 6 mm long, broad, wingless; blade 23 mm long and wide when spread, triangular-ovate, obtuse at the apex, margins undulate. Lateral sepals clawed; claw 13 mm long, narrow, with obscure wings at the base; blade 27 mm long, 23 mm wide, elliptic-ovate in general outline, subobtuse at the apex, cordate at the base, margins somewhat undulate. Lip 25 mm long in total, slightly curved in natural position; basal part 13 mm long and wide, semiorbicular in outline, supported basally by narrow wings, somewhat convex, callus a small, narrow ridge rising in the basal third or so, nose-like, somewhat hooked; apical part 10–11 mm long, 7 mm wide, oblong-triangular, subobtuse at the apex. Gynostemium 11 mm long, sigmoid, connate basally with the lip, lateral appendages obliquely lanceolate, falcate, reaching the anther base (Fig. 85).

*Ecology*: Terrestrial.

*Distribution*: Colombia. Alt. 2,650 m.

*Notes*: This species resembles *C. rangelii*, from which it differs in the form of the dorsal sepal, which is subcordate at the base (vs sagittate base), undulate sepals (vs non-undulate), triangular-ovate, wingless petals (vs obliquely cordate, with wings on the claw), a semiorbicular basal lip part (vs transversely elliptic) and oblong-triangular apical part (vs ligulate). It differs from *C. superbiens* by having entire, non-dentate lateral appendages of gynostemium (vs lateral appendages obliquely oblanceolate, apical margin more or less dentate) and a glabrous lip callus (vs papillate). Cyrtochilum *boyacensis* differs from *C. ortizianum* by having larger flowers, triangular-ovate petal blades, being obtuse at the apex (vs transversely elliptic-reniform, shortly acuminate at the apex), a claw of lateral sepals half as long as the blade (vs claw subequal in length to the blade), the basal lip part being semiorbicular (vs subrectangular-elliptic), and a apical lip part being oblong triangular (vs oblong elliptic).

*Representative specimen*: COLOMBIA. **Boyacá**: Mpio. Paipa. Vereda La Pradera. Abajo del Alto de Las Pavas, 2,650 m, 2 December 1978, *S. Diaz 1482* (COL!; UGDA!—drawing of type).

***Cyrtochilum ortizianum*** Szlach. & Kolan., Webbia 70(1): 94. 2015. TYPE: Colombia. *Caldena & al. 255* (holotype, COL! 442674; UGDA-DLSz!—drawing).

**Figure 86:**
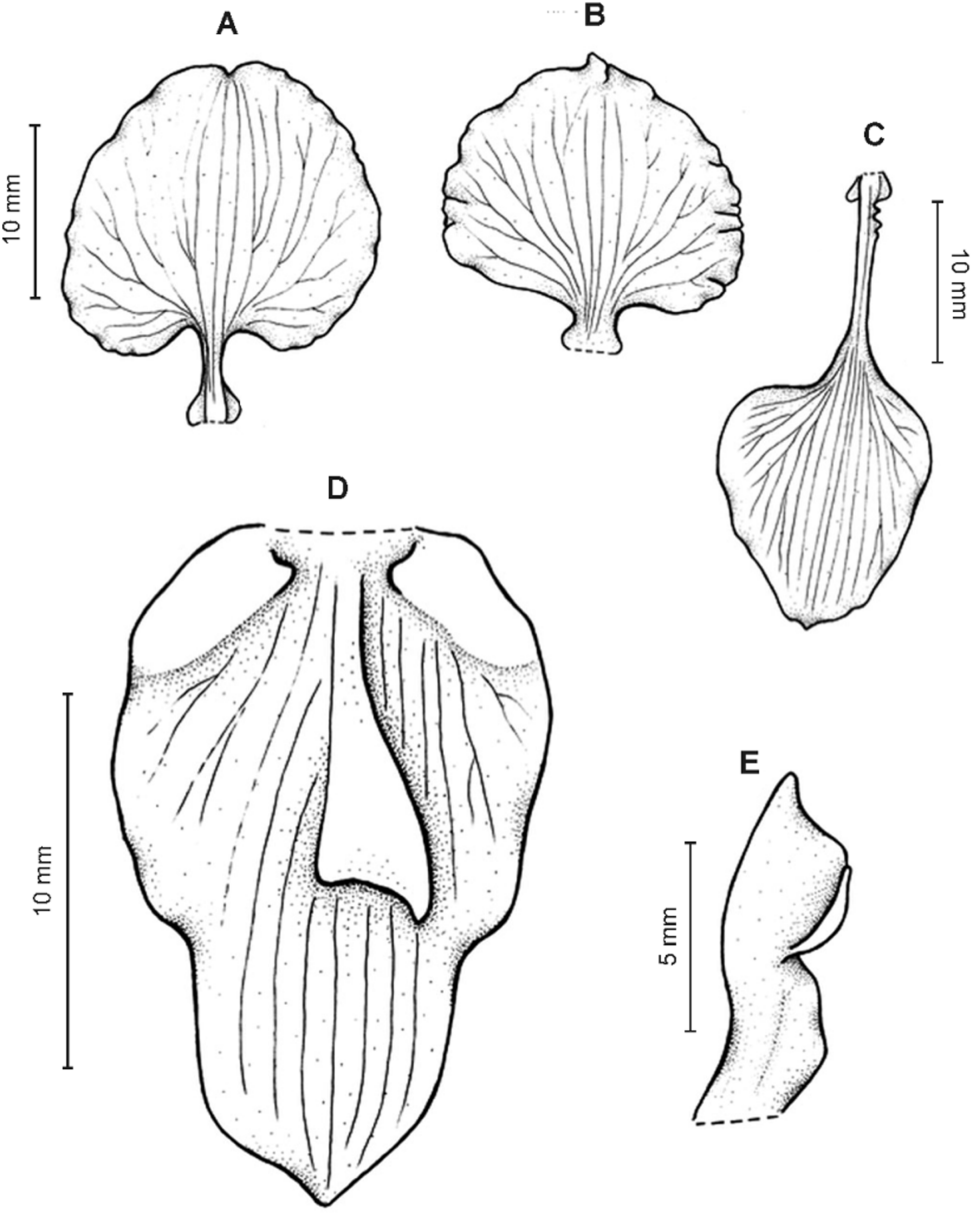
*Cyrtochilum ortizianum* Szlach. & Kolan. (A) Dorsal sepal; (B) Petal; (C) Lateral sepal; (D) Lip; (E) Gynostemium, side view. Drawn by Natalia Olędrzyńska from *Caldena et al. 255* (COL).

Plants long-rhizomatous. Pseudobulbs 15 cm long, 3.5 cm in diameter, oblong-ovoid, somewhat compressed, elliptic in cross-section, basally concealed by leafy sheaths. Leaf up to 90 cm long and 6–7 cm wide, oblanceolate, acute. Inflorescence serpentine, branching. Flowers large, conspicuous. Floral bracts 10 mm long. Pedicel 20 mm long, ovary about 5 mm long. Dorsal sepal clawed; claw 7 mm long, narrow, canaliculate, with prominent, obliquely elliptic wings at the base; blade 18 mm long and wide, elliptic-cordate in outline, base subcordate, margins flat, apex notched. Petals shortly clawed; claw 3 mm long, wingless; blade 18 mm long and wide when spread, transversely elliptic-reniform in outline, shortly acuminate at the apex, margins undulate. Lateral sepals clawed; claw 13 mm long, narrow, with obscure wings at the base, margins undulate; blade 16 mm long, 14 mm wide, obliquely elliptic-ovate in general outline, subobtuse at the apex, rounded at the base, margins flat. Lip 19 mm long in total, slightly curved in natural position; basal part 11–13 mm long, 11 mm wide, subrectangular-elliptic in outline, callus obliquely triangular in the center, somewhat hooked; apical part 6–8 mm long and wide, oblong elliptic, subobtuse at the apex. Gynostemium 9 mm long, sigmoid, connate basally with the lip, lateral appendages obliquely lanceolate, falcate, reaching the anther base (Fig. 86).

*Ecology*: Epiphytic or terrestrial in montane forest.

*Distribution*: Colombia. Alt. 2,700–3,600 m.

*Notes*: This species resembles *C. boyacensis*, but the flowers are somewhat smaller, the claw of lateral sepals is almost equal in length to the blade, petals blade is transversely elliptic-reniform, shortly acuminate at the apex (vs triangular-ovate, obtuse at the apex), basal lip part is oblong-elliptic (vs semiorbicular), and apical part is elliptic (vs oblong-triangular).

*Representative specimens*: COLOMBIA. **Santander**: Mpio. Charala. Vereda Santa Helena. Predio la Sierra, margen izquierdo aguas abajo del rio la Rusia. Santuario de Fauna y Flora. Guanenta Alto Rio Fonce, 6°01′N 73°08′W, 2,700 m, 16 December 1998, *J. Caldena, R. Galindo, H. Garcia, N. Ortiz, S. Galvan & H. Palacios 255* (COL!; UGDA!—drawing of type); Pamplona a Bucamaranga, páramo de Berlín. Terrestre con bulbos grandes, 2,800–3,600 m, 30 May 1969, *H. Garcia-Barriga & R. Jaramillo 19990* (AMES!, UGDA-DLSz!—fragment, photo, drawing).

***Cyrtochilum santanderensis*** Szlach. & Kolan., Webbia 70(1): 96. 2015. TYPE: Colombia. *Mora 4515* (holotype, COL! 471622; UGDA!—drawing).

**Figure 87:**
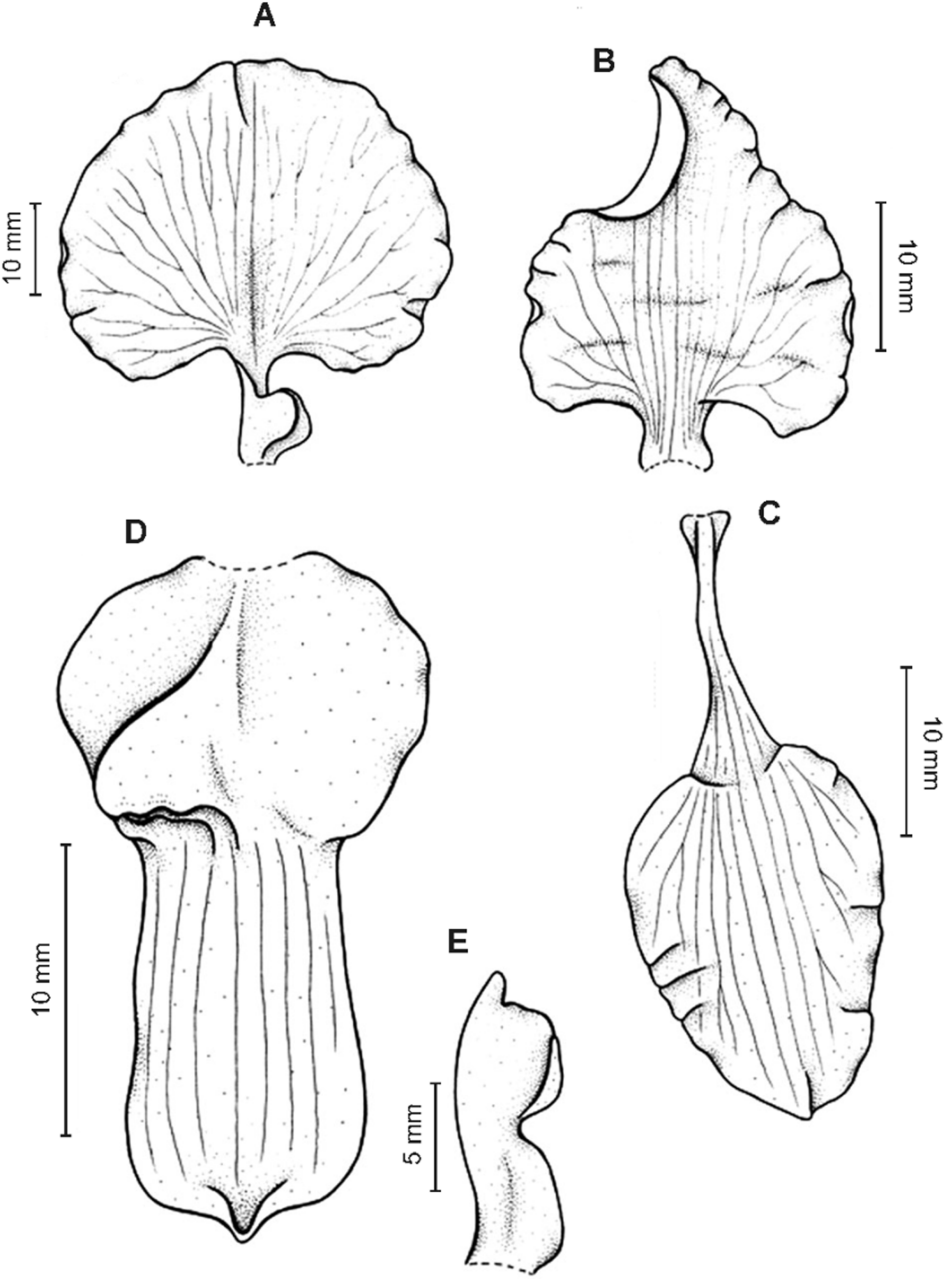
*Cyrtochilum santanderense* Szlach. & Kolan. (A) Dorsal sepal; (B) Petal; (C) Lateral sepal; (D) Lip; (E) Gynostemium. Drawn by Natalia Olędrzyńska from *Mora 4515* (COL).

Plants long-rhizomatous. Pseudobulbs 12 cm long, 4 cm in diameter, oblong-ovoid, somewhat compressed, elliptic in cross-section, basally concealed by leafy sheaths, unifoliate. Leaf up to 40 cm long and 5 cm wide, linear-oblanceolate, acute. Inflorescence serpentine, branching. Flowers large, conspicuous. Floral bracts 20 mm long. Pedicel 30 mm long, ovary 10 mm long. Dorsal sepal clawed; claw 7 mm long, narrow, canaliculate, with prominent, subquadrate wings at the base; blade 25 mm long, 28 mm wide, reniform, cordate at the base, margins somewhat undulate, apex obtuse. Petals clawed; claw 4 mm long, wingless; blade 25 mm long, 24 mm wide when spread, somewhat obliquely triangular-cordate, obtuse at the apex, margins undulate. Lateral sepals clawed; claw 15 mm long, narrow, with obscure wings at the base; blade 30 mm long, 25 mm wide, elliptic-ovate in general outline, subobtuse at the apex, rounded at the base, margins somewhat undulate. Lip 24 mm long in total, slightly curved in natural position; basal part 10 mm long, 13 mm wide, transversely elliptic in outline, somewhat convex, callus a narrow ridge rising in the basal third, nose-like; apical part 14 mm long, 7–8 mm wide, ligulate, truncate at subobtuse apex, somewhat canaliculate apically. Gynostemium 14 mm long, somewhat sigmoid, connate basally with the lip, lateral appendages obliquely lanceolate, falcate, reaching the anther base (Fig. 87).

*Ecology*: No data.

*Distribution*: Colombia. Alt. 3,000 m.

*Notes*: This species resembles *C. rangelii*, but has larger flowers, subcordate base of the dorsal sepal (vs sagittate at the base), rounded base of lateral sepals (vs cordate), claw of lateral sepals being half as long as the blade (vs claw shorter than half of the blade length), wingless claws of the petals (vs prominently winged), petals blade almost as long as wide (vs wider than long) and basal lip part lacking additional basal wings (vs supported basally by additional wings).

*Representative specimen*: COLOMBIA. **Santander**: Carretera Bucamaranga-Pamplona, 3,000 m, 7 August 1968, *L. Mora 4515* (COL!; UGDA!—drawing of type).

**(9) *Ioplocon-group***

Inflorescence short in relation to other species of the genus, usually reaching 1 m or so length. Tepals narrow, clawed, undulate. Lip relatively narrow, with complicated callus. Gynostemium strongly sigmoid, connate with the lip in its lower third, apically swollen.

Eight species of this group were reported from Colombia, with only few representatives from Venezuela and Ecuador.

### Key to the species

1 Gynostemium only basally connate with the lip, erect or suberect, winged, without any lateral appendages2
1* Gynostemium sigmoid, in the basal third or quarter connate with the lip, with prominent appendages below stigma3
2 Lip callus large, with long, linear or ligulate projections*C. dipterum*
2* Lip callus consisting of large, main body and tuft of short digitate processes in front*C. ioplocon*
3 Gynostemium lateral appendages very prominent, obliquely elliptic-lanceolate, with two, filamentous projections on the upper margin*C. sibundoyense*
3* Gynostemium lateral appendages obliquely triangular, apical margins irregularly dentate4
4 Lip callus consisting of parallel lamellae or ridges running from the base of the gynostemium to the base of the apical part of the lip5
4* Lip callus consisting of thick, undivided tissue at the base, more or less palmate at the apex7
5 Lip callus consisting of 5 narrow, oblong projections of variable length, two lateral apically widened and wing-like*C. praetermissum*
5* Lip callus consisting of 3 parallel lamellae6
6 Lamellae separated from each other by prominent grooves*C. refractum*
6* All lamellae closely together, touching each other*C. huertasii*
7 Lip callus a 3-lobed, fleshy pad situated between apical and basal parts of the lip with the presence of two additional mamillate projections near the lip centre on each side of the gynostemium, tepals strongly undulate*C. ospinae*
7* Lip callus consisting of massive, thick, marginally wrinkled mass, tepals weakly undulate*C. divaricatum*

***Cyrtochilum huertasii*** Szlach. & Kolan., Wulfenia 21: 59–60, f. 3A–E. 2014. TYPE: Colombia. *Huertas & Camargo 6308* (holotype, COL! 173029, UGDA-DLSz!—drawing).

**Figure 88:**
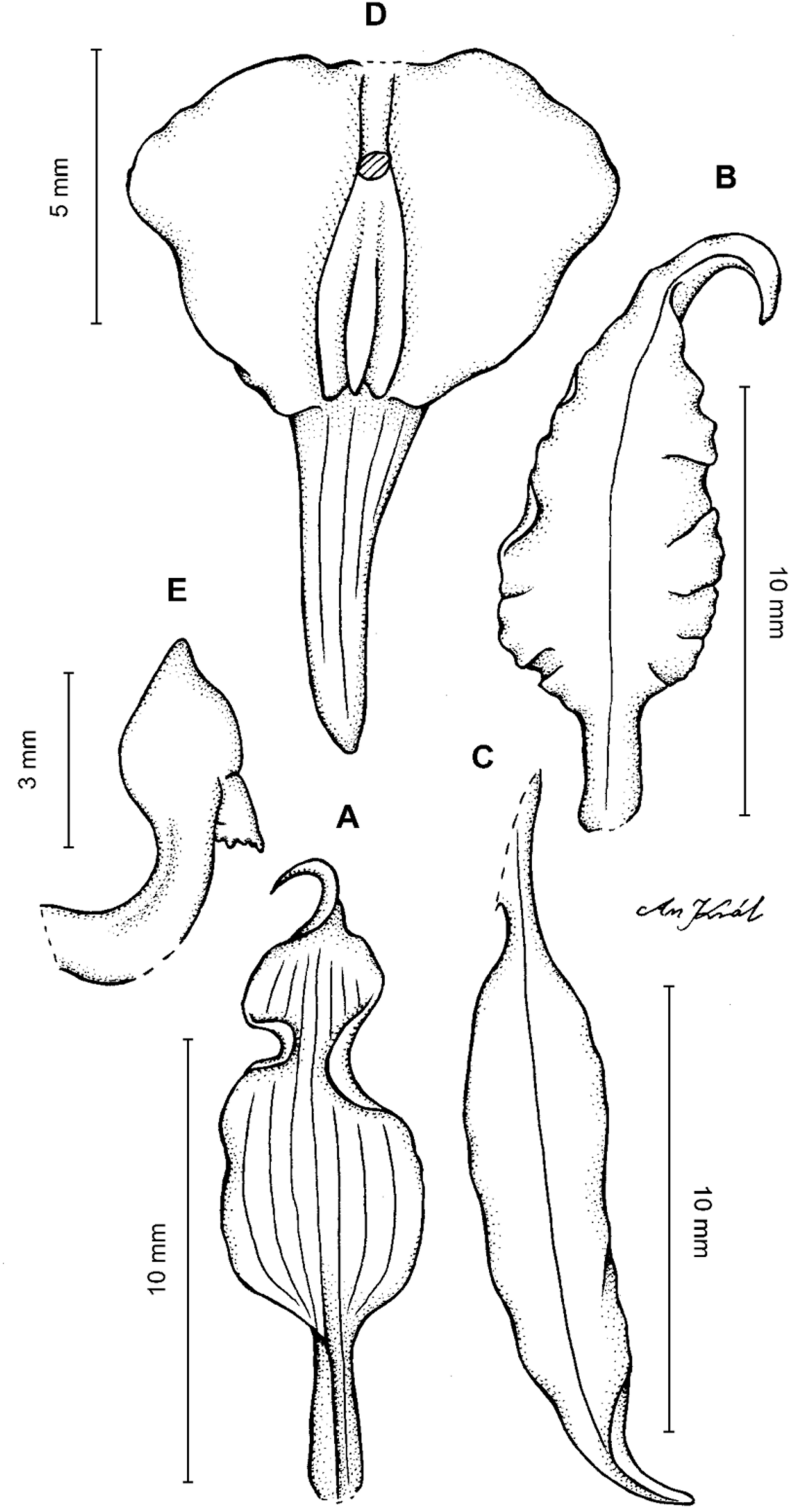
*Cyrtochilum huertasii* Szlach. & Kolan. (A) Dorsal sepal; (B) Petal; (C) Lateral sepal; (D) Lip; (E) Gynostemium. Drawn by Anna Król from *Huertas & Camargo 6308* (COL).

**Figure 89:**
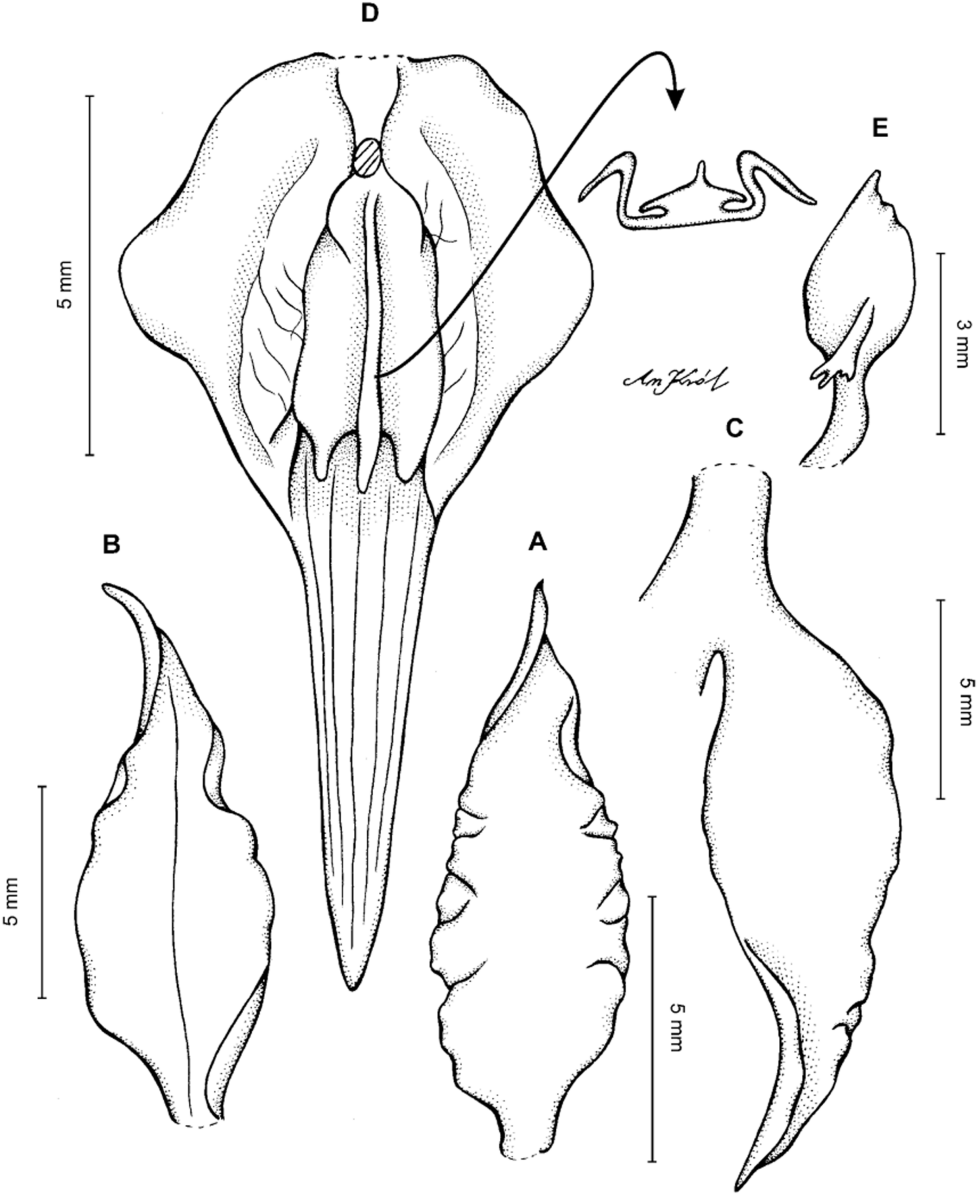
*Cyrtochilum huertasii* Szlach. & Kolan. (A) Dorsal sepal; (B) Petal; (C) Lateral sepal; (D) Lip; (E) Gynostemium. Drawn by Anna Król from *Rivera & Barkley 18A190* (US).

Pseudobulbs 5.8–7.2 cm long, strongly compressed, ovoid, enveloped basally by several persistent, leaf bearing sheaths. Leaves 30 cm long and 2.5 cm wide, linear-oblanceolate, acute. Inflorescence 60–100 cm long, branching. Flowers medium-sized, conspicuous, tepals coffee- or reddish-coloured, with transversal yellow markings, lip yellow. Floral bracts 10–11 mm long. Pedicel 25 mm long, ovary about 8–10 mm long. Dorsal sepal clawed; claw 4 mm long, rather narrow, canaliculate, wingless; blade up to 12 mm long and 5 mm wide, oblong elliptic to elliptic-ovate, somewhat twisted, apex long-acuminate, recurved, margins entire, undulate. Petals shortly clawed; claw 3 mm long, wingless; blade up to 12 mm long and 5 mm wide, ovate-lanceolate, long-acuminate, acute at the recurved apex, somewhat oblique, margins undulate. Lateral sepals clawed; claw 3–4 mm long, narrow, connate in the basal 2/3 together along internal margin; blade up to 15 mm long, 3–5 mm wide, obliquely oblong-lanceolate, long-acuminate, acute at recurved apex, margins entire, flat or somewhat undulate. Lip 10–15 mm long in total, slightly curved in natural position; basal part up to 7 mm long and 9 mm wide, deltoid to transversely elliptic, convex; callus limited to the narrow central part, consisting of 3 narrow ridges running from the base of the gynostemium to the base of the lip apical part; apical part 6–7 mm long, 2 mm wide, linear, subobtuse. Gynostemium 5–6 mm long, strongly sigmoid, connate in the basal fourth with the lip, hence appearing as growing from the centre of the basal lip part; lateral appendages obliquely triangular, apical margins irregularly dentate, pendant (Figs. 88 and 89).

*Ecology*: Terrestrial or epiphytic.

*Distribution*: Colombia. Alt. 1,740–2,850 m.

*Notes*: This species resembles *C. divaricatum* from wich it is distinguishable by the lip shape (deltoid vs transversely elliptic) and the callus location (limited to the central part vs occupying a large part of the lip base) and form (made of 3 narrow ridges vs consisting of a massive, thick, marginally wrinkled mass). Cyrtochilum *huertasii* can be separated from *C. praetermissum* by the lip callus which consists of 3 parallel ridges (vs 5 narrow, oblong projections various in length, two lateral of them are apically widened and wing-like). Cyrtochilum *huertasii* differs from similar *C. refractum* by lamellae forming callus being close together and touching each other (vs lamellae separated by prominent grooves in *C. refractum*).

*Representative specimens*: COLOMBIA. **Antioquia**: 1 km al S de Hoyo Rico, 2,600 m, 26 September 1948, *F. Rivera & F. Barkley 18A190* (US!, UGDA-DLSz!—drawing). **Boyacá**: Mpio. Arcabuco. Alrededores de poblacion, 2,850 m, 20 October 1965, *G. Huertas & L. Camargo 6308* (COL!), Vía Moniquira-Arcabuco. Desvio a la derecha hacia la reserva forestal El Peligro, entre Moniquira y Gachantiva, 2,700 m, 14 May 1996, *J.L. Fernandez & Sist. Veg. 14282* (COL!). **Cauca**: Bei Popayán, 1,740 m, 28 February 1884, *F.C. Lehmann 3595* (US!, UGDA-DLSz!—drawing). **Putumayo**: Road between San Francisco and Mocoa, km 79 from Pasto, 2,800 m, 30 October 1974, *T. Plowman & E. Davis 4319* (COL!). **Valle del Cauca**: La Nevera, 26 January 1980, *I. Guarin O. 88* (COL!).

***Cyrtochilum praetermissum*** Szlach. & Kolan., J. Torrey Bot. Soc. 144(1): 92. 2016(2017). TYPE: Colombia. *Llanos & Gerardino 2836* (holotype, COL! 425723, UGDA-DLSz!—drawing).

**Figure 90:**
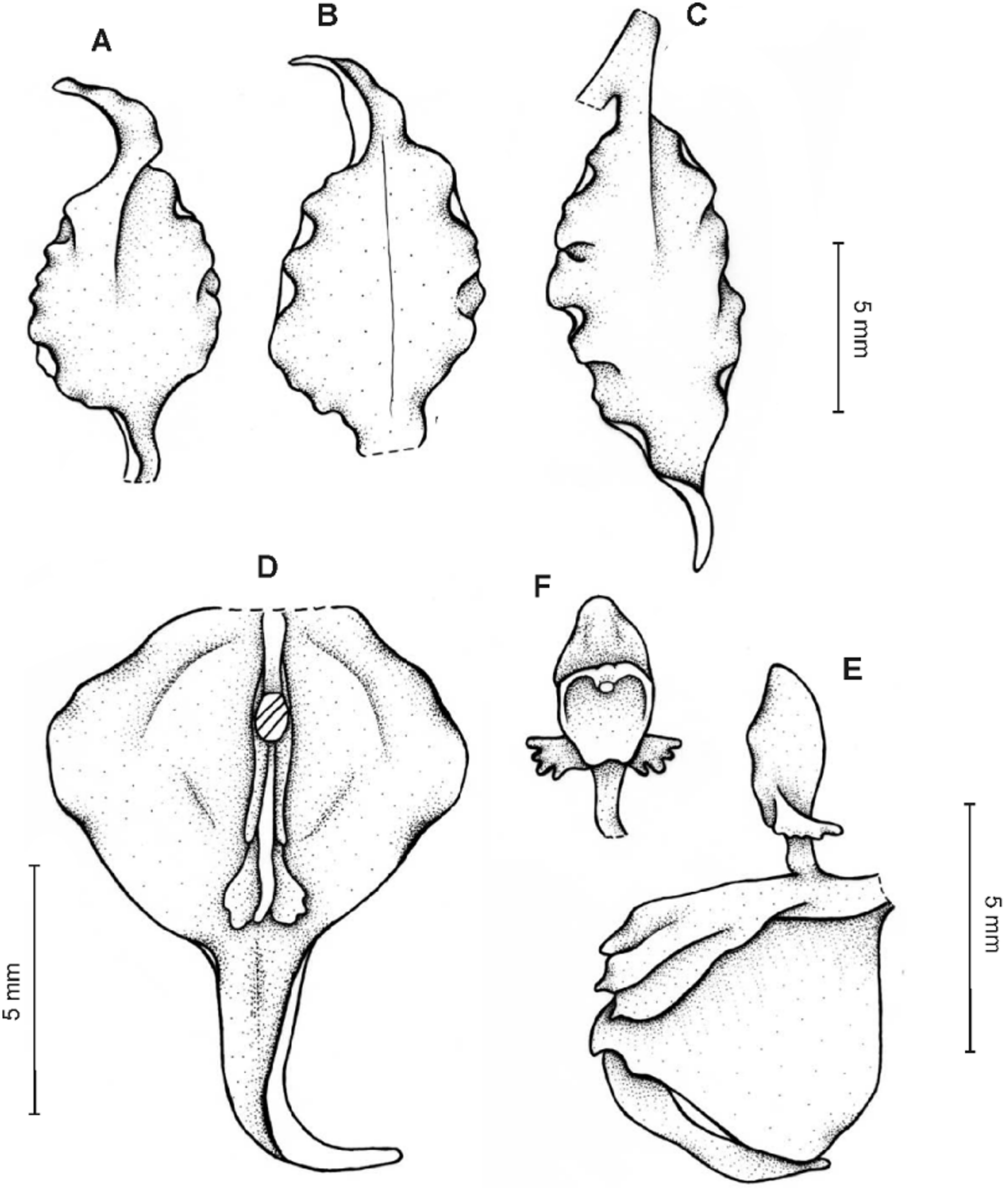
*Cyrtochilum praetermissum* Szlach. & Kolan. (A) Dorsal sepal; (B) Petal; (C) Lateral sepal; (D) Lip; (E) Gynostemium and lip, side view; (F) Gynostemium, ventral view. Drawn by Natalia Olędrzyńska from *Llanos & Gerardino 2836* (COL).

Plants long-rhizomatous. Pseudobulbs up to 8 cm long, about 1 cm in diameter, laterally compressed, more or less elliptic in cross section, concealed basally by leafy sheaths, bifoliate. Leaf about 30 cm long, 2.5 cm wide, linear-oblanceolate, acute. Inflorescence long, serpentine, branching. Flowers medium-sized, conspicuous. Floral bracts 10 mm long. Pedicel 20 mm long, ovary about 7 mm long. Dorsal sepal shortly clawed; claw 3 mm long, narrow, canaliculate, with obscure wings at the base; blade 15 mm long, 5–6 mm wide, elliptic-lancelate to ovate-lanceolate, base rounded, margins strongly undulate, apex acuminate, recurved. Petals shortly clawed; blade 14 mm long, 5 mm wide when spread, elliptic-ovate, base rounded, acuminate at the apex, margins strongly undulate. Lateral sepals shortly clawed; claw 3 mm long, narrow, without basal wings, connate together; blade 18–20 mm long, 5–6 mm wide, obliquely ovate-lanceolate in general outline, rounded basally, acuminate at the apex, margins strongly undulate. Lip 12–13 mm long in total, curved in natural position; basal part 6–7 mm long, 8–9 mm wide, transversely rhombic, convex; callus along the midvein only, consisting of 5 narrow, oblong projections various in length, the two lateral apically widened and wing-like; apical part 6 mm long, 2.5 mm wide, ligulate, gradually attenuating towards apex, subobtuse, curved down. Gynostemium 4 mm long, sigmoid, connate in the basal third to the lip, lateral appendages obliquely obtriangular, irregularly denticulate along apical margins, directed backwards (Fig. 90).

*Ecology*: Growing in montane and high-montane forest.

*Distribution*: Colombia. Alt. 1,800–2,600 m.

*Notes*: The species resembles *C. divaricatum*, from which it is easily separable by a much smaller callus occupying the narrow part along the midvein of the basal lip part (vs massive callus occupying larger part of the basal lip part) and gynostemium projections directed backward (vs forward). It can be misidentified with *C. huertasii*, *C. refractum* and *C. ospinae* but has a different lip callus. In all of those species callus is formed of 3 lamellae or thick mass.

*Representative specimens*: COLOMBIA. **Cundinamarca**: Via Bogotá-Choconta. Represa del Sisga. Pasando el Puente Metalico, 2,600 m, 20 August 1994, *J.L. Fernandez, H. Cardozo & M. Arevalo 11592* (COL!). **Huila**: Mpio. Neiva. Vereda la plata, carretera a Balsillas, cercanias a la finca La Carolina, 1,800 m, 1 Novomber 1996, *F. Llanos & W. Gerardino 2836* (COL!, UGDA-DLSz!—drawing).

***Cyrtochilum refractum*** (Rchb.f.) Kraenzl., Notizbl. Bot. Gart. Berlin-Dahlem 7: 92. 1917. ≡ *Oncidium refractum* Rchb.f., Bonplandia (Hannover) 2: 12. 1854. TYPE: Colombia. [New Grenada]. *Purdie s.n*. (holotype, W-R! 36955; isotypes: AMES! 76197, W-R! 36958, UGDA-DLSz!—drawing).

**Figure 91:**
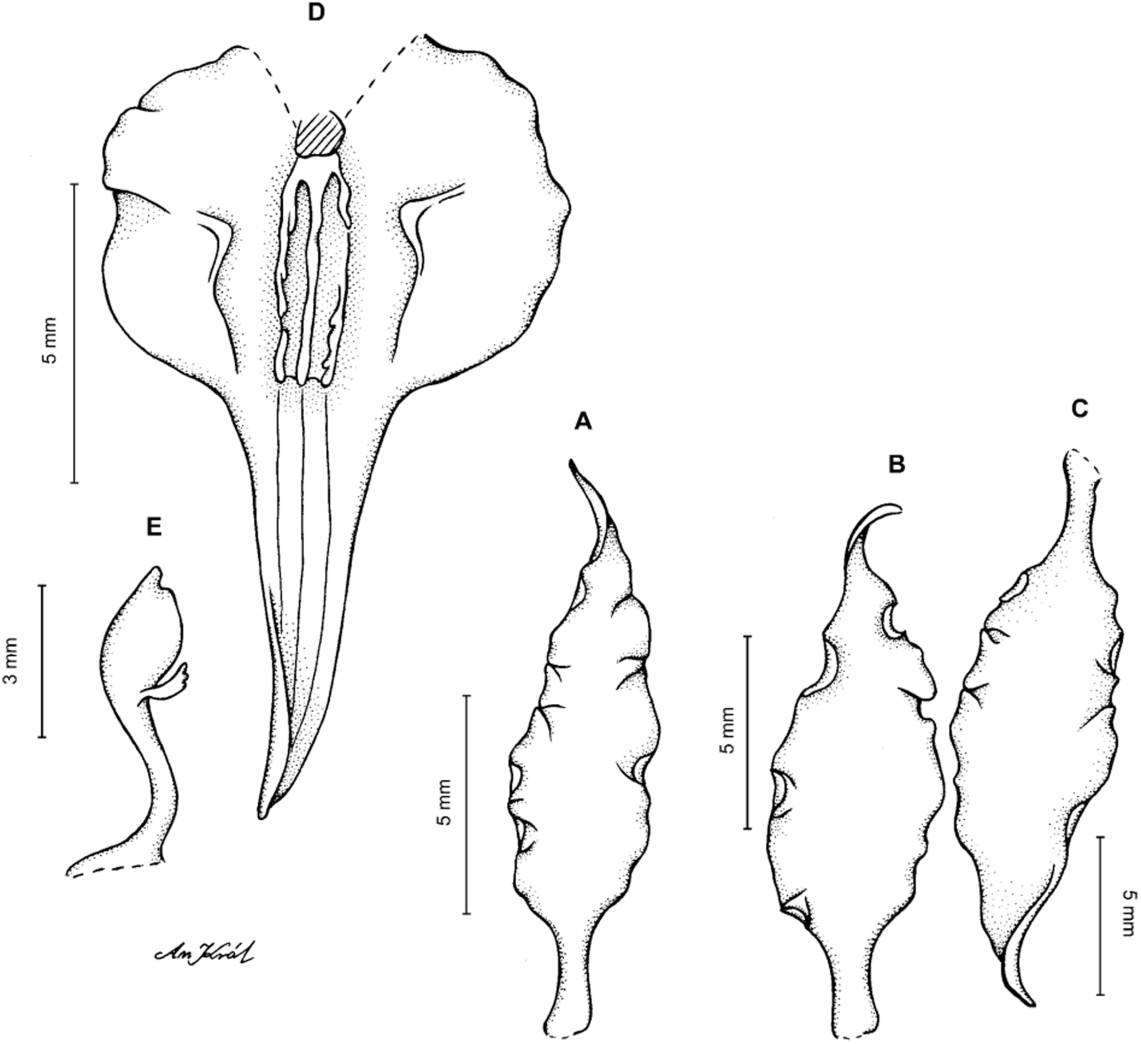
*Cyrtochilum refractum* (Rchb.f.) Kraenzl. (A) Dorsal sepal; (B) Petal; (C) Lateral sepal; (D) Lip; (E) Gynostemium, side view. Drawn by Natalia Olędrzyńska from *Purdie s.n*. (W-R).

Pseudobulbs 6 cm long, 2 cm wide, oblong ovoid, enclothed basally in some leafy bracts, somewhat compressed, bifoliate. Leaves up to 35 cm long and 2.5 cm wide, linear-oblanceolate, acuminate. Inflorescence up to 150 cm long, branching, sparsely many-flowered. Flowers medium-sized. Floral bracts 10–13 mm long. Pedicel and ovary 18–35 mm long. Dorsal sepal clawed; claw 3–4 mm long, narrow, canaliculate, wingless; blade 10–16 mm long, 3.5–4.8 mm wide, lanceolate, margins undulate, base cuneate, apex acuminate, somewhat recurved. Petals clawed; claw about 3 mm long; blade 10–13 mm long, 4–5.5 mm wide when spread, oblique, lanceolate to lanceolate-ovate, base cuneate, apex acuminate, somewhat recurved, margins undulate. Lateral sepals prominently clawed; claw 3–4 mm long, narrow, wingless, basally connate together; blade 14–16 mm long, 3.5–4.5 mm wide, obliquely oblong lanceolate in general outline, basally cuneate, apex acuminate, somewhat recurved, margins undulate. Lip 13 mm long in total, 7–8 mm wide at the base, curved in natural position just above the base, basal half much extended to form transversely elliptic to deltoid lamina; callus consisting of 3 parallel lamellae, with prominent grooves on both sides of central callus; apical half linear-triangular, acuminate. Gynostemium 4.5–6 mm long, sigmoid, connate basally with the lip, lateral appendages obliquely oblanceolate, lower margins dentate, not reaching the anther base (Fig. 91).

*Ecology*: No data.

*Distribution*: Colombia. Alt. 1,500–2,500 m.

*Notes*: The unique character of this species is the lip callus consisting of 3 parallel lamellae with grooves between them. 3-lamellate lip callus can also be found in *C. huertasii*, but in this case they are close together, touching each other.

Dalström (2001) considered this species as conspecific with *C. divaricatum* in which the lip callus consists of thick, undivided tissue at the base and more or less palmate tissue at the apex.

*Representative specimens*: COLOMBIA. **Antioquia**: Boqueron, camino entre Medellin y Palmitas, 2,300–2,500 m, 22 March 1948, *W. Johnson & F. Barkley 18C492* (US!, UGDA-DLSz!—drawing). **Cauca**: Highlands of Popayán, 1,500–1,800 m, May–June, *F.C. Lehmann 8568* (AMES!, UGDA-DLSz!—drawing). [New Grenada], *W. Purdie s.n*. (W-R! 36955 & 36958, AMES!, UGDA-DLSz!—drawing); *J. Linden* 683 (W-R!, UGDA-DLSz!—drawing).

***Cyrtochilum ospinae*** Szlach. & Kolan., Wulfenia 21: 56. 2014. TYPE: Colombia. *Ospina H. 1599* (holotype, COL! 546216, UGDA-DLSz!—drawing).

**Figure 92:**
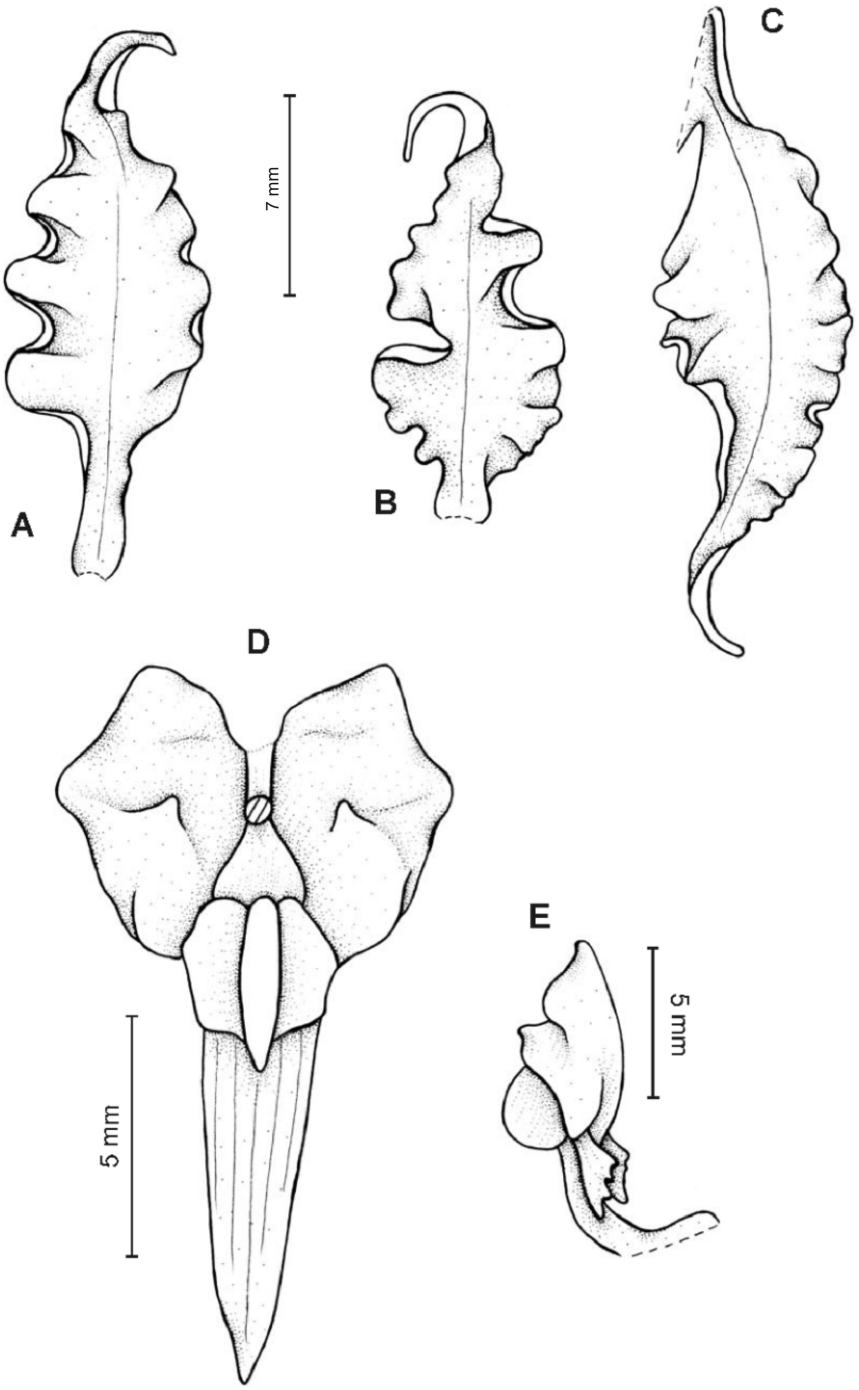
*Cyrtochilum ospinae* Szlach. & Kolan. (A) Dorsal sepal; (B) Petal; (C) Lateral sepal; (D) Lip; (E) Gynostemium, side view. Drawn by Natalia Olędrzyńska from *Ospina H. 1599* (COL).

Pseudobulbs about 6 cm long, strongly compressed, oblong ovoid, enveloped basally by several persistent, leaf bearing sheaths. Leaves 30 cm long and 3.5 cm wide, linear-oblanceolate, acute. Inflorescence long, branching. Flowers medium-sized, tepals coffee- or yellow-coloured, with yellow, transverse blotches, lip coffee-coloured, internal part yellow, apical part sometimes brownish-red, callus white. Floral bracts 10 mm long. Pedicel 25 mm long, ovary about 8–10 mm long. Dorsal sepal clawed; claw 4 mm long, rather narrow, canaliculate, wingless; blade 15 mm long, 6 mm wide, ovate-lanceolate, long-acuminate, apex recurved, margins strongly undulate. Petals shortly clawed; claw 2 mm long, wingless; blade 12 mm long, 5–6 mm wide, ovate-lanceolate, long-acuminate, acute at the recurved apex, somewhat oblique, margins strongly undulate. Lateral sepals clawed; claw 5 mm long, narrow, connate together along internal margin; blade 16 mm long, 6 mm wide, obliquely lanceolate-ovate, long-acuminate, acute at recurved apex, margins strongly undulate. Lip 14–15 mm long in total, slightly curved in natural position; basal part 7 mm long, 9 mm wide, transversely rhombic, convex, callus in form of 3-lobed fleshy pad between apical and basal parts of the lip, middle lobe elevated, ridge-like, two additional mamillate projections near the lip centre on each side of the gynostemium; apical part 6–7 mm long, 1.5–2 mm wide, lanceolate-triangular, acute. Gynostemium 7 mm long, strongly sigmoid, connate in the basal fourth with the lip, hence appearing as growing from the centre of the basal lip part; lateral appendages obliquely triangular, apical margins irregularly dentate, curved back (Fig. 92).

*Ecology*: Terrestrial.

*Distribution*: Colombia. Alt. 2,000–2,300 m.

*Notes*: This species resembles *C. divaricatum*, from which it differs by having strongly undulate tepals, and a unique lip callus. It is 3-lobed, fleshy pad situated between apical and basal parts of the lip with the presence of two additional mamillate projections near the lip centre on each side of the gynostemium. In *C. divaricatum* tepals are weakly undulate and the lip callus consists of a massive, thick, marginally wrinkled mass. Both of these species can be confused with *C. praetermissum*, *C. refractum* and *C. huertasii*, but in all of them lip callus consists of parallel lamellae or ridges running from the base of the gynostemium to the base of the apical part of the lip.

*Representative specimens*: COLOMBIA. **Antioquia**: Carretera entre Las Palmas y Rio Negro. Laderas del Rio Negro, 2,300 m, 12 August 1957, *M. Ospina H. 193a* (COL!, UGDA-DLSz!—drawing). **Boyacá**: Carretera Garagoa-Miraflores, 2,000 m, 5 September 2005, *M. Ospina H. 1599* (COL!, UGDA-DLSz!—drawing). **Cundinamarca**: Pacho. Vereda San Miguel, 2,000 m, 14 October 1990, *M. Ospina H. 1253* (COL!).

***Cyrtochilum divaricatum*** (Lindl.) Dalström, Lindleyana 16: 63. 2001. ≡ *Odontoglossum divaricatum* Lindl., Orchid. Linden.: 17. 1846. TYPE: Venezuela. *Linden 683* (holotype, K). ≡ *Oncidium divaricatum* (Lindl.) Beer, Prakt. Stud. Orchid.: 285. 1854, *nom. illeg*.

= *Cyrtochilum costatum* (Lindl.) Kraenzl., Notizbl. Bot. Gart. Berlin-Dahlem 7: 98. 1917. ≡ *Odontoglossum costatum* Lindl., Fol. Orchid. 1: 11. 1852. TYPE: Colombia. [New Grenada]. *Schlim & Funk s.n*. (syntype: W-R! 36957; UGDA-DLSz!—drawing); *Schlim s.n*. (Syntype: W-R! 36957), *Schlim & Funk 1028* (syntype: W-R! 36957, UGDA-DLSz!—drawing). ≡ *Oncidium costatum* (Lindl.) Beer, Prakt. Stud. Orchid.: 285. 1854.

**Figure 93:**
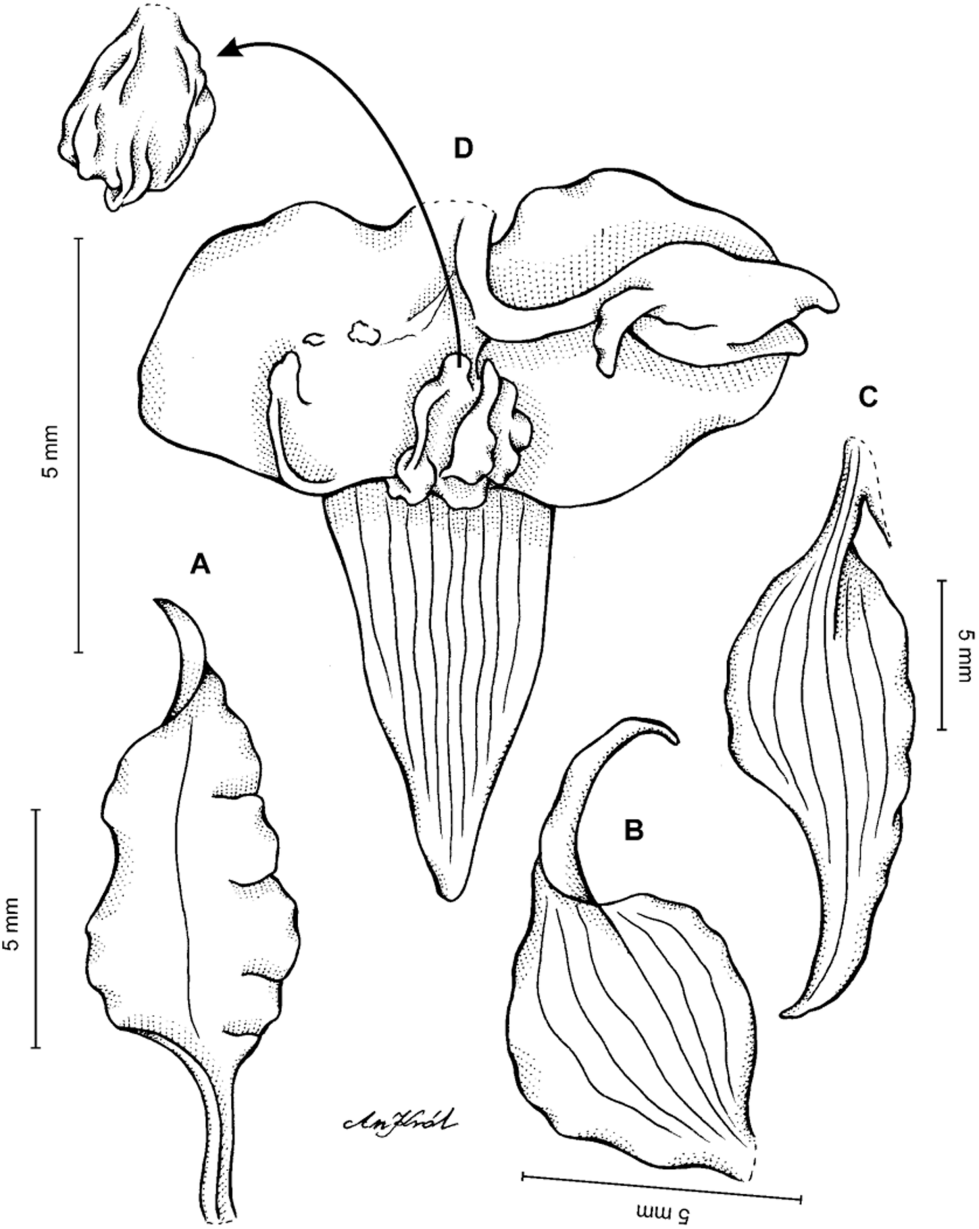
*Cyrtochilum divaricatum* (Lindl.) Dalström. (A) Dorsal sepal; (B) Petal; (C) Lateral sepal; (D) Lip. Drawn by Anna Król from *Schlim & Funk s.n*. (W-R)—type of *Cyrtochilum costatum* (Lindl.) Kraenzl.

Pseudobulbs distant along creeping rhizome, 4–8 cm long, up to 2.5 cm in diameter, ovoid, somewhat compressed, subtended basally by leafy sheaths. Leaves 1–2, 25–40 cm long, 1.5–2.5 cm wide, oblong-oblanceolate, acute. Inflorescence 200 cm long, paniculate, erect, wiry, flexuose in the upper part, with well-spaced flexuous few-flowered branches. Flowers relatively small, brown or brownish, lip yellow, callus white. Floral bracts 10–14 mm long. Pedicel and ovary 25–30 mm long. Dorsal sepal clawed; claw 3–5 mm long, narrow, canaliculate, non-winged; blade 10–14 mm long, 4–5 mm wide, lanceolate-ovate, base broadly cuneate, margins undulate, entire, apex acute, recurved. Petals subsessile; blade 11–16 mm long in total, 4.5–5 mm wide when spread, ovate-lanceolate, acute at the apex and recurved, oblique, margins entire, undulate. Lateral sepals clawed; claw about 5–6 mm long, narrow, non-winged, connate together in the basal half; blade about 12–15 mm long, 4–4.5 mm wide, obliquely elliptic-lanceolate in general outline, base cuneate, apex acute, recurved, margins entire. Lip 9–15 mm long in total, curved in natural position exposing irregular callus; callus consisting of pad-like tissue with palmate, irregular apex; basal part 4–6 mm long, 8–10 mm wide when spread, transversely elliptic in outline, with some thickenings; apical part 5–7 mm long, 2.3–3 mm wide, oblong-obtriangular, subacute, thick. Gynostemium 4.5–5 mm long, sigmoid, connate basally with the lip, swollen at the apex, lateral appendages obliquely lanceolate, with irregular lower margin (Fig. 93).

*Ecology*: Growing within disturbed roadside vegetation and along stream.

*Distribution*: Colombia, Ecuador (Dalström, 2010), Venezuela. Alt. 1,860–2,600 m.

*Notes*: This species has unique a lip callus, which in central part is thick, pad-like, and in apex dissected in narrow, acute segments, more or less palmate.

*Representative specimens*: COLOMBIA. **Antioquia**: Near Carman. Cordillera Central, 2,000 m, 25 April 1948, *A. Ferrara, J. Noval & F. Barkley 18C515* (US! UGDA-DLSz!—drawing); Mpio. Frontino. Km 10 of road Nutibara-Murri, 6°46′N 76°21′W, 1,970 m, 26 September 1987, *J. Zarucchi., A. Brant & C. Castano 5793* (MO!); Along road between Jerico and Tamesis, about 5 mi NW of Tamesis, along stream below huge outcrop, 5°42′N 75°44′W, 1,920 m, 26 January 1990, *T. Croat 70031* (MO!); Mpio. Urrao. Road to Pabon, 18.9 km S of Urrao-Betulia road, remaining roadside trees, 6°10′N 76°09′W, 1,960 m, 31 October 1987, *A. Brant & J. Betancur 1551* (MO!, UGDA-DLSz!—drawing). **Cundinamarca**: Eiphytic in mossy forest between Fosca and Gutierrez, 2,600 m, 5 September 1960, *H. Schmidt-Mumm 1147* (MO!). **Santander**: La Corcova (Tona), 1,866 m, 12 October 1977, *E. Renteria, N. Sierra, E. Mantilla & V. de Merchan 657* (MO!). [New Grenada]. Aqua Obi[…]. *L. Schlim & N. Funk s.n*. (W-R! 36957; UGDA-DLSz!—drawing); *L. Schlim s.n*. (W-R! 36957) & Truxillo. *L. Schlim & N. Funk 1028* (W-R! 36957). VENEZUELA. **Lara**: *G. Davidse & A. Gonzalez 21140* (AMES!, UGDA-DLSz!—drawing). *Sine loc., J. Linden 683* (K).

***Cyrtochilum sibundoyense*** Szlach. & Kolan., ***sp. nov***. TYPE: Colombia. *Ospina H. 139* (holotype, COL! 62633, UGDA-DLSz!—drawing).

Diagnosis: This s*pecies is similar to Cyrtochilum ioplocon from which it differs in the transversely pandurate basal lip part, a lip being slightly widened near the rounded apex and a callus consisting of palmate segments flanked by large, rectangular thickenings*.

**Figure 94:**
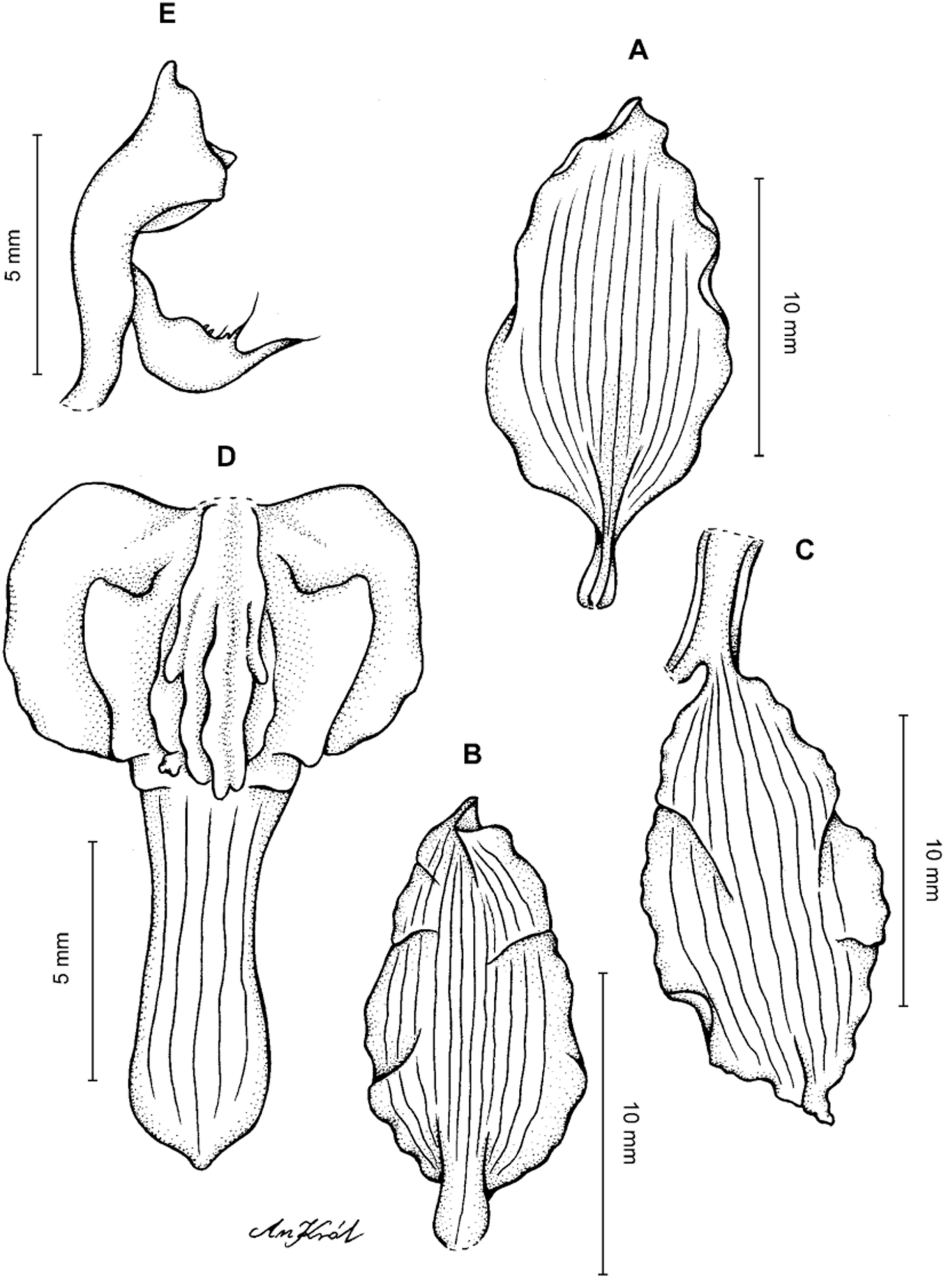
*Cyrtochilum sibundoyense* Szlach. & Kolan. (A) Dorsal sepal; (B) Petal; (C) Lateral sepal; (D) Lip; (E) Gynostemium, side view. Drawn by Anna Król from *Ospina 139* (COL).

Inflorescence paniculate, erect, wiry, with well-spaced few-flowered, fractiflex branches. Flowers rather small. Floral bracts 13 mm long. Pedicel and ovary 35 mm long. Dorsal sepal clawed; claw 3.5 mm long, narrow, canaliculate, wingless; blade 15 mm long, 9 mm wide, oblong elliptic, base broadly cuneate, margins distantly undulate, entire, apex obtuse, straight. Petals clawed; claw 2 mm long; blade 13 mm long, 7–8 mm wide when spread, oblong ovate, acute at the apex, oblique, margins entire, very slightly undulate. Lateral sepals clawed; claw about 5 mm long, narrow, wingless, connate together in the major part; blade 16 mm long, 8 mm wide, obliquely oblong obovate in general outline, base cuneate, apex acute, margins entire, slightly undulate. Lip 14–15 mm long in total, curved in natural position exposing irregular callus; callus consisting of palmate segments, flanked by large, rectangular thickenings; basal part 6 mm long, 8–9 mm wide when spread, transversely pandurate in outline; apical part 8–9 mm long, 3 mm wide, oblong-ligulate, rounded at the apex. Gynostemium 7.5 mm long, sigmoid, connate basally with the lip, swollen at the apex, lateral appendages very prominent, obliquely elliptic-lanceolate, with two filamentous projections on the upper margin (Fig. 94).

*Etymology*: In reference to the place of the origin of the type specimen.

*Ecology*: Growing in montane forest.

*Distribution*: Colombia. Alt. 2,800 m.

*Notes*: The unique characters of this species are pince-like gynostemium appendages. *Cyrtochilum ioplocon* differs from the new species in the lip form and callus structure. The basal lip part of *C. ioplocon* is almost transversely elliptic (vs transversely pandurate) and it is acuminate at the apex (vs slightly widened near the rounded apex). Its callus consists of massive, nose-like tissue, with a tuft of short digitate processes in front (vs palmate segments flanked by large, rectangular thickenings).

*Representative specimen*: COLOMBIA. **Putumayo**: Bosque alto al N del valle de Sibundoy, hacia la mina de San Francisco, Alt. 2,800 m, 9 January 1957, *M. Ospina H. 139* (COL!, UGDA-DLSz!—drawing).

*Conservation status*: This species is described based on material collected ca. 60 years ago. We cannot assess the current threats for this taxon hereby the “data deficient” (DD) category should be applied according to IUCN (2001).

***Cyrtochilum ioplocon*** (Rchb.f.) Dalström, Lindleyana 16: 66. 2001. ≡ *Odontoglossum ioplocon* Rchb.f., Gard. Chron., n.s., 21: 445. 1884. TYPE: Probably Colombia. *Bull 833* (holotype, W-R! 30411; UGDA-DLSz!—drawing).

**Figure 95:**
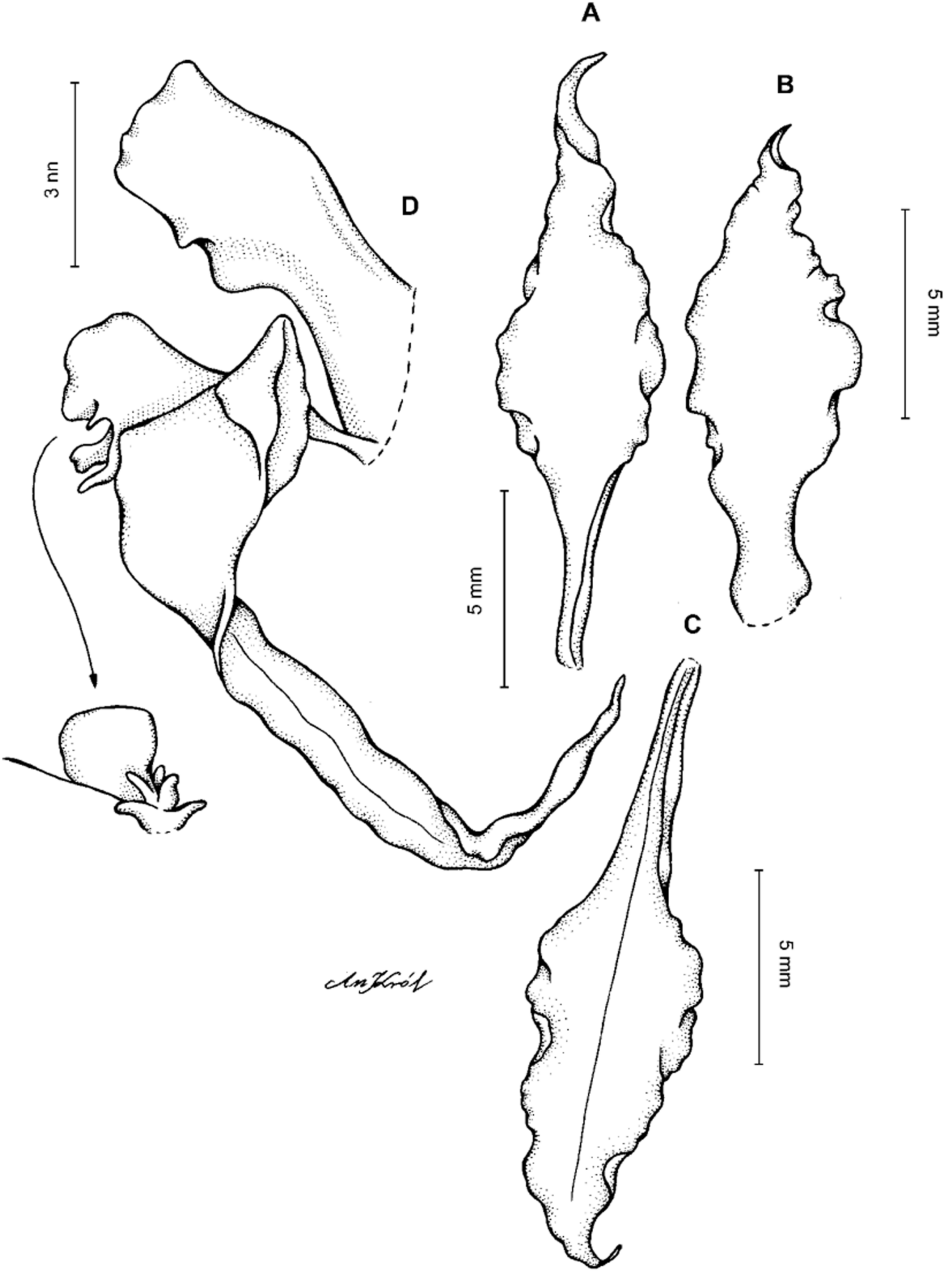
*Cyrtochilum ioplocon* (Rchb.f.) Dalström. (A) Dorsal sepal; (B) Petal; (C) Lateral sepal; (D) Lip and gynostemium, side view. Drawn by Anna Król from *Bull 833* (W-R).

Pseudobulbs 7–8 cm long, 1.2–1.3 cm in diameter, narrowly ovoid, subtended basally by leafy sheaths. Leaves 2, up to 56 cm long and 3 cm wide, linear-oblanceolate, acute. Inflorescence up to 80 cm long, an erect to arching panicle with spreading, few-flowered branches. Flowers large, conspicuous, showy. Floral bracts 3–4 mm long. Pedicel and ovary about 13–14 mm long. Dorsal sepal shortly clawed; claw 4 mm long, rather narrow, canaliculate, wingless; blade 12–13 mm long, 4–5.5 mm wide, lancolate to ovate-lanceolate, base cuneate, margins more or less undulate, apex acuminate, recurved. Petals clawed; claw about 2–3 mm long; blade 10–11 mm long, 4–5.5 mm wide when spread, obliquely oblong ovate, acute-acuminate at the apex, margins undulate. Lateral sepals prominently clawed; claw 6–8 mm long, narrow, wingless; blade 10–12 mm long, 4–4.5 mm wide, lancolate to ovate-lanceolate, somewhat oblique, basally cuneate, acute at the apex, margins more or less undulate. Lip 12–14 mm long in total, curved in natural position exposing massive, nose-like callus, with a tuft of short digitate processes in front; basal part about 5.5–8 mm wide, almost transversely elliptic, base truncate; apical part 2–2.6 mm wide, linear, attenuate towards apex, acuminate. Gynostemium 6 mm long, erect to somewhat arcuate, relatively massive, winged, basally connate with the lip, without any appendages (Fig. 95).

*Ecology*: Terrestrial in paramo and roadsides.

*Distribution*: Colombia. Alt. 2,000–3,300 m.

*Notes*: *Cyrtochilum ioplocon* can be easily distinguished from the species of this group by a relatively massive gynostemium with no lateral appendages. In this respect it is similar to *C. dipterum* which has much longer callus segments, and a massive main callus body.

*Representative specimens*: COLOMBIA. **Cundinamarca**: Mpio. Junin. Corregimiento inspeciones Chuscales, Reserva Carpanta, 4°35′N 73°40′W, 2,500–3,300 m, 27 April 1988, *J. Luteyn, O. Rangel & J. Aguirre 12235* (COL!, UGDA-DLSz!—drawing); About 8 km N of Gutierrez, Alt. 2,600 m, 5 September 1960, *W. Hatheway & H. Schmidt-Mumm 1135* (COL!, UGDA-DLSz!—drawing); Patio Bonito. Batatas Valley. Cordillera de Helicona. 12 km SE of Gachala, Alt. 2,560 m, 22 September 1944, *M. Grant 10227* (COL!, UGDA-DLSz!—drawing); Carretera Fosca-Gutierrez, Alt. 2,000 m, 10 June 1984, *M. Ospina H. 1122* (COL!). **Quindio**: Carretera Calarcá-Cajamarca. 1 km al W del paso de la Linea. Orilla de la carretera, 4°30′N 75°33′W, Alt. 3,150 m, 23 June 2003, *R. Bernal & G. Galeano 3345* (COL!, UGDA-DLSz!—drawing).

***Cyrtochilum dipterum*** (Lindl.) Kraenzl., Notizbl. Bot. Gart. Berlin-Dahlem 7: 99. 1917. ≡ *Odontoglossum dipterum* Lindl., Orchid. Linden.: 16. 1846. TYPE: Colombia [New Grenada]. *Linden 1277* (holotype, K; isotypes: BR, P, W-R! 48758; UGDA-DLSz!—drawing). ≡ *Oncidium dipterum* (Lindl.) Beer, Prakt. Stud. Orchid.: 285. 1854.

= *Odontoglossum calodryas* Rchb.f., Linnaea 41: 3. 1876. TYPE: Colombia [New Grenada]. *Roezl s.n*. (holotype, W-R! 41701; UGDA-DLSz!—drawing).

**Figure 96:**
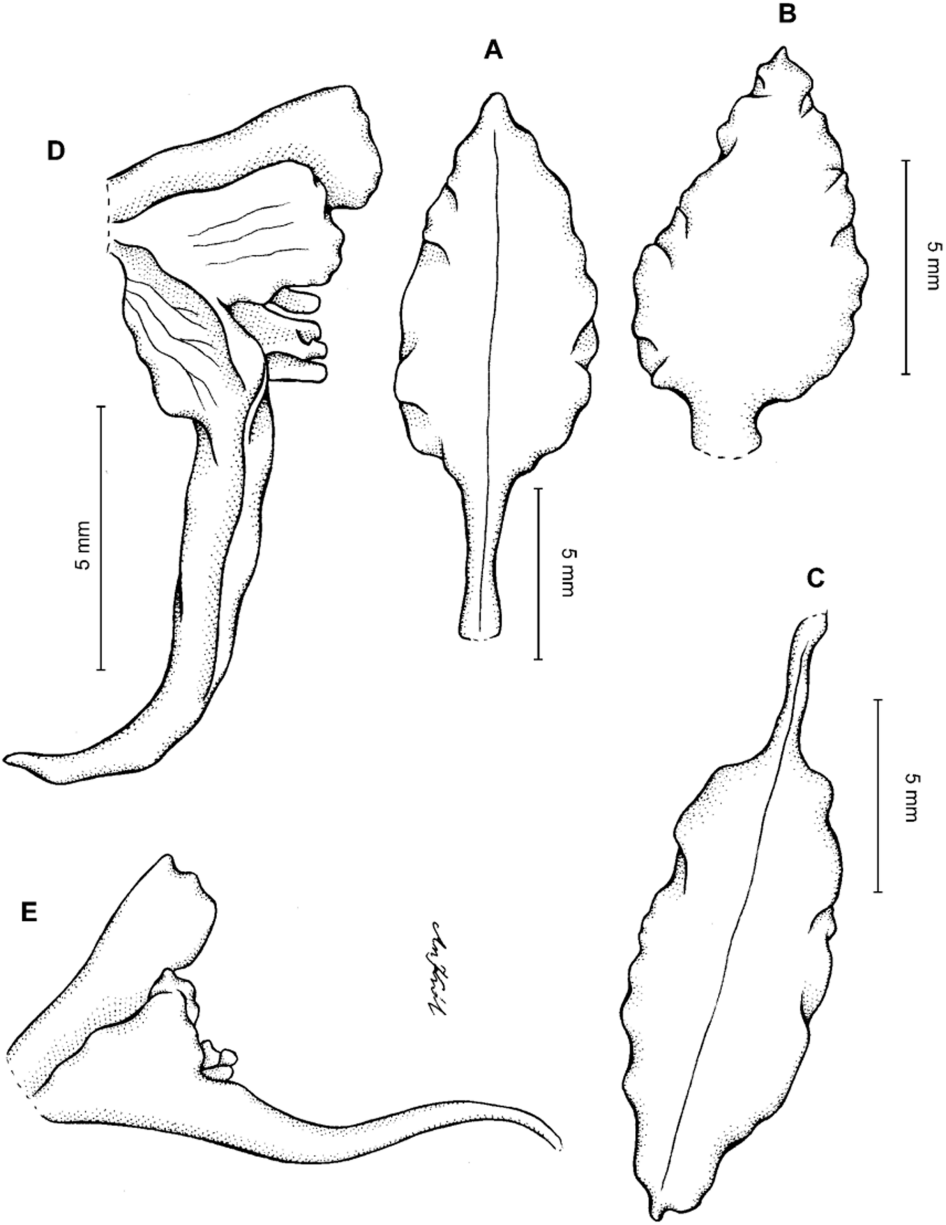
*Cyrtochilum dipterum* (Lindl.) Kraenzl. (A) Dorsal sepal; (B) Petal; (C) Lateral sepal; (D) Lip; (E) Gynostemium and lip, side view. Drawn by Anna Król from *Linden 1277* (W-R).

**Figure 97:**
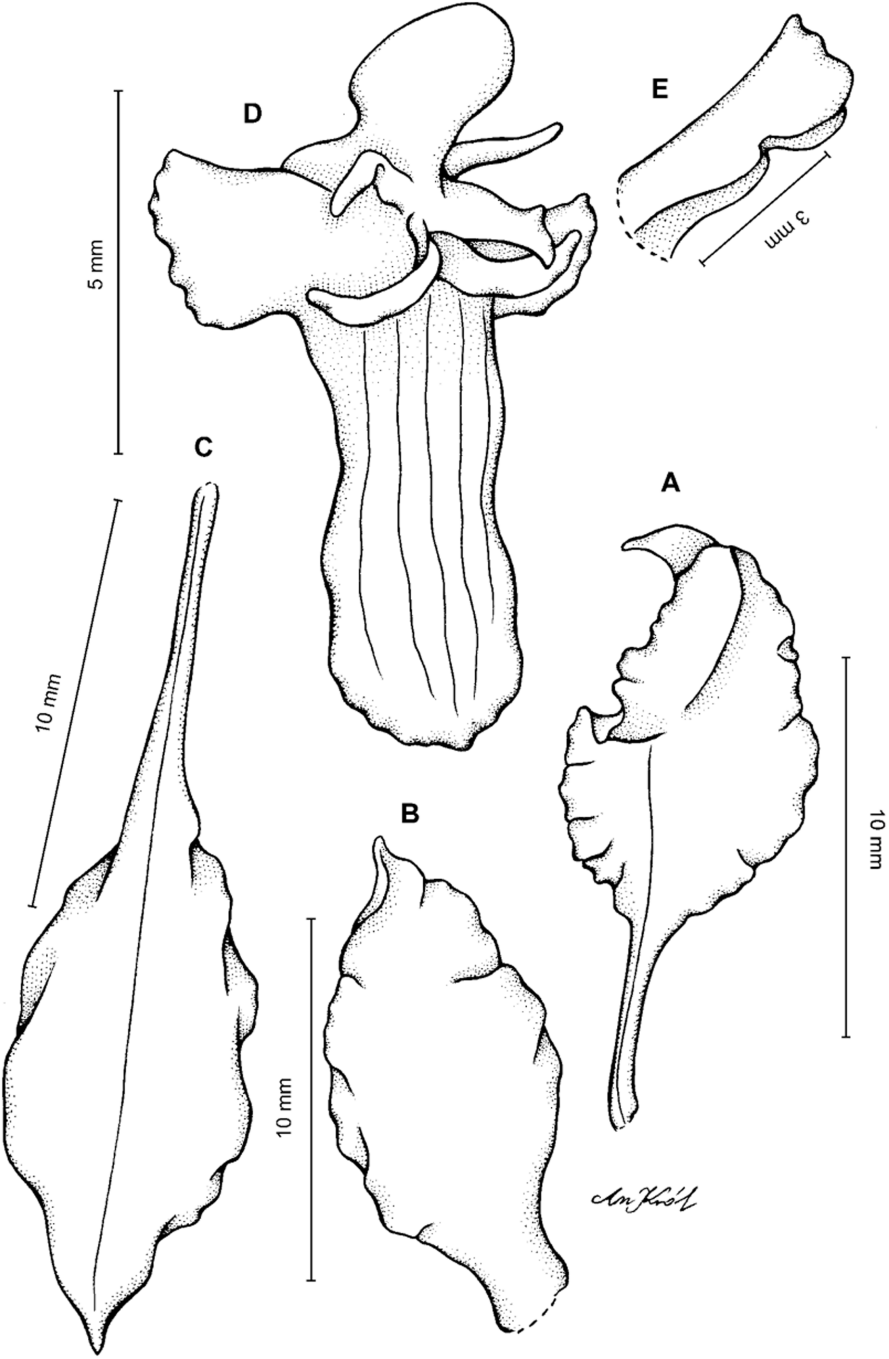
*Cyrtochilum dipterum* (Lindl.) Kraenzl. (A) Dorsal sepal; (B) Petal; (C) Lateral sepal; (D) Lip; (E) Gynostemium. Drawn by Anna Król from *Roezl s.n*. (W-R)—type of *Odontoglossum calodryas* Rchb.f.

Rhizome ascending. Pseudobulbs 4–6 cm long, 2–3 cm in diameter, ovoid, slightly compressed, smooth, subtended basally by foliaceous sheaths. Leaves 15–35 cm long, 2–2.5 cm wide, narrowly obovate, acuminate. Inflorescence up to 50 cm long, an erect to arching panicle with spreading, flexuose few-flowered branches. Flowers medium-sized, tepals purple with white base, lip yellow with yellow callus. Floral bracts 8–10 mm long. Pedicel and ovary 20–30 mm long. Dorsal sepal clawed; claw 6–7 mm long, narrow, canaliculate, wingless; blade 11–13 mm long, 5.5–6.5 mm wide, elliptic-ovate to lanceolate-ovate, base cuneate, margins more or less undulate, entire, apex acute, recurved. Petals broadly clawed; claw about 2 mm long; blade 12–15 mm long, 6 mm wide when spread, elliptic-ovate to oblong ovate, acute at the apex, oblique, margins somewhat undulate, more or less entire. Lateral sepals prominently clawed; claw 6–10 mm long, narrow, wingless; blade 11–13 mm long, 5–6 mm wide, obliquely elliptic-lanceolate in general outline, base cuneate, apex acute, margins somewhat undulate. Lip 8–11 mm long in total, pendent just above the base, prominently 3-lobed; basal part up to 7.5 mm long, transversely elliptic; callus very large, complicated with central, large knob-like outgrowth supported in front with digitate, prominent projection and additionally flanked by two pairs of long, linear to ligulate projections on both sides; apical part ligulate to narrowly triangular in general outline, obtuse to acute. Gynostemium 5 mm long, erect, connate basally with the lip, lateral appendages transformed into wings flanking gynostemium (Figs. 96 and 97).

*Ecology*: Epiphytic or terrestrial.

*Distribution*: Colombia, Ecuador. Alt. 2,600 m.

*Notes*: *Cyrtochilum dipterum* appears to be closely related to *C. ioplocon*, but has a much larger lip callus with long, linear or ligulate projections (vs tuft of short digitate processes in front of nose-like callus).

*Representative specimens*: COLOMBIA. **Antioquia**: *Sine loc., G. Schmidtchen s.n*. (W-R! 11378, UGDA-DLSz!—drawing). *Sine loc., G. Wallis s.n*. (W-R! 41700, UGDA-DLSz!—drawing), *Sine loc., J. Linden 1277* (BR, K, P, W-R! 48758; UGDA-DLSz!—drawing), *Sine loc., B. Roezl s.n*. (W-R! 41701; UGDA-DLSz!—drawing). **Caldas**: Páramo de Letras. Terrestre, entre arbustos bajos, Alt. 3,200 m, *M. Ospina H. 61–2* (AMES!, UGDA-DLSz!—drawing). ECUADOR**. Tungurahua**: Slopes of Volcán Tungurahua, Alt. 2,500–2,900 m, *F.C. Lehmann s.n*. (K, NY—Dalström, 2010).

**(10) *Volubile*-group**

Lip large, shield-like.

Five species of this group are known mostly from Peruvian Andes, with only a few reaching as far north as Ecuador and Colombia.

### Key to the species

1 Disc with callus consisting of 3–5 thick ridges2
1* Disc with central pad-like tissue apically surrounded by free tubercles of various sizes3
2 Tepals flat, lip obtuse*C. caquetanum*
2* Tepals somewhat undulate, lip subacute to acuminate*C. leopoldianum*
3 Lip broadly unguiculate at base*C. loxense*
3* Lip sessile4
4 Lip central callus terminating in three lobules forming a small chamber*C. villenaorum*
4* Lip central callus dentate apically*C. volubile*

***Cyrtochilum loxense*** (Lindl.) Kraenzl., Notizbl. Bot. Gart. Berlin-Dahlem 7: 95. 1917. ≡ *Oncidium loxense* Lindl., Paxt. Fl. Gard. 2: 128. 1851. TYPE: Ecuador. *Hartweg s.n*. (K-L, W).

Pseudobulbs 10–12 cm long, 2.5–5 cm wide, caespitose to distant, narrowly oblong-ovoid, subcompressed, clothed below by a few pairs of evanescent, long, leaf-bearing sheaths. Leaves 1–2, 30–50 cm long, 2.5–8 cm wide, oblanceolate, acute or short-acuminate. Inflorescence up to 300 cm long, elongate, climbing or twining, loosely paniculate. Flowers large, showy, sepals and petals brown or brownish with greenish yellow stripes, lip rich yellow. Floral bracts 5–10 mm long, small, triangular-ovate, clasping, obtuse. Pedicellate ovary up to 60 mm long. Dorsal sepal shortly clawed; claw wingless; blade up to 30 mm long, about 15–18 mm wide, ovate, subobtuse to subacute, margins flat. Petals subsessile; blade 23–25 mm long, 14–19 mm wide, obliquely elliptic, acute, distantly undulate. Lateral sepals clawed; claw 6–8 mm long, completely connate; blade 28–30 mm long, 15–20 mm wide, obliquely elliptic-ovate, subacute, margins flat. Lip 23–25 mm long, 25–28 mm wide near the apex, broadly unguiculate at base; apical lobe broadly rounded to transversely elliptic, apical margin truncate; disc at the base with longitudinal ridge surrounded at the apex by several free, short tubercles. Gynostemium about 10 mm long, arcuate, slender, lateral appendages obscure, digitate.

*Ecology*: Epiphytic in upper montane cloud forest.

*Distribution*: Ecuador, Peru. Alt. 2,700 m.

*Notes*: This very peculiar taxon described from Loja, Ecuador, can be easily distinguished from all other representatives of the genus by unique combinations of tepals colour, basally connate lateral sepals, lip morphology, and lip callus.

*Representative specimens*: ECUADOR. **Loja**: Loja, *C. Hartweg s.n*. (K-L, W); Pahuay in Loja, *J. Kuhn 114* (SEL—Dalström, 2010); Cajanuma, 2,700 m, *J. Kuhn s.n*. (SEL—Dalström, 2010), the same loc., *C. Dodson & G. Frymire 327* (SEL—Dalström, 2010); cult. Sanchez in Cuenca *sub A. Hirtz et al. 5075* (QCNE—Dalström, 2010). PERU. Near Moyobamba, *A. Mathews 1918* (K).

***Cyrtochilum volubile*** Poepp. & Endl., Nov. Gen. Sp. Pl. 1: 35, t. 61. 1836. TYPE: Peru. *Poeppig 1636* (holotype, W! 0047831; isotypes: W! 0047830, W-R! 5054 & 44653, UGDA-DLSz!—drawing). ≡ *Oncidium volubile* (Poepp. & Endl.) Cogn. *in* C.F.P.von Martius & auct. suc. (eds.), Fl. Bras. 3(6): 289. 1905.

= *Oncidium corynephorum* Lindl., Sert. Orchid.: t. 25. 1838. TYPE: Peru. *Mathews 1335* (holotype, K).

**Figure 98:**
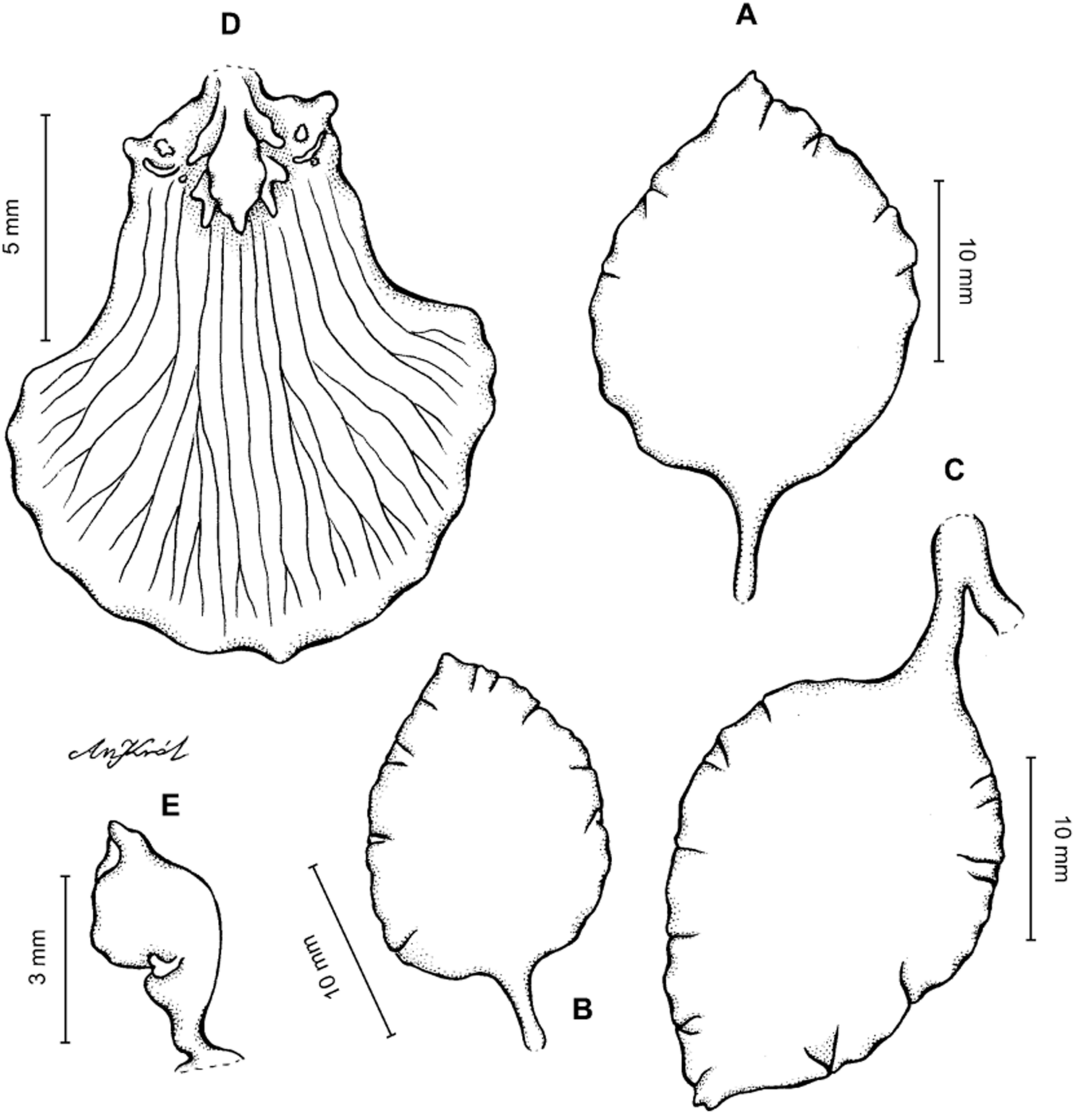
*Cyrtochilum volubile* Poepp. & Endl. (A) Dorsal sepal; (B) Petal; (C) Lateral sepal; (D) Lip; (E) Gynostemium, side view. Drawn by Anna Król from *Poeppig 1636* (W).

Rhizome elongated, stout, creeping. Pseudobulbs 4–5 cm long, commonly aggregated in scattering clusters, narrowly oblong-cylindric, attenuate above, bifoliate, subcompressed, clothed below by a few pairs of evanescent, long, leaf-bearing sheaths. Leaves 30–45 cm long, 3–4 cm wide, linear-oblanceolate, acute or short-acuminate above, more or less long-narrowed below to an often petiole-like, conduplicate base. Scape elongate, climbing or twining, stout, about 500–700 cm long, loosely paniculate above; branches remote (about 19 cm or less apart), simple, loosely few- (3–5) flowered, with the fractiflex rachis about 5–12 cm long. Flowers large, with wide-spreading segments, sepals cinnamon-brown, petals pale cinnamon-brown with snow-white above the middle, lip rose-purple with the basal third white or cinnamon-brown. Floral bracts 7 mm long, small, triangular-ovate, clasping, obtuse. Pedicellate ovary up to 25 mm long. Dorsal sepal clawed; claw 4–5 mm long, narrow, wingless; blade 20–22 mm long, about 12–14 mm wide, suborbicular-ovate, obtuse, distantly undulate. Petals clawed; claw 3–4 mm long, narrow, wingless; blade 16 mm long, 9–10 mm wide, obliquely ovate, acute, distantly undulate. Lateral sepals clawed; about 5 mm long, narrow, wingless, connate basally; blade 20–22 mm long, 12 mm wide, suborbicular-ovate, subacute, slightly oblique, distantly undulate. Lip 12–14 mm long, 12–13 mm wide near the apex, pandurate-obovate, sessile, broadly rounded in front; disc at the base with a small, apically 3-dentate callus surrounded by several free, short tubercles. Gynostemium about 5 mm long, sigmoid, slender, clavate, lateral appendages obscure, digitate (Fig. 98).

*Ecology*: Epiphytic or terrestrial.

*Distribution*: Peru. Alt. 1,480 m.

*Notes*: This species can be easily misidentified with *C. caquetanum*, but both species can be distinguished by the lip callus and lip morphology. In the latter species the lip is almost quadrangular with apically 3-lobed callus and 3 longitudinal keels. Unlike in *C. villenaorum* the lip central callus is apically dentate (vs callus terminating in three lobules forming a small chamber).

*Representative specimens*: PERU. **Huanuco**: Leoncio Prado: Cordillera Azul, 1,480 m, 14 December 1986, *D. & A. Bennett & M. Arias 3706* (MO—Dodson & Bennett, 1989). [**San Martín**]: Near Moyobamba, *A. Mathews 1918* (K). Cassapillo near Cuchero, *E. Poeppig 1636* (W).

***Cyrtochilum leopoldianum*** (Rolfe) Kraenzl., Notizbl. Bot. Gart. Berlin-Dahlem 7: 94. 1917. ≡ *Oncidium leopoldianum* Rolfe, Gard. Chron., ser. 3, 8: 556. 1890. TYPE: Peru. *Linden s.n*. (holotype, K).

Plant large and showy. Rhizome long-creeping, stout according to Kraenzlin. Pseudobulbs more or less distant, about 12.7 cm or less long, narrowly oblong-ovoid to ovoid-cylindric, 1- or 2-leaved, clothed at the base by several pairs of sheaths which are mostly leaf-bearing. Leaves up to 20 cm long and 3 cm wide, on the sheaths larger than those on the pseudobulbs, oblanceolate, gradually narrowed below, acute or acuminate, those on the pseudobulbs oblanceolate, up to 16 cm long and 2 cm wide. Scapes elongate, climbing, paniculate above, many-branched, the existing portion about 250 cm long, clothed at the base by several leaf-bearing sheaths, the branches about equally long and up to 12 cm in length. Flowers rather large, with wide-spreading segments, about 4.5 cm across; sepals and petals white with a crimson-purple lower central portion, lip violet-purple with the basal crest and part of the column yellow. Floral bracts 6 mm long, small, broadly ovate. Pedicellate ovary 25 mm long. Dorsal sepal very shortly clawed, exauriculate at the base, about 23 mm long, about 11 mm wide, lamina broadly elliptical to ovate, obtuse. Lateral sepals very similar, somewhat broader than the dorsal sepal, or narrower. Petals elliptic-ovate, subsessile, subacute, shorter and narrower than the sepals, or broader. Lip much smaller than the sepals and petals, about 13–15 mm long, 7–10 mm wide across the lateral lobes, triangular-lanceolate, lightly 3-lobed near the base, fleshy, subacute to acuminate; lateral lobes rounded; disc at the base with a crest consisting of 3–5 short, fleshy, parallel ridges, the central one highest. Gynostemium short, with a pair of short, rounded, fleshy wings.

*Ecology*: Epiphyte.

*Distribution*: Colombia (Dodson, 1992), Ecuador (Jørgensen & León-Yánez, 1999), Peru.

*Notes:* This species is similar to *C. caquetanum*, but differs in the color of the flowers. In the former species the sepals are brown-yellow in the 2/3 base, the apical third is cream-coloured, petals are brown-yellow in the basal half, the apical part creamy-white, the lip is violet shading into brown-violet in the basal part, keels are white, whereas in the latter species tepals are white with a crimson-purple lower central portion, the lip is violet-purple with the basal crest and part of the column yellow. Additionally, tepals of *C. leopoldianum* are somewhat undulate, and lip subacute to acuminate. Tepals of *C. caquetanum* are flat and lip is obtuse.

*Representative specimens*: PERU. **Loreto**: Mountains east of Moyobamba, transitional country between the savanna woods and the upland evergreen woods, 1,300–1,400 m, *A. Weberbauer 4732* (Schweinfurth, 1961). *Sine loc., J. Linden s.n*. (K).

***Cyrtochilum caquetanum*** P.Ortiz, L.E.Álvarez & A.J.Carrillo, Orquideólogo Supl. 1: 11. 2012. TYPE: Colombia. *Ortiz 1393* (holotype, HPUJ).

Rhizome ascending, short, pseudobulbs separated about 3 cm from each other, leaf sheaths numerous, the oldest dry, the most recent green. Pseudobulbs 8 cm long, 1.3 cm wide, narrow, pyriform, complanate, longitudinally grooved, 2-foliate. Leaves 48 cm long, 3 cm wide, conduplicate, oblong, narrow at the base, acute at the apex, with 7 prominent nerves on underside. Inflorescence up to 190 cm long, axillary, voluble, branched, branches distanced for 15–18 cm, with 2–4 flowers each, peduncle with 3 bracts 6 mm long, 3 mm broad. Flowers showy, sepals in the basal 2/3 with a brown-yellow spot, apical third cream-coloured, petals brown-yellow in the basal half, apical part creamy-white, lip violet shading into brown-violet in the basal part, keels white. Pedicel 3–4 mm long, ovary 3–4 mm long, somewhat thicker. Dorsal sepal 20 mm long, 12 mm wide, obovate with narrow base, basal part centrally thickened. Petals 18 mm long, 10 mm wide, ovate with a narrow base. Lateral sepals 18 mm long, 12 mm wide, oblong-elliptic, basal part centrally thickened. Lip 12 mm long, 10 mm wide, oblong almost quadrangular with rounded angles, apex obtuse, basal part convex, apical part concave; disc with a prominent, broad callus, apex shortly 3-lobed, with a central longitudinal keel at the base and 2 lateral ones oblique. Gynostemium 8 mm long, curved, base narrow, frontally canaliculate in the middle, apically enlarged, stigma concave, almost triangular, with prominent margins, clinandrium with somewhat prominent borders, internally concave.

*Ecology*: Epiphyte.

*Distribution*: Colombia. Alt. 2,000 m.

*Notes*: This species can be confused with *C. leopoldianum* (see notes provided under *C. leopoldianum*).

*Representative specimens*: COLOMBIA. **Caqueta**: Alto de Gabinetes, 2,000 m, J. L. Aguirre, cultivated near Bogotá by A. J. Carrillo, flowered January 2011, *P. Ortiz 1393* (HPUJ); the same loc., September 2009, *P. Ortiz 1340* (HPUJ).

***Cyrtochilum villenaorum*** Christenson, Orchids (West Palm Beach) 71(7): 616–618. 2002. TYPE: Peru. *Christenson 2045* (holotype, K).

Rhizomes ascending, stout. Pseudobulbs 7.5 cm long, 2 cm wide, ovoid, compressed, distant for about 8 cm from each other, subtended by foliaceous bracts, bifoliate. Leaves up to 50 cm long, 3 cm wide, linear, acute. Inflorescence up to 2 m long, pedunculate, paniculate, branches uo to 10 cm long, 3–5-flowered, slightly fractiflex. Floral bracts 0.5 cm long, broadly ovate, acute-acuminate, carinate. Pedicel and ovary 1.7 cm long, terete. Flowers showy, tepals white with mauve-purple centres, lip purple, lip lateral lobes and callus yellow, the U-shaped callus brown, spotted along its crest, central callus projection white-tipped, gynostemium and tabula infrastigmatica greenish white. Dorsal sepal 2.4 cm long, 1.7 cm wide, unguiculate, concave, ovate, obtuse, margins undulate. Petals 2.1 cm long, 1.6 cm wide, unguiculate, concave, ovate, obtuse, margins undulate. Lateral sepals 2.5 cm long, 1.8 cm wide, unguiculate, concave, ovate-deltoid, obtuse, margins undulate. Lip 1.7 cm long, 1.9 cm wide, 3-lobed, sessile, lateral lobes suborbicular, obtuse-rounded, convex; middle lobe transversely semiorbicular, rounded-obtuse; callus biseriate, raised U-shaped ridge with a central oblong callus, central callus terminating in three lobules forming a small chamber. Gynostemium 8 mm long, sigmoid.

*Ecology*: Epiphyte.

*Distribution*: Peru.

*Notes*: This species differs from *C. volubile* in the flower coloration, shortly-clawed sepals and the callus form. Flowers of *C. volubile* have cinnamon brown sepals, petals being cinnamon brown in the basal third and pale-pinkish in the apical part, the central callus is broader and terminates in few small teeth. While in *C. villenaorum* the blade of sepals begins below the column apex, in *C. volubile* the claw is elongate, so the blade begins above column apex causing open gap between dorsal sepal and petals.

*Representative specimen*: PERU. **San Martin**: South of Moyobamba, February 2002, *E.A. Christenson 2045* (K).

***Incertae Sedis***

***Cyrtochilum rostratum*** Schltr., Repert. Spec. Nov. Regni Veg. Beih. 27: 110. 1924. TYPE: Colombia. *Hopp 197* (B†). ≡3 *Oncidium rostratum* (Schltr.) Garay, Taxon 19: 444. 1970.

Rhizome short. Roots filiform, flexuose, glabrous. Pseudobulbs 5–5.5 cm long, 2–2.5 cm wide, ovoid, compressed, ancipitous, unifoliate, laterally ensheathed with foliaceous sheaths. Leaves suberect, narrowly ligulate, subacute, up to 20 cm long, 2.7 cm wide, coriaceous. Inflorescence voluble, distantly shortly racemose, up to 240 cm long. Pedunce rigid, erect, up to 50 cm long, distantly few-sheathed, branches up to 8 cm long, laxly 2–3-flowered, with 2–3 sheaths at the base. Pedicellate ovary 15 mm long, glabrous. Flowers greenish-brown. Sepals patent, acuminate, apex recurved, margin undulate. Dorsal sepal 12 mm long, subsessile, ovate-lanceolate, acuminate, apex recurved, margin undulate. Petals 16 mm long, obliquely elliptic-lanceolate, acuminate, margin undulate, unguiculate. Lateral sepals 18 mm long, unguiculate, shortly connate at the base. obliquely lanceolate, acuminate, apex recurved, margin undulate. Lip 13 mm long, 6.5 mm wide, deflexed-recurved in the middle, base ovate, subcordate-rounded, apical part linear, apex subdilated, obtuse; callus bilobulate, keels 2, forming a “V”. Gynostemium sigmoid-curved.

*Ecology*: Epiphytic.

*Distribution*: Colombia. Alt. 3,500 m.

*Notes*: We have seen no material of this species. Based on Schlechter’s description alone, especially very spare description of the lip callus, it is difficult to speculate the current status of *C. rostratum*.

*Representative specimen*: COLOMBIA. [**Nariño**]: Auf Bãumen auf dem Vulkan Galeras bei Pasto, 3,500 m, April 1922, *W. Hopp 197* (B†).

## Discussion

Neubig et al. (2012) published a comprehensive phylogenetic study on oncidioid orchids. The authors examined sequences of seven DNA regions (both nuclear and plastid) for a set of 122 species, and five DNA regions (nuclear and plastid) for 736 individuals, representing cumulatively about 590 species. They premised that the evolution of DNA fragments can be extrapolated with the phylogeny of species and based on this assumption they propounded a new classification of Oncidinae. Neubig et al. (2012) recognised *Cyrtochilum* as a genus embracing about 120 species including small-flowered representatives of *Buesiella*, *Dasyglossum*, *Neodryas*, *Rusbyella*, *Siederella*, and *Trigonochilum* as well as some *Odontoglossum* species (e.g. *O. ramosissimum* Lindl.). Morphologically, *Neodryas* and *Buesiella* are difficult to separate and we believe that representatives of the latter should be incorporated to *Neodryas*. *Cyrtochilum s.str*. can be distinguished from all the genera recognised by Szlachetko et al. (2017) based on the angle between the lip and gynostemium (more or less right angle in *Siederella, Cyrtochilum* and *Trigonochilum*, gynostemium parallel with the lower part of the lip in *Dasyglossum*, *Neodryas* and *Rusbyella*) as well as based on lip form (lip unguiculate and larger than tepals in *Siederella*, sessile and much smaller than tepals in *Cyrtochilum s.str*.). While the lip of small-flowered representatives of *Trigonochilum* is triangular-cordate in outline, the lip of *Cyrtochilum s.str*. is hastate, pandurate to oblong-ovate or ligulate. In *Dasyglossum*, which is another small-flowered genus, the lip callus consists of fleshy, parallel, adjoining torus while in *Trigonochilum* the lip is ornamented with complex callus usually composed of large, irregular mass of tissue. *Neodryas* and *Rusbyella* can be distinguished based on the gynostemium and the lip form—digitate projections on each sides of the stigma are observed in *Neodryas* (vs very obscure projections or no projections in *Rusbyella*) the lip of which is sessile (long-clawed in *Rusbyella*). This topic was previously discussed in details by Szlachetko et al. (2017).

In this article only representatives of *Cyrtochilum s.str*. were included.

## Conclusions

In our opinion, the broad concept of *Cyrtochilum* proposed by Neubig et al. (2012) should be rejected and only pseudobulbous, 1-4-leaved plants characterised by the right angle between the lip and gynostemium, the sessile lip which is hastate, pandurate to oblong-ovate or ligulate and much smaller than tepals should be included in this genus.

The diversity of *Cyrtochilum s.str*. in Northwestern South America was so far poorly recognised. Noteworthy, the vegetative organs are often absent in the type material of numerous species described in the past. We believe that our work is the first in depth step to reveal the actual variation of the genus representatives in the studied area and to conduct more detailed biogeographical and molecular analyses in the future. The deficiency of comprehensive research on morphology of *Cyrtochilum*; and lack of identification key have resulted in common mistakes in the identification of representatives of this genus. The incorrect determination of specimens was noted in numerous herbaria (notes on herbarium labels) as well as in the previously published local floras (photos not corresponding to the species name). These errors in identification of analysed material can led to an incorrect reconstruction of the phylogeny and infrageneric relationships within *Cyrtochilum* and its relatives.

The taxonomic separateness of three species pairs (*Cyrtochilum hastatum*–*C. ionodon*, *C. diceratum*–*C. englerianum* and *C. tetracopis*–*C. monachicum*) requires further studies.

## Supplemental Information

10.7717/peerj.8566/supp-1 Supplemental Information 1 List of type specimens of species included in this study.

## Additional Information and Declarations

### Data Availability

The following information was supplied regarding data availability: The database of holotypes examined is available as a Supplemental File.

### New Species Registration

The following information was supplied regarding the registration of a newly described species: Cyrtochilum steyermarkii (Foldats) Kolan. & Szlach., comb. nov. 77204672-1; Cyrtochilum klabochii Szlach. & Kolan., sp. nov. 77204671-1; Cyrtochilum garayi Szlach. & Kolan., sp. nov. 77204673-1; Cyrtochilum lehmannii Szlach. & Kolan., sp. nov 77204674-1; Cyrtochilum biscellum Szlach. & Kolan., sp. nov 77204675-1; Cyrtochilum koenigerii Szlach. & Kolan., sp. nov. 77204677-1; Cyrtochilum palmatum Szlach. & Kolan., sp. nov. 77204676-1; Cyrtochilum sibundoyense Szlach. & Kolan., sp. nov. 77204678-1.

## References

[ref-1] Bennett DE, Christenson EA (2001). Icones orchidacearum peruviarum.

[ref-2] Bernal R, Gradstein SR, Celis M (2016). Catálogo de plantas y líquenes de Colombia.

[ref-3] Brako L, Zarucchi J (1993). Catálogo de las Angiospermas y Gimnospermas del Perú.

[ref-4] Carnevali G, Gerlach G, Romero-González GA, Hokche O, Berry PE, Huber O (2008). Orchidaceae. Nuevo Catálogo de la Flora Vascular de Venezuela.

[ref-5] Chase MW, Pridgeon AM, Chase MW, Cribb PJ, Rasmussen FN (2009). Subtribe oncidiinae. Genera Orchidacearum, Vol. 5: Epidendroideae (part II).

[ref-6] Cornejo X, Dodson CH (2011). *Sobralia rhizophorae*: a new species of Orchidaceae from the Mangroves in Northwestern Ecuador. Harvard Papers in Botany.

[ref-7] Dalström S (2001). A synopsis of the genus *Cyrtochilum* (Orchidaceae: Oncidiinae): taxonomic reevaluation and new combinations. Lindleyana.

[ref-8] Dalström S, Dodson CH, Luer CA (2010). *Cyrtochilum* Kunth. Flora of Ecuador 225(3). Orchidaceae. Genera Cyrtochiloides-Epibator.

[ref-9] Dalström S (2015). A new and previously misidentified *Cyrtochilum* (Oncidiinae) from the high plains of central Ecuador. Lankesteriana.

[ref-10] Dalström S, Deburghgraeve G (2015). Eine neue grossblütige *Cyrtochilum*-art aus Venezuela; a new large-flowered *Cyrtochilum* from Venezuela. Orchideen Journal.

[ref-11] Dalström S, Deburghgraeve G, Ruíz Perez S (2012). Three new showy but endangered *Cyrtochilum* species (Oncidiinae: Orchidaceae) from Peru. Lankesteriana.

[ref-12] Damián A, Karremans AP (2016). A new species of *Stelis* (Orchidaceae: Pleurothallidinae) from Peru. Systematic Botany.

[ref-13] Damián A, Ormerod P (2016). *Liparis aphylla* (Malaxideae, Orchidaceae), a new leafless record from Peru. PhytoKeys.

[ref-14] De Azevedo C, Van den Berg C (2012). *Prescottia ecuadorensis*: a new species of Prescottia (Cranichidinae, Orchidaceae) from Ecuador. Phytotaxa.

[ref-15] De Stefano RD, Stauffer F, Riina R, Huber O, Aymard G, Hokche O, Berry PE, Meier W (2009). Assessment of vascular plant diversity and endemism in Venezuela. Candollea.

[ref-16] Dodson CH (1992). Checklist of the orchids of the Western Hemisphere; draft.

[ref-17] Dodson CH, Bennett DE (1989). *Oncidium pastorellii* Dodson & Bennett. Icones Plantarum Tropicarum.

[ref-18] Dodson CH, Dodson PM (1980). Icones plantarum tropicarum: Series I, Fascicles 1–4. Orchids of Ecuador.

[ref-19] Dodson CH, Dodson PM (1982). Icones plantarum tropicarum: Series I, Fascicle 5. Orchids of Ecuador.

[ref-20] Dodson CH, Dodson PM (1984). Icones plantarum tropicarum: Series I, Fascicle 10. Orchids of Ecuador.

[ref-21] Dodson CH, Dodson PM (1989). Icones plantarum tropicarum: Series 2, Fascicle 6. Orchids of Ecuador.

[ref-22] Dorr LJ (2014). Flora of Guaramacal (Venezuela): Monocotyledons. Smithsonian Contributions to Botany.

[ref-23] Dunsterville GCK, Garay LA (1959). Venezuelan orchids illustrated.

[ref-24] Dunsterville GCK, Garay LA (1979). Orchids of Venezuela, an illustrated field guide.

[ref-25] Endara L, León-Yánez S (2006). Endemic Ecuadorian orchids: implications for their conservation.

[ref-26] Fernández-Concha GC, Cetzal-Ix W (2012). A new species of *Macroclinium* (Orchidaceae: Oncidiinae) from Andean Venezuela with brief comments on the biogeography of the genus. Phytotaxa.

[ref-27] Foldats E (1968). Contribución a la Orquidoflora de Venezuela. Acta Botanica Venezuelica.

[ref-28] Garay LA (1974). On the systematics of the monopodial orchids II. Botanical Museum Leaflets, Harvard University.

[ref-29] Garay LA, Harling G, Sparre B (1978). Orchidaceae: cypripedioideae, orchidoideae, neottioideae. Flora of Ecuador.

[ref-30] Giraldo G, Dalström S (2012). A new and extraordinary *Cyrtochilum* (Orchidaceae: Oncidiinae) from Colombia. Lankesteriana.

[ref-31] Higgins WE, Dalström S (2011). New ecuadorian *Cyrtochilum* species. Orchids.

[ref-32] Hokche O, Berry P, Huber O (2008). Nuevo catálogo dela flora vascular de Venezuela.

[ref-33] Idárraga-Piedrahita A, Ortiz RDC, Callejas-Posada R, Merello M (2011). Flora de Antioquia. Catálogo de las Plantas Vasculares, vol. 2, Listado de las Plantas Vasculares del Departamento de Antioquia. Programa Expedición Antioquia-2013, Series Biodiversidad y Recursos Naturales.

[ref-34] IUCN (2001). http://www.iucnredlist.org/technical-documents/categories-and-criteria/2001-categories-criteria#categories.

[ref-35] Jørgensen PM, León-Yánez S (1999). Catalogue of the vascular plants of Ecuador. Monographs in Systematic Botany from the Missouri Botanical Garden.

[ref-36] Jørgensen PM, Nee MH, Beck ST (2014). Catálogo de plantas vasculares de Bolivia.

[ref-37] Jørgensen PM, Ulloa Ulloa C (1994). Seed plants of the high Andes of Ecuador: a checklist. Sustainable Development in The Sahel.

[ref-38] Kolanowska M, Szlachetko DL (2014). A new species of *Hirtzia* (Orchidaceae) from Colombia. Systematic Botany.

[ref-39] Kolanowska M, Szlachetko DL, Medina Trejo R (2016). *Telipogon diabolicus* (Orchidaceae, Oncidiinae), a new species from southern Colombia. PhytoKeys.

[ref-40] Kraenzlin F (1917). Cyrtochilum. Notizblatt des Botanischen Gartens und Museums zu Berlin-Dahlem.

[ref-41] Kunth KC (1816). Nova genera et species plantarum.

[ref-42] Königer W (1994). New species of the genera *Cyrtochilum*, *Masdevallia*, *Odontoglossum*, *Oncidium* and *Sigmatostalix*. Arcula, Botanische Abhandlungen.

[ref-43] Königer W (1995). New species of the genera *Cyrtochilum*, *Masdevallia*, *Oncidium* and *Sigmatostalix*. Arcula, Botanische Abhandlungen.

[ref-44] Leopardi C, Carnevali G, Romero-González GA (2012). *Encyclia lopezii* (Orchidaceae, Laeliinae) a new species from Venezuela. Phytotaxa.

[ref-45] Lindley J (1833). The genera and species of orchidaceous plants: part 3.

[ref-46] Lindley J (1838). Sertum Orchidaceum, a wreath of the most beautiful orchidaceous flowers: part 10.

[ref-47] Neubig KM, Whitten WM, Williams NH, Blanco MA, Endara L, Burleigh JG, Silvera K, Cushman JC, Chase MW (2012). Generic recircumscriptions of Oncidiinae (Orchidaceae: Cymbidieae) based on maximum likelihood analysis of combined DNA datasets. Botanical Journal of the Linnean Society.

[ref-48] Ortiz Valdivieso P (2009). Nuevas especies de orquídeas de Colombia. Orquideologia.

[ref-49] Rodríguez EF, Vásquez R, Rojas R, Calatayud G, León B, Campos J (2006). Nuevas adiciones de angiospermas a la flora del Perú. Revista Peruana de Biologia.

[ref-50] Schweinfurth C (1961). Orchids of Peru. Fieldiana Botany.

[ref-51] Szlachetko DL, Kolanowska M (2014a). Notes on taxonomy of the genus *Cyrtochilum* (Orchidaceae, Oncidieae) and description of two new species of the *Cyrtochilum divaricatum*-alliance from Colombia. Wulfenia.

[ref-52] Szlachetko DL, Kolanowska M (2014b). *Cyrtochilum kraenzlinianum* and *Cyrtochilum maasii* spp. nov. (Orchidaceae, Oncidieae) from Colombia. Nordic Journal of Botany.

[ref-53] Szlachetko DL, Kolanowska M (2017a). Materials to the orchid flora of Colombia.

[ref-54] Szlachetko DL, Kolanowska M (2017b). Two new species of *Cyrtochilum* (Orchidaceae, Oncidieae) from Colombia. Journal of the Torrey Botanical Society.

[ref-55] Szlachetko DL, Kolanowska M, Naczk A, Górniak M, Dudek M, Rutkowski P, Chiron G (2017). Taxonomy of *Cyrtochilum*-alliance (Orchidaceae) in the light of molecular and morphological data. Botanical Studies.

[ref-56] Thiers B (2018). Index Herbariorum: a global directory of public herbaria and associated staff.

[ref-57] Trujillo D (2014). Annotated list of orchidaceae types of the Bennett collection at the forestry Herbarium MOL. Lankesteriana.

[ref-58] Turland NJ, Wiersema JH, Barrie FR, Greuter W, Hawksworth DL, Herendeen PS, Knapp S, Kusber W-H, Li D-Z, Marhold K, May TW, McNeill J, Monro AM, Prado J, Price MJ, Smith GF (2018). International Code of Nomenclature for algae, fungi, and plants (Shenzhen Code) adopted by the 19th International Botanical Congress Shenzhen, China, July 2017. Regnum Vegetabile 159.

[ref-59] Van der Pijl L, Dodson CH (1966). Orchid flowers: their pollination and evolution.

[ref-60] Vargas Calderón JC (1965). Orchids of Machupichu. American Orchid Society Bulletin.

[ref-61] Vásquez R, Dodson CH (1982). Icones plantarum tropicarum, series I, orchids of bolivia.

[ref-62] Zelenko H, Bermúdez P (2009). Orchids—species of Peru.

